# Palladium-catalysed regio- and stereo-controlled C-2 β-fluorovinylation of indoles†

**DOI:** 10.1039/d5qo00521c

**Published:** 2025-04-11

**Authors:** Atul K. Chaturvedi, Alastair J. J. Lennox

**Affiliations:** a School of Chemistry, University of Bristol Cantock's Close Bristol BS8 1TS UK a.lennox@bristol.ac.uk

## Abstract

Vinyl-fluorides appended to heterocycles are a broadly underdeveloped family of functionality with potential application in bioactive compounds. Herein, we disclose a C–H functionalisation strategy for the regio- and stereo-controlled synthesis of *Z*-β-fluorovinyl indoles exclusively in the C-2 position. *Z*-Fluorovinyl iodonium salts, which are formed from alkynes through a Ag-catalysed process, engage in a palladium-catalysed C-2 C–H functionalisation of indoles (and pyrroles) to achieve a broad scope of β-fluorovinyl heterocycles in good to excellent yields. Mechanistic studies and product derivatisations are provided.

## Abstract 2

Vinyl-fluorides appended to heterocycles are a broadly underdeveloped family of functionality with potential application in bioactive compounds.

## Graphical abstract

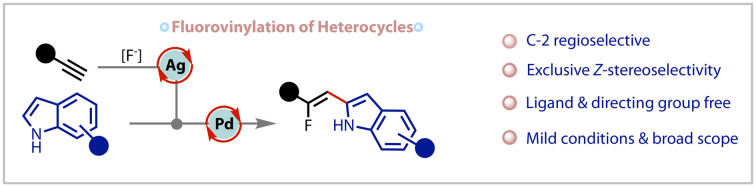

## Introduction

**Fig. 1:**
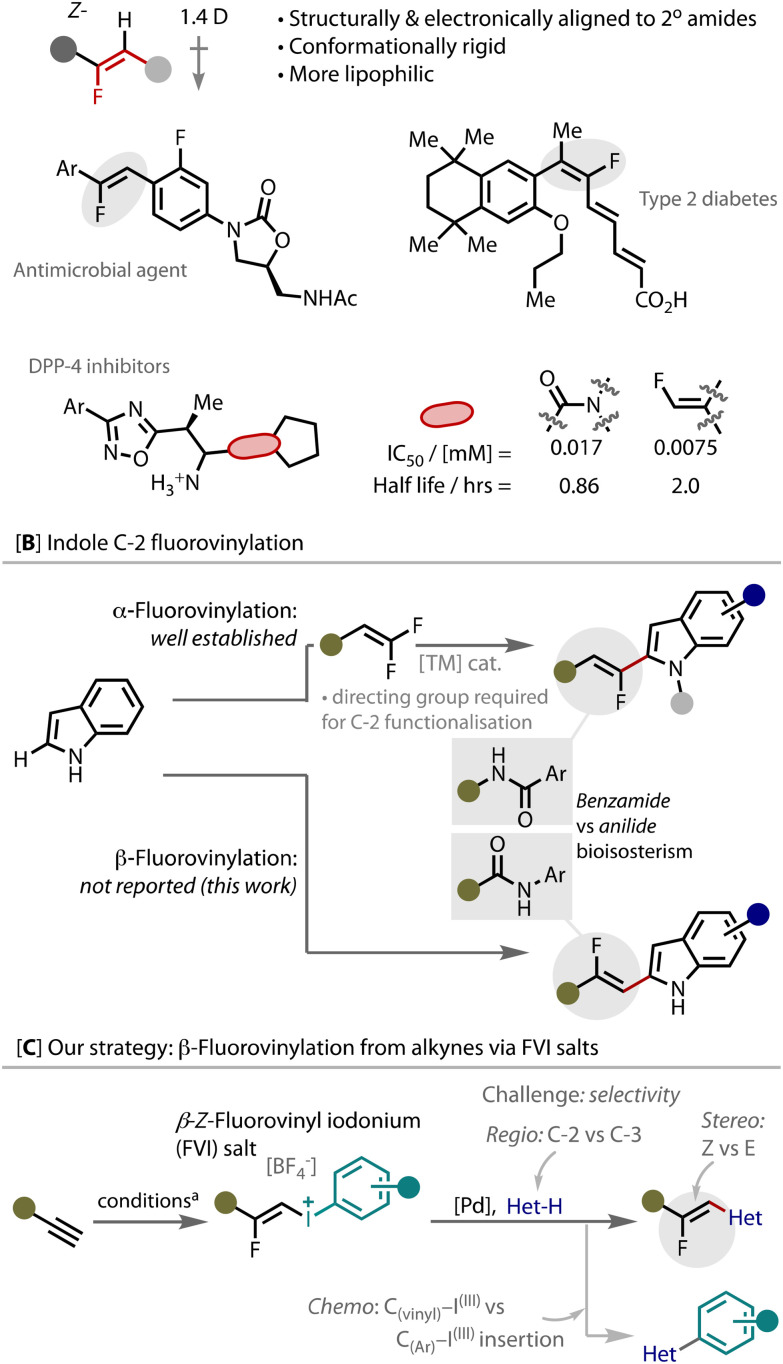
The importance of monofluoroalkenes, the fluorovinylation of indoles at C-2 and our strategy for achieving β-fluorovinylation using fluorovinyl iodonium (FVI) salts. ^*a*^ See ESI† and ref. 15.

Fluorinated moieties are important in drug and agrochemical design,^1,2^ as fluorine has the ability to tune physicochemical and biological properties, such as membrane permeability, lipophilicity, and *in vivo* metabolic stability.^3^ Monofluoroalkenes are a robust, non-nucleophilic peptide bond bioisostere, and a lipophilic peptidomimetic unit used in the design of protease inhibitors to facilitate *cis*/*trans* conformational control.^4^ Several bioactive compounds with various pharmacological activities (anticancer, antimicrobial, anti-HIV, anti-diabetic) bear this motif, Fig. 1A.^5^

Regio- and stereo-controlled routes to monofluoroalkenes remain broadly underdeveloped,^6–8^ which is especially apparent when appended to heteroarenes. There are several methods reported to install the fluoro-vinyl group onto indoles with the fluoride in the *alpha*-position, Fig. 1B; for the most part, *gem*-difluoroalkenes are employed as fluoroalkene surrogates that partner with heteroarenes in transition metal-catalysed coupling reactions.^9–12^ In contrast, there are no methods to install the fluorovinyl group with fluoride in the beta-position. With the orientation of the monofluoroalkene switched, this moiety should be a bioisostere of an anilide, as opposed to a benzamide derivative, Fig. 1B. Indoles that are vinylated in the C-2 position are of notable interest because they are present in active pharmaceutical ingredients and this pattern of unsaturation has been derivatised through pericyclic reactions and macrocyclizations.^13^ Therefore, the development of a method for direct β-fluorovinylation of indole at C-2 would fill an important area of currently inaccessible chemical space that should create potential important utility.

The methods reported to vinylate indoles at C-2 cannot easily be translated to directly access β-fluorovinyl indoles due to several issues, including the use of directing groups and difficulties in achieving high-stereoselectivity.^14^ Heck-type coupling reactions of indoles have been reported with Michael acceptors,^14*a*^ but they also do not work with fluoroalkenes, as we show later, *vide infra*.

We have recently reported a highly efficient silver-catalysed strategy for the stereoselective synthesis of *Z*-fluorovinyl iodonium (FVI) salts (1) from unactivated alkynes.^15^ These stereo-defined FVI salts are stable tuneable building blocks that can engage in palladium catalysis to create new carbon–carbon bonds, as we,^15^ Hara,^16^ and Novák^17^ have demonstrated. Therefore, we considered whether FVI salts could be effective coupling partners to regio- and stereoselectively deliver the β-fluorovinyl unit to indole, Fig. 1C. We reasoned that a Pd^(II)/(IV)^ cycle could be accessed with the use of FVI salts, which should favour the desired C-2 vinylation,^18^ as opposed to the more common C-3 vinylation that is readily accessed through the Pd^(0)/(II)^ Fujiwara–Moritani reaction.^13^ The key challenges associated with this strategy are to maintain exclusive regio- and stereoselectivity, and achieve chemoselectivity by avoiding any competitive arylation *via* cleavage of the Ar–I^(III)^ bond.^18–20^ With these objectives in mind, we now report on the development of such a process.

## Results and discussion

We initiated our study by optimising the C-2 β-fluorovinylation of *N*-methyl indole 2a, using mesitylene substituted iodane 1a as coupling partner. We strategically selected the mesityl (Mes) substituted iodonium salt due to the steric bulk around the hyper-valent iodine^(III)^, which should aid selective cleavage of the C_(vinyl)_–I^(III)^ bond and reduce any competing arylation through cleavage of the C_(Ar)_–I^(III)^ bond. A wide range of copper and palladium salts were evaluated as catalysts in the reaction (see ESI† for screening data, and Table 1 for a selected summary). These screening efforts exposed the ability of PdBr_2_, (CH_3_CN)_2_PdCl_2_ and Pd(OCOCF_3_)_2_ to catalyse this transformation (entries 1–3), but they proved to be less effective than Pd(OAc)_2_, with the expected product 3a obtained in 54% yield in acetic acid at room temperature (Table 1, entry 4). The palladium catalyst delivered the β-fluorovinyl unit exclusively to the C-2 position of indole, with no evidence of any reaction at C-3, and with complete retention of stereochemistry (confirmed by 2D NMR and coupling constant analysis).

**Table 1:** Optimization of reaction conditions^a^

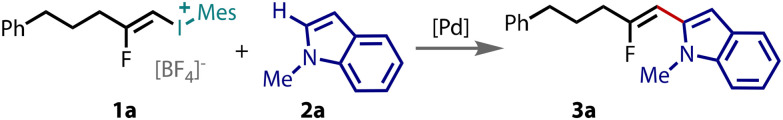			
Entry	Catalyst (*x* mol%)	Solvent	Yield^b^
1	PdBr_2_ (5)	AcOH	50
2	(CH_3_CN)_2_PdCl_2_ (5)	AcOH	30
3	Pd(OCOCF_3_)_2_ (5)	AcOH	32
4	Pd(OAc)_2_ (5)	AcOH	54
5	Pd(OAc)_2_ (5)	DCE	29
6	Pd(OAc)_2_ (5)	MeOH	12
7	Pd(OAc)_2_ (5)	HFIP	35
8	Pd(OAc)_2_ (5)	EtOAc	64
9	Pd(OAc)_2_ (10)	EtOAc	65
10	Pd(OAc)_2_ (2)	EtOAc	42
11^c^	Pd(OAc)_2_ (5)	EtOAc	74
12^d^	Pd(OAc)_2_ (5)	EtOAc	68
13^e^	Pd(OAc)_2_ (5)	EtOAc	80

a Reaction conditions: 1a (0.1 mmol), 2a (0.1 mmol), Pd(OAc)_2_ (5 mol%) in 1 mL solvent at room temperature for 12 h.

b ^19^F NMR yield using 4,4′-difluorobiphenyl as internal standard.

c Reaction performed at 50 °C for 4 h.

d 1.5 equiv. 1a.

e 1.5 equiv. of 2a.

The corresponding ketone was formed as a side-product when acetic acid was used as a solvent, which could be decreased by employing a suitably dry solvent. DCE, methanol and HFIP each afforded lower yields (entries 5–7), however, EtOAc improved the yield to 64% (entry 8). Further screening of the catalyst loading, temperature and equivalents of coupling partner revealed that 5 mol% catalyst loading was adequate (entries 8 *vs*. 9 *vs*. 10), 50 °C was optimum (entry 11) and a slight excess of indole led to the highest yield of 3a (entries 12 *vs*. 13). Different arenes in the iodonium salts were trialled under these optimal conditions. The influence of electronics was found to be significant, as a large range of yields were observed for different *para*-substitution (see ESI† for details) however, the bulky mesityl displayed the best balance of yield and selectivity, and hence was retained for subsequent studies.

**Fig. 2:**
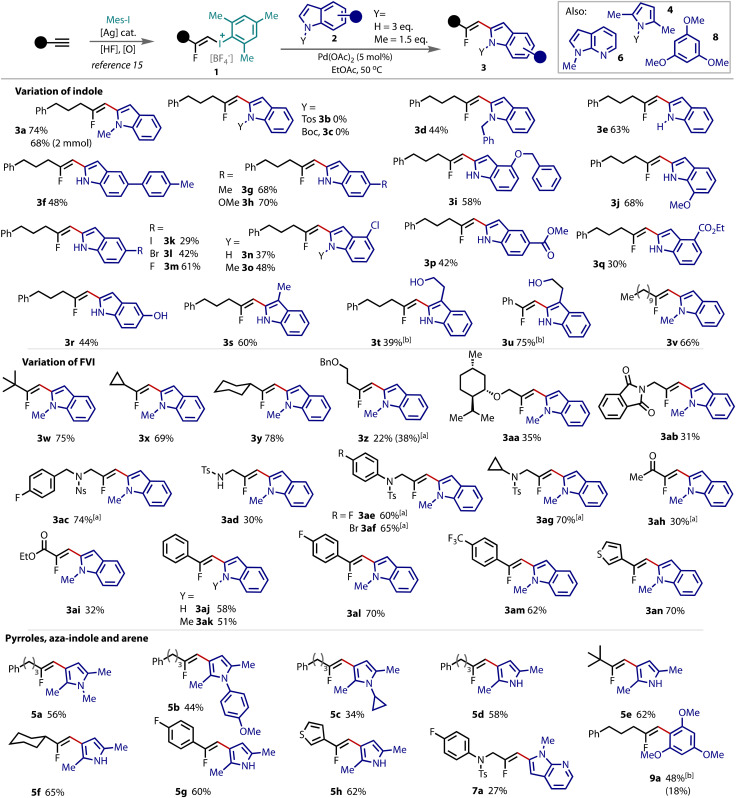
Isolated yields given, with ^19^F NMR yields given in parentheses. Reactions conducted on a 0.1 mmol scale of FVI 1 with either *N*–Me indole (0.15 mmol, 1.5 equiv.) 2a or *N*–H indole (0.3 mmol, 3 equiv.) 2b and Pd(OAc)_2_ (5 mol%) in EtOAc at 50 °C for 2–6 h. ^*a*^ Reaction ran at room temperature (20 °C). ^*b*^ Reaction performed with Cu(OTf)_2_ (10 mol%) in DCM from 0 °C–rt for 30 minutes.

We proceeded to examine the generality of the substrate scope with respect to both FVI 1 and indole 2, Fig. 2. The model substrates were coupled, and product 3a isolated in very good yield, and without an appreciable drop in yield on larger scale. *N*-Tosyl and *N*-boc protected indoles did not react, however, *N*-benzylated and unprotected *N*–H indoles were both suitable coupling partners, as products 3d and 3e, were successfully formed. The unprotected *N*–H indoles led to higher yields when used in an excess (3 equiv.). Substitution on the indole was accommodated with electron withdrawing and donating substituents in different positions around the ring, including aryl (3f), alkyl (3g), alkoxy (3h–j), halogen (3k–o), ester (3p,q) and alcohol (3r) moieties. The tolerance to iodo- and bromo-substitution on the ring is noteworthy considering the opportunity for oxidative addition with a palladium catalyst. Substitution on the C-3 position did not affect the coupling at the C-2 position to give 3s. This result rules out a mechanism that involves initial vinylation at C-3 with a subsequent migration to C-2.^19^ The natural product, tryptophol, was found to transform better using a copper catalyst to selectively yield the C-2 fluorovinylated indoles 3t and 3u.

The generality of the reaction was then explored with different FVIs (1). Although the *N*–Me indole was used for this scope, the unprotected *N*–H indoles (3 equiv.) work comparably well under the conditions (*cf*. 3a*vs*. 3e). Cyclic, acyclic and (hetero)aryl FVIs were transformed efficiently under the optimised conditions, delivering the corresponding β-fluorovinyl indoles (3v–am) in moderate to very good yields. Ethers (3z,aa) and amines (3ab–ag) were tolerated, several of which furnished the corresponding products in better yields at room temperature, rather than elevated temperatures. Ketone 3ah and α,β-unsaturated ester 3ai were both compatible with this Pd-catalysed strategy, as were styrenyl-FVIs 3aj–am and thienyl containing 3an, which were all transformed successfully to β-fluorovinyl indoles.

The suitability of pyrroles (4) as coupling partners in place of indoles was then scrutinised under the catalytic conditions, Fig. 2. The use of unsubstituted *N*-methyl pyrrole created an inseparable mixture of compounds (see ESI† for details) due to the high reactivity of this heterocycle, however, the dimethylated *N*-methyl pyrrole (4a) provided the 3-fluorovinylated pyrrole 5a in good yield. Variation of the *N*-substituent to an arene or cyclopropyl group successfully led to the desired products 5b and 5c, respectively. The unprotected N–H pyrrole 5d fared well under the optimised conditions. Acyclic and cyclic alkyl, styrenyl and thienyl FVIs were all competent coupling partners that furnished the β-fluorovinyl pyrroles in good yields (5e–5h). Aza-indole 6 delivered the desired coupled product 7a, but an enhanced temperature of 90 °C was required. Trimethoxybenzene 8 gave the fluorovinylated arene 9a in enhanced yield using copper catalysis in place of palladium.

**Fig. 3:**
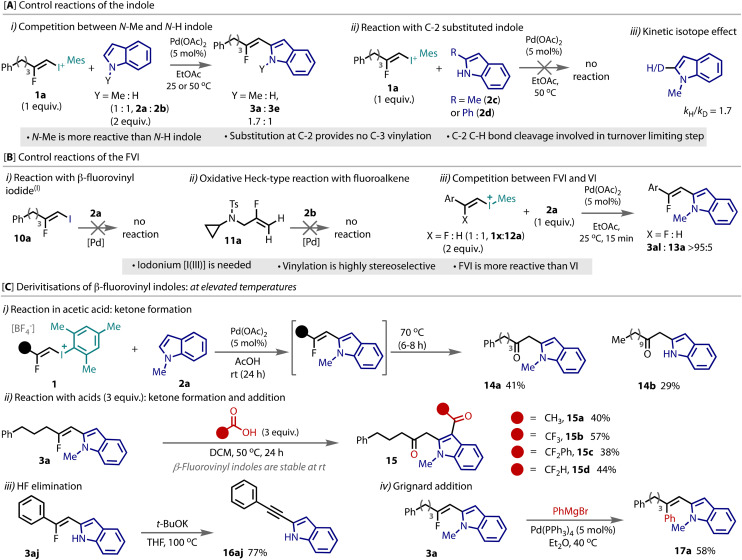
Mechanistic control reactions for β-fluorovinyl indole synthesis and preliminary derivatisation studies.

To gain insight into the reactivity, we conducted a series of control experiments. The *N*–Me (2a) and unprotected *N*–H (2b) indoles were competed for limiting FVI 1a, Fig. 3Ai, which revealed the *N*–Me indole to be more reactive. When the indole was substituted in the C-2 position with either a methyl (2c) or phenyl (2d) group, there was no reaction, neither to a C-2 fluorovinylated species nor the C-3 fluorovinylated product, Fig. 3Aii. This evidence further excludes a mechanism that involves initial reaction at the C-3 position. Independent yield measurements at partial conversion for both indole 2a and [^2^H]_0.7_-2a revealed a kinetic isotope effect of 1.7 at the C-2 position, Fig. 3Aiii, indicating cleavage of this bond is involved in the turnover limiting step.

Concerning the FVI coupling partner, the β-fluorovinyl iodide(i) (10a) was tested in place of the β-fluorovinyl iodonium (I(iii)) salt 1a, Fig. 3Bi. This I(i) building block was unreactive under the conditions. When a fluoroalkene 11a was reacted under typical oxidative Heck conditions, there was also no reaction, Fig. 3Bii. Together, this evidence demonstrates the absolute necessity for a hypervalent I(iii) and that other, Heck-type conditions are not suitable to install fluorinated alkenes. The vinyl iodonium(vi) 12a was reacted in place of the fluoro-vinyl iodonium (FVI) 1j, which successfully led to the desired product, see ESI† for details. However, when the two were in competition for limiting indole 2a, Fig. 3Biii, only the product derived from the fluorinated FVI 12a was observed, thus indicating the superior reactivity of this coupling partner.

A Pd(0)/(ii) cycle that proceeds through oxidative addition, indole coordination, migratory insertion and β-hydride elimination was considered. However, preliminary kinetic studies and reaction analysis using Pd(0) pre-catalysts revealed greatly attenuated reaction conversion and lower yields compared to the use of Pd(ii)(OAc)_2_ (see ESI†). In addition, this mechanism was deemed unlikely considering the tolerance to aryl-bromides, as well as the necessity to proceed through a highly unfavourable anti-β-hydride elimination step. Two *E*-FVIs were tested to determine the stereochemistry of the coupling, see ESI,† and a single stereoisomer, aligned to the stereochemistry in the starting material was observed, indicating a stereospecific, or a highly stereoselective, vinylation. The combined evidence provided therefore supports a Pd(ii)/Pd(iv) catalytic cycle that involves electrophilic C-2 palladation, oxidative addition and reductive elimination, which is consistent with that reported for indole arylation.^18^

The reactivity of β-fluorovinyl indole was explored. Conducting the Pd-catalyzed coupling reaction in acetic acid solvent initially formed the β-fluorovinyl indole (^19^F NMR), but when the temperature was raised to 70 °C hydrolysis to ketones (14a,b) was observed, Fig. 3Ci. This hydrolysis was not observed without strong heating under any other conditions (see ESI†). When β-fluorovinyl indoles (3) were heated with carboxylic acids in reagent quantities (3 equiv.), as opposed to solvent-level quantities, the corresponding diketones (15a–d) were afforded in moderate yields, Fig. 3Cii. Dehydrodefluorination of 3aj was achieved to afford the alkyne 16aj in excellent yield with *t*-BuOK in THF,^9^Fig. 3Ciii. The defluorinative coupling of 3a under palladium catalysis with a Grignard reagent (PhMgBr) delivered arylated product 17a in good yield,^21^Fig. 3Civ.

## Conclusions

In summary, we have developed a Pd(ii)-catalysed C–H fluorovinylation of indoles and other pyrroles. This reaction proceeds with exquisite stereo-selectively under ligand-free, directing-group-free and base-free conditions from fluorovinyl iodonium salts, which themselves are formed easily from alkynes in a single silver-catalysed process. The reaction proved to be general, as a wide range of heteroarenes and *Z*-FVIs were successfully employed in the reaction.

## Conflicts of interest

There are no conflicts to declare.

## Supplementary Material

QO-012-D5QO00521C-s001

## Data Availability

The data supporting this article have been included as part of the ESI.†
