# Supplementary material for: Palladium-catalysed regio- and stereo-controlled C-2 β-fluorovinylation of indoles
Source: Org Chem Front. 2025 Apr 11;12(16):4410–6. doi: 10.1039/d5qo00521c (PMC12004106; doi:10.1039/d5qo00521c)

Supplementary Information (SI) for Organic Chemistry Frontiers.
This journal is © the Partner Organisations 2025

Pd-catalysed Regio- and Stereo-Controlled C-2 β-Fluorovinylation of Indoles

Atul K. Chaturvedi, Alastair J. J. Lennox*

School of Chemistry, University of Bristol, Cantock's Close, Bristol, BS8 1TS (UK)
Corresponding author email: a.lennox@bristol.ac.uk

Contents

General Experimental Details 4
Techniques 4
Solvents 4
Reagents 4
Chromatography 4
Analysis 4
Compound naming 4
^{19}F NMR Calibration 4
Use of HF reagents (Warning) 5
Optimisation Studies 6
Catalyst optimisation 6
Solvent optimisation 7
Optimisation with additives 8
Optimisation with time, temperature and catalyst loading 8
Optimisation with different arenes in the iodonium salts 9
General Procedures 10
General Procedures 1: Synthesis of *N*-substituted pyrrole 10
General Procedures 2a**:** Synthesis of *Z*-fluorovinyl iodonium salts 10
Conditions A 10
Conditions B 11
General Procedures 2b: Synthesis of *E*-fluorovinyl iodonium salts 12
General Procedures 3a: Synthesis of fluorovinylated indole 12
General Procedures 3b: Synthesis of 2-fluorovinylated tryptophol 12
General Procedures 3c: Synthesis of fluorovinylated Pyrole 13
General Procedures 3d: Synthesis of 2-fluorovinylated trimethoxybenzene 13
General Procedures 4: Synthesis of ketone compound 13
General Procedures 5: Synthesis of di-ketone compound 14
General Procedures 6: Synthesis of 2-(phenylethynyl)-1*H*-indole 14
General Procedures 7: Synthesis of (*Z*)-2-(2,5-diphenylpent-1-en-1-yl)-1-methyl-1*H*-indole 14
General Procedures 8: Reaction with vinyl iodane 15
General Procedures 9: Synthesis of fluoroalkene 15
General Procedures 10a: Reaction with fluoroalkene 15
General Procedures 10b: Reaction with fluoroalkene (with oxidant) 16
Reactivity of E-FVI 16
Reaction of indole with E-FVI 16

Reaction of pyrrole with E-FVI 16
Kinetics experiment with Pd(0) and Pd (II) 17
Deuterium Experiment 18
Synthesis of deuterated indole 18
K_H/K_D experiment........ 18
Competitive reaction between fluorovinyl iodane and vinyl iodane 20
Competitive reaction between N-methylindole and 1-*H* indole........ 20
Stability of flurovinylindole........ 21
Plausible mechanism for fluorovinylation of indole 21
Plausible mechanism for diketone compound 21
References 22
Spectral data........ 22
NMR spectra of novel compound........ 61

General Experimental Details

Techniques

Manipulations involving air and moisture-sensitive materials were conducted employing standard Schlenk-line and glovebox techniques, using vacuum lines attached to a double manifold with greaseless J. Youngs valves equipped with an oil pump (0.1 mmHg) under an atmosphere of dry nitrogen. All glassware was dried overnight before use, in a 180 ºC oven and then allowed to cool under vacuum at 0.05 mbar. The removal of solvents in vacuo was achieved using a Büchi rotary evaporator (bath temperatures up to 40 °C) at a pressure of 15 mmHg (diaphragm pump), or at 0.05 mbar (oil pump) on a vacuum line at room temperature. The addition of < 200 µL of liquids was via a Gilson PIPETMAN p20, for larger volumes standard syringe practices were employed.

Solvents

Anhydrous ethyl acetate was purchased from Sigma Aldrich and used under nitrogen atmosphere. Other solvents for anhydrous conditions, were dried by storage over activated molecular sieves (3Å) under nitrogen, with THF (tetrahydrofuran), dichloromethane (DCM), acetonitrile (MeCN), hexane, and diethyl ether (Et2O) dried using an Anhydrous Engineering alumina column drying system situated in the University of Bristol’s chemistry department, and collected into Strauss flasks, using a gas-tight J. Youngs valve, containing activated molecular sieves. Molecular sieves were activated by heating to 300 °C under vacuum for 30 minutes, followed by cooling (still under vacuum). Deuterated solvents for NMR analysis were purchased from Sigma Aldrich.

Reagents

All reagents were purchased from TCI UK, Apollo Scientific, Sigma Aldrich, Alfa Aesar or Fluorochem and used as received.

Chromatography

TLC analysis was performed on Merck Silica gel 60F254 glass-backed plates. Visualisation was achieved by UV fluorescence (254 nm) or staining with basic KMnO4 or PMA. Flash column chromatography was conducted using Merck 60 silica: 230-400 mesh (40-63 µm) or using an automated flash purification system (Biotage Selekt or Buchi Pure C-850 Flashprep) using Biotage Sfar Duo pre-packed columns of size 5 g, 25 g or 50 g.

Analysis

NMR spectra were recorded on Bruker Nano 400 or Bruker Advance III HD 500 cryo spectrometers. Chemical shifts (δ) are quoted in parts per million (ppm), referenced to the residual solvent peak (1H and 13C NMR) and coupling constants (J) are given in Hz. Multiplicities are abbreviated as: s (singlet), d (doublet), t (triplet), q (quartet), m (multiplet) or combinations thereof. NMR shifts for novel compounds have been assigned with the use of the appropriate 2D NMR experiments, such as COSY, HSQC and HMBC. Infrared spectra were recorded using a Perkin Elmer Spectrum Two FTIR spectrometer.

Compound naming

Compound names were generated by ChemDraw Professional 20.0 (PerkinElmer) following IUPAC nomenclature.

^{19}F NMR Calibration

The ^{19}F NMR are internally referenced to $CFCl_3$ and externally to 4,4’-difluoro-biphenyl as internal standard (with 16 scan and 10s relaxation delay)

Use of HF reagents (Warning)

The hazards of hydrogen fluoride solutions are well-categorised. Therefore, personal protection is of utmost importance. It is advised to wear two pairs of nitrile gloves when handling, and if the gloves come into contact with HF, they are removed immediately, and the area affected is washed thoroughly with water, then with Hexafluorine solutionTM. Calcium glucanoate gel is applied to the area and medical attention is sought. It is advised that Hexafluorine solutionTM and calcium glucanoate gel is kept nearby.

If HF has spilled on gloves:

Note: Time is of the essence as exposure to HF is a life-threatening emergency.

• Immediately remove gloves and wash area thoroughly with water

• If any HF has penetrated through gloves, then rub calcium glucanoate gel into the area for several minutes, reapplying once an hour for several hours.

If HF has been spilled on skin:

• Immediately wash the area with hexafluorine solution and large quantities of water for 5 minutes • Then rub calcium gluconate gel into the area for several minutes

• Monitor area for 15 minutes and if redness and swelling develop, proceed to the closest A&E.

Optimisation Studies

Catalyst optimisation

To a 10 mL dried Schlenk tube equipped with a stirrer bar was charged with catalyst (0.005 mmol, 0.05 equiv), *N*-methylindole (13.12 mg, 0.1 mmol, 1.0 equiv.), and fluorovinyliodane (0.1 mmol, 1.0 equiv., 49 mg). Solvent 1mL was added and reaction was allowed to stir at room temperature. After 12h, reaction mixture was diluted with ethyl acetate 4mL and filtered through a short pad of celite and transferred into a separating funnel. Distilled water (4 mL) was added to the separating funnel and the aqueous and organic layers were separated, and the aqueous phase was extracted with ethylacetate (3 x). The combined organic layers were evaporated to ~1-2 mL volume, and 4,4'-difluoro-1,1'-biphenyl was added. The mixture was analysed by ^{19}F NMR to determine NMR yields by integration relative to 4,4'-difluoro-1,1'-biphenyl.

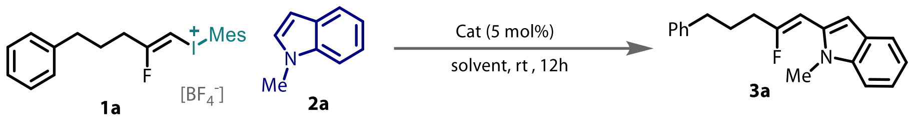

Entry	Catalyst (x mol%)	Solvent	Yield[b]
1	$Pd(OAc)_2$ (5)	AcOH	54
2	$PdBr_2$ (5)	AcOH	50
3	$(CH_3CN)_2PdCl_2$ (5)	AcOH	30
4	$Pd(OCOCF_3)_2$ (5)	AcOH	32
5	PEPSI (5)	AcOH	nr
6	$Pd(dppf)Cl_2$ (5)	AcOH	10
7	$Pd_2(dba)_3$ (5)	EtOAc	23
8	$Pd(PPh_3)_4$ (5)	EtOAc	50
9	$Cu(OTf)_2$ (10)	EtOAc	13
10	$Cu(OTf)_2$ (10)	DCE	14

*[a] Reaction conditions: **1** (0.1mmol), **2** (0.1mmol), catalyst (5 mol%) in 1 mL solvent at room temperature for 12 h. [b] ^{19}FNMR yield using 4,4'-difluorobiphenyl as internal standard.*

Solvent optimisation

To a 10 mL dried Schlenk tube equipped with a stirrer bar was charged with $Pd(OAc)_2$ (0.005 mmol, 0.05 equiv), *N*-methylindole (13.12 mg, 0.1 mmol, 1.0 equiv.), and fluorovinyliodane (0.1 mmol, 1.0 equiv., 49 mg). Solvent 1mL was added and reaction was allowed to stir at room temperature. After 12h, reaction mixture was diluted with ethyl acetate 4mL and filtered through a short pad of celite and transferred into a separating funnel. Distilled water (4 mL) was added to the separating funnel and the aqueous and organic layers were separated, and the aqueous phase was extracted with ethylacetate (3 x). The combined organic layers were evaporated to ~1-2 mL volume, and 4,4'-difluoro-1,1'-biphenyl was added. The mixture was analysed by ^{19}F NMR to determine NMR yields by integration relative to 4,4'-difluoro-1,1'-biphenyl.

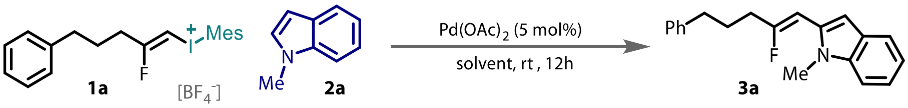

Entry	Catalyst (x mol%)	Solvent	Yield[b]
1	$Pd(OAc)_2$ (5)	DCE	29
2	$Pd(OAc)_2$ (5)	DCM	27
3	$Pd(OAc)_2$ (5)	MeOH	12
4	$Pd(OAc)_2$ (5)	HFIP	35
5	$Pd(OAc)_2$ (5)	MeCN	10
6	$Pd(OAc)_2$ (5)	EtOAc	64

*[a] Reaction conditions: **1** (0.1mmol), **2** (0.1mmol), $Pd(OAc)_2$ (5 mol%) in 1 mL solvent at room temperature for 12 h. [b] ^{19}FNMR yield using 4,4'-difluorobiphenyl as internal standard.*

Optimisation with additives

To a 10 mL dried Schlenk tube equipped with a stirrer bar was charged with $Pd(OAc)_2$ (0.005 mmol, 0.05 equiv), *N*-methylindole (13.12 mg, 0.1 mmol, 1.0 equiv.), fluorovinyliodane (0.1 mmol, 1.0 equiv., 49 mg) and additive (2equiv.). Ethyl acetate 1mL was added and reaction was allowed to stir at room temperature. After 12h, reaction mixture was diluted with ethyl acetate 4mL and filtered through a short pad of celite and transferred into a separating funnel. Distilled water (4 mL) was added to the separating funnel and the aqueous and organic layers were separated, and the aqueous phase was extracted with ethylacetate (3 x). The combined organic layers were evaporated to ~1-2 mL volume, and 4,4'-difluoro-1,1'-biphenyl was added. The mixture was analysed by ^{19}F NMR to determine NMR yields by integration relative to 4,4'-difluoro-1,1'-biphenyl.

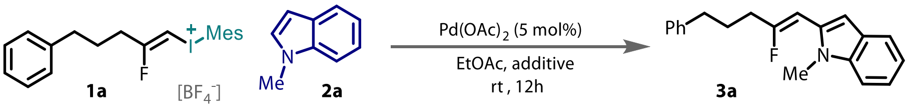

Entry	Catalyst (x mol%)	Additive	Yield[b]
1	$Pd(OAc)_2$ (5)	Acetic acid	60
2	$Pd(OAc)_2$ (5)	TFA	14
3	$Pd(OAc)_2$ (5)	TsOH	11
4	$Pd(OAc)_2$ (5)	Triflic acid	10

*[a] Reaction conditions: **1** (0.1mmol), **2** (0.1mmol), $Pd(OAc)_2$ (5 mol%) in 1 mL ethyl acetate at room temperature for 12 h. [b] ^{19}FNMR yield using 4,4'-difluorobiphenyl as internal standard.*

Optimisation with time, temperature and catalyst loading

Variation in yield with catalyst loading and temperature were monitored by following above standard conditions and the results summarised below.

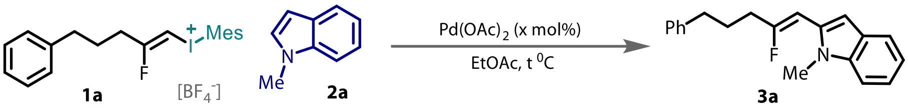

Entry	Catalyst (x mol%)	Time (h)	Temperature (⁰C)	Yield[b]
9	$Pd(OAc)_2$ (10)	12	rt	65
10	$Pd(OAc)_2$ (2)	12	rt	42
11	$Pd(OAc)_2$ (5)	7	40	72
12	$Pd(OAc)_2$ (5)	12	50	55
13	$Pd(OAc)_2$ (5)	4	50	74
13[c]	$Pd(OAc)_2$ (5)	4	50	80
13[d]	$Pd(OAc)_2$ (5)	4	50	69

*[a] Reaction conditions: **1** (0.1mmol), **2** (0.1mmol), $Pd(OAc)_2$ (x mol%) in 1 mL ethyl acetate. [b] ^{19}FNMR yield using 4,4'-difluorobiphenyl as internal standard. [c] 1.5 equiv. **2**. [d] 1.5 equiv. **1***

Optimisation with different arenes in the iodonium salts

To a 10 mL dried Schlenk tube equipped with a stirrer bar was charged with $Pd(OAc)_2$ (0.005 mmol, 0.05 equiv), *N*-methylindole (13.12 mg, 0.1 mmol, 1.0 equiv.) and fluorovinyliodane (0.1 mmol, 1.0 equiv.). ethyl acetate 1mL was added and reaction was allowed to stir at 50 ^{0}C. After 4 h, reaction mixture was diluted with ethyl acetate 4mL and filtered through a short pad of celite and transferred into a separating funnel. Distilled water (4 mL) was added to the separating funnel and the aqueous and organic layers were separated, and the aqueous phase was extracted with ethylacetate (3 x). The combined organic layers were evaporated to ~1-2 mL volume, and 4,4'-difluoro-1,1'-biphenyl was added. The mixture was analysed by ^{19}F NMR to determine NMR yields by integration relative to 4,4'-difluoro-1,1'-biphenyl.

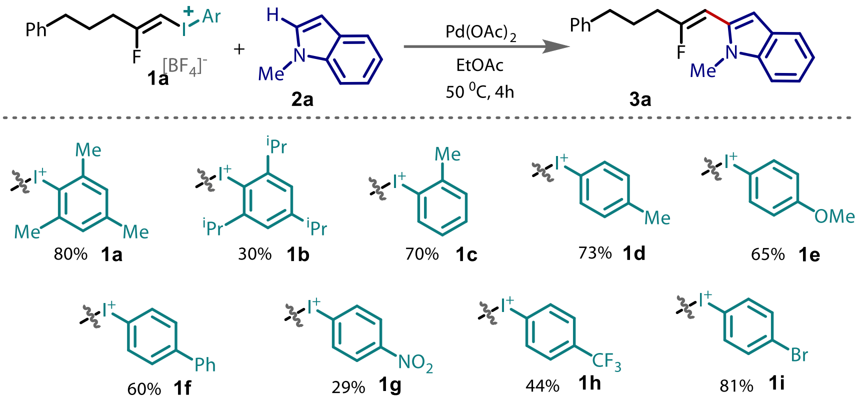

General Procedures

General Procedures 1: Synthesis of *N*-substituted pyrrole

Pyrrole substrates **4a** and **4b** were purchased from Aldrich and used as received. Substrates **4c** and **4d** were synthesized as described.[1]

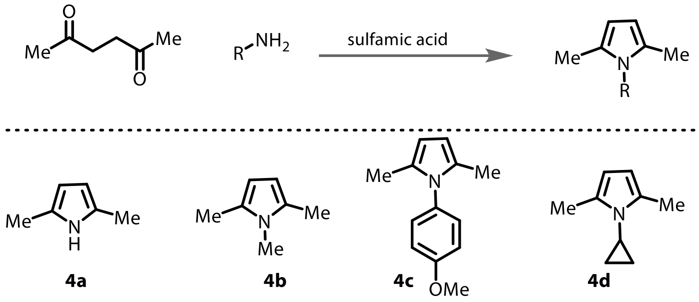

A mixture of hexane-2,5-dione (1.2 equiv, 6 mmol), aniline (1.0 equiv, 5 mmol), and sulfamic acid (0.05 equiv, 0.25 mol) was stirred at room temperature under solvent-free conditions for 30 min. After completion of the reaction, as indicated by thin-layer chromatography (TLC), the reaction mixture was diluted with diethyl ether. The ether layer was washed with brine, dried ($MgSO_4$), and concentrated in vacuo. The residue was purified by silica-gel column chromatography (5% ethyl acetate in hexane) to afford the pure product.

General Procedures 2a: Synthesis of *Z*-fluorovinyl iodonium salts

z-Fluorovinyl iodane were prepared by following two sets of conditions developed in our lab.[2]

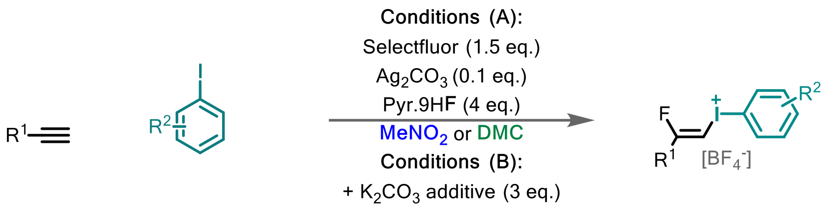

Conditions A

To an HDPE vial equipped with a stirrer bar, was added aryl iodide (1.1 equiv.), Selectfluor (1.5 equiv.), and Ag_2CO_3 (0.1 equiv.). The vial was wrapped in aluminium foil before being placed in a -20 °C cooling bath. Solvent (MeNO2 or dimethyl carbonate) was added in the amount specified to give a 0.125 M concentration of alkyne. Pyridine.9HF (4 equiv.) was added drop-wise, and the reaction was left to stir for 5 minutes. Alkyne (1 equiv.) was added slowly, and the reaction mixture was allowed to warm to room temperature, at which it was stirred for 24 hours. Distilled water (half the reaction volume) was added to the reaction mixture, which was then transferred into a separating funnel, along with a further rinse of the vial with dimethyl carbonate. The aqueous and organic layers were separated, and the aqueous phase was extracted with dimethyl carbonate (2 x). The aqueous phase was slowly poured into a saturated aqueous solution of $NaHCO_3$. The combined organic extracts were washed with a saturated aqueous solution of $NaBF_4$, and twice with distilled water, then evaporated under reduced pressure. To the resulting crude product was added either Et_2O or pentane (10-15 mL per mmol of alkyne added at the start of the reaction). The suspension was sonicated for 10-15 minutes, then carefully decanted (leaving behind the (Z)-FVI). This trituration process was typically repeated 1-3 times. Evaporation of the residual solvent yielded the Z-fluorovinyl iodonium Salt.

Conditions B

To an HDPE vial equipped with a stirrer bar, was added aryl iodide (1.1 equiv.), Selectfluor (1.5 equiv.), K_2CO_3 (3 equiv.), and Ag_2CO_3 (0.1 equiv.). The vial was wrapped in aluminium foil before being placed in a -20 ◦C cooling bath. Nitromethane was added in the amount specified to give a 0.125 M concentration of alkyne. Pyridine.9HF (4 equiv.) was added drop-wise, and the reaction was left to stir for 5-10 minutes. Alkyne (1 equiv.) was added slowly, and the reaction mixture was allowed to warm to room temperature. The procedure from this point onwards is identical to that under "Conditions A".

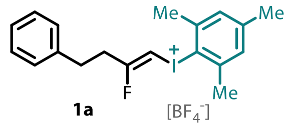
1a

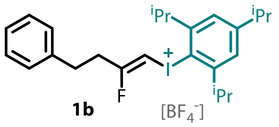
1b

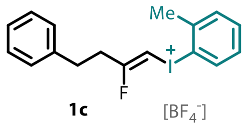
1c

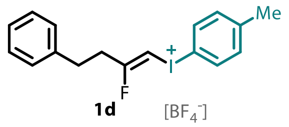
1d

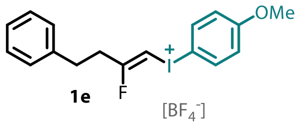
1e

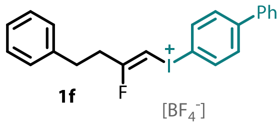
1f

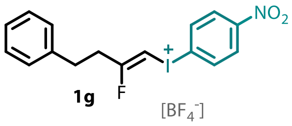
1g

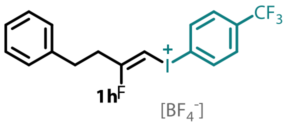
1h

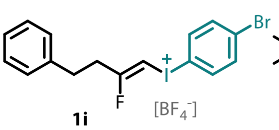
1i

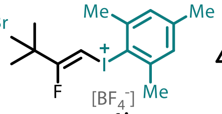
1j

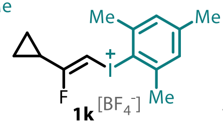
1k

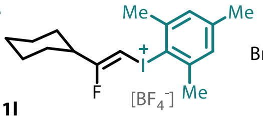
1l

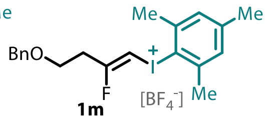
1m

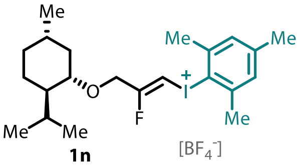
1n

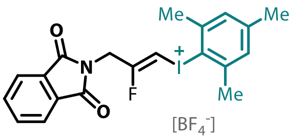
1o

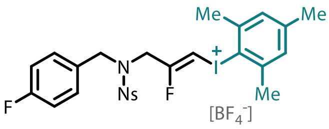
1p

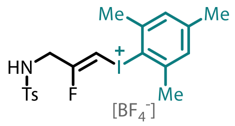
1q

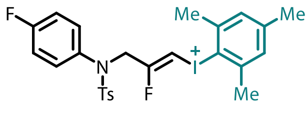
1r

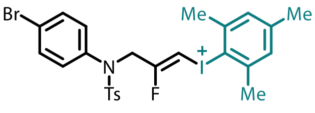
1s

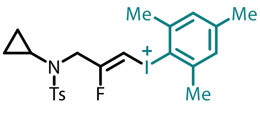
1t

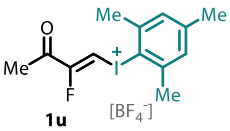
1u

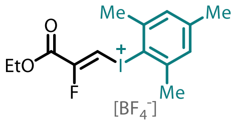
1v

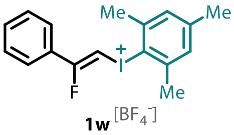
1w

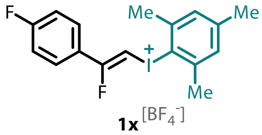
1x

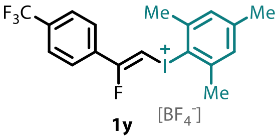
1y

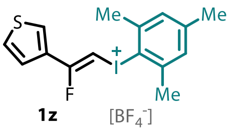
1z

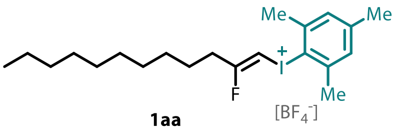
1aa

General Procedures 2b: Synthesis of *E*-fluorovinyl iodonium salts

E-Fluorovinyl iodane were synthesized as described in ref. 3.

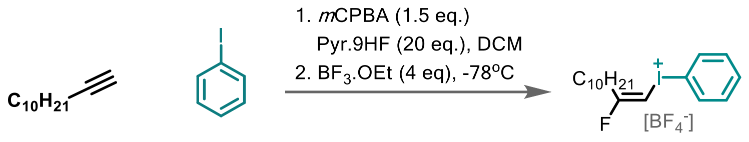

To an HDPE vial equipped with a stirrer bar were added PhI (0.612 g, 3.0 mmol, 1.5 equiv), Py·HF (1.144 g, 40 mmol, 20 equiv), *m*-CPBA (0.688 g, 3.0 mmol, 1.5 equiv), and CH_2Cl_2 (8 mL). The mixture was stirred at room temperature for 3 h, and then alkyne **1** (2.0 mmol, 1.0 equiv) was added at this temperature. After the mixture was cooled to −78 °C, BF_3·Et_2O (1.0 mL, 8.0 mmol, 4 equiv) was added, and the mixture stirred for 10 min. The reaction mixture was warmed to room temperature and stirred for 20 min. The reaction mixture was poured into water (25 mL) and extracted with CH_2Cl_2 (10 mL × 3). The combined organic layer was washed with an aqueous solution (20 mL) of $NaBF_4$ (1.096 g, 10.0 mmol, 5 equiv) and dried over anhydrous Na_2SO_4. After evaporation of the solvent, the residue was submitted to column chromatography on silica gel. Organic compounds containing *m*-chlorobenzoic acid were first eluted with hexane/EtOAc (9:1), and β-fluorovinyliodonium salts were eluted with EtOAc.

General Procedures 3a: Synthesis of fluorovinylated indole

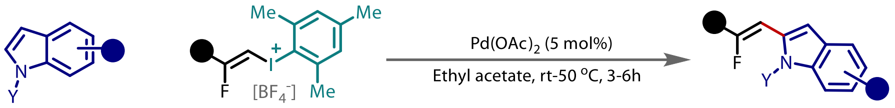

Y = Me (1.5 equiv)
Y = H (3.0 equiv)

To a 10 mL dried Schlenk tube equipped with a stirrer bar was charged with $Pd(OAc)_2$ (0.010 mmol, 0.05 equiv), indole (1.5-3.0 equiv.)[1.5 equiv for *N*-methylindole and 3.0 equiv for N-H indole], and fluorovinyliodane (0.2 mmol, 1.0 equiv.). Dry ethylacetate 2 mL was added and reaction was allowed to stir at rt-50 ^{0}C for 3-6 h. After completion of reaction, mixture was diluted with ethyl acetate 4 mL and filtered through a short pad of celite and transferred into a separating funnel. Distilled water (4 mL) was added to the separating funnel and the aqueous and organic layers were separated, and the aqueous phase was extracted with 5 mL of ethylacetate (3 times). The combined organic layers were evaporated, and the mixture was purified by silica gel column chromatography using pentane/ethylacetate (95:5) to get the pure compound.

General Procedures 3b: Synthesis of 2-fluorovinylated tryptophol

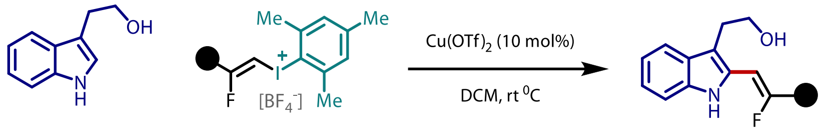

In a 10 mL flame-driedd Schlenk tube equipped with a stirrer bar was added $Cu(OTf)_2$ (0.020 mmol, 0.10 equiv), tryptophol (0.3 mmol, 1.5 equiv.), and DCM 2 mL under argon. The reaction was cooled to 0 ^{0}C and stirred for 5 minutes. Fluorovinyliodane (0.2 mmol, 1.0 equiv.) was added, and the reaction was left to stir for 5 minutes at 0 ^{0}C. The reaction mixture was then allowed to warm to room temperature, at which it was stirred for 30 minutes. After completion of reaction, mixture was diluted with DCM (5 mL) and quenched by the addition of saturated aqueous solution of $NaHCO_3$(5 mL). The aqueous and organic layers were separated, and the aqueous phase was extracted with 5 mL of DCM (3 times). The combined organic layers were dried over Na_2SO_4 and concentrated in vacuo. The mixture was purified by silica gel column chromatography using pentane/ethylacetate (95:5) to afford the desire product.

General Procedures 3c: Synthesis of fluorovinylated Pyrole

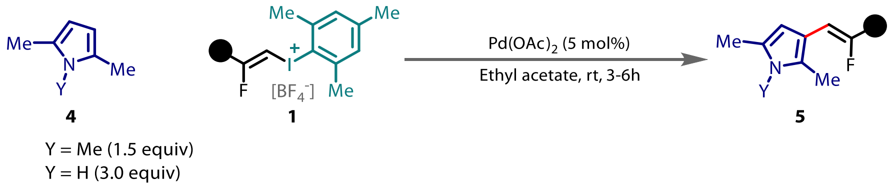

To a 10 mL dried Schlenk tube equipped with a stirrer bar was charged with $Pd(OAc)_2$ (0.01 mmol, 0.05 equiv), pyrrole (1.5-3.0 equiv.)[1.5 equiv for *N*-methylpyrole and 3.0 equiv for N-*H* pyrole]. Dry ethylacetate 2mL was added and reaction was allowed to stir at room temperature for 3-4 h. After completion of reaction, mixture was diluted with ethyl acetate 4 mL and filtered through a short pad of celite and transferred into a separating funnel. Distilled water (4 mL) was added to the separating funnel and the aqueous and organic layers were separated, and the aqueous phase was extracted with 5 mL of ethylacetate (3 times). The combined organic layers were evaporated, and the mixture was purified by neutral alumina column chromatography using pentane/ethylacetate (97:3) to get the pure compound.

General Procedures 3d: Synthesis of 2-fluorovinylated trimethoxybenzene

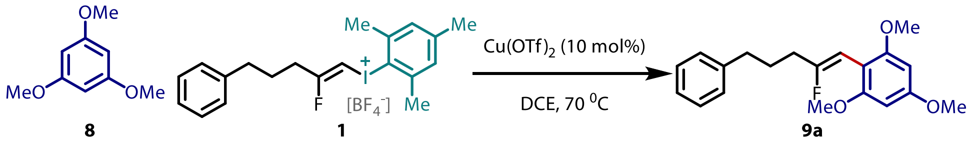

In a 10 mL flame-driedd Schlenk tube equipped with a stirrer bar was added $Cu(OTf)_2$ (0.020mmol, 0.10 equiv), Trimethoxybenzene (0.3 mmol, 1.5 equiv.), Fluorovinyliodane (0.2 mmol, 1.0 equiv.) and DCE 2mL under nitrogen. The reaction was Heated to 70 ^{0}C for 4 hours. After completion of reaction, mixture was diluted with DCM (5 mL) and quenched by the addition of saturated aqueous solution of $NaHCO_3$ (5 mL). The aqueous and organic layers were separated, and the aqueous phase was extracted with 5 mL of DCM (3 times). The combined organic layers were dried over Na_2SO_4 and concentrated in vacuo. The mixture was purified by neutral alumina column chromatography using pentane/ethylacetate (95:5) to afford the desire product.

General Procedures 4: Synthesis of ketone compound

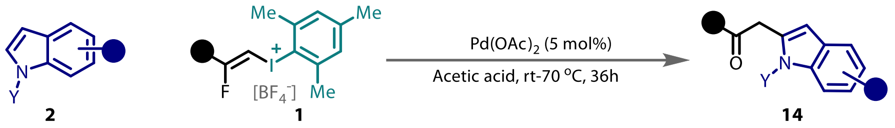

To a 10 mL dried Schlenk tube equipped with a stirrer bar was charged with $Pd(OAc)_2$ (0.01 mmol, 0.05 equiv), indole (1.5-3.0 equiv.)[1.5 equiv for *N*-methylindole and 3.0 equiv for N-H indole], and fluorovinyliodane (0.2 mmol, 1.0 equiv.). Acetic acid 2 mL was added and reaction was allowed to stir at rt for 16 h and then heated at 70 ^{0}C for 8 h. After completion of reaction, mixture was quenched with saturated solution of $NaHCO_3$. Ethyl acetate 10mL was added to the separating funnel and the aqueous and organic layers were separated, and the aqueous phase was extracted with 5 mL of ethylacetate (3 time). The combined organic layers were evaporated, and the mixture was purified by silica gel column chromatography using pentane/ethylacetate (90:10) to get the pure compound.

General Procedures 5: Synthesis of di-ketone compound

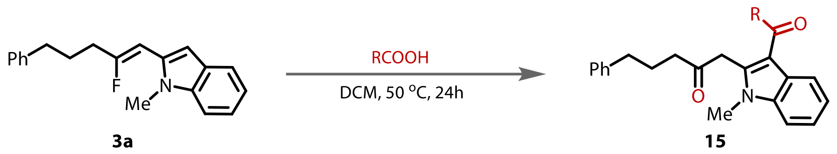

In a pressure tube equipped with stirrer bar fluoro-vinylindole (0.2 mmol, 1 equiv) acid (0.5 mmol, 2.5 equiv) was added and the reaction mixture was allowed to heat at 50 °C for 24 h. After completion of reaction (TLC), the reaction mixture was transferred into round bottom flask and evaporated to get crude product. The crude product was purified by silica gel column chromatography using pentane/ethylacetate (90:10).

General Procedures 6: Synthesis of 2-(phenylethynyl)-1*H*-indole

HF elimination reaction was performed as described in ref. 4.

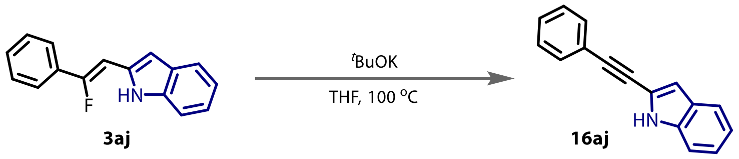

A 10 ml flame-dried pressure tube was charged with *t*-BuOK (68.9 mg, 0.4 mmol) and (*Z*)-2-(2-fluoro-2-phenylvinyl)-1*H*-indole (0.1 mmol) followed by adding anhydrous THF (1.0 ml) through syringe and then closed tightly. After stirring at 100 °C for 24 hours, saturated ammonium chloride (2 ml) was added and the resulting mixture was extracted with dichloromethane (2x5 ml). Removal of the solvent in vacuo and purification of the residue by silica gel column chromatography using pentane/ethylacetate (95:5) afforded the desired product.

General Procedures 7: Synthesis of (*Z*)-2-(2,5-diphenylpent-1-en-1-yl)-1-methyl-1*H*-indole

Coupling reaction was performed as described in ref. 5.

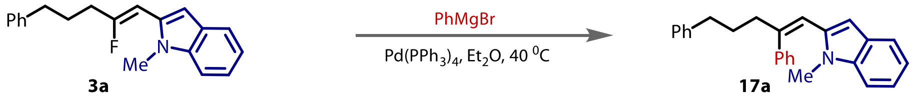

To a solution of fluoro-vinylindole (29.3 mg, 0.1 mmol) and $Pd(PPh_3)_4$ (5.8 mg, 0.005 mmol, 5 mol%) in diethyl ether in a flame-dried Schlenk tube was added dropwise a solution of PhMgBr in THF (0.24 mmol, 2.4 equiv) at room temperature under an argon atmosphere. The mixture was stirred for 2 h at 40 °C. After completion (monitored by TLC) of the reaction, the reaction mixture was quenched with a saturated aqueous solution of NH_4Cl (5 mL) and extracted with ethyl acetate (3 × 10 mL). The combined organic layer was washed with water and brine, then dried over anhydrous Na_2SO_4, filtered, and concentrated under vacuum. The crude residue was then purified by column chromatography on silica gel using pentane/ethylacetate (98:2) as the eluent to afford target product **16a** as a colorless oil (58% yield).

General Procedures 8: Reaction with vinyl iodane

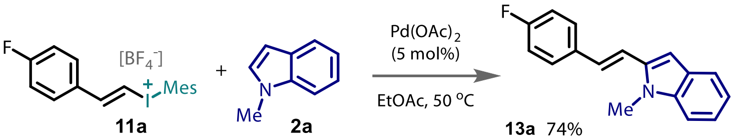

To a 10 mL dried Schlenk tube equipped with a stirrer bar was charged with $Pd(OAc)_2$ (0.005 mmol, 0.05 equiv), *N*-methylindole (0.15 mmol, 1.5 equiv., 19 mg), and vinyl iodane (0.1 mmol, 1.0 equiv., 45 mg). Dry ethylacetate 1 mL was added and reaction was allowed to stir at rt-50 ^{0}C for 3-6 h. After completion of reaction, mixture was diluted with ethyl acetate 4 mL and filtered through a short pad of celite and transferred into a separating funnel. Distilled water (4 mL) was added to the separating funnel and the aqueous and organic layers were separated, and the aqueous phase was extracted with 5 mL of ethylacetate (3 times). The combined organic layers were evaporated, and the mixture was purified by silica gel column chromatography using pentane/ethylacetate (95:5) to get the pure compound.

General Procedures 9: Synthesis of fluoroalkene

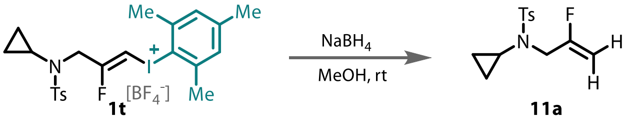

To a 10 mL dried glass vial equipped with a stirrer bar, was added (*Z*)-(3-((*N*-cyclopropyl-4-methylphenyl)sulfonamido)-2-fluoroprop-1-en-1-yl)(mesityl)iodonium BF4 (301 mg, 0.5 mmol, 1 equiv.) and methanol (5 mL). The solution was cooled to 0 °C and sodium borohydride (38 mg, 1 mmol, 2 equiv.) was added. The mixture was allowed to stir for 5 hours before adding water (5 mL) and DCM (10 mL). The aqueous and organic layers were separated, and the aqueous phase was extracted with DCM (3 x). The combined organic extracts were filtered and evaporated under reduced pressure. The resulting crude was subjected to flash-column chromatography (0 to 25% EtOAc in pentane) to afford the product as a white solid (130 mg, 77%).

General Procedures 10a: Reaction with fluoroalkene

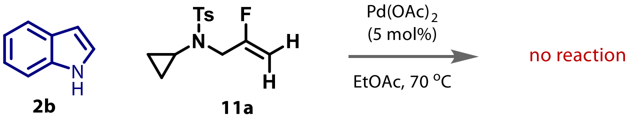

To a 10 mL dried Schlenk tube equipped with a stirrer bar was charged with $Pd(OAc)_2$ (0.005 mmol, 0.05 equiv), indole (0.15 mmol, 1.5 equiv., 17 mg), and *N*-cyclopropyl-*N*-(2-fluoroallyl)-4-methylbenzenesulfonamide (0.1 mmol, 1.0 equiv., 27 mg). Dry ethylacetate 1 mL was added and reaction was allowed to stir at 70 ^{0}C for 12 h. After 12 h, 4,4'-difluoro-1,1'-biphenyl as NMR standard was added. The mixture was analysed by ^{19}F NMR to determine NMR yields by integration relative to 4,4'-difluoro-1,1'-biphenyl.

General Procedures 10b: Reaction with fluoroalkene (with oxidant)

Coupling reaction was performed as described in ref. 7.

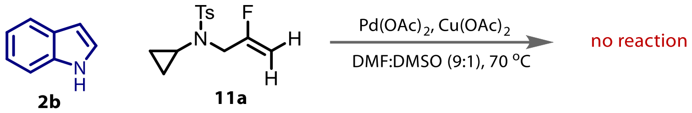

To a 10 mL dried Schlenk tube equipped with a stirrer bar was charged with $Pd(OAc)_2$ (0.010 mmol, 0.05 equiv), copper acetate (0.18 mmol, 1.8 equiv, 33 mg) indole (0.15 mmol, 1.5 equiv., 17 mg), and *N*-cyclopropyl-*N*-(2-fluoroallyl)-4-methylbenzenesulfonamide (0.1 mmol, 1.0 equiv., 27 mg). DMF (0.9 mL) and DMSO (0.1 mL) was added and reaction was allowed to stir at 70 ^{0}C for 12 h. After 12 h, 4,4'-difluoro-1,1'-biphenyl as NMR standard was added. The mixture was analysed by ^{19}F NMR to determine NMR yields by integration relative to 4,4'-difluoro-1,1'-biphenyl.

Reactivity of E-FVI

Reaction of indole with E-FVI

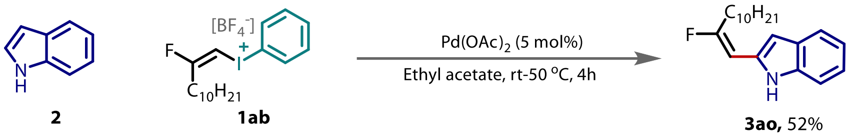

To a 10 mL dried Schlenk tube equipped with a stirrer bar was charged with $Pd(OAc)_2$ (0.010 mmol, 0.05 equiv), indole (0.6 mmol, 3.0 equiv., 70 mg) and E-fluorovinyliodane (0.2 mmol, 1.0 equiv., 104 mg). Dry ethylacetate 2 mL was added and reaction was allowed to stir at rt-50 ^{0}C for 4 h. After completion of reaction, mixture was diluted with ethyl acetate 4 mL and filtered through a short pad of celite and transferred into a separating funnel. Distilled water (4 mL) was added to the separating funnel and the aqueous and organic layers were separated, and the aqueous phase was extracted with 5 mL of ethylacetate (3 times). The combined organic layers were evaporated, and the mixture was purified by silica gel column chromatography using pentane/ethylacetate (95:5) to get **3ao** in 52% yield.

Reaction of pyrrole with E-FVI

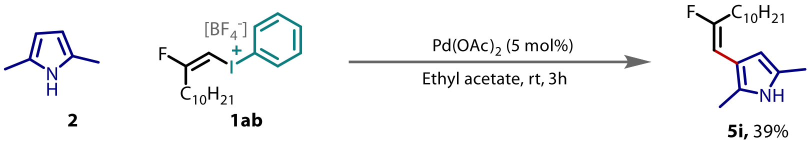

To a 10 mL dried Schlenk tube equipped with a stirrer bar was charged with $Pd(OAc)_2$ (0.010 mmol, 0.05 equiv), pyrrole (0.6 mmol, 3.0 equiv., 57 mg) and E-fluorovinyliodane (0.2 mmol, 1.0 equiv., 104 mg). Dry ethylacetate 2 mL was added and reaction was allowed to stir at rt-50 ^{0}C for 4 h. After completion of reaction, mixture was diluted with ethyl acetate 4 mL and filtered through a short pad of celite and transferred into a separating funnel. Distilled water (4 mL) was added to the separating funnel and the aqueous and organic layers were separated, and the aqueous phase was extracted with 5 mL of ethylacetate (3 times). The combined organic layers were evaporated, and the mixture was purified by silica gel column chromatography using pentane/ethylacetate (98:2) to get **5i** in 39% yield.

Kinetics experiment with Pd(0) and Pd (II)

Kinetics study of reaction were performed at 0.05 mmol scale with $Pd_2(dba)_3$ and $Pd(OAc)_2$ at 25 ^{0}C. Indole (1.0 equiv, 0.05 mmol) and FVI (1.0 equiv, 0.05 mmol) and Pd-catalyst (0.0025 mmol) were added in NMR tube and the data were recorded after every 100 second.

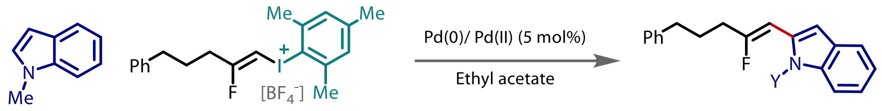

The study revealed that the reaction started immidiately with Pd(0), however became sluggish later on.

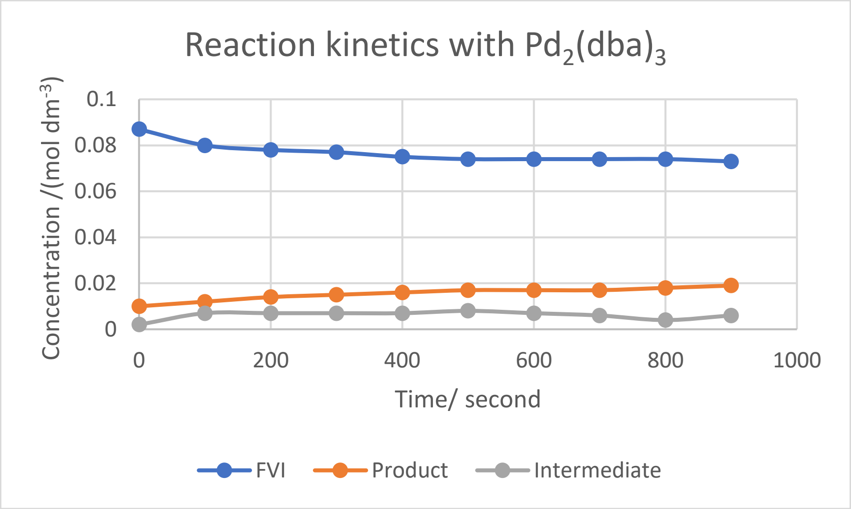

The reaction with $Pd(OAc)_2$ showed significant progress with time.

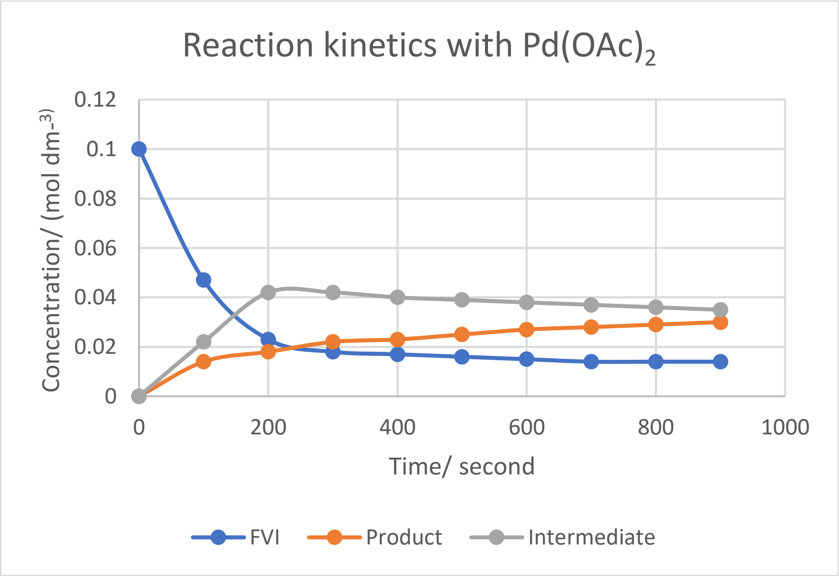

An intermediate was noticed during reaction which get converted into the product when worked up with water.

Deuterium Experiment

Synthesis of deuterated indole

Deuterated indole was synthesized as described in ref. 6.

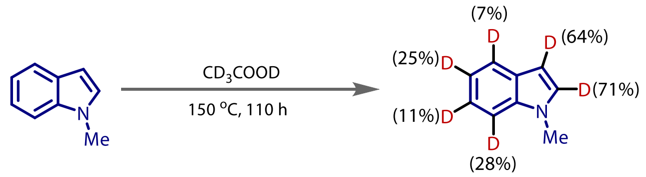

A solution of N-methyl indoles in deuterated acetic acid (CD_3CO_2D) (0.1 M) was heated at 150 °C for 110 h in a sealed tube. The mixture was concentrated under reduced pressure. The residue was purified by column chromatography on silica gel (hexane/EtOAc = 95:5) to give deuterated indoles 84% yield (71% deuteration at C-2 position).

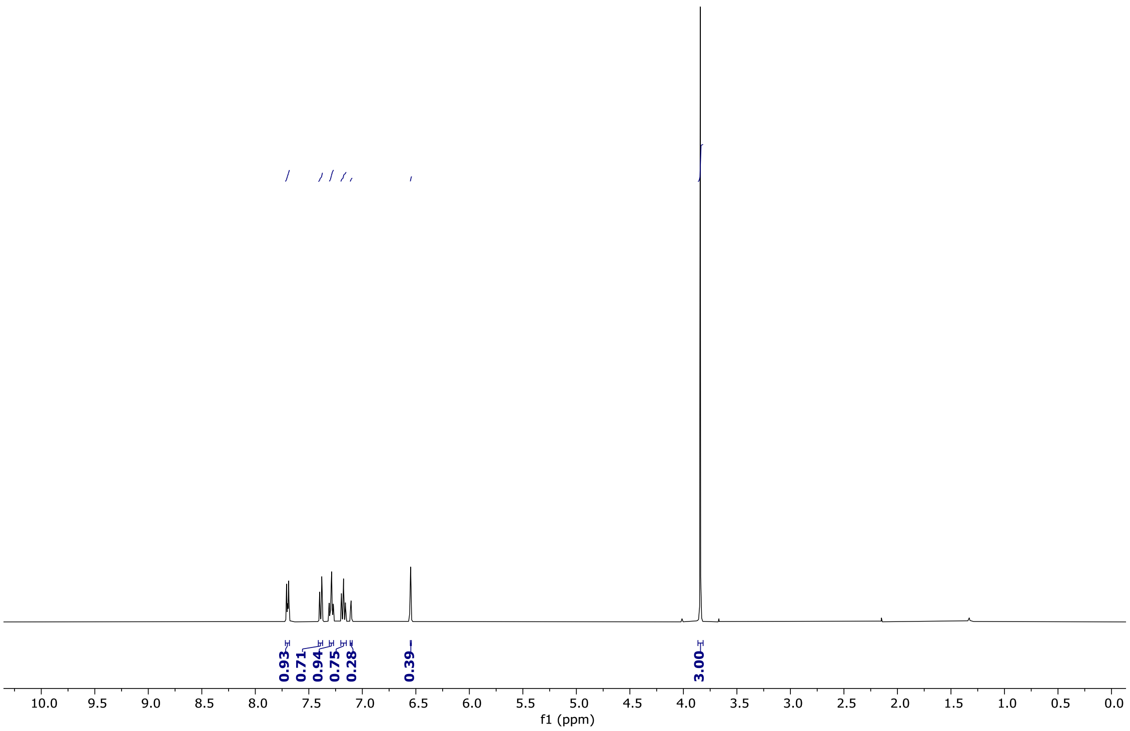

K_H/K_D experiment

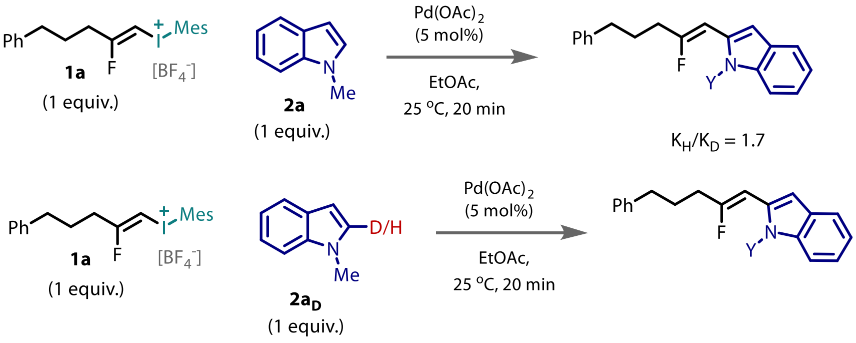

Two different reactions, one with non-deuterated indole and other with deuterated indole were performed in a 10 mL dried Schlenk tube following the standard procedure (general procedure 3a).

After 20 minutes both the reactions were quenched by adding 4 mL of distilled water and aqueous layer was extracted with 4 mL of ethyl acetate (3 times). The combine organic layer was concentrated to 1 mL and ^{19}FNMR yield was recorded separately for both the crude products using 4,4'-difluoro-1,1'-biphenyl as NMR standard.

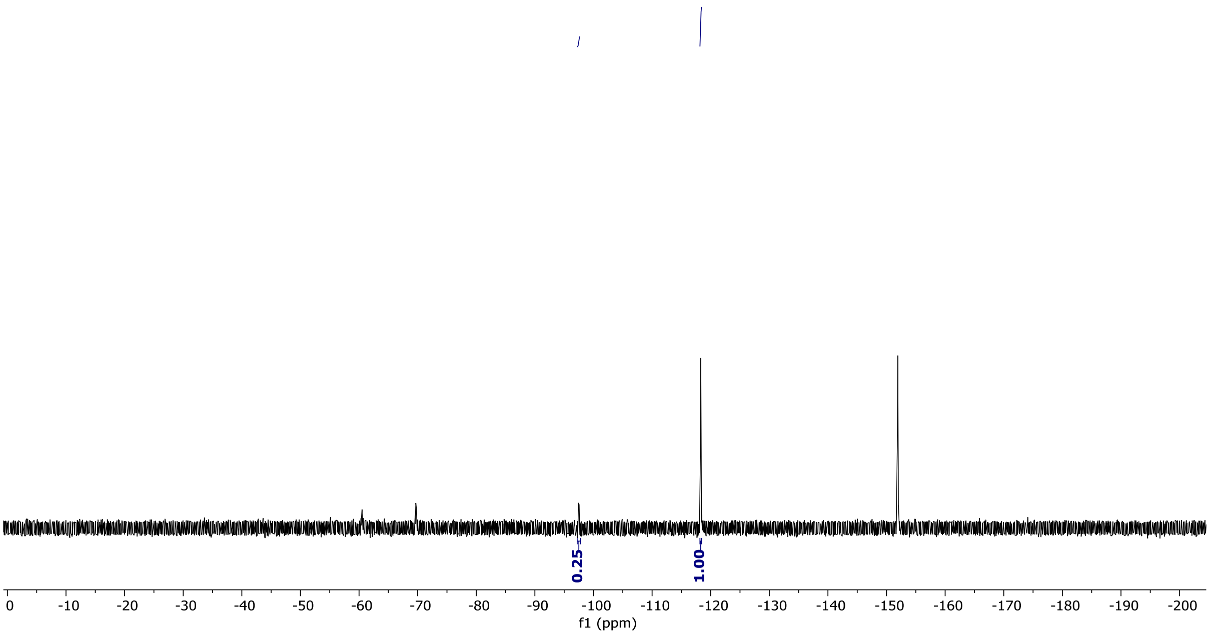

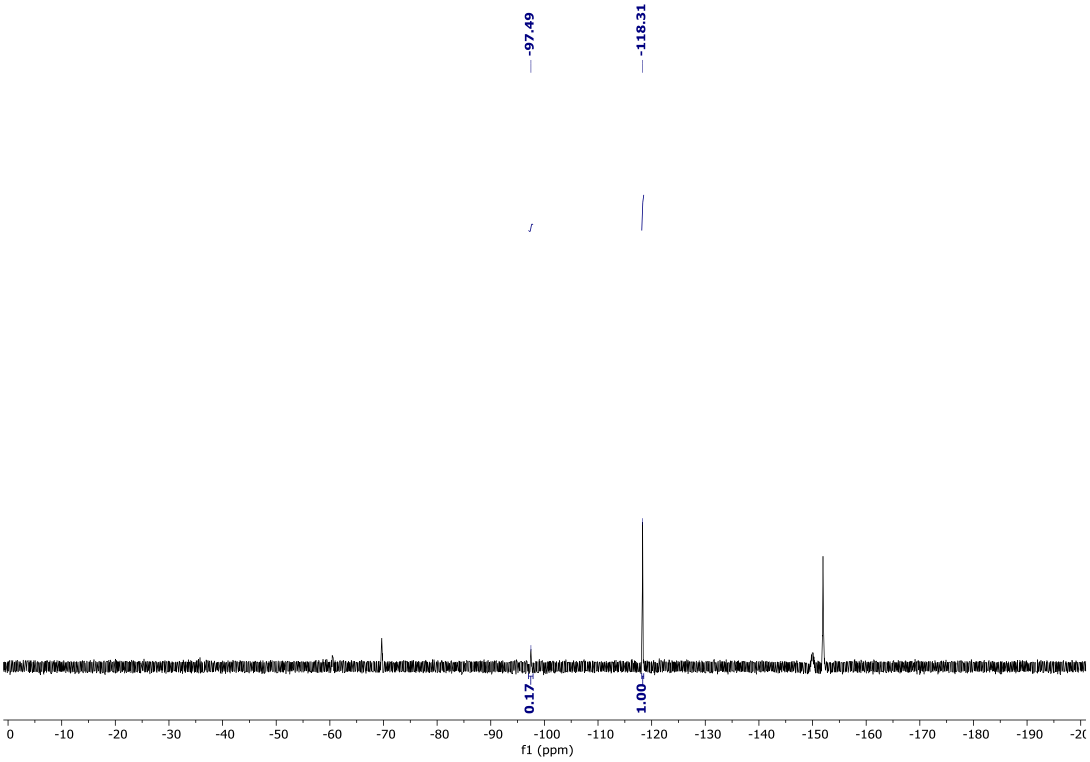

Competitive reaction between fluorovinyl iodane and vinyl iodane

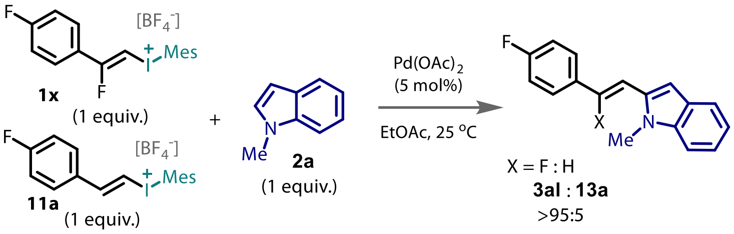

To a 10 mL dried Schlenk tube equipped with a stirrer bar was added $Pd(OAc)_2$ (0.005 mmol, 0.05 equiv), *N*-methylindole (0.10 mmol, 1.0 equiv., 13 mg), vinyl iodane **11a** (0.1 mmol, 1.0 equiv., 45 mg) and fluorovinyl iodane **1x** (0.1 mmol, 1.0 equiv., 47 mg). Dry ethylacetate 1 mL was added and reaction was allowed to stir at rt for 20 minutes. After 20 minutes, reaction was quenched with 4 mL of water and extracted with 5 mL of ethyl acetate. The organic layer was evaporated to 1 mL in *vacua* and ^{19}FNMR yield of crude product was recorded.

Competitive reaction between N-methylindole and 1-*H* indole

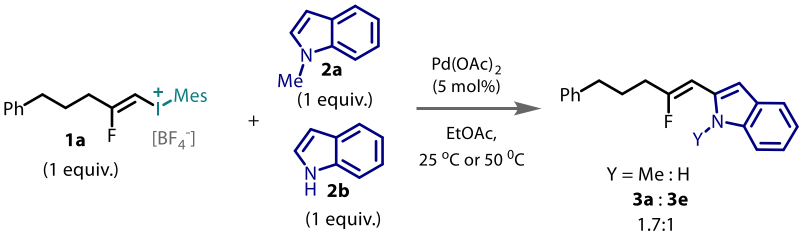

To a 10 mL dried Schlenk tube equipped with a stirrer bar was added $Pd(OAc)_2$ (0.005 mmol, 0.05 equiv), *N*-methylindole **2a** (0.10 mmol, 1.0 equiv., 13 mg), 1-*H*-lindole **2b** (0.10 mmol, 1.0 equiv., 12 mg) and fluorovinyl iodane **1a** (0.1 mmol, 1.0 equiv., 49 mg). Dry ethylacetate 1 mL was added and reaction was allowed to stir at rt for 20 minutes. After 20 minutes, reaction was quenched with 4 mL of water and extracted with 5 mL of ethyl acetate. The organic layer was evaporated to 1 mL in *vacua* and ^{19}FNMR yield of crude product was recorded.

Stability of flurovinylindole

(Z)-2-(2-fluoro-5-phenylpent-1-en-1-yl)-1-methyl-1H-indole subjected to below mentioned conditions to check the hydrolytic stability.

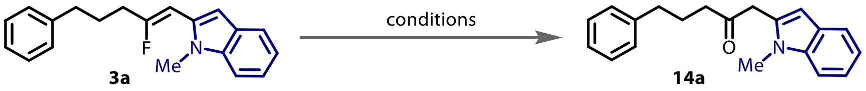

Entry	Conditions	Results
1	Stirred in acetic acid for 6 h at rt	20% Decomposition of compound **3a** was noticed
2	Stirred in acetic acid for 6 h at 70 ^{0}C	Decomposition of compound **3a** was noticed, no spot for ketone **14a**
3	Stirred in acetic acid and 10 eq water for 6h at rt	Decomposition of compound **3a** was noticed, no spot for ketone **14a**
4	Stirred in acetic acid and 10 eq water for 6h at 70 ^{0}C	Decomposition of compound **3a** was noticed, no spot for ketone **14a**
5	Stirred with K_2CO_3 (1 eq) in ethyl acetate for 6 h at rt	Decomposition of compound **3a** was noticed, with 2-3 faint spot on TLC

Plausible mechanism for fluorovinylation of indole

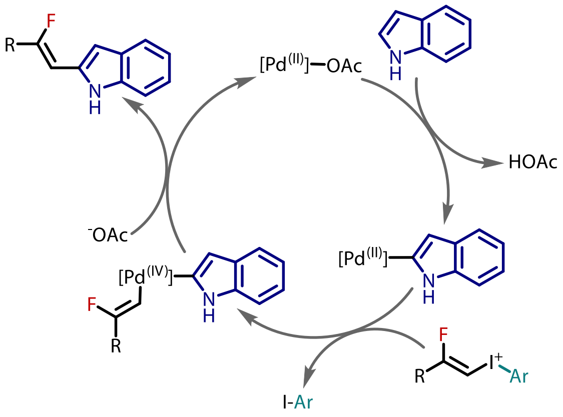

Plausible mechanism for diketone compound

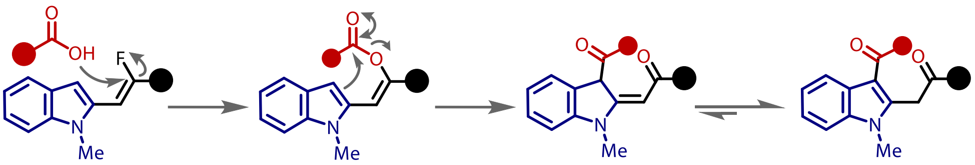

References

1. S. De *Synthetic Commun*., *2008*, **38**, 803–809.
2. A. T. Sedikides and A. J. J. Lennox *J. Am. Chem. Soc., 2024*, **146**, 15672−15680.
3. T. Kitamura, S. Mizuno, K. Muta and J. Oyamada, *J. Org. Chem., 2018*, **83**, 2773−2778.
4. P. Tian, C. Feng and T.-P Loh, *Nat. Commun., 2015*, ***6***, 7472.
5. X. Li, Y. Li, W. Shan, Z. Wang, R. Liu, Z. Zhang, X. Li and D. Shi, *Chem. Commun.,* 2023, **59**, 6893-6896.
6. T. Yamada, K. Arai, R. Kikuchi and S. Okamoto, *ACS Omega, 2021*, **6**, 19956−19963.
7. N. P. Grimster, C. Gauntlett, C. R. A. Godfrey and M. J. Gaunt, *Angew. Chem. Int. Ed., 2005***,** **44**, 3125−3129.

Spectral data

1-(4-methoxyphenyl)-2,5-dimethyl-1*H*-pyrrole **(4c)**

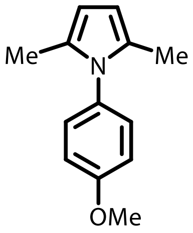

Compound **4c** was prepared according to general procedure 1 using hexane-2,5-dione (685 mg, 6 mmol), 4-methoxyaniline (616 mg, 5 mmol) and purified using silica gel chromatography (5% EtOAc/pentane) to yield a yellow solid (674 mg, 67%).

^{1}H NMR (400 MHz, $CDCl_3$) δ 7.16 (d, J = 8.9 Hz, 1H), 6.99 (d, J = 8.9 Hz, 1H), 5.91 (s, 1H), 3.88 (s, 2H), 2.05 (s, 3H).

^{13}C NMR (101 MHz, $CDCl_3$) δ 159.0, 131.9, 129.3, 129.1, 114.3, 105.4, 55.6, 13.1.

HRMS (ESI+) calc: $[M]^+$ ($C_{13}H_{15}ON$ 201.1148; measured: 201.1144 = 1.99 ppm

difference.

IR (neat) vmax/cm^{-1}: 2940, 1510, 1404, 1296, 1238, 1163, 842, 769, 559.

R_f = 0.44 (5% EtOAc/pentane).

1-cyclopropyl-2,5-dimethyl-1*H*-pyrrole **(4d)**

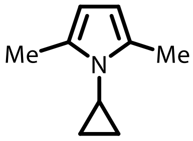

Compound **4d** was prepared according to general procedure 1 using hexane-2,5-dione (685 mg, 6 mmol), cyclopropanamine (285 mg, 5 mmol) and purified using silica gel chromatography (5% EtOAc/pentane) to yield a yellow solid (399 mg, 59%).

^{1}H NMR (400 MHz, $CDCl_3$) δ 5.63 (s, 2H), 2.80 (tt, J = 7.0, 4.1 Hz, 1H), 2.20 (s, 6H), 0.94 – 0.88 (m, 2H), 0.87 – 0.80 (m, 2H).

^{13}C NMR (101 MHz, $CDCl_3$) δ 130.3, 105.3, 25.7, 13.5, 7.4.

HRMS (ESI+) calc: $[M]^+$ ($C_9H_{13}N$) 135.1043; measured: 135.1039 = 2.96 ppm difference.

IR (neat) vmax/cm^{-1}: 2921, 1520, 1410, 1371, 1030, 747, 563.

R_f = 0.7 (5% EtOAc/pentane).

(*Z*)-(2-fluoro-5-phenylpent-1-en-1-yl)(4-nitrophenyl)iodonium BF_4 **(1g)**

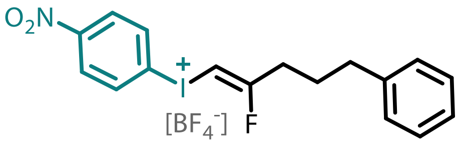

Compound **1g** was prepared according to general procedure 2a (condition b) using pent-4-yn-1-ylbenzene (288 mg, 2.0 mmol) and 1-iodo-4-nitrobenzene (548 mg, 2.20 mmol) and purified by trituration with pentane to yield a yellow sticky solid (419 mg, 42%).

^{1}H NMR (400 MHz, $CDCl_3$) δ 8.23 – 8.17 (m, 4H), 7.29 – 7.25 (m, 2H), 7.21 – 7.17 (m, 1H), 7.13 – 7.11 (m, 2H), 6.58 (d, J = 32.9 Hz, 1H), 2.62 – 2.55 (m, 4H), 1.89 (h, J = 7.0 Hz, 2H).

^{13}C NMR (101 MHz, $CDCl_3$) δ 174.6 (d, J = 281.3 Hz), 150.2, 140.4, 136.5, 128.8, 128.6, 126.7, 126.5, 116.9, 75.0 (d, J = 21.5 Hz), 34.6, 31.7 (d, J = 23.4 Hz), 27.0.

^{19}F NMR (377 MHz, $CDCl_3$) δ -61.35 (dt, J = 33.9, 17.3 Hz), -144.57 – -144.62 (BF_4).

HRMS (ESI+) calc: $[M]^+$ ($C_{17}H_{16}$FINO2) 412.0204; measured: 412.0191 = 3.16 ppm difference.

IR (neat) vmax/cm^{-1}: 3114, 1651, 1525, 1466, 1351, 1308, 1010, 995, 846, 733, 520.

(*Z*)-(2-fluoro-5-phenylpent-1-en-1-yl)(4-(trifluoromethyl)phenyl)iodonium BF_4 **(1h)**

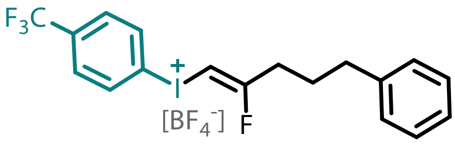

Compound **1h** was prepared according to general procedure 2a (condition b) using pent-4-yn-1-ylbenzene (288 mg, 2.0 mmol) and 1-iodo-4-(trifluoromethyl)benzene (598 mg, 2.20 mmol) and purified by trituration with pentane to yield a white sticky solid (449 mg, 43%).

^{1}H NMR (400 MHz, $CDCl_3$) δ 8.16 (d, J = 8.4 Hz, 2H), 7.66 (d, J = 8.6 Hz, 2H), 7.30 – 7.26 (m, 2H), 7.22 – 7.18 (m, 1H), 7.14 – 7.11 (m, 2H), 6.59 (d, J = 33.0 Hz, 1H), 2.64 – 2.54 (m, 4H), 1.90 (h, J = 7.0 Hz, 2H).

^{13}C NMR (101 MHz, $CDCl_3$) δ 174.2 (d, J = 280.6 Hz), 140.5, 135.9, 134.6 (q, J = 33.5 Hz), 128.9 (q, J = 3.7 Hz), 128.7, 128.6, 126.4, 123.1 (q, J = 273.2 Hz), 114.7, 75.0 (d, J = 21.7 Hz), 34.6, 31.7 (d, J = 23.5 Hz), 27.0.

^{19}F NMR (377 MHz, $CDCl_3$) δ -62.08 (dt, J = 33.7, 17.1 Hz), -63.35 (s), -144.62 – -144.68 (BF_4).

HRMS (ESI+) calc: $[M]^+$ ($C_{18}H_{16}F_4I$) 435.0227; measured: 435.0209 = 4.14 ppm

difference.

IR (neat) vmax/cm^{-1}: 3104, 1641, 1594, 1400, 1323, 1133, 1066, 834, 703.

(*Z*)-(2-cyclopropyl-2-fluorovinyl)(mesityl)iodonium BF_4 **(1k)**

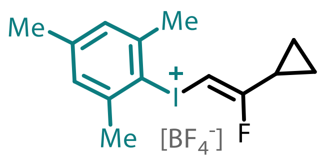

Compound **1k** was prepared according to general procedure 2a (condition b) using ethynylcyclopropane (132 mg, 2.0 mmol) and 2-iodo-1,3,5-trimethylbenzene (541 mg, 2.20 mmol) and purified by trituration with pentane to yield a white sticky solid (476.51 mg, 57%).

^{1}H NMR (400 MHz, $CDCl_3$) δ 7.06 (s, 2H), 6.28 (d, J = 34.0 Hz, 1H), 2.64 (s, 6H), 2.33 (s, 3H), 1.92 (dp, J = 24.7, 6.7 Hz, 1H), 0.99 (d, J = 6.6 Hz, 4H).

^{13}C NMR (101 MHz, $CDCl_3$) δ 173.9 (d, J = 270.3 Hz), 144.3, 142.5, 130.3, 119.5, 67.6 (d, J = 24.4 Hz), 27.0 (d, J = 1.3 Hz), 21.1, 12.9 (d, J = 26.2 Hz), 8.0 (d, J = 1.7 Hz).

^{19}F NMR (377 MHz, $CDCl_3$) δ -79.96 (dd, J = 34.1, 24.7 Hz), -148.07 – -148.13 (BF_4).

HRMS (ESI+) calc: $[M]^+$ ($C_{12}H_{17}FI$) 331.0353; measured: 331.0339 = 4.23 ppm

difference.

IR (neat) vmax/cm^{-1}: 3110, 1633, 1456, 1381, 1191, 1069, 1029, 921, 855, 734.

((*Z*)-2-fluoro-3-(((1*S*,2*R*,5*S*)-2-isopropyl-5-methylcyclohexyl)oxy)prop-1-en-1-yl)(mesityl)iodonium BF_4 **(1n)**

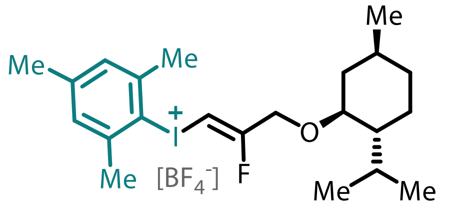

Compound **1n** was prepared according to general procedure 2a (condition b) using (1I,2S,4S)-1-isopropyl-4-methyl-2-(prop-2-yn-1-yloxy)cyclohexane (389 mg, 2.0 mmol) and 2-iodo-1,3,5-trimethylbenzene (541 mg, 2.20 mmol) and purified by trituration with pentane to yield a yellow sticky solid (557 mg, 51%).

^{1}H NMR (400 MHz, $CDCl_3$) δ 7.08 (s, 2H), 6.45 (d, J = 33.6 Hz, 1H), 4.37 – 4.19 (m, 2H), 3.10 (td, J = 10.6, 4.2 Hz, 1H), 2.66 (s, 6H), 2.34 (s, 3H), 2.06 – 1.93 (m, 2H), 1.65 – 1.57 (m, 2H), 1.33 – 1.16 (m, 4H), 0.91 – 0.83 (m, 7H), 0.65 (d, J = 6.9 Hz, 3H).

^{13}C NMR (101 MHz, $CDCl_3$) δ 170.0 (d, J = 278.9 Hz), 144.6, 142.7, 130.4, 119.0, 81.0, 74.2 (d, J = 19.4 Hz), 65.0 (d, J = 31.6 Hz), 48.1, 40.0, 34.4, 31.5, 27.2, 25.8, 23.4, 22.3, 21.2, 20.9, 16.2.

^{19}F NMR (377 MHz, $CDCl_3$) δ -76.76 (dt, J = 33.6, 9.2 Hz) -147.59 – -147.65 (BF_4).

HRMS (ESI+) calc: $[M+H]^+$ ($C_{22}H_{33}FIO$) 459.1555; measured: 459.1545 = 2.18 ppm

difference.

<u>(*E*)-(2-fluorododec-1-en-1-yl)(phenyl)iodonium BF_4 **(1ab)**</u>

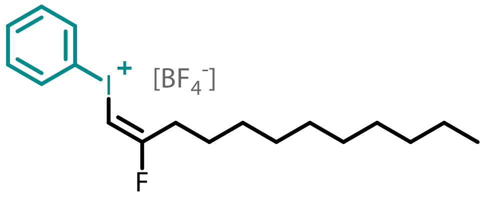

Compound **1ab** was prepared according to general procedure 2b using Dodecyne (166 mg, 1 mmol) and iodobenzene (306 mg, 1.50 mmol) and purified using silica gel chromatography (90% EtOAc/pentane) to yield a colourless oil (286 mg, 60%).

^{1}H NMR (400 MHz, $CDCl_3$) δ 7.97 (d, J = 8 Hz, 2H), 7.63 – 7.59 (m, 1H), 7.47 (dd, J = 8.4, 7.2 Hz, 2H), 6.72 (d, J = 14.4 Hz, 1H), 2.79 (dt, J = 22.2, 7.6 Hz, 2H), 1.50 (q, J = 7.1 Hz, 2H), 1.28 – 1.20 (m, 14H), 0.88 (t, J = 7 Hz 3H).

These data are consistent with those previously reported.[3]

<u>*N*-cyclopropyl-*N*-(2-fluoroallyl)-4-methylbenzenesulfonamide **(11a)**</u>

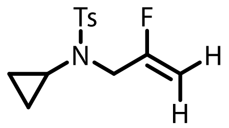

Compound **11a** was prepared according to general procedure 9 using (*Z*)-(3-((*N*-cyclopropyl-4-methylphenyl)sulfonamido)-2-fluoroprop-1-en-1-yl)(mesityl)iodonium BF_4 (301 mg, 0.5 mmol) and Sodium borohydride (38 mg, 1.0 mmol) and purified using silica gel chromatography (90% EtOAc/pentane) to yield a white solid (286 mg, 60%).

^{1}H NMR (500 MHz, $CDCl_3$) δ 7.78 – 7.77 (m, 2H), 7.34 (d, *J* = 8.0 Hz, 2H), 4.72 (dd, *J* = 16.3, 3.2 Hz, 1H), 4.56 (dd, *J* = 47.9, 3.1 Hz, 1H), 3.96 (d, *J* = 14.6 Hz, 2H), 2.46 (s, 3H), 2.14 (ttd, *J* = 6.9, 3.7, 1.0 Hz, 1H), 0.93 – 0.89 (m, 2H), 0.73 – 0.69 (m, 2H).

^{13}C NMR (126 MHz, $CDCl_3$) δ 161.6 (d, *J* = 261.1 Hz), 143.8, 135.5, 129.6, 128.0, 94.0 (d, *J* = 17.6 Hz), 50.7 (d, *J* = 31.8 Hz), 30.7, 21.7, 7.7.

^{19}F NMR (376 MHz, $CDCl_3$) δ -100.65 (dq, *J* = 47.9, 15.1 Hz).

HRMS (ESI+) calc: $[M+H]^+$ ($C_{13}H_{16}FNO_2S$) 270.0959; measured: 270.0953 = 2.22 ppm difference.

R_f = 0.5 (5% EtOAc/pentane).

(*Z*)-2-(2-fluoro-5-phenylpent-1-en-1-yl)-1-methyl-1*H*-indole **(3a)**

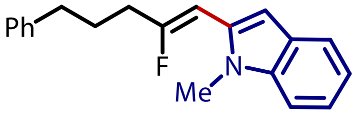

Compound **3a** was prepared according to general procedure 3a using 1-methyl-1*H*-indole (20 mg, 0.15 mmol) and (*Z*)-(2-fluoro-5-phenylpent-1-en-1-yl)(mesityl)iodonium BF_4 (50 mg, 0.1 mmol) and purified using silica gel chromatography (4% EtOAc/pentane) to yield a colourless oil (22 mg, 74%).

^{1}H NMR (400 MHz, $CDCl_3$) δ 7.58 (d, J = 7.8 Hz, 1H), 7.33 – 7.28 (m, 2H), 7.26 – 7.17 (m, 5H), 7.08 (ddd, J = 8.1, 6.9, 1.1 Hz, 1H), 6.84 (d, J = 2.5 Hz, 1H), 5.63 (d, J = 36.6 Hz, 1H), 3.69 (s, 3H), 2.73 (t, J = 7.6 Hz, 2H), 2.44 (dt, J = 17.6, 7.5 Hz, 2H), 1.99 (p, J = 7.6 Hz, 2H).

^{13}C NMR (101 MHz, $CDCl_3$) δ 162.1 (d, J = 269.7 Hz), 141.6, 136.9, 132.4, 128.6, 128.6, 128.3, 126.2, 121.6, 120.5, 119.7, 109.1, 102.6 (d, J = 11.8 Hz), 96.0 (d, J = 11.4 Hz), 35.1, 32.7 (d, J = 25.6 Hz), 29.8, 28.0.

^{19}F NMR (376 MHz, $CDCl_3$) δ -94.43 (dt, J = 36.1, 17.7 Hz).

HRMS (ESI+) calc: $[M+H]^+$ ($C_{20}H_{20}NF$) 294.1653; measured: 294.1642 = 3.74 ppm difference.

R_f = 0.5 (5% EtOAc/pentane).

(*Z*)-1-benzyl-2-(2-fluoro-5-phenylpent-1-en-1-yl)-1*H*-indole **(3d)**

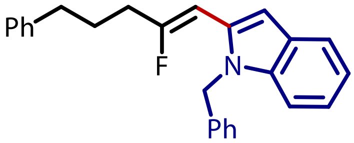

Compound **3d** was prepared according to general procedure 3a using 1-benzyl-1*H*-indole (62 mg, 0.3 mmol) and (*Z*)-(2-fluoro-5-phenylpent-1-en-1-yl)(mesityl)iodonium BF_4 (50 mg, 0.1 mmol) and purified using silica gel chromatography (5% EtOAc/pentane) to yield a colourless oil (16 mg, 44%).

^{1}H NMR (400 MHz, $CDCl_3$) δ 7.63 (dd, J = 7.2, 1.7 Hz, 1H), 7.29 – 7.24 (m, 5H), 7.23 – 7.18 (m, 2H), 7.15 – 7.08 (m, 4H), 7.01 – 6.99 (m, 2H), 6.95 (d, J = 2.5 Hz, 1H), 5.52 (d, J = 36.5 Hz, 1H), 5.35 (s, 2H), 2.63 (t, J = 7.6 Hz, 2H), 2.34 (dt, J = 17.8, 7.3 Hz, 2H), 1.90 (p, J = 7.5 Hz, 2H).

^{13}**C NMR** (101 MHz, $CDCl_3$) δ 162.3 (d, J = 270.1 Hz), 141.5, 137.9, 136.7, 132.2, 129.0, 128.60, 128.56, 128.5, 127.5, 126.10, 126.01, 122.0, 120.7, 120.1, 109.4, 103.3 (d, J = 12.3 Hz), 96.1 (d, J = 11.2 Hz), 46.6, 34.9, 32.5 (d, J = 25.7 Hz), 27.8.

19F NMR (376 MHz, $CDCl_3$) δ -93.91 (dt, J = 36.3, 18.0 Hz).

HRMS (ESI+) calc: $[M+H]^+$ ($C_{26}H_{24}FN$) 370.1966; measured: 370.1949 = 4.59 ppm difference.

IR (neat) vmax/cm^{-1}: 2990, 2907, 1692, 1453, 1067, 880, 560.

R_f = 0.4 (5% EtOAc/pentane).

(*Z*)-2-(2-fluoro-5-phenylpent-1-en-1-yl)-1*H*-indole **(3e)**

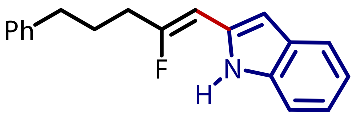

Compound **3e** was prepared according to general procedure 3a using 1*H*-indole (35 mg, 0.3 mmol) and (*Z*)-(2-fluoro-5-phenylpent-1-en-1-yl)(mesityl)iodonium BF_4 (50 mg, 0.1 mmol) and purified using silica gel chromatography (5% EtOAc/pentane) to yield a colourless oil (18mg, 63%).

^{1}**H NMR** (400 MHz, $CDCl_3$) δ: 8.64 (s, 1H), 7.47 (dq, J = 7.8, 0.9 Hz, 1H), 7.28 – 7.21 (m, 3H), 7.16 – 7.07 (m, 4H), 7.00 (ddd, J = 8.0, 7.0, 1.1 Hz, 1H), 6.32 (d, J = 2.1 Hz, 1H), 5.61 (d, J = 40.8 Hz, 1H), 2.65 (t, J = 7.6 Hz, 2H), 2.34 (dt, J = 18.6, 7.5 Hz, 2H), 1.89 (tt, J = 8.5, 6.8 Hz, 2H).

^{13}**C NMR** (101 MHz, $CDCl_3$) δ: 160.6 (d, J = 261.2 Hz), 141.6, 136.7, 131.9, 128.6, 128.1, 126.2, 122.4, 120.4, 120.1, 110.8, 102.6 (d, J = 3.4 Hz), 98.8 (d, J = 10.0 Hz), 35.1, 32.1 (d, J = 26.4 Hz), 27.95.

^{19}**F NMR** (377 MHz, $CDCl_3$) δ: -103.0 (dtd, J = 40.7, 18.6, 6.1 Hz).

HRMS (ESI+) calc: $[M+H]^+$ ($C_{19}H_{18}NF$) 280.1496; measured: 280.1488 = 2.86 ppm difference.

IR (neat) $\tilde{v}_{max}$/ cm^{-1}: 3021, 2959, 1376, 1070, 1027.

R_f = 0.6 (5% EtOAc/pentane).

(*Z*)-2-(2-fluoro-5-phenylpent-1-en-1-yl)-5-(p-tolyl)-1*H*-indole **(3f)**

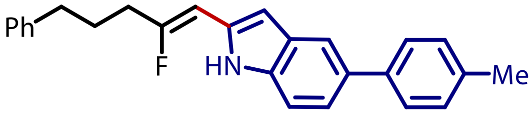

Compound **3f** was prepared according to general procedure 3a using 5-(p-tolyl)-1*H*-indole (62 mg, 0.3 mmol) and (*Z*)-(2-fluoro-5-phenylpent-1-en-1-yl)(mesityl)iodonium BF_4 (50 mg, 0.1 mmol) and purified using silica gel chromatography (5% EtOAc/pentane) to yield a colourless sticky solid (18 mg, 48%).

^{1}**H NMR** (400 MHz, $CDCl_3$) δ: 8.65 (s, 1H), 7.66 (dt, J = 1.7, 0.8 Hz, 1H), 7.47 – 7.45 (m, 2H), 7.35 – 7.28 (m, 2H), 7.26 – 7.21 (m, 2H), 7.18 – 7.12 (m, 5H), 6.35 (d, J = 2.0 Hz, 1H), 5.61 (d, J = 40.8 Hz, 1H), 2.65 (t, J = 7.6 Hz, 2H), 2.39 – 2.30 (m, 5H), 1.90 (p, J = 7.6 Hz, 2H).

^{13}**C NMR** (101 MHz, $CDCl_3$) δ: 160.7 (d, J = 261.4 Hz), 141.5, 139.8, 136.1, 133.5, 132.6, 129.5, 128.6, 128.6, 127.3, 126.2, 122.3, 118.6, 110.9, 102.8, 98.8 (d, J = 9.8 Hz), 35.1, 32.1 (d, J = 26.1 Hz), 27.9, 21.2.

^{19}**F NMR** (377 MHz, $CDCl_3$) δ: -102.7 (dtd, J = 40.8, 18.6, 6.1 Hz).

HRMS (ESI+) calc: $[M+H]^+$ ($C_{26}H_{24}NF$) 370.1966; measured: 370.1967 = 0.27 ppm difference.

IR (neat) vmax/cm^{-1}: 2924, 1692, 1454, 1321, 1066, 800, 700.

R_f = 0.4 (5% EtOAc/pentane).

(Z)-2-(2-fluoro-5-phenylpent-1-en-1-yl)-5-methyl-1*H*-indole **(3g)**

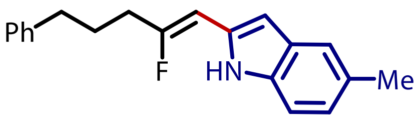

Compound **3g** was prepared according to general procedure 3a using 5-methyl-1*H*-indole (39 mg, 0.3 mmol) and (*Z*)-(2-fluoro-5-phenylpent-1-en-1-yl)(mesityl)iodonium BF_4 (50 mg, 0.1 mmol) and purified using silica gel chromatography (5% EtOAc/pentane) to yield a colourless sticky solid (20 mg, 68%).

^{1}**H NMR** (400 MHz, $CDCl_3$) δ: 8.54 (s, 1H), 7.26 – 7.20 (m, 3H), 7.17– 7.11 (m, 4H), 6.91 (dd, J = 8.3, 1.7 Hz, 1H), 6.23 (s, 1H), 5.58 (d, J = 40.9 Hz, 1H), 2.64 (t, J = 7.6 Hz, 2H), 2.37 – 2.28 (m, 5H), 1.88 (p, J = 7.6 Hz, 1H).

^{13}**C NMR** (101 MHz, $CDCl_3$) δ: 160.3 (d, J = 261.1 Hz), 141.6, 135.0, 132.0, 129.2, 128.6, 128.6, 128.3, 126.2, 124.1, 120.1, 110.4, 102.2, 98.9 (d, J = 10.0 Hz), 35.1, 32.1 (d, J = 26.4 Hz), 27.9, 21.6.

^{19}**F NMR** (377 MHz, $CDCl_3$) δ: -103.3 (dtd, J = 40.8, 18.5, 6.0 Hz).

HRMS (ESI+) calc: $[M+H]^+$ ($C_{20}H_{20}NF$) 294.1653; measured: 294.1640 = 4.42 ppm difference.

IR (neat) $\tilde{v}_{max}$/ cm^{-1}: 2923, 1449, 1312, 1029, 842, 791, 548.

R_f = 0.5 (5% EtOAc/pentane).

(*Z*)-2-(2-fluoro-5-phenylpent-1-en-1-yl)-5-methoxy-1*H*-indole **(3h)**

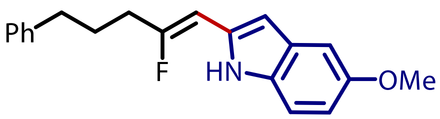

Compound **3h** was prepared according to general procedure 3a using 5-methoxy-1*H*-indole (44 mg, 0.3 mmol) and (*Z*)-(2-fluoro-5-phenylpent-1-en-1-yl)(mesityl)iodonium BF_4 (50 mg, 0.1 mmol) and purified using silica gel chromatography (5% EtOAc/pentane) to yield a colourless sticky solid (22 mg, 70%).

^{1}**H NMR** (400 MHz, $CDCl_3$) δ: 8.59 (s, 1H), 7.32 – 7.28 (m, 2H), 7.23 – 7.19 (m, 4H), 7.00 (d, J = 2.4 Hz, 1H), 6.83 (dd, J = 8.8, 2.4 Hz, 1H), 6.32 (d, J = 2.1 Hz, 1H), 5.65 (d, J = 40.8 Hz, 1H), 3.84 (s, 3H), 2.71 (t, J = 7.6 Hz, 2H), 2.40 (dt, J = 18.6, 7.5 Hz, 2H), 1.95 (p, J = 7.5 Hz, 2H).

^{13}C NMR (101 MHz, $CDCl_3$) δ: 160.4 (d, J = 261.1 Hz), 154.4, 141.6, 132.6, 131.9 (d, J = 3.3 Hz), 128.6, 128.5, 126.2, 112.7, 111.5, 102.4 (d, J = 3.3 Hz), 102.0, 98.8 (d, J = 10.0 Hz), 56.0, 35.1, 32.1 (d, J = 26.3 Hz), 28.0.

^{19}F NMR (377 MHz, $CDCl_3$) δ: -102.9 (dtd, J = 40.8, 18.7, 6.0 Hz).

HRMS (ESI+) calc: $[M+H]^+$ ($C_{20}H_{20}FNO$) 310.1602; measured: 310.1603 = 0.32 ppm difference.

IR (neat) vmax/cm^{-1}: 3473, 2924, 1482, 1449, 1198, 1141, 1032, 848, 790, 699, 551.

R_f = 0.3 (5% EtOAc/pentane).

(*Z*)-4-(benzyloxy)-2-(2-fluoro-5-phenylpent-1-en-1-yl)-1*H*-indole **(3i)**

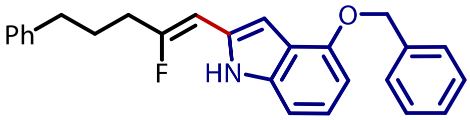

Compound **3i** was prepared according to general procedure 3a using 4-(benzyloxy)-1*H*-indole (134 mg, 0.6 mmol) and (*Z*)-(2-fluoro-5-phenylpent-1-en-1-yl)(mesityl)iodonium BF_4 (99 mg, 0.2 mmol) and purified using silica gel chromatography (5% EtOAc/pentane) to yield a colourless sticky solid (45 mg, 58%).

^{1}H NMR (400 MHz, $CDCl_3$) δ: 8.64 (s, 1H), 7.43 – 7.40 (m, 2H), 7.33 – 7.28 (m, 2H), 7.26 – 7.20 (m, 3H), 7.15 – 7.10 (m, 3H), 6.99 (t, J = 7.9 Hz, 1H), 6.90 (dt, J = 8.2, 0.9 Hz, 1H), 6.48 – 6.46 (m, 2H), 5.58 (d, J = 40.9 Hz, 1H), 5.13 (s, 2H), 2.63 (t, J = 7.6 Hz, 2H), 2.32 (dt, J = 18.7, 7.4 Hz, 2H), 1.87 (p, J = 7.6 Hz, 2H).

^{13}C NMR (101 MHz, $CDCl_3$) δ: 160.2 (d, J = 260.5 Hz), 152.4, 141.6, 138.1 (d, J = 3.3 Hz), 137.8, 130.6, 128.6, 128.6, 127.8, 127.5, 126.2, 123.2, 119.2, 104.5, 101.3, 100.2 (d, J = 3.2 Hz), 98.8 (d, J = 9.9 Hz), 70.1, 35.0, 32.0 (d, J = 26.4 Hz), 27.9.

^{19}F NMR (377 MHz, $CDCl_3$) δ: -103.7 (dtd, J = 41.0, 18.9, 6.2 Hz).

HRMS (ESI+) calc: $[M+H]^+$ ($C_{26}H_{24}FNO$) 386.1915; measured: 386.1908 = 1.81 ppm difference.

IR (neat) vmax/cm^{-1}: 3025, 2924, 1700, 1604, 1580, 1504, 1453, 1251, 1088, 907.

R_f = 0.3 (5% EtOAc/pentane).

(*Z*)-2-(2-fluoro-5-phenylpent-1-en-1-yl)-7-methoxy-1*H*-indole **(3j)**

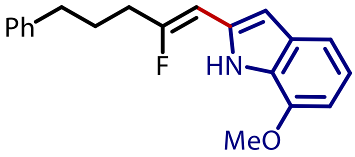

Compound **3j** was prepared according to general procedure 3a using 7-methoxy-1*H*-indole (88 mg, 0.6 mmol) and (*Z*)-(2-fluoro-5-phenylpent-1-en-1-yl)(mesityl)iodonium BF_4 (99 mg, 0.2 mmol) and purified using silica gel chromatography (5% EtOAc/pentane) to yield a colourless sticky solid (42 mg, 68%).

^{1}H NMR (400 MHz, $CDCl_3$) δ: 8.78 (s, 1H), 7.25 – 7.21 (m, 2H), 7.15 – 7.12 (m, 3H), 7.08 (dt, J = 8.0, 0.8 Hz, 1H), 6.91 (t, J = 7.8 Hz, 1H), 6.55 (dd, J = 7.7, 0.8 Hz, 1H), 6.30 (d, J = 2.3

Hz, 1H), 5.59 (d, J = 40.5 Hz, 1H), 3.89 (s, 3H), 2.65 (t, J = 7.6 Hz, 2H), 2.33 (dt, J = 18.6, 7.4 Hz, 2H), 1.92 – 1.85 (m, 2H).

^{13}C NMR (101 MHz, $CDCl_3$) δ: 160.4 (d, J = 261.6 Hz), 145.9, 141.6, 131.6, 129.3, 128.6, 128.6, 127.2, 126.2, 120.3, 113.2, 102.8 (d, J = 3.3 Hz), 102.3, 98.8 (d, J = 10.1 Hz), 55.5, 35.1, 32.1 (d, J = 26.2 Hz), 27.9.

^{19}F NMR (377 MHz, $CDCl_3$) δ -102.7 (dtd, J = 40.5, 18.5, 5.4 Hz).

HRMS (ESI+) calc: $[M+H]^+$ ($C_{20}H_{20}FNO$) 310.1602; measured: 310.1603 =0.32 ppm difference

IR (neat) vmax/cm^{-1}: 3489, 2928, 1693, 1580, 1407, 1327, 1254, 1095, 729.

R_f = 0.7 (10% EtOAc/pentane).

<u>(*Z*)-2-(2-fluoro-5-phenylpent-1-en-1-yl)-5-iodo-1*H*-indole **(3k)**</u>

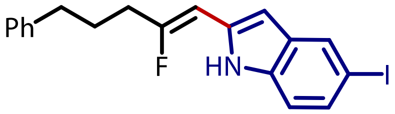

Compound **3k** was prepared according to general procedure 3a using 5-iodo-1*H*-indole (73 mg, 0.3 mmol) and (*Z*)-(2-fluoro-5-phenylpent-1-en-1-yl)(mesityl)iodonium BF_4 (50 mg, 0.1 mmol) and purified using silica gel chromatography (5% EtOAc/pentane) to yield a colourless sticky solid (12 mg, 29%).

^{1}H NMR (400 MHz, $CDCl_3$) δ 8.72 (s, 1H), 7.87 (d, J = 1.7 Hz, 1H), 7.40 (dd, J = 8.6, 1.7 Hz, 1H), 7.30 (t, J = 7.4 Hz, 2H), 7.22 – 7.18 (m, 3H), 7.11 (d, J = 8.4 Hz, 1H), 6.29 (d, J = 2.1 Hz, 1H), 5.65 (d, J = 40.6 Hz, 1H), 2.71 (t, J = 7.6 Hz, 2H), 2.44 – 2.36 (m, 2H), 1.96 (q, J = 7.6 Hz, 2H).

^{13}C NMR (151 MHz, $CDCl_3$) δ 161.3 (d, J = 262.4 Hz), 141.4, 135.7 (d, J = 3.0 Hz), 132.8, 130.7, 130.6, 129.1, 128.6, 128.6, 126.2, 112.7, 101.6 (d, J = 3.1 Hz), 98.4 (d, J = 9.8 Hz), 83.4, 35.1, 32.1 (d, J = 26.3 Hz), 27.9.

^{19}F NMR (376 MHz, $CDCl_3$) δ -101.42 (dtd, J = 40.4, 18.8, 6.4 Hz).

HRMS (ESI+) calc: $[M+H]^+$ ($C_{19}H_{17}FIN$) 406.0462; measured: 406.0476 = 3.45 ppm difference.

IR (neat) vmax/cm^{-1}: 2920, 1450, 1304, 1111, 883, 790, 698, 506.

R_f = 0.6 (5% EtOAc/pentane).

<u>(*Z*)-5-bromo-2-(2-fluoro-5-phenylpent-1-en-1-yl)-1*H*-indole **(3l)**</u>

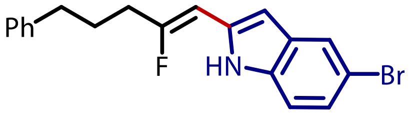

Compound **3l** was prepared according to general procedure 3a using 5-bromo-1*H*-indole (59 mg, 0.3 mmol) and (*Z*)-(2-fluoro-5-phenylpent-1-en-1-yl)(mesityl)iodonium BF_4 (50 mg, 0.1 mmol) and purified using silica gel chromatography (5% EtOAc/pentane) to yield a colourless sticky solid (15mg, 42%).

^{1}H NMR (400 MHz, $CDCl_3$) δ 8.73 (s, 1H), 7.66 (d, J = 1.8 Hz, 1H), 7.29 (q, J = 7.5 Hz, 2H), 7.29 – 7.18 (m, 5H), 6.31 (d, J = 2.1 Hz, 1H), 5.65 (d, J = 40.5 Hz, 1H), 2.71 (t, J = 7.6 Hz, 2H), 2.40 (dt, J = 18.5, 7.4 Hz, 2H), 1.95 (p, J = 7.5 Hz, 2H).

^{13}C NMR (126 MHz, $CDCl_3$) δ 161.3 (d, J = 262.5 Hz), 141.4, 135.2 (d, J = 3.1 Hz), 133.2(d, J = 1.1 Hz), 129.8, 128.6, 128.6, 126.2, 125.2, 122.8 (d, J = 1.2 Hz), 113.2, 112.1, 101.2 (d, J = 3.3 Hz), 98.5 (d, J = 9.8 Hz), 35.1, 32.0 (d, J = 26.1 Hz), 27.9.

^{19}F NMR (376 MHz, $CDCl_3$) δ -101.42 (dtd, J = 40.5, 18.8, 6.4 Hz).

HRMS (ESI+) calc: $[M+H]^+$ ($C_{19}H_{17}BrFN$) 358.0601; measured: 358.0611 = 2.79 ppm difference.

IR (neat) vmax/cm^{-1}: 2975, 2898, 1405, 1242, 1047, 861, 408

R$_f$ = 0.6 (5% EtOAc/pentane).

(*Z*)-5-fluoro-2-(2-fluoro-5-phenylpent-1-en-1-yl)-1*H*-indole **(3m)**

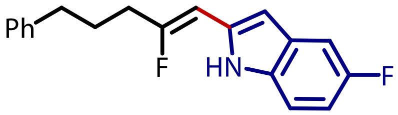

Compound **3m** was prepared according to general procedure 3a using 5-fluoro-1H-indole (41 mg, 0.3 mmol) and (Z)-(2-fluoro-5-phenylpent-1-en-1-yl)(mesityl)iodonium BF_4 (50 mg, 0.1 mmol) and purified using silica gel chromatography (5% EtOAc/pentane) to yield a colourless sticky solid (18mg, 61%).

^{1}H NMR (400 MHz, $CDCl_3$) δ 8.70 (s, 1H), 7.31 (dd, J = 8.5, 6.4 Hz, 2H), 7.25 – 7.17 (m, 5H), 6.91 (td, J = 9.1, 2.5 Hz, 1H), 6.35 (d, J = 2.1 Hz, 1H), 5.66 (d, J = 40.6 Hz, 1H), 2.72 (t, J = 7.6 Hz, 2H), 2.41 (dt, J = 18.6, 7.5 Hz, 2H), 1.96 (p, J = 7.5 Hz, 2H).

^{13}C NMR (126 MHz, $CDCl_3$) δ 161.9 (d, J = 262.2 Hz), 158.0 (d, J = 234.2 Hz), 141.5, 133.7, 133.1 (d, J = 3.1 Hz), 128.63, 128.60, 128.4 (d, J = 10.3 Hz), 126.2, 111.3 (d, J = 9.7 Hz), 110.7 (d, J = 26.4 Hz), 105.1 (dd, J = 23.6, 1.2 Hz), 102.6 (dd, J = 4.8, 3.2 Hz), 98.6 (d, J = 9.8 Hz), 35.1, 32.0 (d, J = 26.1 Hz), 27.9.

^{19}F NMR (376 MHz, $CDCl_3$) δ -101.78 (dtd, J = 40.5, 18.6, 6.1 Hz), -124.55 (td, J = 9.5, 4.3 Hz).

HRMS (ESI+) calc: $[M+H]^+$ ($C_{19}H_{17}NF_2$) 298.1402; measured: 298.1389 = 4.36 ppm difference.

IR (neat) vmax/cm^{-1}: 3473, 2924, 1484, 1448, 1178, 1129, 861, 698, 552.

R$_f$ = 0.5 (5% EtOAc/pentane).

(*Z*)-4-chloro-2-(2-fluoro-5-phenylpent-1-en-1-yl)-1*H*-indole **(3n)**

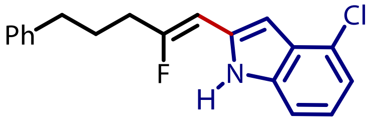

Compound **3n** was prepared according to general procedure 3a using 4-chloro-1*H*-indole (91 mg, 0.6 mmol) and (*Z*)-(2-fluoro-5-phenylpent-1-en-1-yl)(mesityl)iodonium BF_4 (99 mg, 0.2

mmol) and purified using silica gel chromatography (5% EtOAc/pentane) to yield a colourless sticky solid (23 mg, 37%).

^{1}H NMR (400 MHz, $CDCl_3$) δ 8.84 (s, 1H), 7.36 – 7.32 (m, 2H), 7.27 – 7.23 (m, 4H), 7.11 (d, J = 0.6 Hz, 1H), 7.10 (d, J = 1.2 Hz, 1H), 6.51 (d, J = 2.2 Hz, 1H), 5.73 (d, J = 40.6 Hz, 1H), 2.75 (t, J = 7.6 Hz, 2H), 2.45 (dt, J = 19.1, 7.5 Hz, 2H), 2.00 (p, J = 7.6 Hz, 2H).

^{13}C NMR (126 MHz, $CDCl_3$) δ 161.3 (d, J = 262.4 Hz), 141.4, 137.2 (d, J = 3.1 Hz), 132.5, 128.63, 128.61, 127.0, 126.2, 125.70 (d, J = 1.4 Hz), 122.9, 119.8, 109.3, 101.0 (d, J = 3.2 Hz), 98.5 (d, J = 9.8 Hz), 35.0, 32.0 (d, J = 26.1z Hz), 27.9.

^{19}FNMR (376 MHz, $CDCl_3$) δ -101.57 (dtd, J = 40.6, 18.8, 6.5 Hz).

HRMS (ESI+) calc: $[M+H]^+$ ($C_{19}H_{17}ClFN$) 314.1106; measured: 314.1106 = 0 ppm difference.

IR (neat) vmax/cm^{-1}: 3356, 2947, 1097, 1615, 1435, 1257, 1123, 769, 700.

R_f = 0.3 (5% EtOAc/pentane).

(*Z*)-4-chloro-2-(2-fluoro-5-phenylpent-1-en-1-yl)-1-methyl-1*H*-indole **(3o)**

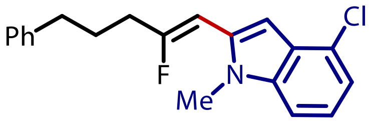

Compound **3o** was prepared according to general procedure 3a using 4-chloro-1-methyl-1*H*-indole (50 mg, 0.3 mmol) and (*Z*)-(2-fluoro-5-phenylpent-1-en-1-yl)(mesityl)iodonium BF_4 (99 mg, 0.2 mmol) and purified using silica gel chromatography (5% EtOAc/pentane) to yield a colourless sticky solid (32 mg, 48%).

^{1}H NMR (400 MHz, $CDCl_3$) δ: 7.31 – 7.27 (m, 2H), 7.23 – 7.19 (m, 3H), 7.17 – 7.13 (m, 1H), 7.07 (dd, J = 4.5, 0.9 Hz, 2H), 6.91 (d, J = 2.4 Hz, 1H), 5.61 (d, J = 36.1 Hz, 1H), 3.68 (s, 3H), 2.75 – 2.71 (m, 2H), 2.44 (dt, J = 17.7, 7.5 Hz, 2H), 1.99 (p, J = 7.5 Hz, 2H).

^{13}C NMR (101 MHz, $CDCl_3$) δ: 162.9 (d, J = 271.3 Hz), 141.5, 137.7, 133.2, 128.6, 128.6, 127.1, 126.2, 125.8, 122.1, 119.5, 107.8, 101.1 (d, J = 12.2 Hz), 95.7 (d, J = 11.5 Hz), 35.1, 32.7 (d, J = 25.5 Hz), 30.2, 28.0.

^{19}F NMR (377 MHz, $CDCl_3$) δ: -92.9 (dtd, J = 36.0, 17.8, 2.5 Hz).

HRMS (ESI+) calc: $[M+H]^+$ ($C_{20}H_{19}ClFN$) 328.1263; measured: 328.1252= 3.35 ppm difference.

IR (neat) vmax/cm^{-1}: 2932, 1685, 1600, 1453, 1283, 1124, 763, 564.

R_f = 0.4 (5% EtOAc/pentane).

Methyl (*Z*)-2-(2-fluoro-5-phenylpent-1-en-1-yl)-1*H*-indole-5-carboxylate **(3p)**

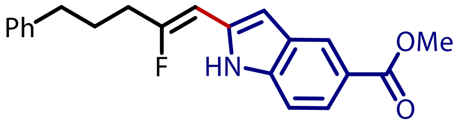

Compound **3p** was prepared according to general procedure 3a using methyl 1*H*-indole-5-carboxylate (105 mg, 0.6 mmol) and (*Z*)-(2-fluoro-5-phenylpent-1-en-1-yl)(mesityl)iodonium

BF_4 (99 mg, 0.2 mmol) and purified using silica gel chromatography (5% EtOAc/pentane) to yield a colourless sticky solid (28 mg, 42%).

^{1}H NMR (500 MHz, $CDCl_3$) δ 8.92 (s, 1H), 8.35 (dt, J = 1.5, 0.8 Hz, 1H), 7.91 (dd, J = 8.6, 1.6 Hz, 1H), 7.38 – 7.32 (m, 3H), 7.26 – 7.22 (m, 3H), 6.50 (s, 1H), 5.72 (d, J = 40.5 Hz, 1H), 3.95 (s, 3H), 2.75 (t, J = 7.6 Hz, 2H), 2.48 – 2.40 (m, 2H), 1.99 (p, J = 7.6 Hz, 2H).

^{13}C NMR (126 MHz, $CDCl_3$) δ 168.3, 161.3 (d, J = 262.4 Hz), 141.4, 139.2 (d, J = 2.8 Hz), 133.4 (d, J = 1.1 Hz), 128.62, 128.59, 127.7, 126.2, 123.4 (d, J = 1.2 Hz), 122.2, 110.4, 103.5 (d, J = 3.3 Hz), 98.5 (d, J = 9.7 Hz), 52.0, 35.0, 32.0 (d, J = 26.0 Hz), 27.9.

19F NMR (376 MHz, $CDCl_3$) δ -101.11 – -101.93 (m).

HRMS (ESI+) calc: $[M+H]^+$ ($C_{21}H_{20}FNO_2$) 338.1551; measured: 338.1560 = 3.25 ppm difference

IR (neat) vmax/cm^{-1}: 3356, 2947, 1097, 1615, 1435, 1257, 1123, 769, 700.

R_f = 0.3 (5% EtOAc/pentane).

Ethyl (*Z*)-2-(2-fluoro-5-phenylpent-1-en-1-yl)-1*H*-indole-4-carboxylate **(3q)**

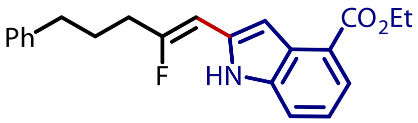

Compound **3q** was prepared according to general procedure 3a using ethyl 1*H*-indole-4-carboxylate (114 mg, 0.6 mmol) and (*Z*)-(2-fluoro-5-phenylpent-1-en-1-yl)(mesityl)iodonium BF_4 (99 mg, 0.2 mmol) and purified using silica gel chromatography (5% EtOAc/pentane) to yield a colourless sticky solid (21 mg, 30%).

^{1}H NMR (500 MHz, $CDCl_3$) δ 8.91 (d, J = 6.0 Hz, 1H), 7.91 (dd, J = 7.6, 0.9 Hz, 1H), 7.55 (dt, J = 8.1, 1.0 Hz, 1H), 7.34 (dd, J = 8.6, 6.6 Hz, 2H), 7.26 – 7.22 (m, 4H), 7.07 (d, J = 2.1 Hz, 1H), 5.78 (d, J = 40.7 Hz, 1H), 4.47 (q, J = 7.2 Hz, 2H), 2.76 (t, J = 7.6 Hz, 2H), 2.46 (dt, J = 18.8, 7.4 Hz, 2H), 2.00 (p, J = 7.6 Hz, 2H), 1.48 (d, J = 14.3 Hz, 3H).

^{13}C NMR (126 MHz, $CDCl_3$) δ 167.7, 161.6 (d, J = 262.8 Hz), 141.4, 137.5 (d, J = 3.0 Hz), 134.0, 128.67, 128.61, 127.7, 126.2, 123.5, 121.5, 121.4, 115.3, 104.0 (d, J = 2.9 Hz), 98.8 (d, J = 9.6 Hz), 60.7, 35.0, 32.1 (d, J = 26.1 Hz), 27.9, 14.6.

19FNMR (376 MHz, $CDCl_3$) δ -101.00 (dtd, J = 42.5, 18.9, 6.4 Hz).

HRMS (ESI+) calc: $[M+H]^+$ ($C_{22}H_{22}FNO_2$) 352.1707; measured: 352.1691 = 4.54 ppm difference.

IR (neat) vmax/cm^{-1}: 3378, 2927, 1691, 1497, 1341, 1270, 1187, 752.

R_f = 0.6 (10% EtOAc/pentane).

(*Z*)-2-(2-fluoro-5-phenylpent-1-en-1-yl)-1*H*-indol-5-ol **(3r)**

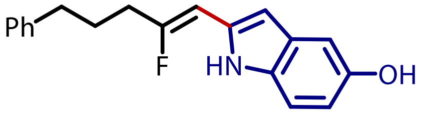

Compound **3r** was prepared according to general procedure 3a using 1*H*-indol-5-ol (40 mg, 0.3 mmol) and (*Z*)-(2-fluoro-5-phenylpent-1-en-1-yl)(mesityl)iodonium BF_4 (50 mg, 0.1 mmol)

and purified using silica gel chromatography (5% EtOAc/pentane) to yield a colourless sticky solid (13 mg, 44%).

^{1}H NMR (400 MHz, $CDCl_3$) δ 8.63 (s, 1H), 7.36 – 7.30 (m, 2H), 7.27 – 7.21 (m, 4H), 6.98 (d, J = 2.4 Hz, 1H), 6.77 (dd, J = 8.6, 2.4 Hz, 1H), 6.31 (d, J = 2.1 Hz, 1H), 5.67 (d, J = 40.8 Hz, 1H), 4.55 (s, 1H), 2.75 (t, J = 7.6 Hz, 2H), 2.43 (dt, J = 18.8, 7.4 Hz, 2H), 1.99 (p, J = 7.6 Hz, 2H).

^{13}C NMR (101 MHz, $CDCl_3$) δ 160.5 (d, J = 261.6 Hz), 149.6, 141.4, 132.9, 131.9 (d, J = 3.4 Hz), 128.7, 128.5, 126.1, 112.1, 111.3, 104.6, 101.9 (d, J = 3.2 Hz), 98.7 (d, J = 10.0 Hz), 34.9, 31.9 (d, J = 26.2 Hz), 27.8.

^{19}F NMR (377 MHz, $CDCl_3$) δ -102.60 (dtd, J = 40.8, 18.7, 6.0 Hz).

HRMS (ESI+) calc: $[M+H]^+$ ($C_{20}H_{20}FNO$) 332.1421; measured: 332.1414 = 2.11 ppm difference.

IR (neat) vmax/cm^{-1}: 3401, 2930, 1692, 1451, 1182, 699, 468, 408.

R$_f$ = 0.3 (5% EtOAc/pentane).

<u>(*Z*)-2-(2-fluoro-5-phenylpent-1-en-1-yl)-3-methyl-1*H*-indole **(3s)**</u>

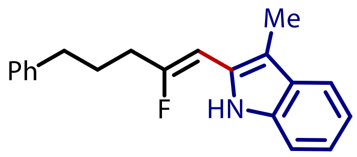

Compound **3s** was prepared according to general procedure 3a using 3-methyl-1*H*-indole (79 mg, 0.6 mmol) and (*Z*)-(2-fluoro-5-phenylpent-1-en-1-yl)(mesityl)iodonium BF_4 (99 mg, 0.2 mmol) and purified using silica gel chromatography (5% EtOAc/pentane) to yield a colourless sticky solid (35 mg, 60%).

^{1}H NMR (400 MHz, $CDCl_3$) δ 8.64 (d, J = 6.5 Hz, 1H), 7.52 (d, J = 7.8 Hz, 1H), 7.34 – 7.32 (m, 3H), 7.25 – 7.20 (m, 3H), 7.21 – 7.16 (m, 1H), 7.10 (t, J = 7.5 Hz, 1H), 5.73 (d, J = 41.3 Hz, 1H), 2.74 (t, J = 7.6 Hz, 2H), 2.45 (dt, J = 19.0, 7.5 Hz, 2H), 2.30 (s, 3H), 1.99 (p, J = 7.6 Hz, 2H).

^{13}C NMR (101 MHz, $CDCl_3$) δ 159.9 (d, J = 260.2 Hz), 141.6, 136.0 (d, J = 3.3 Hz), 128.6, 128.6, 128.4, 128.2, 126.2, 122.5, 119.3, 118.7, 110.5, 109.8 (d, J = 2.9 Hz), 96.7 (d, J = 9.9 Hz), 35.1, 32.3 (d, J = 26.6 Hz), 28.1, 8.8.

^{19}F NMR (376 MHz, $CDCl_3$) δ -104.97 (dtd, J = 41.1, 19.0, 6.5 Hz).

HRMS (ESI+) calc: $[M+H]^+$ ($C_{20}H_{20}NF$) 294.1653; measured: 294.1654 = 0.34 ppm difference.

IR (neat) vmax/cm^{-1}: 2948, 1394, 1317, 1062, 699.

R$_f$ = 0.5 (5% EtOAc/pentane).

<u>(*Z*)-2-(2-(2-fluoro-5-phenylpent-1-en-1-yl)-1*H*-indol-3-yl)ethan-1-ol **(3t)**</u>

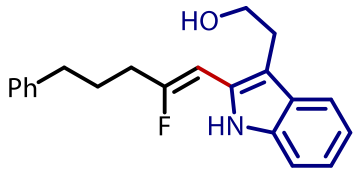

Compound **3t** was prepared according to general procedure 3b using 2-(1*H*-indol-3-yl)ethan-1-ol (97 mg, 0.6 mmol) and (*Z*)-(2-fluoro-5-phenylpent-1-en-1-yl)(mesityl)iodonium BF_4 (99 mg, 0.2 mmol) and purified using silica gel chromatography (5% EtOAc/pentane) to yield a colourless sticky solid (25 mg, 39%).

^{1}H NMR (500 MHz, $CDCl_3$) δ 8.76 (d, J = 6.7 Hz, 1H), 7.55 (dd, J = 7.9, 1.0 Hz, 1H), 7.35 – 7.30 (m, 3H), 7.23 – 7.18 (m, 4H), 7.10 (ddd, J = 8.0, 7.0, 1.0 Hz, 1H), 5.78 (d, J = 41.0 Hz, 1H), 3.85 (t, J = 6.4 Hz, 2H), 3.02 (t, J = 6.4 Hz, 2H), 2.73 (t, J = 7.6 Hz, 2H), 2.44 (dt, J = 19.1, 7.5 Hz, 2H), 1.98 (ddd, J = 15.1, 8.3, 6.9 Hz, 2H).

^{13}C NMR (126 MHz, $CDCl_3$) δ 160.8 (d, J = 261.5 Hz), 141.5, 136.2 (d, J = 3.1 Hz), 129.6 (d, J = 1.9 Hz), 128.64, 128.62, 127.7, 126.2, 122.8, 119.7, 118.7 (d, J = 1.3 Hz), 110.8, 110.2 (d, J = 2.9 Hz), 96.5 (d, J = 9.4 Hz), 63.1, 35.1, 32.3 (d, J = 26.5 Hz), 28.0, 27.9.

^{19}F NMR (377 MHz, $CDCl_3$) δ -103.51 (dtd, J = 41.0, 19.1, 6.9 Hz).

HRMS (ESI+) calc: $[M+H]^+$ ($C_{21}H_{22}FNO$) 324.1758; measured: 324.1773 = 4.63 ppm difference.

IR (neat) vmax/cm^{-1}: 3441, 2926, 1690, 1495, 1455, 1313, 1043, 742, 700.

R$_f$ = 0.4 (5% EtOAc/pentane).

<u>(*Z*)-2-(2-(2-fluoro-2-phenylvinyl)-1*H*-indol-3-yl)ethan-1-ol **(3u)**</u>

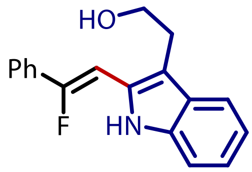

Compound **3u** was prepared according to general procedure 3b using 2-(1*H*-indol-3-yl)ethan-1-ol (97 mg, 0.6 mmol) and (*Z*)-(2-fluoro-2-phenylvinyl)(mesityl)iodonium BF_4 (91 mg, 0.2 mmol) and purified using silica gel chromatography (5% EtOAc/pentane) to yield a colourless sticky solid (42 mg, 75%).

^{1}H NMR (500 MHz, $CDCl_3$) δ 8.97 (d, J = 6.4 Hz, 1H), 7.73 – 7.65 (m, 2H), 7.64 – 7.61 (m, 1H), 7.48 – 7.45 (m, 2H), 7.42 – 7.40 (m, 2H), 7.31 – 7.24 (m, 1H), 7.16 (ddd, J = 8.0, 7.0, 1.0 Hz, 1H), 6.63 (d, J = 41.1 Hz, 1H), 3.93 (t, J = 6.4 Hz, 2H), 3.17 (t, J = 6.4 Hz, 2H).

^{13}C NMR (126 MHz, $CDCl_3$) δ 156.8 (d, J = 251.7 Hz), 136.6 (d, J = 3.5 Hz), 132.0 (d, J = 27.7 Hz), 129.9 (d, J = 2.3 Hz), 129.3, 128.9 (d, J = 2.3 Hz), 127.8, 124.1 (d, J = 7.8 Hz), 123.4, 119.9, 118.8 (d, J = 1.5 Hz), 112.7 (d, J = 3.3 Hz), 111.0, 95.5 (d, J = 11.3 Hz), 63.1, 28.1.

^{19}F NMR (377 MHz, $CDCl_3$) δ -118.06 (dd, J = 41.1, 7.0 Hz).

HRMS (ESI+) calc: $[M+H]^+$ ($C_{18}H_{16}FNO$) 282.1289; measured: 282.1279 = 3.54 ppm

IR (neat) vmax/cm^{-1}: 3463, 2933, 1494, 1447, 1317, 1036, 1006, 761, 739, 466.

R$_f$ = 0.4 (5% EtOAc/pentane).

<u>(*Z*)-2-(2-fluorododec-1-en-1-yl)-1-methyl-1*H*-indole **(3v)**</u>

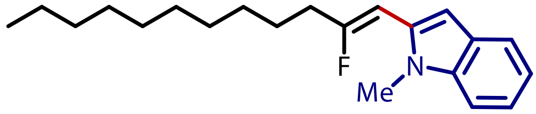

Compound **3v** was prepared according to general procedure 3a using 1-methyl-1*H*-indole (39 mg, 0.3 mmol) and (*Z*)-(2-fluorododec-1-en-1-yl)(mesityl)iodonium BF_4 (104 mg, 0.2 mmol) and purified using silica gel chromatography (5% EtOAc/pentane) to yield a colourless sticky solid (42 mg, 66%).

^{1}H NMR (400 MHz, $CDCl_3$) δ: 7.49 (dt, J = 7.9, 1.0 Hz, 1H), 7.19 – 7.17 (m, 1H), 7.11 – 7.07 (m, 1H), 6.99 (ddd, J = 7.9, 6.9, 1.1 Hz, 1H), 6.75 (d, J = 2.5 Hz, 1H), 5.55 (d, J = 36.6 Hz, 1H), 3.61 (s, 3H), 2.32 (dt, J = 17.8, 7.5 Hz, 2H), 1.60 – 1.51 (m, 2H), 1.29 – 1.16 (m, 15H), 0.83 – 0.79 (m, 3H).

^{13}C NMR (101 MHz, $CDCl_3$) δ: 162.7 (d, J = 269.6 Hz), 136.9, 132.6 (d, J = 2.2 Hz), 128.4, 121.6, 120.5, 119.7, 109.1, 102.5 (d, J = 11.9 Hz), 95.6 (d, J = 11.6 Hz), 33.3 (d, J = 25.5 Hz), 32.0, 29.8, 29.7, 29.7, 29.5, 29.5, 29.1, 26.5, 22.8, 14.3.

^{19}F NMR (377 MHz, $CDCl_3$) δ -94.0 (dtd, J = 36.3, 17.8, 2.5 Hz).

HRMS (ESI+) calc: $[M+H]^+$ ($C_{21}H_{30}FN$) 316.2435; measured: 316.2428 = 2.21 ppm

difference.

IR (neat) vmax/cm^{-1}: 2925, 2852, 1723, 1466, 1265, 1102, 733.

R_f = 0.7 (5% EtOAc/pentane).

(*Z*)-2-(2-fluoro-3,3-dimethylbut-1-en-1-yl)-1-methyl-1*H*-indole **(3w)**

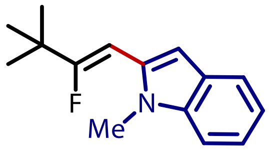

Compound **3w** was prepared according to general procedure 3b using 1-methyl-1*H*-indole (39 mg, 0.3 mmol) and (*Z*)-(2-fluoro-3,3-dimethylbut-1-en-1-yl)(mesityl)iodonium BF_4 (87 mg, 0.2 mmol) and purified using silica gel chromatography (5% EtOAc/pentane) to yield a colourless sticky solid (35 mg, 75%).

^{1}H NMR (400 MHz, $CDCl_3$) δ: 7.49 (dt, J = 7.9, 1.0 Hz, 1H), 7.19 – 7.15 (m, 1H), 7.09 (ddd, J = 8.2, 6.9, 1.2 Hz, 1H), 6.99 (ddd, J = 8.0, 7.0, 1.1 Hz, 1H), 6.75 (d, J = 2.7 Hz, 1H), 5.60 (d, J = 37.7 Hz, 1H), 3.61 (s, 3H), 1.19 (s, 9H).

^{13}C NMR (101 MHz, $CDCl_3$) δ: 169.5 (d, J = 272.8 Hz), 137.0, 132.8, 128.4, 121.5, 120.5, 119.7, 109.1, 102.4 (d, J = 12.6 Hz), 92.2 (d, J = 12.4 Hz), 35.8 (d, J = 23.0 Hz), 29.8, 27.5 (d, J = 2.6 Hz).

^{19}F NMR (377 MHz, $CDCl_3$) δ: -102.4 (dd, J = 37.7, 2.6 Hz).

HRMS (ESI+) calc: $[M+H]^+$ ($C_{15}H_{18}FNO$) 232.1496; measured: 232.1485 = 4.73 ppm difference.

IR (neat) vmax/cm^{-1}: 2964, 1682, 1462, 1301, 1072, 870, 776, 506.

R_f = 0.7 (5% EtOAc/pentane).

(*Z*)-2-(2-cyclopropyl-2-fluorovinyl)-1-methyl-1*H*-indole **(3x)**

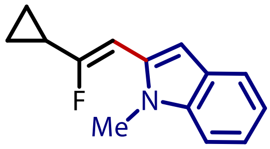

Compound **3x** was prepared according to general procedure 3b using 1-methyl-1*H*-indole (39 mg, 0.3 mmol) and (*Z*)-(2-cyclopropyl-2-fluorovinyl)(mesityl)iodonium BF_4 (84 mg, 0.2 mmol) and purified using silica gel chromatography (5% EtOAc/pentane) to yield a colourless sticky solid (30 mg, 69%).

^{1}H NMR (400 MHz, $CDCl_3$) δ: 7.48 (dt, J = 7.8, 1.0 Hz, 1H), 7.19 – 7.17 (m, 1H), 7.09 (ddd, J = 8.2, 7.0, 1.2 Hz, 1H), 6.99 (ddd, J = 8.0, 7.0, 1.1 Hz, 1H), 6.69 (dt, J = 2.7, 0.7 Hz, 1H), 5.64 (d, J = 36.2 Hz, 1H), 3.62 (s, 3H), 1.62 (dtt, J = 21.6, 8.3, 5.2 Hz, 1H), 0.87 – 0.83 (m, 2H), 0.81 – 0.75 (m, 2H).

^{13}C NMR (101 MHz, $CDCl_3$) δ: 162.1 (d, J = 264.8 Hz), 136.9, 132.8 (d, J = 3.0 Hz), 128.4, 121.5, 120.4, 119.7, 109.1, 102.3 (d, J = 11.8 Hz), 94.0 (d, J = 13.3 Hz), 29.8, 13.4 (d, J = 28.3 Hz), 5.6 (d, J = 2.1 Hz).

^{19}F NMR (377 MHz, $CDCl_3$) δ: -108.2 (ddd, J = 36.1, 21.1, 2.7 Hz).

HRMS (ESI+) calc: $[M+H]^+$ ($C_{14}H_{14}FN$) 216.1183; measured: 216.1183 = 0 ppm difference.

IR (neat) vmax/cm^{-1}: 2978, 1394, 1250, 1062, 895.

R_f = 0.7 (5% EtOAc/pentane).

(*Z*)-2-(2-cyclohexyl-2-fluorovinyl)-1-methyl-1*H*-indole **(3y)**

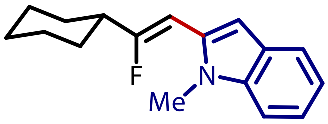

Compound **3y** was prepared according to general procedure 3a using 1-methyl-1*H*-indole (39 mg, 0.3 mmol) and (*Z*)-(2-cyclohexyl-2-fluorovinyl)(mesityl)iodonium BF_4 (92 mg, 0.2 mmol) and purified using silica gel chromatography (5% EtOAc/pentane) to yield a colourless sticky solid (40 mg, 78%).

^{1}H NMR (400 MHz, $CDCl_3$) δ: 7.49 (dt, J = 7.8, 1.0 Hz, 1H), 7.19 – 7.17 (m, 1H), 7.09 (ddd, J = 8.2, 6.9, 1.2 Hz, 1H), 6.99 (ddd, J = 8.0, 6.9, 1.1 Hz, 1H), 6.74 (d, J = 2.5 Hz, 1H), 5.52 (d, J = 37.6 Hz, 1H), 3.61 (s, 3H), 2.25 (ddd, J = 14.8, 7.2, 3.8 Hz, 1H), 1.95 – 1.90 (m, 2H), 1.79 – 1.74 (m, 2H), 1.68 – 1.63 (m, 1H), 1.36 – 1.14 (m, 5H).

^{13}C NMR (101 MHz, $CDCl_3$) δ: 166.6 (d, J = 271.0 Hz), 137.0, 132.7 (d, J = 2.0 Hz), 128.4, 121.5, 120.5, 119.7, 109.1, 102.4 (d, J = 12.1 Hz), 93.5 (d, J = 11.6 Hz), 41.7 (d, J = 23.7 Hz), 30.2, 30.2, 29.8, 26.1, 26.0.

^{19}F NMR (377 MHz, $CDCl_3$) δ: -98.5 (ddd, J = 37.6, 14.8, 2.5 Hz).

HRMS (ESI+) calc: $[M+H]^+$ ($C_{17}H_{20}NF$) 258.1653; measured: 258.1652 = 0.39 ppm

IR (neat) vmax/cm^{-1}: 2929, 2854, 1721, 1466, 1317, 741.

R_f = 0.7 (5% EtOAc/pentane).

(*Z*)-2-(4-(benzyloxy)-2-fluorobut-1-en-1-yl)-1-methyl-1*H*-indole **(3z)**

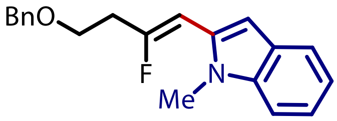

Compound **3z** was prepared according to general procedure 3a using 1-methyl-1*H*-indole (39 mg, 0.3 mmol) and (*Z*)-(4-(benzyloxy)-2-fluorobut-1-en-1-yl)(mesityl)iodonium BF_4 (102 mg, 0.2 mmol) and purified using silica gel chromatography (5% EtOAc/pentane) to yield a colourless sticky solid (14 mg, 22%).

^{1}H NMR (400 MHz, $CDCl_3$) δ 7.58 (d, J = 7.9 Hz, 1H), 7.35 – 7.31 (m, 4H), 7.31 – 7.27 (m, 2H), 7.20 – 7.16 (m, 1H), 7.07 (t, J = 7.5 Hz, 1H), 6.84 (d, J = 2.4 Hz, 1H), 5.75 (d, J = 36.7 Hz, 1H), 4.57 (s, 2H), 3.75 (t, J = 6.4 Hz, 2H), 3.68 (s, 3H), 2.72 (dt, J = 17.6, 6.4 Hz, 2H).

^{13}C NMR (101 MHz, $CDCl_3$) δ 138.2, 136.9, 132.3, 128.6, 128.3, 127.9, 127.9, 121.7, 120.6, 119.7, 102.8 (d, J = 12.0 Hz), 97.3 (d, J = 10.8 Hz), 73.3, 66.5, 34.1 (d, J = 25.8 Hz), 29.8.

^{19}F NMR (376 MHz, $CDCl_3$) δ -95.45 (dt, J = 35.7, 17.2 Hz).

HRMS (ESI+) calc: $[M+H]^+$ ($C_{20}H_{20}FNO$) 310.1602; measured: 310.1611 = 2.9 ppm

IR (neat) vmax/cm^{-1}: 2937, 2857, 1733, 1546, 1426, 1217, 760.

R_f = 0.5 (5% EtOAc/pentane).

2-((*Z*)-2-fluoro-3-(((1*R*,2*S*,5*R*)-2-isopropyl-5-methylcyclohexyl)oxy)prop-1-en-1-yl)-1-methyl-1*H*-indole **(3aa)**

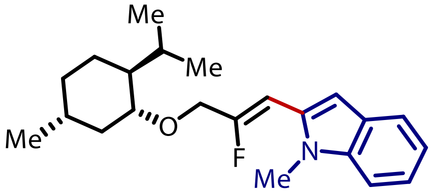

Compound **3aa** was prepared according to general procedure 3a using 1-methyl-1*H*-indole (39 mg, 0.15 mmol) and ((*Z*)-2-fluoro-3-(((1R,2S,5R)-2-isopropyl-5-methylcyclohexyl)oxy)prop-1-en-1-yl)(mesityl)iodonium BF_4 (109 mg, 0.2 mmol) and purified using silica gel chromatography (5% EtOAc/pentane) to yield a colourless sticky solid (24 mg, 35%).

^{1}H NMR (400 MHz, $CDCl_3$) δ 7.60 (dt, J = 7.9, 1.0 Hz, 1H), 7.29 (dd, J = 8.3, 1.0 Hz, 1H), 7.20 (ddd, J = 8.2, 7.0, 1.2 Hz, 1H), 7.09 (ddd, J = 7.9, 7.0, 1.1 Hz, 1H), 6.92 (d, J = 2.2 Hz, 1H), 5.97 (d, J = 35.9 Hz, 1H), 4.35 – 4.29 (m, 1H), 4.16 – 4.10 (m, 1H), 3.73 (s, 3H), 3.25 (td, J = 10.6, 4.2 Hz, 1H), 2.29 (pd, J = 7.0, 2.7 Hz, 1H), 2.15 (dtd, J = 12.1, 3.6, 1.8 Hz, 1H), 1.71 – 1.63 (m, 2H), 1.44 – 1.21 (m, 3H), 1.07 – 0.99 (m, 1H), 0.98 – 0.93 (m, 6H), 0.91 – 0.85 (m, 1H), 0.82 (d, J = 7.0 Hz, 3H).

^{13}C NMR (101 MHz, $CDCl_3$) δ 158.4 (d, J = 270.6 Hz), 137.1, 131.7 (d, J = 2.8 Hz), 128.2, 122.0, 120.8, 119.8, 109.2, 103.5 (d, J = 12.0 Hz), 97.7 (d, J = 8.7 Hz), 79.9, 66.9 (d, J = 31.4 Hz), 48.4, 40.5, 34.6, 31.7, 29.8, 25.9, 23.5, 22.5, 21.1, 16.4.

^{19}F NMR (377 MHz, $CDCl_3$) δ -104.49 (dtd, J = 35.9, 13.5, 2.3 Hz).

HRMS (ESI+) calc: $[M+H]^+$ ($C_{22}H_{30}FNO$) 344.2384; measured: 344.2373 = 3.20 ppm difference.

IR (neat) vmax/cm^{-1}: 2952, 2920, 1462, 1317, 1235, 1084, 784, 748.

R_f = 0.5 (5% EtOAc/pentane).

(*Z*)-2-(2-fluoro-3-(1-methyl-1*H*-indol-2-yl)allyl)isoindoline-1,3-dione **(3ab)**

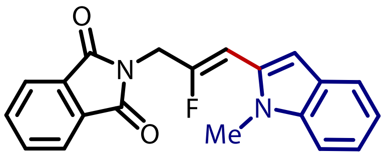

Compound **3ab** was prepared according to general procedure 3a using 1-methyl-1*H*-indole (39 mg, 0.15 mmol)and (*Z*)-(3-(1,3-dioxoisoindolin-2-yl)-2-fluoroprop-1-en-1-yl)(mesityl)iodonium BF_4 (108 mg, 0.2 mmol) and purified using silica gel chromatography (5% EtOAc/pentane) to yield a colourless sticky solid (21 mg, 31%).

^{1}H NMR (400 MHz, $CDCl_3$) δ 7.90 (dd, J = 5.5, 3.1 Hz, 2H), 7.75 (dd, J = 5.5, 3.0 Hz, 2H), 7.57 (dt, J = 8.0, 1.0 Hz, 1H), 7.19 (ddd, J = 8.3, 6.9, 1.2 Hz, 1H), 7.07 (ddd, J = 8.0, 6.9, 1.1 Hz, 1H), 6.87 (d, J = 2.3 Hz, 1H), 6.08 (d, J = 34.9 Hz, 1H), 4.61 (d, J = 16.4 Hz, 2H), 3.73 (s, 3H).

^{13}C NMR (101 MHz, $CDCl_3$) δ 167.7, 154.4 (d, J = 271.3 Hz), 137.2, 134.4, 132.1, 131.0, 128.1, 123.8, 122.3, 120.9, 119.9, 109.3, 104.1 (d, J = 12.2 Hz), 99.8 (d, J = 9.2 Hz), 39.3 (d, J = 30.4 Hz), 29.9.

^{19}F NMR (377 MHz, $CDCl_3$) δ -103.93 (dtd, J = 35.0, 16.4, 2.4 Hz).

HRMS (ESI+) calc: $[M+H]^+$ ($C_{20}H_{15}FN_2O_2$) 335.1190; measured: 335.1191 = 0.30 ppm difference.

IR (neat) vmax/cm^{-1}: 2922, 1773, 1715, 1466, 1388, 1103, 949, 731, 530.

R_f = 0.6 (15% EtOAc/pentane).

(Z)-*N*-(2-fluoro-3-(1-methyl-1*H*-indol-2-yl)allyl)-*N*-(4-fluorobenzyl)-4-nitrobenzenesulfonamide **(3ac)**

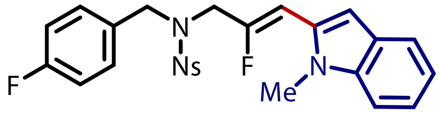

Compound **3ac** was prepared according to general procedure 3a using 1-methyl-1*H*-indole (20 mg, 0.15 mmol) and (*Z*)-(2-fluoro-3-((*N*-(4-fluorobenzyl)-4-nitrophenyl)sulfonamido)prop-1-en-1-yl)(mesityl)iodonium BF_4 (70 mg, 0.1 mmol) and purified using silica gel chromatography (10% EtOAc/pentane) to yield a colourless sticky solid (37 mg, 74%).

^{1}H NMR (400 MHz, $CDCl_3$) δ: 8.34 – 8.30 (m, 2H), 8.05 – 8.03 (m, 2H), 7.56 (dt, J = 7.9, 1.0 Hz, 1H), 7.35 – 7.32 (m, 2H), 7.28 – 7.20 (m, 2H), 7.11 – 7.04 (m, 3H), 6.69 (d, J = 2.4 Hz, 1H), 5.70 (d, J = 35.4 Hz, 1H), 4.47 (s, 2H), 4.12 (d, J = 18.7 Hz, 2H), 3.63 (s, 3H).

^{13}C NMR (101 MHz, $CDCl_3$) δ 162.8 (d, J = 247.8 Hz), 153.85 (d, J = 271.4 Hz), 150.0, 145.7, 137.1, 130.3 (d, J = 8.0 Hz), 129.9 (d, J = 3.2 Hz), 128.5, 127.8, 124.3, 122.7, 120.9, 120.2, 116.0, 115.8, 109.2, 104.4 (d, J = 12.4 Hz), 101.1 (d, J = 9.2 Hz), 50.3, 47.7zz (d, J = 26.6 Hz), 29.7.

^{19}F NMR (377 MHz, $CDCl_3$) δ: -103.9 (dtd, J = 35.1, 18.6, 2.5 Hz), -113.1 (tt, J = 8.5, 5.2 Hz).

HRMS (ESI+) calc: $[M+H]^+$ ($C_{25}H_{21}F_2N_3O_4S$) 498.1294; measured: 498.1273 = 4.22 ppm

difference.

IR (neat) vmax/cm^{-1}: 2958, 1603, 1529, 1348, 1163, 854, 743, 605, 571.

R_f = 0.5 (25% EtOAc/pentane).

(*Z*)-*N*-(2-fluoro-3-(1-methyl-1*H*-indol-2-yl)allyl)-4-methylbenzenesulfonamide **(3ad)**

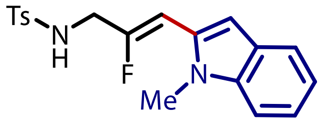

Compound **3ad** was prepared according to general procedure 3a using 1-methyl-1*H*-indole (20 mg, 0.15 mmol) and (*Z*)-(2-fluoro-3-((4-methylphenyl)sulfonamido)prop-1-en-1-yl)(mesityl)iodonium BF_4 (56 mg, 0.1 mmol) and purified using silica gel chromatography (10% EtOAc/pentane) to yield a colourless sticky solid (11 mg, 30%).

^{1}H NMR (400 MHz, $CDCl_3$) δ: 7.69 (d, J = 8.4 Hz, 2H), 7.48 (dt, J = 7.9, 1.0 Hz, 1H), 7.20 – 7.17 (m, 3H), 7.13 (ddd, J = 8.2, 6.8, 1.2 Hz, 1H), 7.01 (ddd, J = 8.0, 6.9, 1.2 Hz, 1H), 6.64 (d, J = 2.4 Hz, 1H), 5.73 (d, J = 35.6 Hz, 1H), 3.86 (ddd, J = 13.8, 6.5, 0.7 Hz, 2H), 3.55 (s, 3H), 2.25 (s, 3H).

^{13}C NMR (101 MHz, $CDCl_3$) δ: 155.0 (d, J = 268.5 Hz), 143.9, 137.0, 129.8, 129.7, 127.9, 127.1, 126.5, 122.2, 120.7, 119.8, 109.1, 103.8 (d, J = 12.1 Hz), 98.6 (d, J = 9.1 Hz), 44.5 (d, J = 31.4 Hz), 29.6, 21.4.

^{19}F NMR (377 MHz, $CDCl_3$) δ: -105.0 (dtd, J = 35.7, 13.7, 2.5 Hz).

HRMS (ESI+) calc: $[M+H]^+$ ($C_{19}H_{19}FN_2O_2S$) 359.1240; measured: 359.1224 = 4.46 ppm difference.

IR (neat) vmax/cm^{-1}: 3664, 2978, 1394, 1251, 1062.

R_f = 0.5 (25% EtOAc/pentane).

(*Z*)-N-(2-fluoro-3-(1-methyl-1*H*-indol-2-yl)allyl)-*N*-(4-fluorophenyl)-4-methylbenzenesulfonamide **(3ae)**

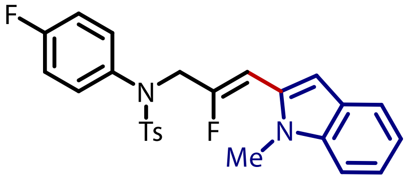

Compound **3ae** was prepared according to general procedure 3a using 1-methyl-1*H*-indole (39 mg, 0.3 mmol) and (*Z*)-(2-fluoro-3-((*N*-(4-fluorophenyl)-4-methylphenyl)sulfonamido)prop-1-en-1-yl)(mesityl)iodonium BF_4 (131 mg, 0.2 mmol) and purified using silica gel chromatography (10% EtOAc/pentane) to yield a colourless sticky solid (54 mg, 60%).

^{1}H NMR (400 MHz, $CDCl_3$) δ: 7.57 – 7.53 (m, 3H), 7.28 – 7.23 (m, 3H), 7.19 (ddd, J = 8.3, 6.9, 1.2 Hz, 1H), 7.12 – 7.07 (m, 3H), 7.02 – 6.96 (m, 2H), 6.79 (d, J = 2.3 Hz, 1H), 5.78 (d, J = 34.9 Hz, 1H), 4.48 (d, J = 15.4 Hz, 2H), 3.60 (s, 3H), 2.43 (s, 3H).

^{13}C NMR (101 MHz, $CDCl_3$) δ: 158.8 (d, J = 472.8 Hz), 144.1, 137.2, 135.8, 134.9 (d, J = 3.3 Hz), 131.4 (d, J = 8.8 Hz), 130.9, 129.7, 128.0, 127.9, 122.4, 120.8, 120.0, 116.5, 116.2, 109.3, 103.9 (d, J = 11.8 Hz), 100.3 (d, J = 9.5 Hz), 53.1 (d, J = 29.6 Hz), 29.8, 21.7.

^{19}F NMR (377 MHz, $CDCl_3$) δ: -102.8 (dtd, J = 35.3, 15.3, 2.4 Hz), -112.1 (tt, J = 8.0, 4.8 Hz).

HRMS (ESI+) calc: $[M+H]^+$ ($C_{25}H_{22}NF_2N2O_2S$) 453.1443; measured: 453.1459 = 3.53 ppm

IR (neat) vmax/cm^{-1}: 2977, 1394, 1257, 1063, 879, 551.

R_f = 0.5 (15% EtOAc/pentane).

(*Z*)-N-(4-bromophenyl)-*N*-(2-fluoro-3-(1-methyl-1*H*-indol-2-yl)allyl)-4-methylbenzenesulfonamide **(3af)**

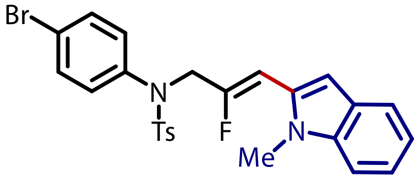

Compound **3af** was prepared according to general procedure 3a using 1-methyl-1*H*-indole (20 mg, 0.15 mmol) and (*Z*)-(3-((*N*-(4-bromophenyl)-4-methylphenyl)sulfonamido)-2-fluoroprop-1-en-1-yl)(mesityl)iodonium BF_4 (65 mg, 0.1 mmol) and purified using silica gel chromatography (10% EtOAc/pentane) to yield a colourless sticky solid (33 mg, 60%).

^{1}H NMR (400 MHz, $CDCl_3$) δ: 7.57 – 7.52 (m, 3H), 7.45 – 7.41 (m, 2H), 7.28 – 7.23 (m, 3H), 7.19 (ddd, J = 8.2, 6.8, 1.2 Hz, 1H), 7.07 (ddd, J = 8.0, 6.8, 1.1 Hz, 1H), 7.03 – 6.99 (m, 2H), 6.78 (d, J = 2.3 Hz, 1H), 5.79 (d, J = 35.0 Hz, 1H), 4.48 (d, J = 15.1 Hz, 2H), 3.60 (s, 3H), 2.43 (s, 3H).

^{13}C NMR (101 MHz, $CDCl_3$) δ: 154.8 (d, J = 270.7 Hz), 144.0, 138.0, 137.0, 135.5, 132.5, 130.8, 130.7, 129.7, 127.9, 127.7, 122.5, 122.3, 120.7, 119.9, 109.2, 103.8 (d, J = 11.9 Hz), 100.2 (d, J = 9.4 Hz), 52.6 (d, J = 29.9 Hz), 29.7, 21.6.

^{19}F NMR (377 MHz, $CDCl_3$) δ -102.9 (dtd, J = 35.1, 15.0, 2.4 Hz).

HRMS (ESI+) calc: $[M+H]^+$ ($C_{25}H_{22}BrFN_2O_2S$) 513.0642; measured: 513.0618 = 4.68 ppm difference.

IR (neat) vmax/cm^{-1}: 2914, 1483, 1348, 1318, 1157, 1071, 877, 750, 583, 545.

R_f = 0.5 (15% EtOAc/pentane).

(*Z*)-*N*-cyclopropyl-*N*-(2-fluoro-3-(1-methyl-1*H*-indol-2-yl)allyl)-4-methylbenzenesulfonamide **(3ag)**

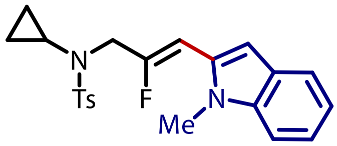

Compound **3ag** was prepared according to general procedure 3a using 1-methyl-1*H*-indole (20 mg, 0.15 mmol) and (*Z*)-(3-((N-cyclopropyl-4-methylphenyl)sulfonamido)-2-fluoroprop-1-en-1-yl)(mesityl)iodonium BF_4 (60 mg, 0.1 mmol) and purified using silica gel chromatography (10% EtOAc/pentane) to yield a colourless sticky solid (28 mg, 70%).

^{1}H NMR (400 MHz, $CDCl_3$) δ: 7.78 – 7.75 (m, 2H), 7.58 (dt, J = 7.9, 1.0 Hz, 1H), 7.30 – 7.27 (m, 2H), 7.21 (ddd, J = 8.2, 6.9, 1.2 Hz, 1H), 7.09 (ddd, J = 7.9, 6.9, 1.1 Hz, 1H), 6.79 (d, J = 2.3 Hz, 1H), 5.96 (d, J = 35.2 Hz, 1H), 4.16 (d, J = 16.4 Hz, 2H), 3.71 (s, 3H), 2.41 (s, 3H), 2.22 (dtd, J = 6.8, 3.2, 1.2 Hz, 1H), 0.93 (td, J = 4.3, 3.1 Hz, 2H), 0.77 – 0.70 (m, 2H).

^{13}C NMR (101 MHz, $CDCl_3$) δ: 156.5 (d, J = 272.7 Hz), 143.9, 135.5, 129.7, 128.1, 128.0, 122.3, 120.8, 120.0, 109.3, 103.9 (d, J = 12.1 Hz), 99.8 (d, J = 9.6 Hz), 52.0 (d, J = 28.9 Hz), 30.9, 29.9, 21.7, 8.0.

^{19}F NMR (377 MHz, $CDCl_3$) δ: -100.7 (dt, J = 34.0, 16.4 Hz).

HRMS (ESI+) calc: $[M+H]^+$ ($C_{22}H_{23}FN_2O_2S$) 399.1537; measured: 399.1519 = 4.51 ppm difference.

IR (neat) vmax/cm^{-1}: 2943, 1598, 1466, 1323, 1157, 815, 666, 547.

R_f = 0.5 (15% EtOAc/pentane).

<u>(*Z*)-3-fluoro-4-(1-methyl-1*H*-indol-2-yl)but-3-en-2-one **(3ah)**</u>

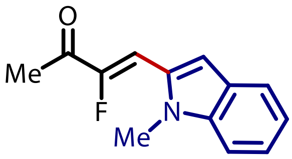

Compound **3ah** was prepared according to general procedure 3a using 1-methyl-1*H*-indole (39 mg, 0.3 mmol) and (*Z*)-(2-fluoro-3-oxobut-1-en-1-yl)(mesityl)iodonium BF_4 (67 mg, 0.2 mmol) and purified using silica gel chromatography (5% EtOAc/pentane) to yield a colourless sticky solid (13 mg, 30%).

^{1}H NMR (400 MHz, $CDCl_3$) δ 7.66 (dt, J = 8.0, 1.1 Hz, 1H), 7.34 – 7.27 (m, 2H), 7.22 (d, J = 2.0 Hz, 1H), 7.13 (ddd, J = 8.0, 6.4, 1.5 Hz, 1H), 7.05 (d, J = 33.7 Hz, 1H), 3.81 (s, 3H), 2.45 (d, J = 3.8 Hz, 3H).

^{13}C NMR (101 MHz, $CDCl_3$) δ 192.0 (d, J = 34.0 Hz), 154.5 (d, J = 274.1 Hz), 138.5, 130.1 (d, J = 5.0 Hz), 128.1, 124.2, 121.9, 120.6, 109.7, 108.7 (d, J = 15.1 Hz), 104.1 (d, J = 8.2 Hz), 29.9, 25.8 (d, J = 1.5 Hz).

^{19}F NMR (377 MHz, $CDCl_3$) δ -119.71 (dqd, J = 33.7, 3.8, 2.0 Hz).

HRMS (ESI+) calc: $[M+H]^+$ ($C_{13}H_{12}FNO$) 218.0976; measured: 218.0972 = 1.83 ppm difference.

IR (neat) vmax/cm^{-1}: 2976, 1677, 1624, 1461, 1394, 1324, 1255, 1065, 869.

R_f = 0.5 (5% EtOAc/pentane).

<u>Ethyl (*z*)-2-fluoro-3-(1-methyl-1*H*-indol-2-yl)acrylate **(3ai)**</u>

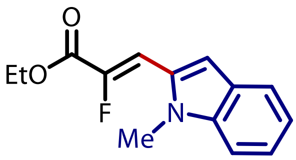

Compound **3ai** was prepared according to general procedure 3a using 1-methyl-1*H*-indole (39 mg, 0.3 mmol) and (*Z*)-(3-ethoxy-2-fluoro-3-oxoprop-1-en-1-yl)(mesityl)iodonium BF_4 (73 mg, 0.2 mmol) and purified using silica gel chromatography (5% EtOAc/pentane) to yield a colourless sticky solid (16 mg, 32%).

^{1}H NMR (400 MHz, $CDCl_3$) δ: 7.66 – 7.63 (m, 1H), 7.33 – 7.25 (m, 2H), 7.19 (d, J = 2.0 Hz, 1H), 7.15 – 7.07 (m, 2H), 4.38 (q, J = 7.1 Hz, 2H), 3.79 (s, 3H), 1.40 (t, J = 7.1 Hz, 3H).

^{13}C NMR (101 MHz, $CDCl_3$) δ: 161.4 (d, J = 32.6 Hz), 147.2 (d, J = 270.4 Hz), 138.2, 129.9 (d, J = 5.1 Hz), 128.0, 123.9, 121.7, 120.5, 109.7, 108.2 (d, J = 14.7 Hz), 106.7 (d, J = 7.5 Hz), 62.1, 29.9, 14.4.

^{19}F NMR (376 MHz, $CDCl_3$) δ: -121.3 (d, J = 32.4 Hz).

HRMS (ESI+) calc: $[M+H]^+$ ($C_{14}H_{14}FNO_2$) 248.1081; measured: 248.1072 = 3.63 ppm

difference.

IR (neat) vmax/cm^{-1}: 2991, 1716, 1661, 1461, 1367, 1255, 1095, 790, 752, 646.

R_f = 0.5 (5% EtOAc/pentane).

<u>(*Z*)-2-(2-fluoro-2-phenylvinyl)-1-methyl-1*H*-indole **(3aj)**</u>

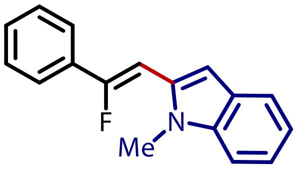

Compound **3aj** was prepared according to general procedure 4 using 1-methyl-1*H*-indole (39 mg, 0.3 mmol) and (*Z*)-(2-fluoro-2-phenylvinyl)(mesityl)iodonium BF_4 (91 mg, 0.2 mmol) and purified using silica gel chromatography (5% EtOAc/pentane) to yield a colourless sticky solid (29 mg, 58%).

^{1}H NMR (400 MHz, $CDCl_3$) δ: 7.61 – 7.60 (m, 2H), 7.55 (dt, J = 7.8, 1.0 Hz, 1H), 7.38 – 7.34 (m, 2H), 7.32 – 7.28 (m, 1H), 7.23 (d, J = 8.2 Hz, 1H), 7.18 – 7.16 (m, 1H), 7.03 (ddd, J = 8.0, 7.0, 1.1 Hz, 1H), 6.99 (d, J = 2.6 Hz, 1H), 6.40 (d, J = 36.5 Hz, 1H), 3.73 (s, 3H).

^{13}C NMR (101 MHz, $CDCl_3$) δ: 158.1 (d, J = 260.8 Hz), 137.5, 132.5 (d, J = 7.4 Hz), 129.3, 128.9 (d, J = 2.3 Hz), 128.5, 124.3, 124.2, 122.2, 120.9, 120.0, 109.2, 104.1 (d, J = 13.7 Hz), 95.2 (d, J = 13.2 Hz), 29.9.

^{19}F NMR (377 MHz, $CDCl_3$) δ: -108.7 (dd, J = 36.4, 2.4 Hz).

HRMS (EI+) calc: $[M+H]^+$ ($C_{17}H_{14}NF$) 252.1183; measured: 252.1185 = 0.79 ppm

IR (neat) vmax/cm^{-1}: 2920, 1463, 1318, 1075, 1014, 760, 886, 612.

R_f = 0.6 (5% EtOAc/pentane).

(*Z*)-2-(2-fluoro-2-phenylvinyl)-1*H*-indole **(3ak)**

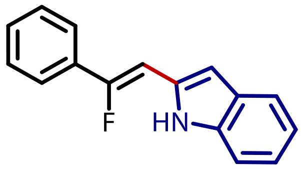

Compound **3ak** was prepared according to general procedure 3a using 1*H*-indole (70 mg, 0.6 mmol) and (*Z*)-(2-fluoro-2-phenylvinyl)(mesityl)iodonium BF_4 (91 mg, 0.2 mmol) and purified using silica gel chromatography (5% EtOAc/pentane) to yield a colourless sticky solid (25 mg, 51%).

^{1}H NMR (400 MHz, $CDCl_3$) δ: 8.89 (s, 1H), 7.65 – 7.58 (m, 3H), 7.45 – 7.37 (m, 4H), 7.21 (ddd, J = 8.2, 7.0, 1.2 Hz, 1H), 7.10 (ddd, J = 8.1, 7.0, 1.1 Hz, 1H), 6.61 (d, J = 2.1 Hz, 1H), 6.50 (d, J = 40.8 Hz, 1H).

^{13}C NMR (101 MHz, $CDCl_3$) δ 156.7 (d, J = 251.7 Hz), 137.1, 132.1, 131.8, 129.2, 128.9 (d, J = 2.3 Hz), 128.1, 124.1, 124.0, 123.0, 120.6, 120.3, 110.9, 104.8 (d, J = 3.7 Hz), 97.9 (d, J = 11.9 Hz).

^{19}F NMR (376 MHz, $CDCl_3$) δ: -117.6 (dd, J = 40.9, 6.2 Hz).

HRMS (ESI+) calc: $[M+H]^+$ ($C_{16}H_{12}NF$) 238.1027; measured: 238.1022 = 2.10 ppm

IR (neat) vmax/cm^{-1}: 3437, 2979, 1410, 1283, 1077, 785, 749, 608.

R_f = 0.5 (5% EtOAc/pentane).

(*Z*)-2-(2-fluoro-2-(4-fluorophenyl)vinyl)-1-methyl-1*H*-indole **(3al)**

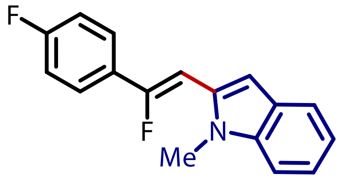

Compound **3al** was prepared according to general procedure 3a using 1-methyl-1*H*-indole (39 mg, 0.3 mmol) and (*Z*)-(2-fluoro-2-(4-fluorophenyl)vinyl)(mesityl)iodonium BF_4 (94 mg, 0.2 mmol) and purified using silica gel chromatography (5% EtOAc/pentane) to yield a colourless sticky solid (38 mg, 70%).

^{1}H NMR (400 MHz, $CDCl_3$) δ: 7.59 – 7.54 (m, 3H), 7.24 – 7.21 (m, 1H), 7.14 (ddd, J = 8.2, 6.9, 1.2 Hz, 1H), 7.07 – 7.01 (m, 3H), 6.97 (dt, J = 2.8, 0.7 Hz, 1H), 6.31 (d, J = 36.4 Hz, 1H), 3.72 (s, 3H).

^{13}C NMR (101 MHz, $CDCl_3$) δ: 163.4 (d, J = 249.9 Hz), 157.3 (d, J = 262.4 Hz), 137.5, 132.4, 128.4, 126.2, 122.3, 120.9, 120.0, 116.1 (d, J = 2.1 Hz), 115.9 (d, J = 2.1 Hz), 109.2, 104.1 (d, J = 13.7 Hz), 95.0 (d, J = 11.1 Hz), 29.9.

^{19}F NMR (377 MHz, $CDCl_3$) δ: -108.1 (dd, J = 36.5, 2.3 Hz), -111.3 (dddd, J = 13.7, 8.5, 5.2, 1.6 Hz).

HRMS (EI+) calc: $[M+H]^+$ ($C_{17}H_{13}F_2N$) 270.1085; measured: 270.1089 = 1.48 ppm difference.

IR (neat) vmax/cm^{-1}: 2952, 1600, 1505, 1467, 1223, 1071, 831, 735, 515.

R_f = 0.6 (5% EtOAc/pentane).

(*Z*)-2-(2-fluoro-2-(4-(trifluoromethyl)phenyl)vinyl)-1-methyl-1*H*-indole **(3am)**

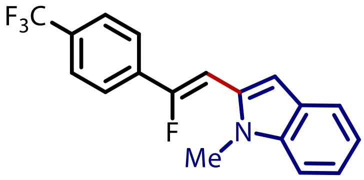

Compound **3am** was prepared according to general procedure 3a using 1-methyl-1*H*-indole (39 mg, 0.3 mmol) and (*Z*)-(2-fluoro-2-(4-(trifluoromethyl)phenyl)vinyl)(mesityl)iodonium BF_4 (104 mg, 0.2 mmol) and purified using silica gel chromatography (5% EtOAc/pentane) to yield a colourless sticky solid (40 mg, 62%).

^{1}H NMR (400 MHz, $CDCl_3$) δ: 7.78 – 7.75 (m, 2H), 7.68 – 7.61 (m, 3H), 7.32 – 7.29 (m, 1H), 7.26 – 7.22 (m, 1H), 7.14 – 7.10 (m, 2H), 6.57 (d, J = 36.1 Hz, 1H), 3.81 (s, 3H).

^{13}C NMR (101 MHz, $CDCl_3$) δ 156.31 (d, J = 260.4 Hz), 137.5, 131.7, 130.9, 130.5, 128.2, 125.7 (q, J = 3.62 Hz), 125.0 (q, J = 271.7 Hz), 124.2, 124.1, 122.6, 121.0, 120.1, 109.2, 105.0 (d, J = 14.2 Hz), 97.2 (d, J = 12.9 Hz), 29.8.

^{19}F NMR (377 MHz, $CDCl_3$) δ: -110.0 (dd, J = 36.0, 2.8 Hz), -62.71.

HRMS (ESI+) calc: $[M+H]^+$ ($C_{18}H_{13}F_4N$) 320.1057; measured: 320.1042 = 4.69 ppm difference.

IR (neat) vmax/cm^{-1}: 2963, 1613, 1463, 1319, 1112, 1066, 825, 735, 614.

R_f = 0.6 (5% EtOAc/pentane).

(*Z*)-2-(2-fluoro-2-(thiophen-3-yl)vinyl)-1-methyl-1*H*-indole **(3an)**

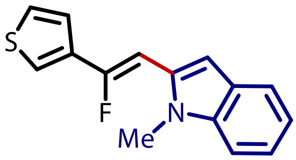

Compound **3an** was prepared according to general procedure 3a using 1-methyl-1*H*-indole (39 mg, 0.3 mmol) and (*Z*)-(2-fluoro-2-(thiophen-3-yl)vinyl)(mesityl)iodonium BF_4 (92 mg, 0.2 mmol) and purified using silica gel chromatography (5% EtOAc/pentane) to yield a colourless sticky solid (36 mg, 70%).

^{1}H NMR (400 MHz, $CDCl_3$) δ: 7.54 (dt, J = 7.9, 1.0 Hz, 1H), 7.49 (dt, J = 3.0, 1.0 Hz, 1H), 7.30 (dt, J = 5.1, 2.9 Hz, 1H), 7.24 – 7.21 (m, 2H), 7.15 – 7.11 (m, 1H), 7.03 (ddd, J = 8.0, 7.0, 1.1 Hz, 1H), 6.94 (s, 1H), 6.20 (d, J = 36.5 Hz, 1H), 3.71 (s, 3H).

^{13}C NMR (101 MHz, $CDCl_3$) δ: 155.1 (d, J = 257.9 Hz), 137.3, 134.7 (d, J = 29.6 Hz), 132.2 (d, J = 3.1 Hz), 128.3, 126.9 (d, J = 2.1 Hz), 123.9 (d, J = 6.6 Hz), 122.1 (d, J = 4.7 Hz), 122.0, 120.7, 119.8, 109.0, 103.7 (d, J = 13.2 Hz), 94.9 (d, J = 12.4 Hz), 29.8.

^{19}F NMR (377 MHz, $CDCl_3$) δ: -105.5 (dd, J = 36.4, 2.9 Hz).

HRMS (ESI+) calc: $[M+H]^+$ ($C_{15}H_{12}FNS$) 258.0747; measured: 258.0745 = 0.77 ppm difference.

IR (neat) vmax/cm^{-1}: 2978, 1394, 1250, 1061, 892.

R_f = 0.7 (5% EtOAc/pentane).

(*E*)-2-(2-fluorododec-1-en-1-yl)-1*H*-indole **(3ao)**

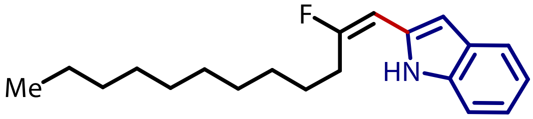

Compound **3ao** was prepared according to general procedure 3a using 1*H*-indole (70 mg, 0.6 mmol) and (*E*)-(2-fluorododec-1-en-1-yl)(mesityl)iodonium BF_4 (104 mg, 0.2 mmol) and purified using silica gel chromatography (5% EtOAc/pentane) to yield a colourless sticky solid (31 mg, 52%).

^{1}H NMR (400 MHz, $CDCl_3$) δ: 7.77 (s, 1H), 7.49 (dq, J = 7.7, 0.9 Hz, 1H), 7.24 (dq, J = 8.0, 1.0 Hz, 1H), 7.10 – 7.00 (m, 2H), 6.31 (dt, J = 2.0, 0.9 Hz, 1H), 6.02 (dd, J = 20.2, 0.8 Hz, 1H), 2.53 (dt, J = 23.3, 7.6 Hz, 2H), 1.63 – 1.55 (m, 2H), 1.35 – 1.19 (m, 14H), 0.82 – 0.79 (m, 3H).

^{13}C NMR (101 MHz, $CDCl_3$) δ: 164.5 (d, J = 256.5 Hz), 136.2, 131.8 (d, J = 15.8 Hz), 129.0, 122.1, 120.3, 110.6, 101.6 (d, J = 3.7 Hz), 99.9 (d, J = 33.1 Hz), 32.0, 30.1 (d, J = 26.5 Hz), 29.7, 29.7, 29.5, 29.5, 29.4, 26.3, 22.8, 14.3.

^{19}F NMR (377 MHz, $CDCl_3$) δ: -95.0 (td, J = 23.4, 20.1 Hz).

HRMS (ESI+) calc: $[M+H]^+$ ($C_{20}H_{28}FN$) 302.2279; measured: 302.2275 = 1.32 ppm difference.

IR (neat) vmax/cm^{-1}: 2991, 2904, 1394, 1250 1062, 891, 749.

R_f = 0.8 (5% EtOAc/pentane).

(*Z*)-3-(2-fluoro-5-phenylpent-1-en-1-yl)-1,2,5-trimethyl-1*H*-pyrrole **(5a)**

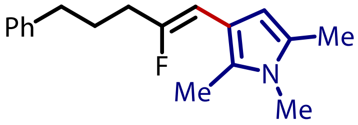

Compound **5a** was prepared according to general procedure 3c using 1,2,5-trimethyl-1*H*-pyrrole (33 mg, 0.3 mmol) and (*Z*)-(2-fluoro-5-phenylpent-1-en-1-yl)(mesityl)iodonium BF_4 (99 mg, 0.2 mmol) and purified using neutral alumina chromatography (5% EtOAc/pentane) to yield a colourless sticky solid (30 mg, 56%).

^{1}H NMR (400 MHz, $CDCl_3$) δ 7.21 – 7.16 (m, 2H), 7.12 – 7.08 (m, 3H), 6.10 (dd, J = 3.5, 1.1 Hz, 1H), 5.25 (d, J = 40.5 Hz, 1H), 3.26 (s, 3H), 2.61 – 2.57 (m, 2H), 2.22 (dt, J = 18.2, 7.3 Hz, 2H), 2.10 (s, 3H), 2.06 (s, 3H), 1.84 – 1.76 (m, 2H).

^{13}C NMR (101 MHz, $CDCl_3$) δ 156.90 (d, J = 257.7 Hz), 142.2, 128.7, 128.5, 125.9, 112.1, 105.7 (d, J = 9.4 Hz), 99.3 (d, J = 12.1 Hz), 35.1, 32.5 (d, J = 27.0 Hz), 30.3, 28.4, 12.4, 10.3.

^{19}F NMR (377 MHz, $CDCl_3$) δ -109.07 (dtd, J = 40.3, 18.3, 3.6 Hz).

HRMS (ESI+) calc: $[M+H]^+$ ($C_{18}H_{22}FN$) 272.1809; measured: 272.1806 = 1.10 ppm difference.

IR (neat) vmax/cm^{-1}: 2922, 1699, 1453, 1378, 1259, 1064, 699.

R_f = 0.7 (5% EtOAc/pentane).

(*Z*)-3-(2-fluoro-5-phenylpent-1-en-1-yl)-1-(4-methoxyphenyl)-2,5-dimethyl-1*H*-pyrrole **(5b)**

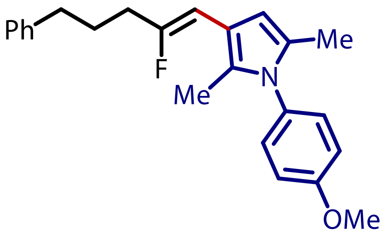

Compound **5b** was prepared according to general procedure 3c using 1-(4-methoxyphenyl)-2,5-dimethyl-1*H*-pyrrole (60 mg, 0.3 mmol) and (*Z*)-(2-fluoro-5-phenylpent-1-en-1-yl)(mesityl)iodonium BF_4 (99 mg, 0.2 mmol) and purified using neutral alumina chromatography (5% EtOAc/pentane) to yield a colourless sticky solid (32 mg, 44%).

^{1}H NMR (400 MHz, $CDCl_3$) δ 7.22 – 7.17 (m, 2H), 7.13 – 7.08 (m, 3H), 7.00 (d, J = 8.9 Hz, 2H), 6.87 (d, J = 8.8 Hz, 2H), 6.28 – 6.18 (m, 1H), 5.30 (d, J = 40.5 Hz, 1H), 3.76 (s, 3H), 2.66 – 2.57 (m, 2H), 2.25 (dt, J = 18.3, 7.5 Hz, 2H), 1.91 (s, 3H), 1.88 (s, 3H), 1.82 (t, J = 7.5 Hz, 2H).

^{13}C NMR (101 MHz, $CDCl_3$) δ 159.1, 157.3 (d, J = 258.1 Hz), 142.2, 131.6, 129.4, 129.3, 128.7, 128.5, 126.5, 125.9, 114.4, 112.7, 106.2 (d, J = 9.4 Hz), 99.2 (d, J = 11.9 Hz), 55.6, 35.1, 32.5 (d, J = 26.8 Hz), 28.4, 12.9, 10.9.

^{19}F NMR (377 MHz, $CDCl_3$) δ -108.44 (dtd, J = 40.3, 18.3, 3.6 Hz).

HRMS (ESI+) calc: $[M+H]^+$ ($C_{24}H_{26}FNO$) 364.2071; measured: 364.2065 = 1.65 ppm difference.

IR (neat) vmax/cm^{-1}: 2920, 1511, 1453, 1292, 1245, 1033, 833, 699, 583.

R_f = 0.5 (5% EtOAc/pentane).

<u>(*Z*)-1-cyclopropyl-3-(2-fluoro-5-phenylpent-1-en-1-yl)-2,5-dimethyl-1*H*-pyrrole **(5c)**</u>

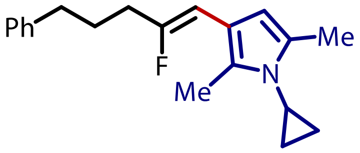

Compound **5c** was prepared according to general procedure 3c using 1-cyclopropyl-2,5-dimethyl-1*H*-pyrrole (41 mg, 0.3 mmol) and (*Z*)-(2-fluoro-5-phenylpent-1-en-1-yl)(mesityl)iodonium BF_4 (99 mg, 0.2 mmol) and purified using neutral alumina chromatography (5% EtOAc/pentane) to yield a colourless sticky solid (20 mg, 34%).

^{1}H NMR (400 MHz, $CDCl_3$) δ 7.33 – 7.29 (m, 2H), 7.23 – 7.20 (m, 3H), 6.19 (d, J = 3.5 Hz, 1H), 5.35 (d, J = 40.6 Hz, 1H), 2.89 (tt, J = 7.0, 4.1 Hz, 1H), 2.73 – 2.69 (m, 2H), 2.38 – 2.30 (m, 2H), 2.30 (s, 3H), 2.28 (s, 3H), 1.95 – 1.88 (m, 2H), 1.07 – 1.01 (m, 2H), 0.90 (dt, J = 4.0, 1.6 Hz, 2H).

^{13}C NMR (101 MHz, $CDCl_3$) δ 157.1 (d, J = 257.9 Hz), 142.2, 130.4 (d, J = 2.3 Hz), 128.7, 128.4, 127.6, 125.9, 112.3, 106.2 (d, J = 9.2 Hz), 99.1 (d, J = 12.1 Hz), 35.1, 32.5 (d, J = 27.1 Hz), 28.4, 25.8, 13.4, 11.1, 7.7.

^{19}F NMR (377 MHz, $CDCl_3$) δ -108.90 (dtd, J = 40.3, 18.4, 3.3 Hz).

HRMS (ESI+) calc: $[M+H]^+$ ($C_{20}H_{24}FN$) 298.1966; measured: 298.1961 = 1.68 ppm difference.

IR (neat) vmax/cm^{-1}: 2938, 1657, 1453, 1394, 1228, 1052, 742, 699.

R_f = 0.6 (5% EtOAc/pentane).

(*Z*)-3-(2-fluoro-5-phenylpent-1-en-1-yl)-2,5-dimethyl-1*H*-pyrrole **(5d)**

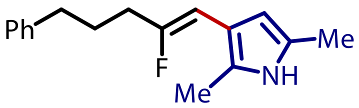

Compound **5d** was prepared according to general procedure 3c using 2,5-dimethyl-1*H*-pyrrole (57 mg, 0.6 mmol) and (*Z*)-(2-fluoro-5-phenylpent-1-en-1-yl)(mesityl)iodonium BF_4 (99 mg, 0.2 mmol) and purified using neutral alumina chromatography (5% EtOAc/pentane) to yield a colourless sticky solid (40 mg, 58%).

^{1}H NMR (400 MHz, $CDCl_3$) δ 7.39 (s, 1H), 7.23 – 7.19 (m, 2H), 7.14 – 7.11 (m, 3H), 6.10 – 6.08 (m, 1H), 5.25 (d, J = 40.6 Hz, 1H), 2.63 – 2.60 (m, 2H), 2.24 (dt, J = 18.1, 7.3 Hz, 2H), 2.14 (s, 3H), 2.11 (s, 3H), 1.83 (dtd, J = 9.3, 7.8, 6.7 Hz, 2H).

^{13}C NMR (101 MHz, $CDCl_3$) δ 157.1 (d, J = 257.9 Hz), 142.2, 128.7, 128.5, 126.1, 125.9, 123.5, 113.3, 106.6 (d, J = 9.3 Hz), 99.0 (d, J = 12.4 Hz), 35.2, 32.4 (d, J = 27.0 Hz), 28.4, 13.0, 11.3.

^{19}F NMR (377 MHz, $CDCl_3$) δ -108.93 (dtd, J = 40.3, 18.2, 3.5 Hz).

HRMS (ESI+) calc: $[M+H]^+$ ($C_{17}H_{20}NF$) 258.1653; measured: 258.1645 = 3.10 ppm difference.

IR (neat) vmax/cm^{-1}: 3668, 2978, 1394, 1251, 1061, 891.

R_f = 0.6 (5% EtOAc/pentane).

(*Z*)-3-(2-fluoro-3,3-dimethylbut-1-en-1-yl)-2,5-dimethyl-1*H*-pyrrole **(5e)**

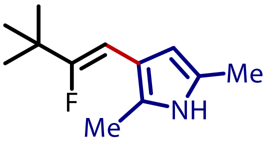

Compound **5e** was prepared according to general procedure 3c using 2,5-dimethyl-1*H*-pyrrole (57 mg, 0.6 mmol) and (*Z*)-(2-fluoro-3,3-dimethylbut-1-en-1-yl)(mesityl)iodonium BF_4 (87 mg, 0.2 mmol) and purified using neutral alumina chromatography (5% EtOAc/pentane) to yield a colourless sticky solid (24 mg, 62%).

^{1}H NMR (400 MHz, $CDCl_3$) δ 7.38 (s, 1H), 6.10 – 6.09 (m, 1H), 5.29 (d, J = 41.8 Hz, 1H), 2.14 (d, J = 0.9 Hz, 3H), 2.12 (s, 3H), 1.11 (d, J = 0.8 Hz, 9H).

^{13}C NMR (101 MHz, $CDCl_3$) δ 164.7 (d, J = 261.0 Hz), 128.1, 126.1, 123.7, 113.3, 106.5 (d, J = 10.2 Hz), 94.9 (d, J = 13.2 Hz), 35.2 (d, J = 24.4 Hz), 27.8 (d, J = 2.5 Hz), 13.0, 11.3.

^{19}F NMR (377 MHz, $CDCl_3$) δ -116.59 (dd, J = 41.7, 3.7 Hz).

HRMS (ESI+) calc: $[M+H]^+$ ($C_{12}H_{18}FN$) 196.1496; measured: 196.1499 = 1.53 ppm difference.

IR (neat) vmax/cm^{-1}: 3572, 2970, 1636, 1394, 1068, 866.

R_f = 0.8 (5% EtOAc/pentane).

(*Z*)-3-(2-cyclohexyl-2-fluorovinyl)-2,5-dimethyl-1*H*-pyrrole **(5f)**

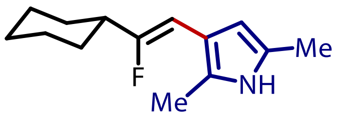

Compound **5f** was prepared according to general procedure 3c using 2,5-dimethyl-1*H*-pyrrole (57 mg, 0.6 mmol) and (*Z*)-(2-cyclohexyl-2-fluorovinyl)(mesityl)iodonium BF_4 (92 mg, 0.2 mmol) and purified using neutral alumina chromatography (5% EtOAc/pentane) to yield a colourless sticky solid (29 mg, 65%).

^{1}H NMR (400 MHz, $CDCl_3$) δ 7.49 (s, 1H), 6.20 – 6.18 (m, 1H), 5.31 (s, 1H), 2.26 – 2.18 (m, 7H), 1.97 – 1.94 (m, 2H), 1.84 – 1.81 (m, 2H), 1.74 – 1.70 (m, 1H), 1.41 – 1.24 (m, 5H).

^{13}C NMR (101 MHz, $CDCl_3$) δ 161.8 (d, J = 259.2 Hz), 126.1 (d, J = 2.5 Hz), 123.5, 113.3, 106.5 (d, J = 9.5 Hz), 96.2 (d, J = 12.4 Hz), 41.4 (d, J = 25.0 Hz), 30.5, 30.4, 26.2, 26.1, 13.0, 11.3.

^{19}F NMR (377 MHz, $CDCl_3$) δ -112.91 (ddd, J = 41.8, 15.4, 3.6 Hz).

HRMS (ESI+) calc: $[M+H]^+$ ($C_{14}H_{20}F$) 222.1653; measured: 222.1648 = 2.25 ppm

difference.

IR (neat) vmax/cm^{-1}: 2927, 2854, 1638, 1448, 1157, 1002.

R_f = 0.7 (5% EtOAc/pentane).

(*Z*)-3-(2-fluoro-2-(4-fluorophenyl)vinyl)-2,5-dimethyl-1*H*-pyrrole **(5g)**

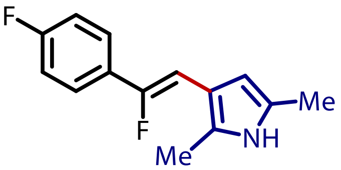

Compound **5g** was prepared according to general procedure 3c using 2,5-dimethyl-1*H*-pyrrole (57 mg, 0.6 mmol) and (*Z*)-(2-fluoro-2-(4-fluorophenyl)vinyl)(mesityl)iodonium BF_4 (94 mg, 0.2 mmol) and purified using neutral alumina chromatography (5% EtOAc/pentane) to yield a colourless sticky solid (28 mg, 60%).

^{1}H NMR (400 MHz, $CDCl_3$) δ 7.59 (s, 1H), 7.55 – 7.50 (m, 2H), 7.04 (t, J = 8.7 Hz, 2H), 6.30 (s, 1H), 6.12 (d, J = 40.3 Hz, 1H), 2.28 (s, 3H), 2.24 (s, 3H).

^{13}C NMR (126 MHz, $CDCl_3$) δ 162.4 (d, J = 247.2 Hz), 153.2 (d, J = 248.7 Hz), 130.3 (dd, J = 28.5, 3.3 Hz), 126.6 (d, J = 2.8 Hz), 125.6 (d, J = 1.7 Hz), 125.2 (t, J = 7.6 Hz), 115.5 (dd, J = 21.9, 2.1 Hz), 113.5, 106.7 (d, J = 10.1 Hz), 99.08 (dd, J = 14.0, 2.1 Hz), 13.0, 11.5.

^{19}F NMR (376 MHz, $CDCl_3$) δ -114.18 (t, J = 7.8 Hz), -121.33 (d, J = 40.2 Hz).

HRMS (ESI+) calc: $[M+H]^+$ ($C_{14}H_{13}F_2N$) 234.1082; measured: 234.1089 = 2.99 ppm difference.

IR (neat) vmax/cm^{-1}: 3668, 2978, 1506, 1394, 1231, 1061, 833.

R_f = 0.5 (5% EtOAc/pentane).

(*Z*)-3-(2-fluoro-2-(thiophen-3-yl)vinyl)-2,5-dimethyl-1*H*-pyrrole **(5h)**

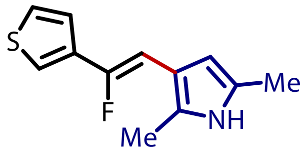

Compound **5h** was prepared according to general procedure 3c using 2,5-dimethyl-1*H*-pyrrole (57 mg, 0.6 mmol) and (*Z*)-(2-fluoro-2-(thiophen-3-yl)vinyl)(mesityl)iodonium BF_4 (92 mg, 0.2 mmol) and purified using neutral alumina chromatography (5% EtOAc/pentane) to yield a colourless sticky solid (27 mg, 62%).

^{1}H NMR (400 MHz, $CDCl_3$) δ 7.50 – 7.49 (m, 1H), 7.26 (dt, J = 3.1, 1.0 Hz, 1H), 7.21 (dt, J = 5.1, 3.0 Hz, 1H), 7.14 (dt, J = 5.0, 1.2 Hz, 1H), 6.21 – 6.19 (m, 1H), 5.94 (d, J = 40.3 Hz, 1H), 2.19 (s, 3H), 2.16 (d, J = 0.9 Hz, 3H).

^{13}C NMR (101 MHz, $CDCl_3$) δ 151.5 (d, J = 246.8 Hz), 136.2 (d, J = 30.6 Hz), 126.6 (d, J = 2.6 Hz), 126.2 (d, J = 2.1 Hz), 125.3 (d, J = 1.6 Hz), 124.0 (d, J = 6.6 Hz), 119.1 (d, J = 4.6 Hz), 113.4, 106.7 (d, J = 9.8 Hz), 99.2 (d, J = 12.9 Hz), 13.0, 11.4.

^{19}F NMR (377 MHz, $CDCl_3$) δ -118.17 (dt, J = 40.6, 3.5 Hz).

HRMS (ESI+) calc: $[M+H]^+$ ($C_{12}H_{12}FNS$) 222.0747; measured: 222.0752 = 2.25 ppm difference.

IR (neat) vmax/cm^{-1}: 3680, 2978, 1394, 1062, 891.

R_f = 0.7 (5% EtOAc/pentane).

(*E*)-3-(2-fluorododec-1-en-1-yl)-2,5-dimethyl-1*H*-pyrrole **(5i)**

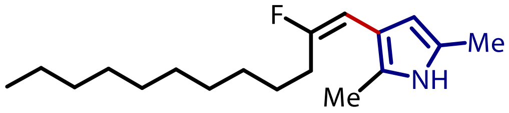

Compound **5i** was prepared according to general procedure 3c using 2,5-dimethyl-1*H*-pyrrole (57 mg, 0.6 mmol) and (*E*)-(2-fluorododec-1-en-1-yl)(mesityl)iodonium BF_4 (104 mg, 0.2 mmol) and purified using neutral alumina chromatography (5% EtOAc/pentane) to yield a colourless sticky solid (22 mg, 39%).

^{1}H NMR (400 MHz, $CDCl_3$) δ 7.44 (s, 1H), 5.84 (d, J = 22.9 Hz, 1H), 5.68 (d, J = 2.7 Hz, 1H), 2.38 (dt, J = 23.8, 7.7 Hz, 2H), 2.15 (s, 3H), 2.08 (s, 3H), 1.56 – 1.47 (m, 2H), 1.32 – 1.19 (m, 14H), 0.83 – 0.79 (m, 3H).

^{13}C NMR (101 MHz, $CDCl_3$) δ 160.0 (d, J = 244.6 Hz), 126.1, 124.4 (d, J = 5.6 Hz), 112.6 (d, J = 13.6 Hz), 105.2 (d, J = 1.6 Hz), 100.8 (d, J = 30.0 Hz), 32.1, 29.8, 29.7, 29.6, 29.5, 29.53, 29.4 (d, J = 27.8 Hz), 26.6, 22.8, 14.3, 13.0, 11.3.

^{19}F NMR (377 MHz, $CDCl_3$) δ -104.46 (q, J = 23.6 Hz).

HRMS (ESI+) calc: $[M+H]^+$ ($C_{18}H_{31}FN$) 280.2441; measured: 280.2432 = 3.2 ppm difference.

IR (neat) vmax/cm^{-1}: 3668, 2971, 1723, 1393, 1251, 1062, 894.

R_f = 0.7 (5% EtOAc/pentane).

(*Z*)-N-(2-fluoro-3-(1-methyl-1*H*-pyrrolo[2,3-b]pyridin-2-yl)allyl)-*N*-(4-fluorophenyl)-4-methylbenzenesulfonamide **(7a)**

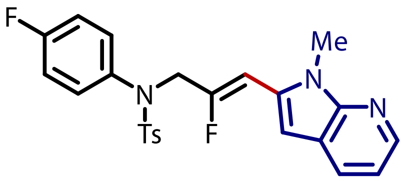

Compound **7a** was prepared according to general procedure 3a using 1-methyl-1*H*-pyrrolo[2,3-b]pyridine (40 mg, 0.3 mmol) and (*E*)-(2-fluorododec-1-en-1-yl)(mesityl)iodonium BF_4 (131 mg, 0.2 mmol) at 70 ^{0}C and purified using neutral alumina chromatography (40% EtOAc/pentane) to yield a colourless sticky solid (25 mg, 27%).

^{1}H NMR (400 MHz, $CDCl_3$) δ 8.18 (dd, J = 4.7, 1.6 Hz, 1H), 7.73 (dd, J = 7.9, 1.5 Hz, 1H), 7.44 (d, J = 8.3 Hz, 2H), 7.18 (t, J = 1.1 Hz, 1H), 7.02 – 6.99 (m, 2H), 6.94 – 6.88 (m, 4H), 6.64 (d, J = 2.2 Hz, 1H), 5.72 (d, J = 34.9 Hz, 1H), 4.40 (d, J = 14.8 Hz, 2H), 3.74 (s, 3H), 2.43 (s, 3H).

^{13}C NMR (126 MHz, $CDCl_3$) δ 162.4 (d, J = 249.2 Hz), 156.3 (d, J = 272.5 Hz), 144.2, 143.4, 135.6, 134.8 (d, J = 3.3 Hz), 131.2 (d, J = 8.8 Hz), 129.8, 129.7 (d, J = 1.3 Hz), 128.5, 127.9, 120.7, 116.5, 116.34, 116.31, 101.7 (d, J = 12.6 Hz), 99.9 (d, J = 9.0 Hz), 53.0, 52.8, 28.2, 21.7.

^{19}F NMR (377 MHz, $CDCl_3$) δ -101.23 (dt, J = 32.2, 14.7 Hz), -111.94 (tt, J = 8.2, 4.8 Hz).

HRMS (ESI+) calc: $[M+H]^+$ ($C_{24}H_{21}F_2N_3O_2S$) 454.1395; measured: 454.1378 = 3.74 ppm difference.

IR (neat) vmax/cm^{-1}: 2935,1597, 1505, 1348, 1162, 1091, 812, 1552.

R$_f$ = 0.5 (50% EtOAc/pentane).

(*Z*)-2-(2-fluoro-5-phenylpent-1-en-1-yl)-1,3,5-trimethoxybenzene **(9a)**

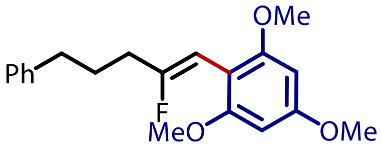

Compound **9a** was prepared according to general procedure 3d using 1,3,5-trimethoxy benzene (51 mg, 0.3 mmol) and (*Z*)-(2-fluoro-5-phenylpent-1-en-1-yl)(mesityl)iodonium BF_4 (99 mg, 0.2 mmol) and purified using neutral alumina chromatography (40% EtOAc/pentane) to yield a colourless sticky solid (32 mg, 48%).

^{1}H NMR (600 MHz, $CDCl_3$) δ 6.18 (s, 2H), 5.39 (d, J = 40.1 Hz, 1H), 3.84 (s, 3H), 3.84 (s, 6H), 2.76 (t, J = 7.34 Hz, 2H), 2.39 (dt, J = 16.8, 7.3 Hz, 1H), 1.96 (p, J = 7.4 Hz, 1H).

^{13}C NMR (151 MHz, $CDCl_3$) δ 160.7, 159.7 (d, J = 263.0 Hz), 158.7, 142.3, 128.7, 128.5, 125.9, 104.1, 96.9 (d, J = 14.2 Hz), 90.9, 56.0, 55.5, 34.9, 32.2 (d, J = 27.3 Hz), 28.1.

^{19}F NMR (377 MHz, $CDCl_3$) δ -97.62 (dt, J = 40.1, 16.8 Hz).

HRMS (ESI+) calc: $[M]^+$ ($C_{20}H_{23}O_3F$) 330.1626; measured: 330.1620 = 1.82 ppm difference.

IR (neat) vmax/cm^{-1}: 2975,1606, 1454, 1410, 1228, 1061, 889.

R$_f$ = 0.7 (5% EtOAc/pentane).

(*E*)-2-(4-fluorostyryl)-1-methyl-1*H*-indole **(13a)**

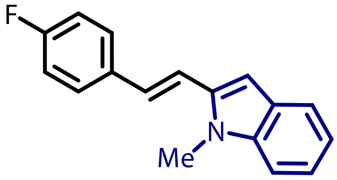

Compound **13a** was prepared according to general procedure 8 using 1-methyl-1*H*-indole (39 mg, 0.15 mmol) and (*E*)-(4-fluorostyryl)(mesityl)iodonium BF_4 (45 mg, 0.1 mmol) and purified using silica gel column chromatography (40% EtOAc/pentane) to yield a colourless sticky solid (19 mg, 74%).

^{1}H NMR (400 MHz, $CDCl_3$) δ 7.51 (dt, *J* = 7.9, 1.0 Hz, 1H), 7.44 – 7.40 (m, 2H), 7.24 – 7.21 (m, 1H), 7.15 – 6.97 (m, 6H), 6.71 (d, *J* = 0.8 Hz, 1H), 3.74 (s, 3H).

^{19}F NMR (377 MHz, $CDCl_3$) δ -113.73 (tt, *J* = 8.6, 5.4 Hz).

These data are consistent with those previously reported.

1-(1-Methyl-1*H*-indol-2-yl)-5-phenylpentan-2-one **(14a)**

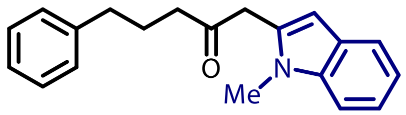

Compound **14a** was prepared according to general procedure 4 using 1-methyl-1*H*-indole (39 mg, 0.3 mmol) and (*Z*)-(2-fluoro-5-phenylpent-1-en-1-yl)(mesityl)iodonium BF_4 (99 mg, 0.2 mmol) and purified using silica gel chromatography (10% EtOAc/pentane) to yield a colourless oil (24 mg, 41%).

^{1}H NMR (400 MHz, $CDCl_3$) δ 7.49 (dt, J = 7.8, 1.0 Hz, 1H), 7.22 – 7.09 (m, 5H), 7.04 – 7.00 (m, 3H), 6.28 (d, J = 0.8 Hz, 1H), 3.75 (s, 2H), 3.54 (s, 3H), 2.51 – 2.47 (m, 2H), 2.43 (t, J = 7.3 Hz, 2H), 1.81 (p, J = 7.4 Hz, 2H).

^{13}C NMR (101 MHz, $CDCl_3$) δ 206.9, 141.5, 137.9, 133.2, 128.6, 128.5, 127.8, 126.1, 121.5, 120.4, 119.8, 109.3, 102.1, 42.3, 40.8, 35.0, 30.0, 25.2.

HRMS (ESI+) calc: $[M+H]^+$ ($C_{20}H_{21}NO$) 292.1696; measured: 292.1693 = 1.03 ppm difference.

IR (neat) vmax/cm^{-1}: 2930, 1712, 1601, 1453, 1115, 848, 739, 699.

R_f = 0.5 (15% EtOAc/pentane).

<u>1-(1-Methyl-1*H*-indol-2-yl)dodecan-2-one **(14b)**</u>

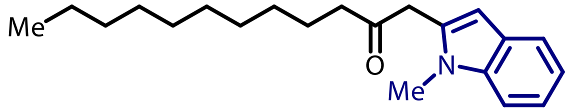

Compound **14b** was prepared according to general procedure 4 using 1-methyl-1*H*-indole (39 mg, 0.3 mmol) and (*Z*)-(2-fluorododec-1-en-1-yl)(mesityl)iodonium BF_4 (104 mg, 0.2 mmol) and purified using silica gel chromatography (10% EtOAc/pentane) to yield a colourless oil (24 mg, 38%).

^{1}H NMR (400 MHz, $CDCl_3$) δ 7.56 (dt, J = 7.8, 1.0 Hz, 1H), 7.30 – 7.20 (m, 1H), 7.20 (ddd, J = 8.2, 7.0, 1.2 Hz, 1H), 7.09 (ddd, J = 7.9, 7.0, 1.1 Hz, 1H), 6.39 (d, J = 0.9 Hz, 1H), 3.85 (s, 2H), 3.64 (s, 3H), 2.48 (t, J = 7.4 Hz, 2H), 1.29 – 1.23 (m, 16H), 0.88 (t, J = 6.8 Hz, 3H).

^{13}C NMR (101 MHz, $CDCl_3$) δ 207.3, 133.2, 127.7, 121.4, 120.2, 119.6, 109.2, 101.9, 42.2, 41.6, 31.9, 29.9, 29.5, 29.4, 29.4, 29.1, 23.7, 22.7, 14.1.

HRMS (ESI+) calc: $[M+H]^+$ ($C_{21}H_{31}NO$) 314.2478; measured: 314.2476 = 0.6 ppm difference.

IR (neat) vmax/cm^{-1}: 2920, 1715, 1557, 1464, 1052, 743, 725.

R_f = 0.6 (15% EtOAc/pentane).

1-(3-Acetyl-1-methyl-1*H*-indol-2-yl)-5-phenylpentan-2-one **(15a)**

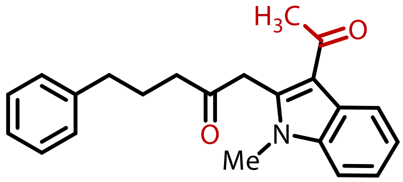

Compound **15a** was prepared according to general procedure 5 using (Z)-2-(2-fluoro-5-phenylpent-1-en-1-yl)-1-methyl-1H-indole (58 mg, 0.2 mmol) and acetic acid (36 mg, 0.6 mmol) and purified using silica gel chromatography (10% EtOAc/pentane) to yield a colourless oil (27 mg, 40%).

^{1}H NMR (400 MHz, $CDCl_3$) δ 7.89 – 7.87 (m, 1H), 7.39 – 7.36 (m, 1H), 7.29 (dt, J = 6.4, 3.7 Hz, 2H), 7.27 – 7.23 (m, 2H), 7.18 – 7.13 (m, 3H), 4.40 (s, 2H), 3.68 (s, 3H), 2.71 – 2.67 (m, 5H), 2.62 (t, J = 7.6 Hz, 2H), 1.94 (p, J = 7.5 Hz, 2H).

^{13}C NMR (101 MHz, $CDCl_3$) δ 206.1, 194.8, 141.8, 141.6, 137.2, 128.6, 128.5, 126.0, 126.0, 122.6, 122.3, 120.7, 114.5, 110.3, 42.2, 40.7, 35.2, 31.8, 29.9, 25.2.

HRMS (ESI+) calc: $[M+H]^+$ ($C_{22}H_{23}NO_2$) 334.1802; measured: 334.1795 = 2.09 ppm difference.

IR (neat) vmax/cm^{-1}: 2974, 1719, 1640, 1511, 1416, 1213, 1088, 738, 700, 503.

R_f = 0.6 (15% EtOAc/pentane).

1-(1-Methyl-3-(2,2,2-trifluoroacetyl)-1*H*-indol-2-yl)-5-phenylpentan-2-one **(15b)**

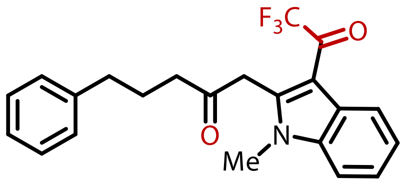

Compound **15b** was prepared according to general procedure 5 using (Z)-2-(2-fluoro-5-phenylpent-1-en-1-yl)-1-methyl-1H-indole (58 mg, 0.1 mmol) and 2,2,2-trifluoroacetic acid (68 mg, 0.6 mmol) and purified using silica gel chromatography (10% EtOAc/pentane) to yield a colourless oil (44 mg, 57%).

^{1}H NMR (400 MHz, $CDCl_3$) δ 7.98 (ddt, J = 5.4, 2.7, 1.4 Hz, 1H), 7.41 – 7.37 (m, 1H), 7.36 – 7.31 (m, 2H), 7.30 – 7.24 (m, 2H), 7.18 (tt, J = 8.0, 1.5 Hz, 3H), 4.34 (s, 2H), 3.72 (s, 3H), 2.74 (t, J = 7.3 Hz, 2H), 2.65 (t, J = 7.6 Hz, 2H), 1.98 (p, J = 7.5 Hz, 2H).

^{13}C NMR (101 MHz, $CDCl_3$) δ 204.7, 175.5 (q, J = 36.6 Hz), 147.1, 141.6, 137.3, 128.6, 128.5, 126.1, 124.3, 123.7, 123.6, 121.0 (q, J = 5.2 Hz), 117.3 (q, J = 289.5 Hz), 110.3, 108.5, 42.5, 41.0, 35.1, 30.4, 25.0.

^{19}F NMR (376 MHz, $CDCl_3$) δ -74.62.

HRMS (ESI+) calc: $[M+H]^+$ ($C_{22}H_{20}NO_2F_3$) 387.1441; measured: 387.1435 = 1.55 ppm

difference.

IR (neat) vmax/cm^{-1}: 2934, 1708, 1644, 1484, 1348, 1158, 1072, 878, 748, 583, 545.

R$_f$ = 0.4 (15% EtOAc/pentane).

1-(3-(2,2-Difluoro-2-phenylacetyl)-1-methyl-1*H*-indol-2-yl)-5-phenylpentan-2-one **(15c)**

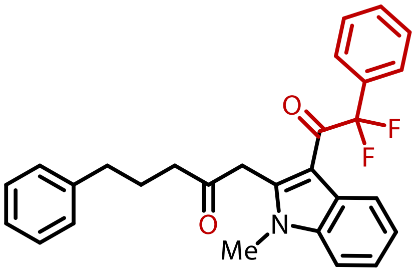

Compound **15c** was prepared according to general procedure 5 using (Z)-2-(2-fluoro-5-phenylpent-1-en-1-yl)-1-methyl-1H-indole (58 mg, 0.1 mmol) and 2,2-difluoro-2-phenylacetic acid (103 mg, 0.6 mmol) and purified using silica gel chromatography (10% EtOAc/pentane) to yield a colourless oil (35 mg, 38%).

^{1}H NMR (400 MHz, $CDCl_3$) δ 7.81 (d, J = 8.2 Hz, 1H), 7.62 – 7.57 (m, 2H), 7.47 – 7.34 (m, 4H), 7.28 – 7.23 (m, 3H), 7.19 – 7.4 (m, 4H), 4.30 (s, 2H), 3.69 (s, 3H), 2.71 (t, J = 7.3 Hz, 2H), 2.63 (t, J = 7.6 Hz, 2H), 1.95 (p, J = 7.5 Hz, 2H).

^{13}C NMR (151 MHz, $CDCl_3$) δ 205.3, 185.3 (t, J = 32.6 Hz), 145.6, 141.7, 137.3, 133.61 (t, J = 25.4 Hz), 131.2, 130.9, 128.8, 128.73, 128.7, 128.5, 126.4 (t, J = 5.6 Hz), 126.1, 124.8, 123.2, 122.9, 122.3 (t, J = 6.8 Hz), 117.0 (t, J = 250.7 Hz), 111.0, 110.0, 42.2, 41.1, 35.2, 30.3, 25.1.

^{19}F NMR (376 MHz, $CDCl_3$) δ -97.00.

HRMS (ESI+) calc: $[M+H]^+$ ($C_{28}H_{25}NO_2F_2$) 446.1926; measured: 446.1921 = 1.12 ppm difference.

IR (neat) vmax/cm^{-1}: 2944, 1720, 1645, 1453, 1410, 1262, 1051, 748, 698.

R_f = 0.5 (15% EtOAc/pentane).

<u>1-(3-(2,2-Difluoroacetyl)-1-methyl-1*H*-indol-2-yl)-5-phenylpentan-2-one **(15d)**</u>

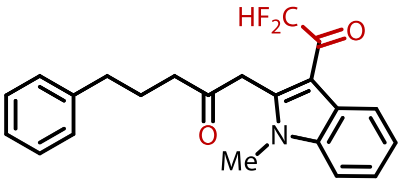

Compound **15d** was prepared according to general procedure 5 using (Z)-2-(2-fluoro-5-phenylpent-1-en-1-yl)-1-methyl-1H-indole (58 mg, 0.1 mmol) and 2,2-difluoroacetic acid (58 mg, 0.6 mmol) and purified using silica gel chromatography (10% EtOAc/pentane) to yield a colourless oil (33 mg, 44%).

^{1}H NMR (600 MHz, $CDCl_3$) δ 7.89 – 7.88 (m, 1H), 7.44 – 7.42 (m, 1H), 7.39 – 7.35 (m, 2H), 7.30 – 7.28 (m, 2H), 7.22 – 7.18 (m, 3H), 6.47 (t, J = 54.1 Hz, 1H), 4.42 (s, 2H), 3.75 (s, 3H), 2.76 (t, J = 7.3 Hz, 2H), 2.67 (t, J = 7.6 Hz, 2H), 2.00 (p, J = 7.4 Hz, 2H).

^{13}C NMR (151 MHz, $CDCl_3$) δ 205.1, 183.0 (t, J = 24.9 Hz), 145.5, 141.6, 137.4, 128.7, 128.5, 126.1, 124.7, 123.5, 123.5, 120.9 (t, J = 4.0 Hz), 110.51, 110.5 (t, J = 250.8 Hz), 110.3, 42.5, 40.9, 35.2, 30.4, 25.1.

^{19}F NMR (376 MHz, $CDCl_3$) δ -126.17 (d, J = 54.1 Hz).

HRMS (ESI+) calc: $[M+H]^+$ ($C_{22}H_{21}NO_2F_2$) 370.1613; measured: 370.1607 = 1.62 ppm

difference.

IR (neat) vmax/cm^{-1}: 2947, 1722, 1654, 1453, 1405, 1258, 1060, 746.

R$_f$ = 0.5 (15% EtOAc/pentane).

<u>2-(phenylethynyl)-1*H*-indole **(16aj)**</u>

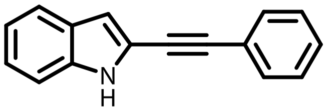

Compound **16aj** was prepared according to general procedure 6 using (Z)-2-(2-fluoro-5-phenylpent-1-en-1-yl)-1-methyl-1H-indole (29.3 mg, 0.1 mmol) and PhMgBr (0.24 mmol) and purified using silica gel chromatography (10% EtOAc/pentane) to yield a colourless oil (17 mg, 77%).

^{1}H NMR (400 MHz, $CDCl_3$) δ 8.25 (s, 1H), 7.61 (dd, J = 7.9, 1.0 Hz, 1H), 7.56 – 7.53 (m, 2H), 7.39 – 7.33 (m, 4H), 7.25 – 7.22 (m, 1H), 7.13 (ddd, J = 8.0, 7.0, 1.0 Hz, 1H), 6.84 (dd, J = 2.1, 1.0 Hz, 1H).

^{13}C NMR (101 MHz, $CDCl_3$) δ 136.3, 131.6, 128.8, 128.6, 127.9, 123.7, 122.7, 121.0, 120.6, 118.9, 110.9, 109.0, 92.7, 81.9.

HRMS (ESI+) calc: $[M+H]^+$ ($C_{16}H_{11}N$) 218.0964; measured: 218.0962 = 0.92 ppm

difference.

IR (neat) vmax/cm^{-1}: 3378, 2975, 1596, 1397, 1062, 797, 1074, 748, 529.

(*Z*)-2-(2,5-diphenylpent-1-en-1-yl)-1-methyl-1*H*-indole **(17a)**

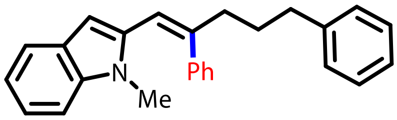

Compound **17a** was prepared according to general procedure 7 using (Z)-2-(2-fluoro-5-phenylpent-1-en-1-yl)-1-methyl-1H-indole (29.3 mg, 0.1 mmol) and PhMgBr (0.24 mmol) and purified using silica gel chromatography (10% EtOAc/pentane) to yield a colourless oil (21 mg, 58%).

^{1}H NMR (400 MHz, $CDCl_3$) δ 7.26 – 7.23 (m, 2H), 7.22 – 7.18 (m, 5H), 7.14 – 7.08 (m, 6H), 7.03 (ddd, J = 8.2, 7.0, 1.2 Hz, 1H), 6.89 (ddd, J = 7.9, 7.0, 1.1 Hz, 1H), 6.39 (s, 1H), 3.56 (s, 3H), 2.63 – 2.59 (m, 2H), 2.54 (td, J = 7.5, 1.3 Hz, 2H), 1.76 – 1.68 (m, 2H).

^{13}C NMR (101 MHz, $CDCl_3$) δ 142.3, 138.8, 137.8, 134.1, 128.6, 128.6, 128.5, 128.5, 126.0, 121.3, 120.2, 119.8, 119.4, 109.2, 98.0, 35.5, 33.0, 31.0, 29.9.

HRMS (MALDI+) calc: $[M+Na]^+$ ($C_{26}H_{25}N$) 374.1879; measured: 374.1871 = 2.14 ppm

difference.

IR (neat) vmax/cm^{-1}: 2923, 1701, 1602, 1465, 1368, 1239, 1074, 743, 699.

NMR spectra of novel compound

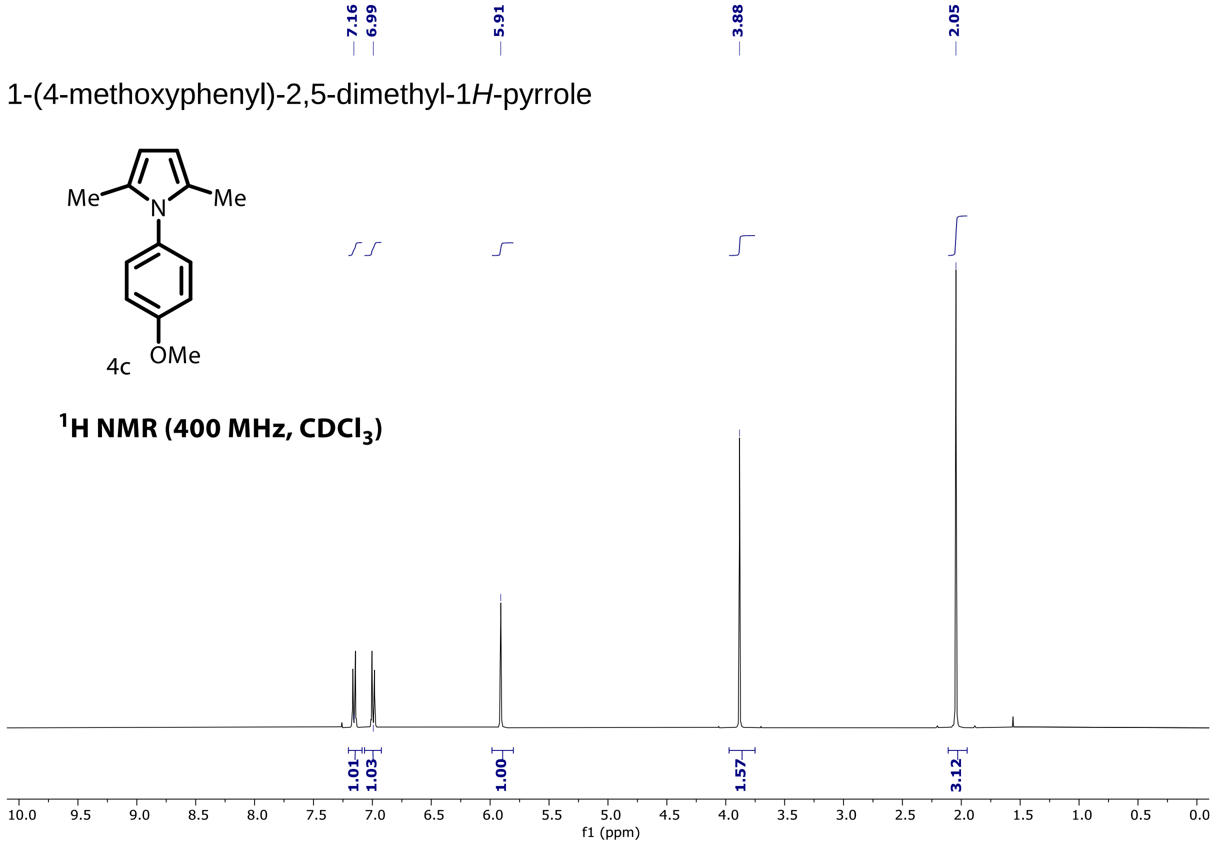
7.16
6.99
5.91
3.88
2.05
1-(4-methoxyphenyl)-2,5-dimethyl-1H-pyrrole
Me
N
Me
4c
OMe
1H NMR (400 MHz, CDCl3)
1.01
1.03
1.00
1.57
3.12
10.0 9.5 9.0 8.5 8.0 7.5 7.0 6.5 6.0 5.5 5.0 4.5 4.0 3.5 3.0 2.5 2.0 1.5 1.0 0.5 0.0
f1 (ppm)

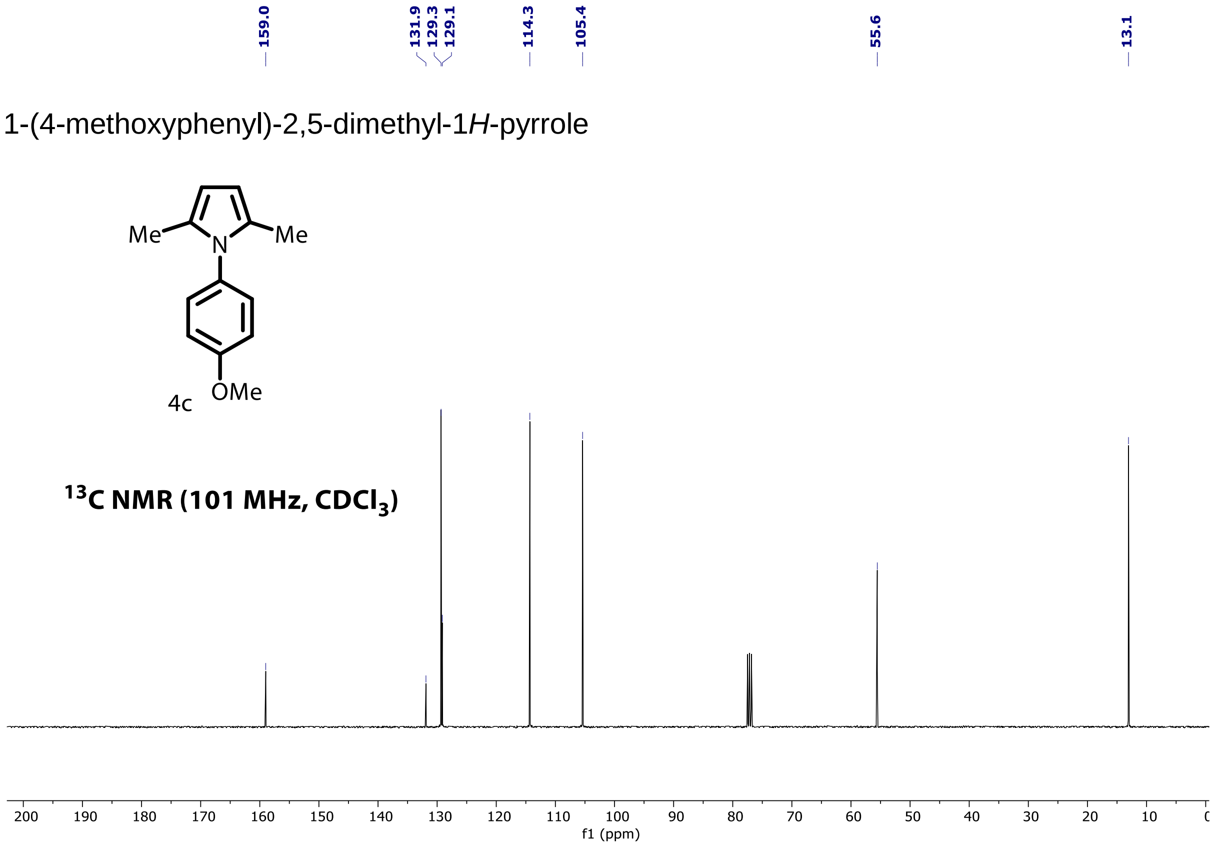
159.0
131.9
129.3
129.1
114.3
105.4
55.6
13.1
1-(4-methoxyphenyl)-2,5-dimethyl-1H-pyrrole
Me
N
Me
4c
OMe
13C NMR (101 MHz, CDCl3)
200 190 180 170 160 150 140 130 120 110 100 90 80 70 60 50 40 30 20 10 0
f1 (ppm)

1-cyclopropyl-2,5-dimethyl-1*H*-pyrrole

4d

^{1}H NMR (400 MHz, CDCl$_3$)

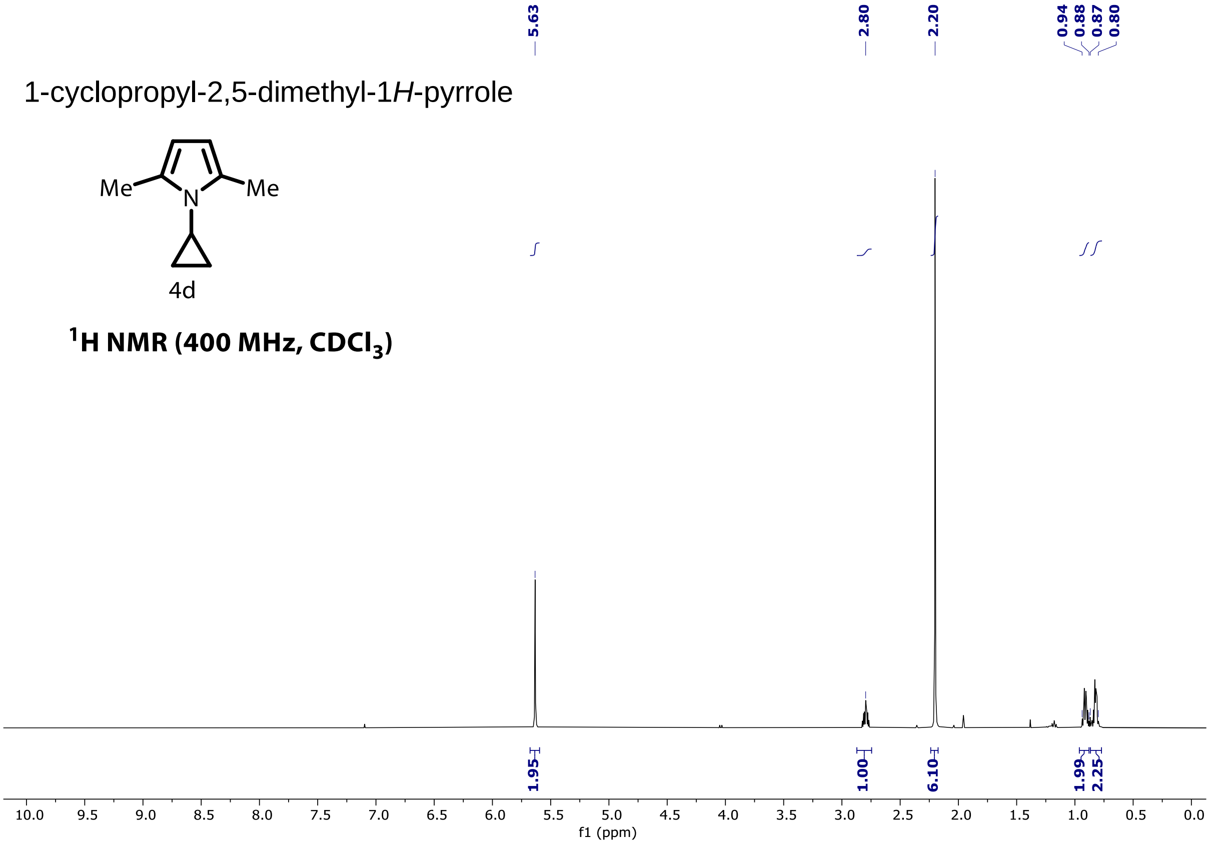

1-cyclopropyl-2,5-dimethyl-1*H*-pyrrole

4d

^{13}C NMR (101 MHz, CDCl$_3$)

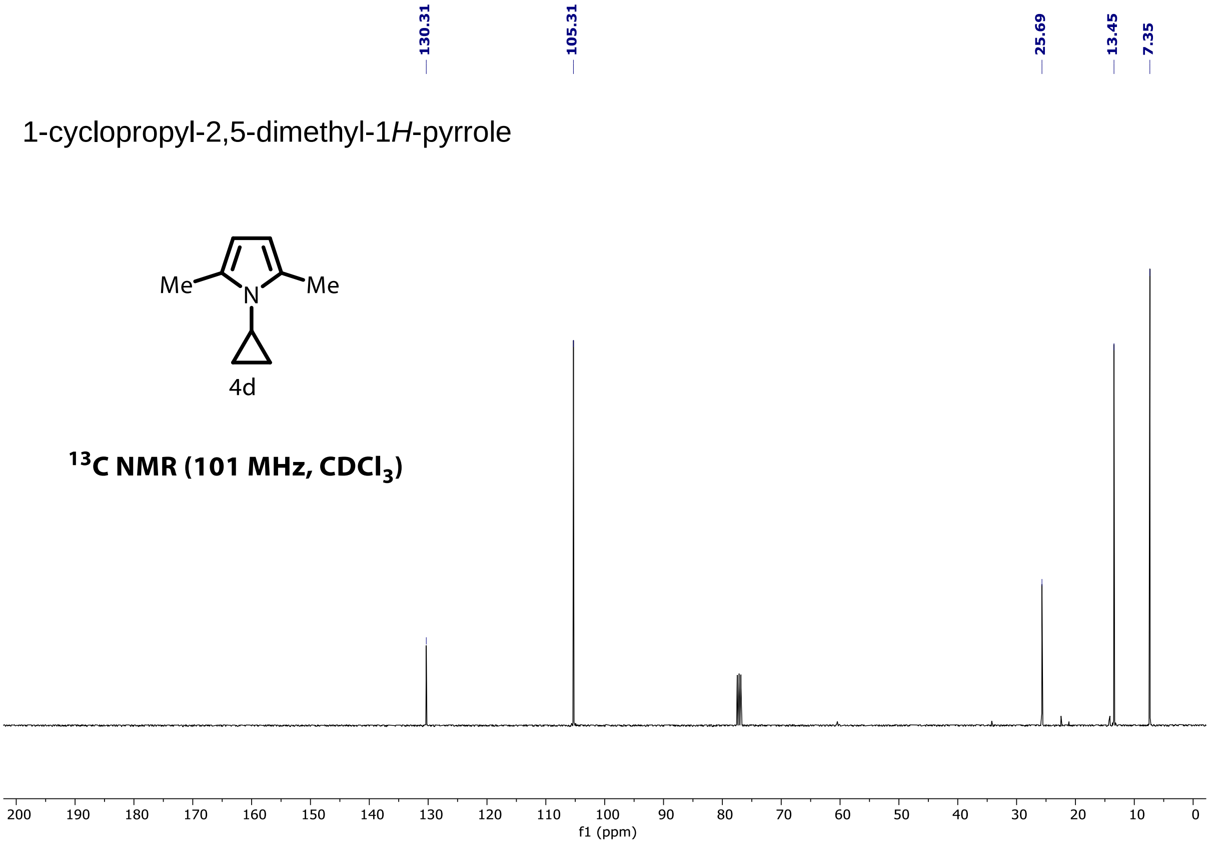

(*Z*)-(2-fluoro-5-phenylpent-1-en-1-yl)(4-nitrophenyl)iodonium BF_4

^{1}H NMR (400 MHz, $CDCl_3$)

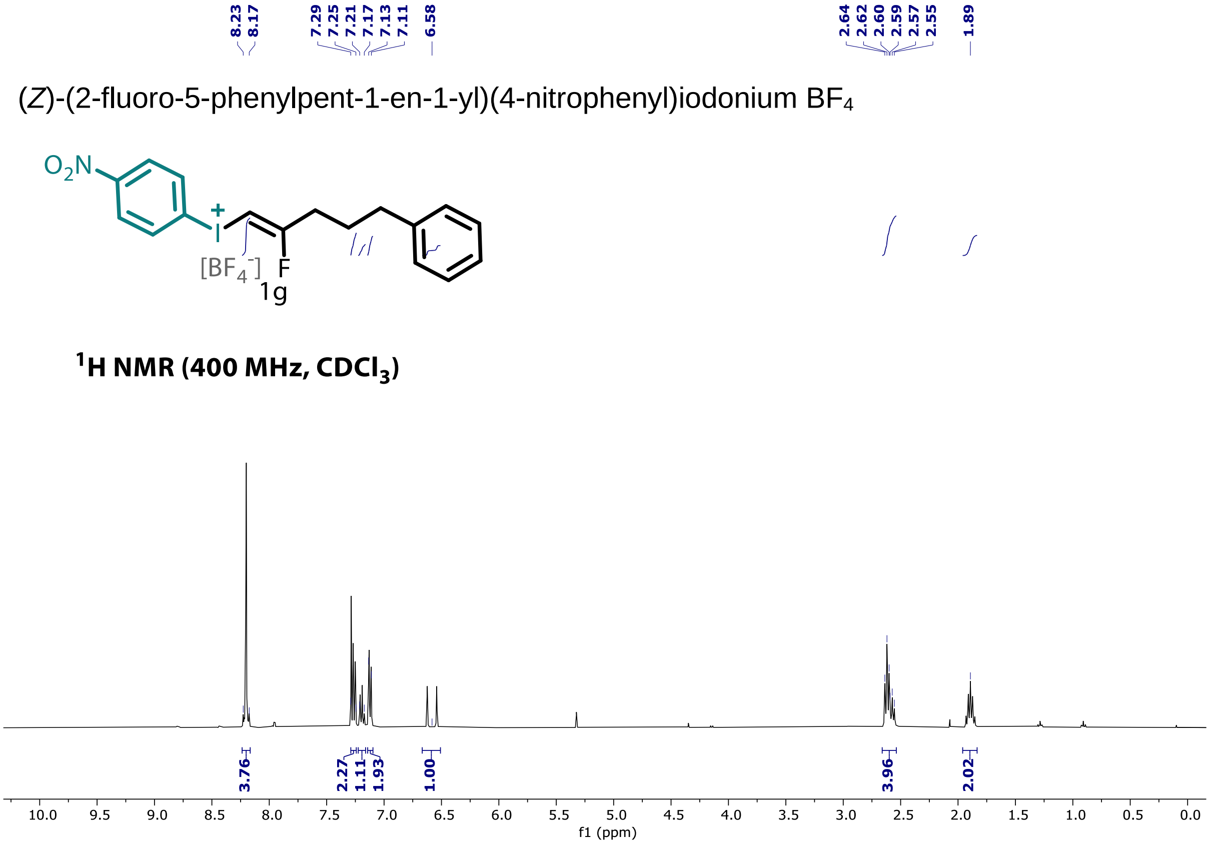

(*Z*)-(2-fluoro-5-phenylpent-1-en-1-yl)(4-nitrophenyl)iodonium BF_4

^{13}C NMR (101 MHz, $CDCl_3$)

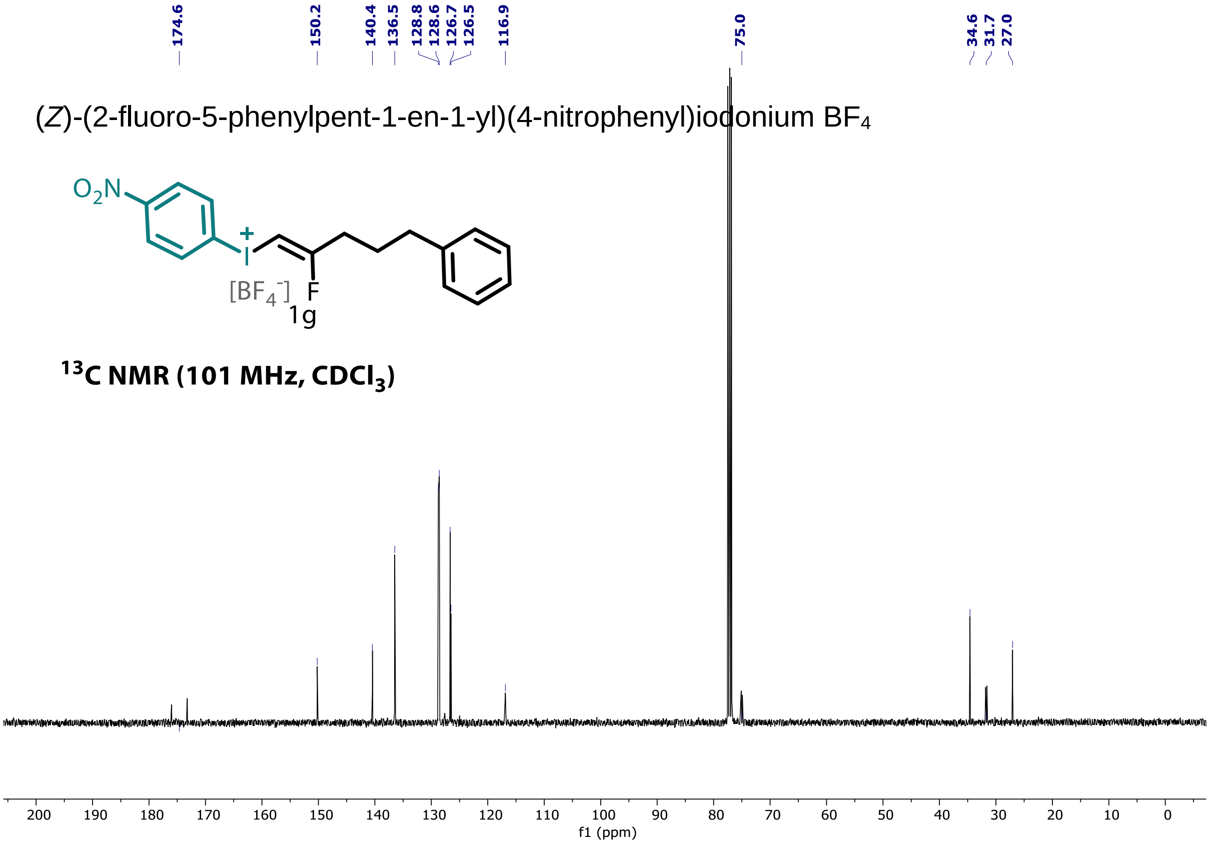

(*Z*)-(2-fluoro-5-phenylpent-1-en-1-yl)(4-nitrophenyl)iodonium BF_4

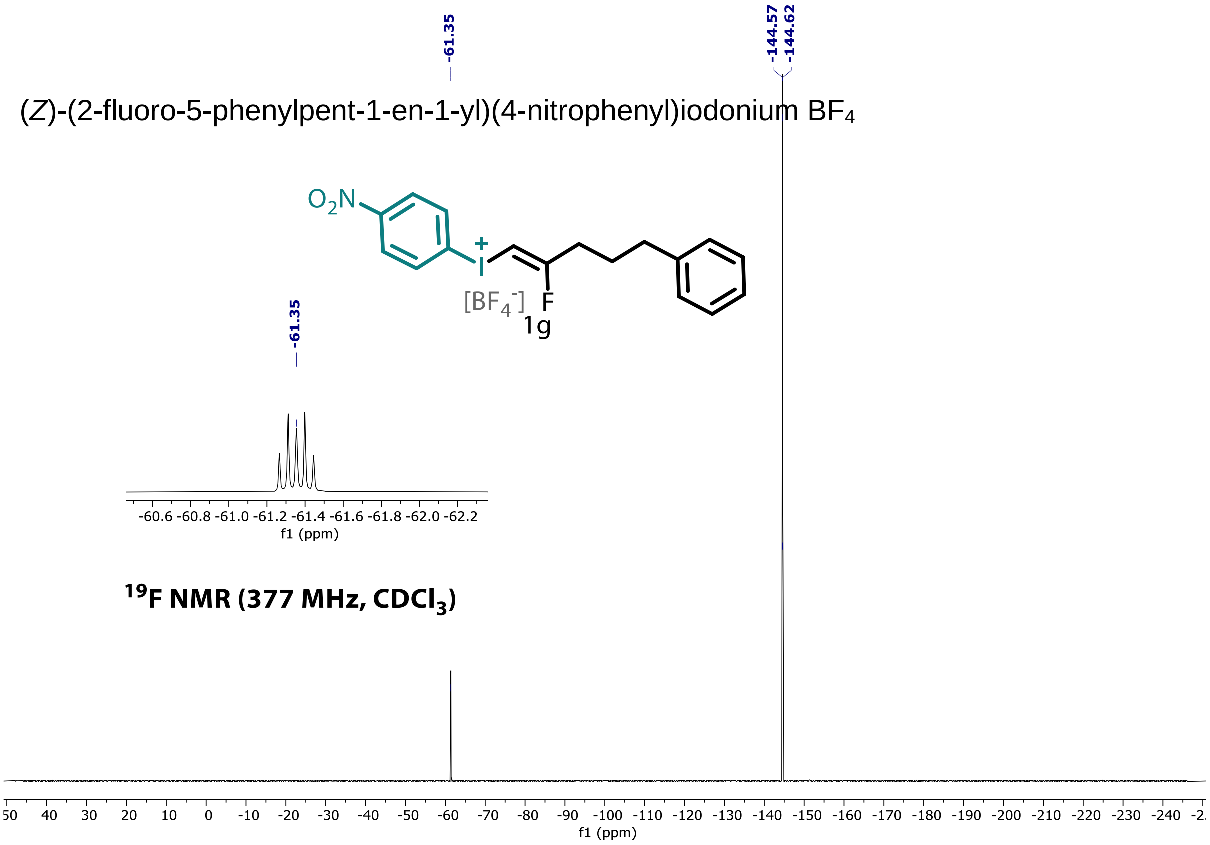

^{19}F NMR (377 MHz, $CDCl_3$)

(*Z*)-(2-fluoro-5-phenylpent-1-en-1-yl)(4-(trifluoromethyl)phenyl)iodonium BF_4

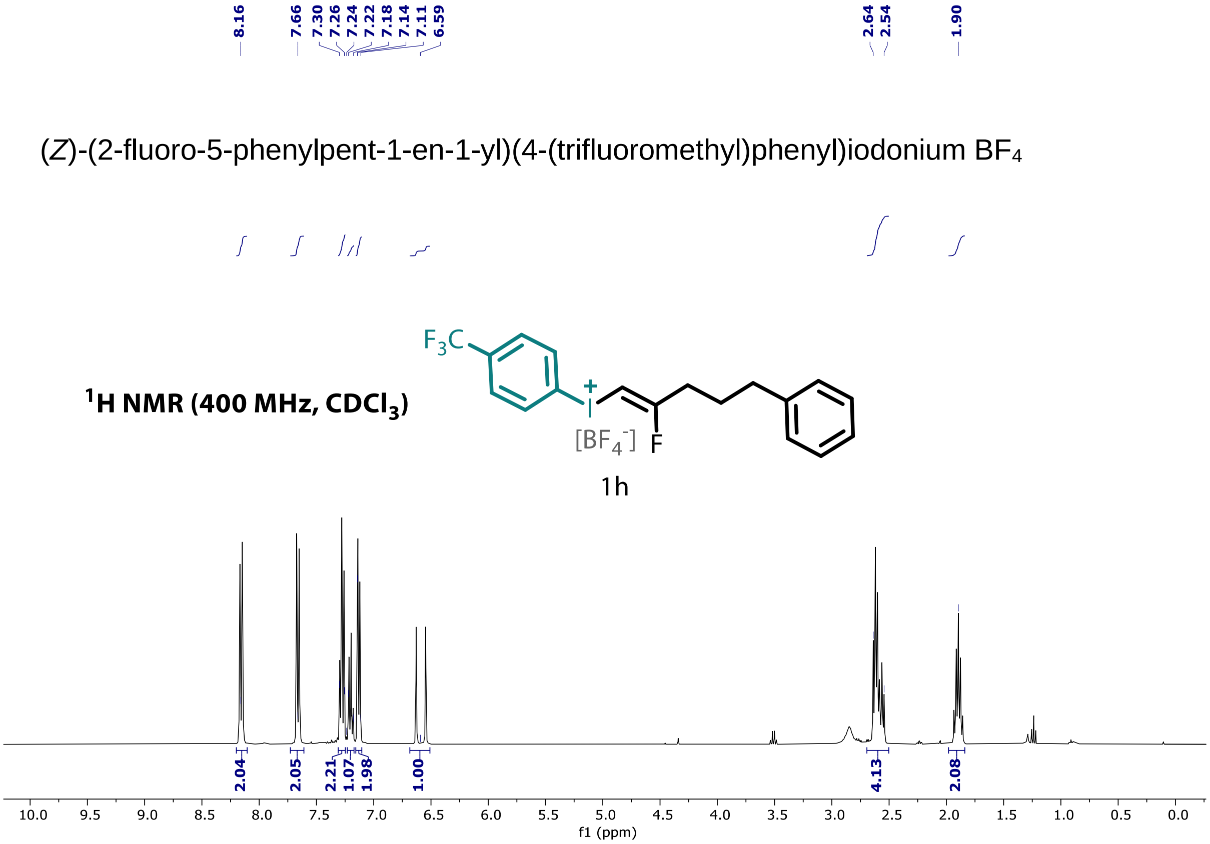

^{1}H NMR (400 MHz, $CDCl_3$)

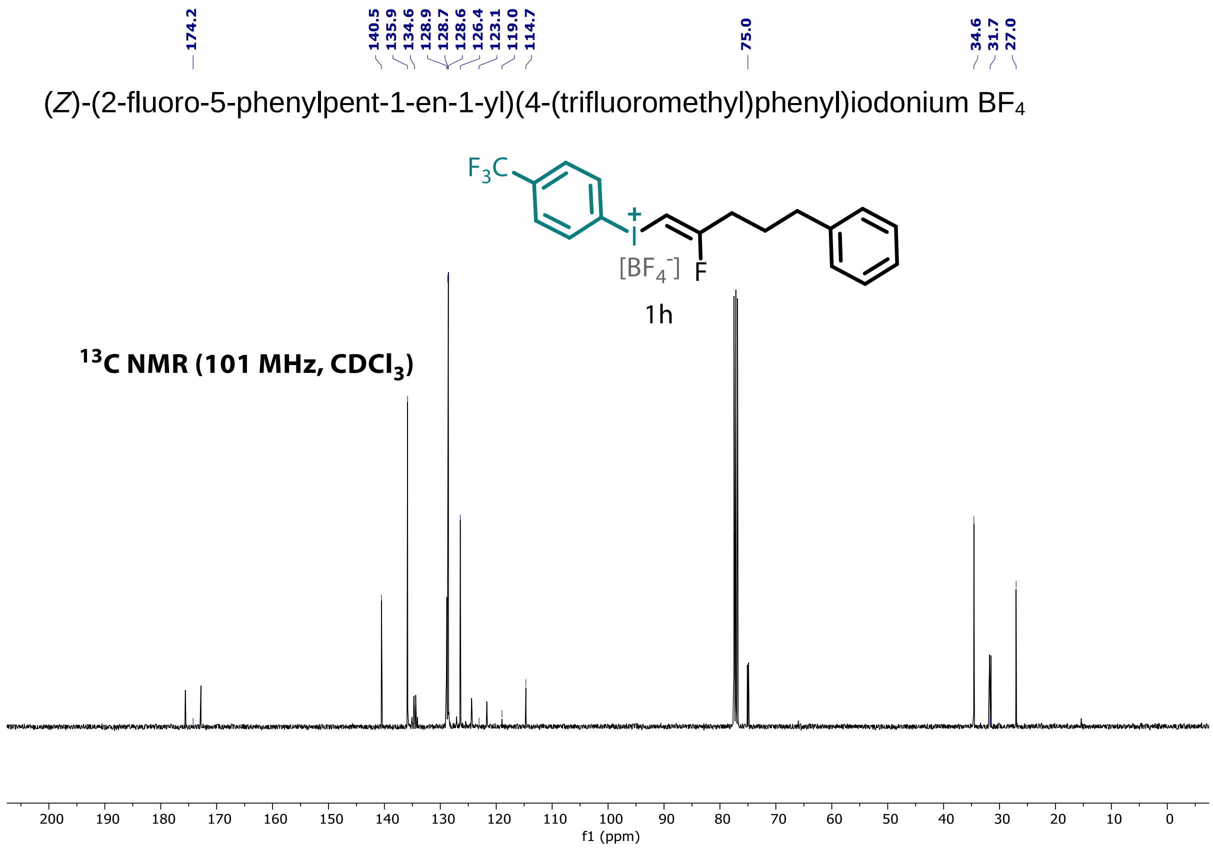
174.2
140.5
135.9
134.6
128.9
128.7
128.6
126.4
123.1
119.0
114.7
75.0
34.6
31.7
27.0
(Z)-(2-fluoro-5-phenylpent-1-en-1-yl)(4-(trifluoromethyl)phenyl)iodonium BF4
F3C
[BF4-]
F
1h
13C NMR (101 MHz, CDCl3)
200 190 180 170 160 150 140 130 120 110 100 90 80 70 60 50 40 30 20 10 0
f1 (ppm)

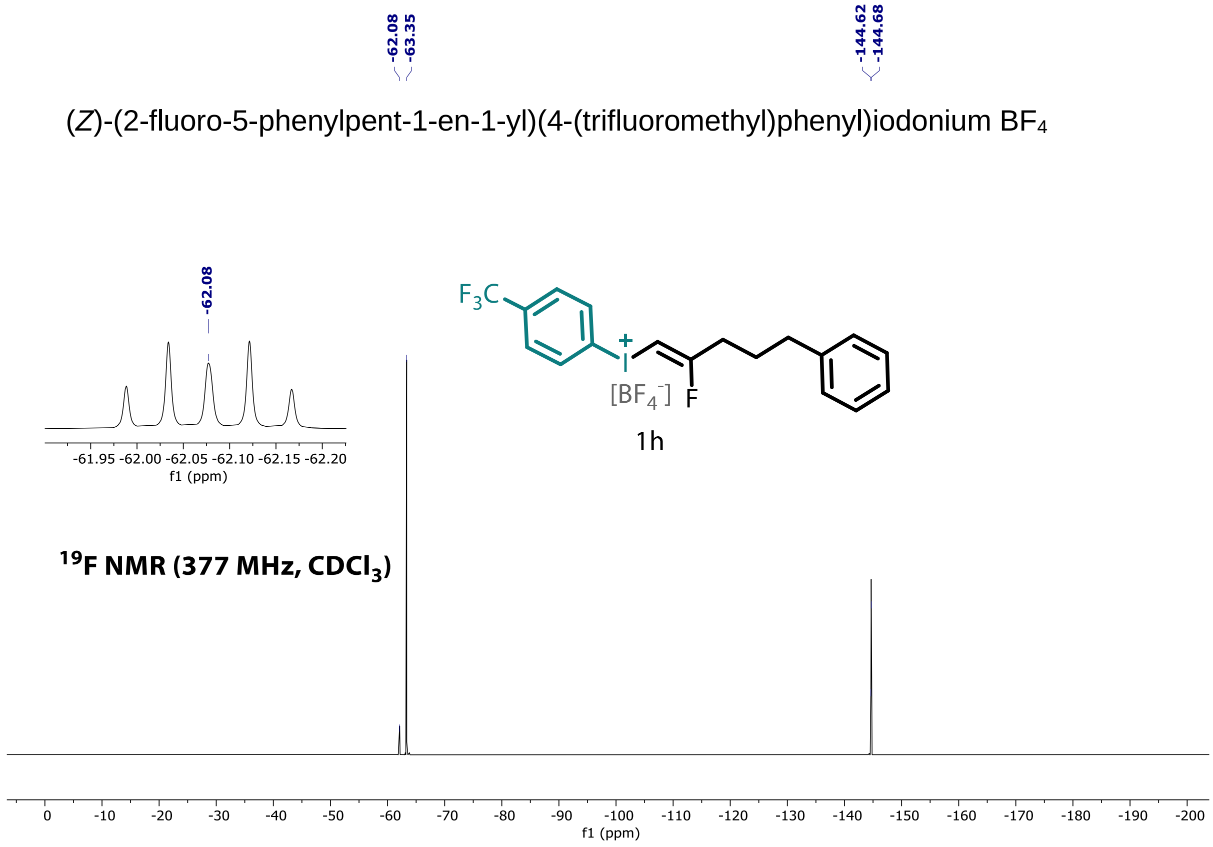
-62.08
-63.35
-144.62
-144.68
(Z)-(2-fluoro-5-phenylpent-1-en-1-yl)(4-(trifluoromethyl)phenyl)iodonium BF4
-62.08
-61.95 -62.00 -62.05 -62.10 -62.15 -62.20
f1 (ppm)
F3C
[BF4-]
F
1h
19F NMR (377 MHz, CDCl3)
0 -10 -20 -30 -40 -50 -60 -70 -80 -90 -100 -110 -120 -130 -140 -150 -160 -170 -180 -190 -200
f1 (ppm)

(*Z*)-(2-cyclopropyl-2-fluorovinyl)(mesityl)iodonium BF_4

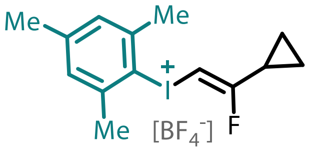

1k

^{1}H NMR (400 MHz, $CDCl_3$)

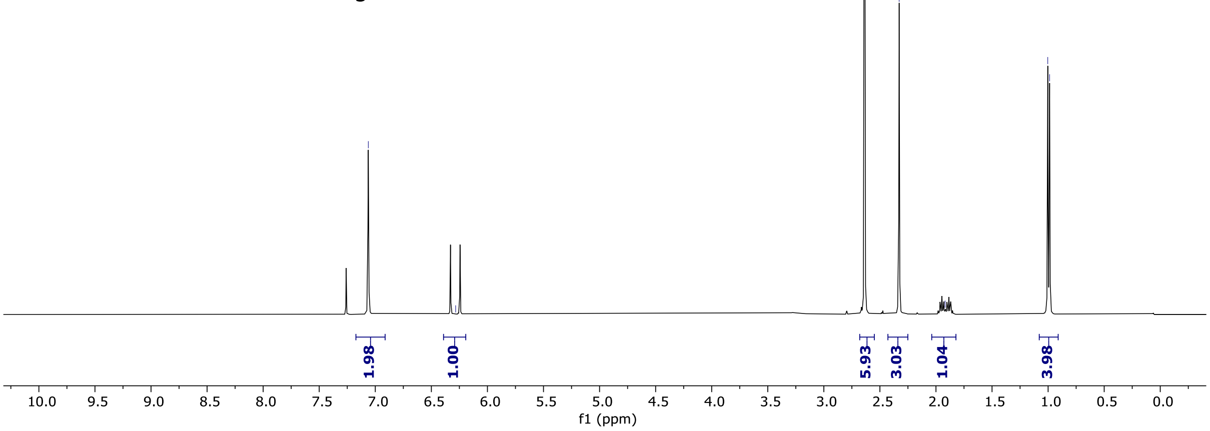

(*Z*)-(2-cyclopropyl-2-fluorovinyl)(mesityl)iodonium BF_4

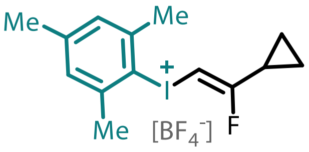

1k

^{13}C NMR (101 MHz, $CDCl_3$)

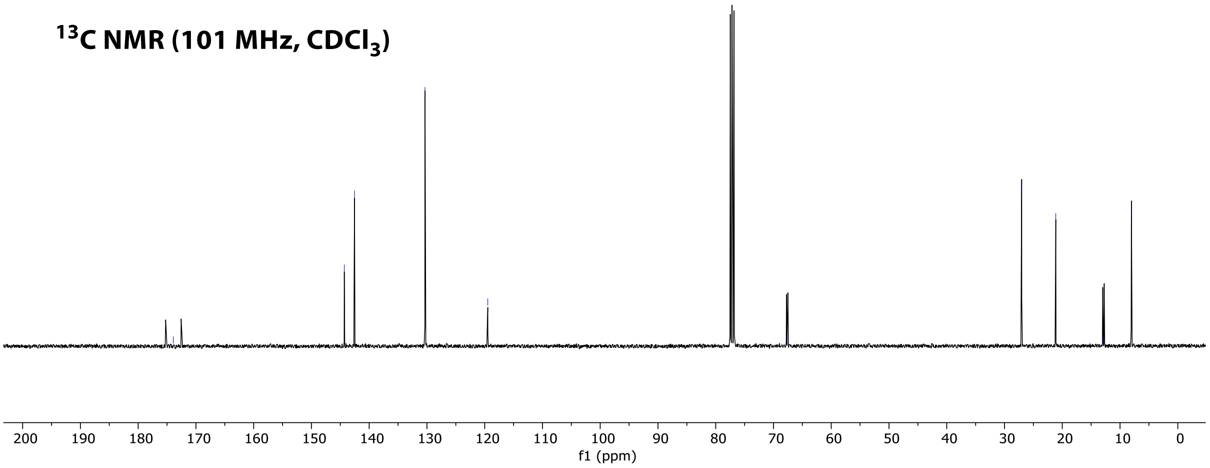

(*Z*)-(2-cyclopropyl-2-fluorovinyl)(mesityl)iodonium BF_4

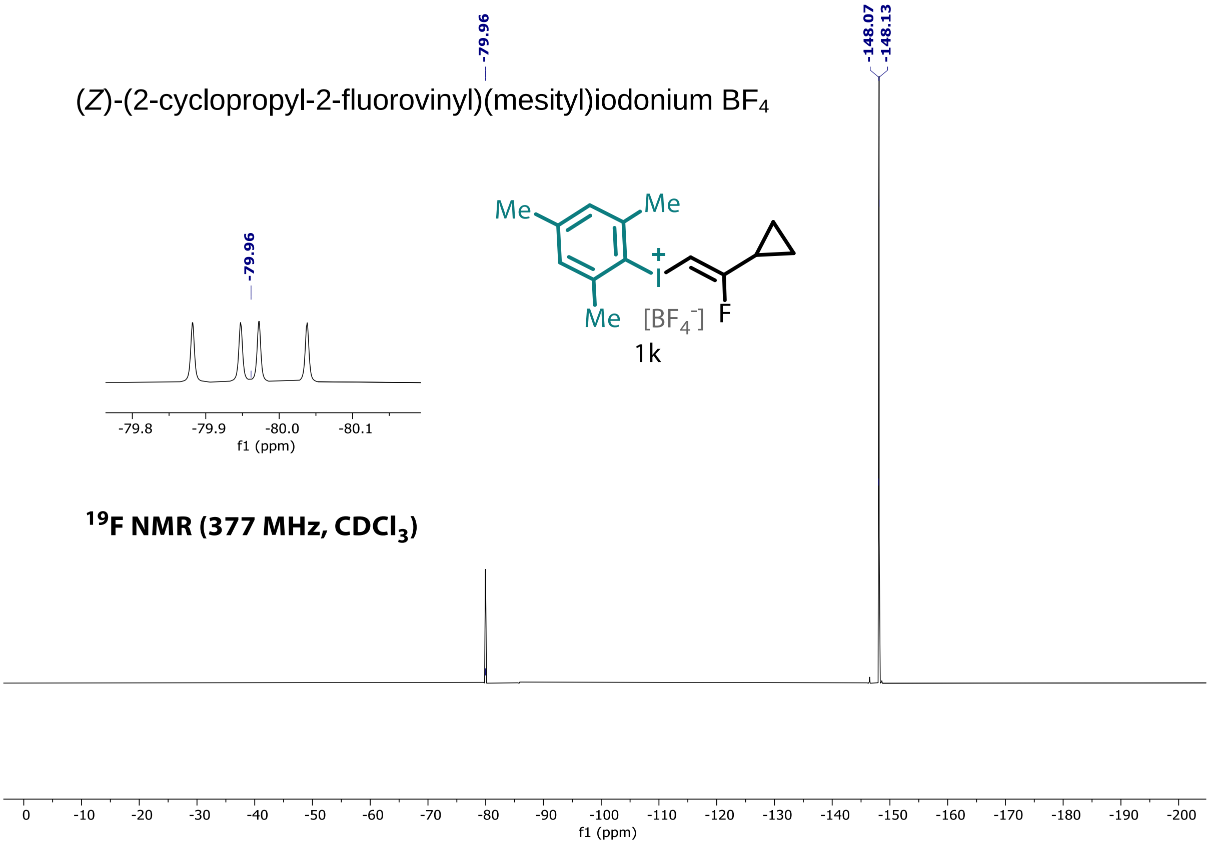

^{19}F NMR (377 MHz, $CDCl_3$)

((*Z*)-2-fluoro-3-(((1*S*,2*R*,5*S*)-2-isopropyl-5-methylcyclohexyl)oxy)prop-1-en-1-yl)(mesityl)iodonium BF_4

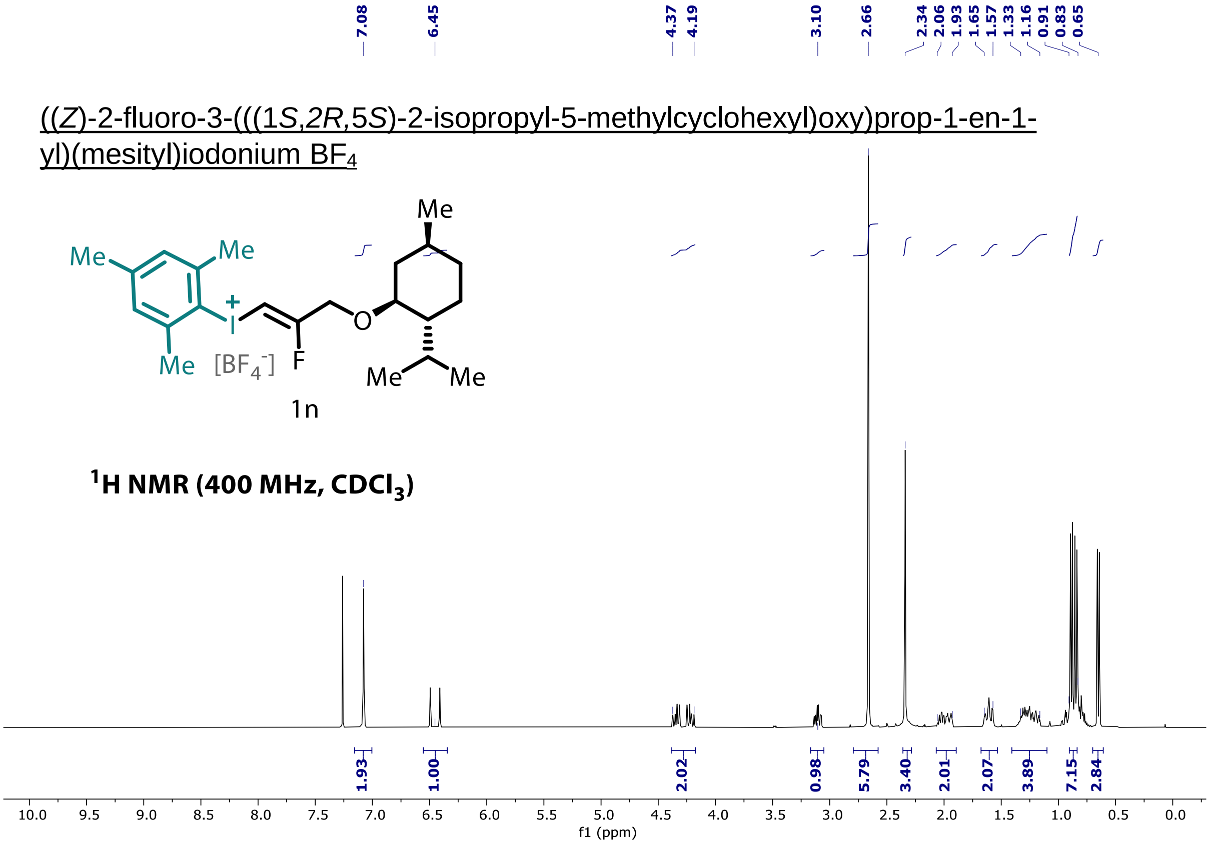

^{1}H NMR (400 MHz, $CDCl_3$)

((*Z*)-2-fluoro-3-(((1*S*,2*R*,5*S*)-2-isopropyl-5-methylcyclohexyl)oxy)prop-1-en-1-yl)(mesityl)iodonium BF_4

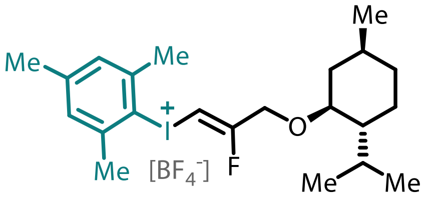

1n

^{13}C NMR (101 MHz, CDCl$_3$)

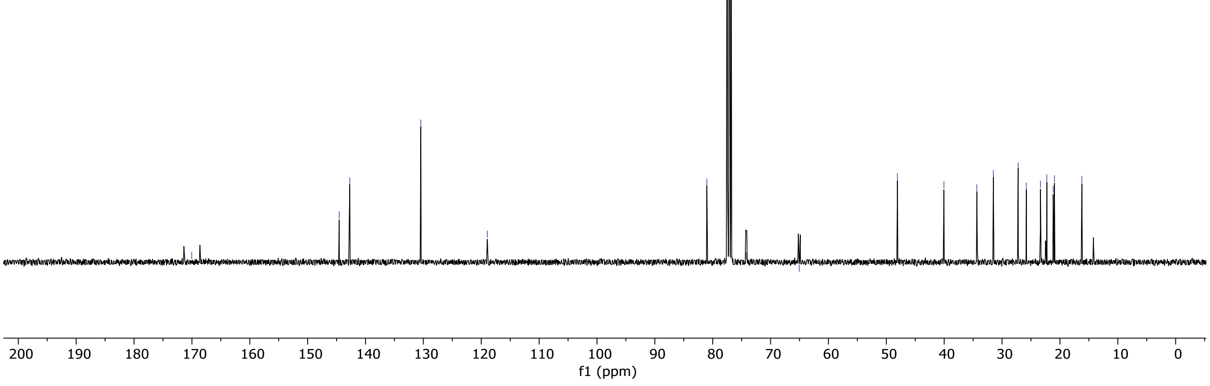

((*Z*)-2-fluoro-3-(((1*S*,2*R*,5*S*)-2-isopropyl-5-methylcyclohexyl)oxy)prop-1-en-1-yl)(mesityl)iodonium BF_4

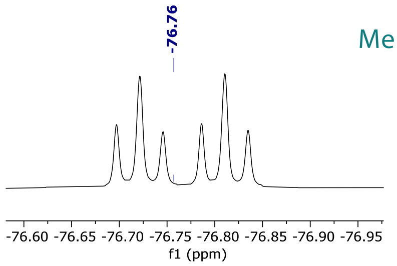

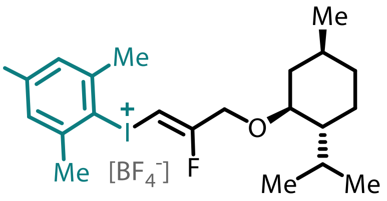

1n

^{19}F NMR (377 MHz, CDCl$_3$)

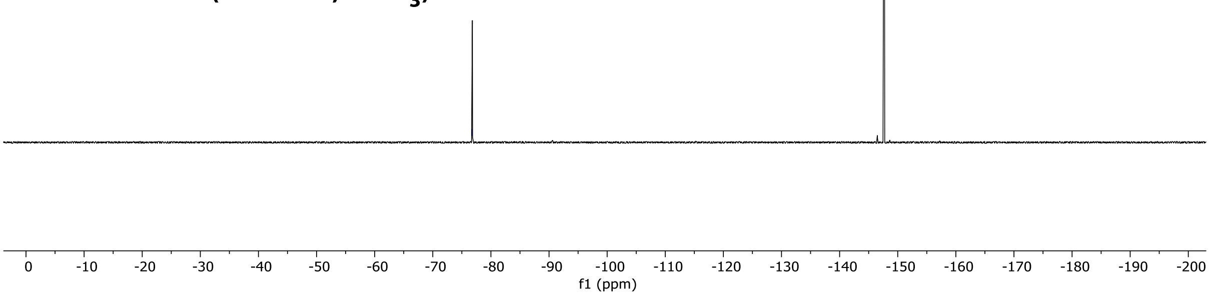

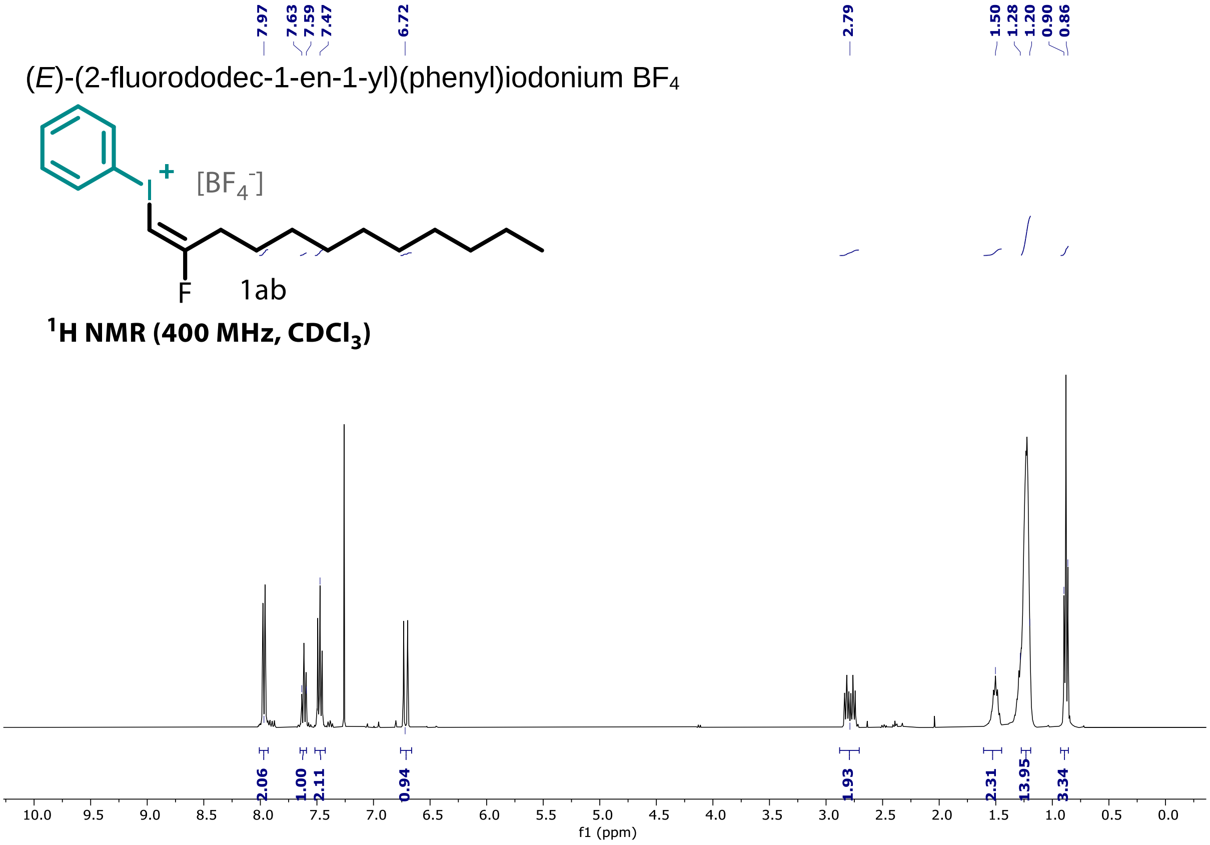
(*E*)-(2-fluorododec-1-en-1-yl)(phenyl)iodonium BF_4
[BF_4^-]
1ab
^{1}H NMR (400 MHz, $CDCl_3$)
7.97 7.63 7.59 7.47 6.72 2.79 1.50 1.28 1.20 0.90 0.86
2.06 1.00 2.11 0.94 1.93 2.31 13.95 3.34
f1 (ppm)

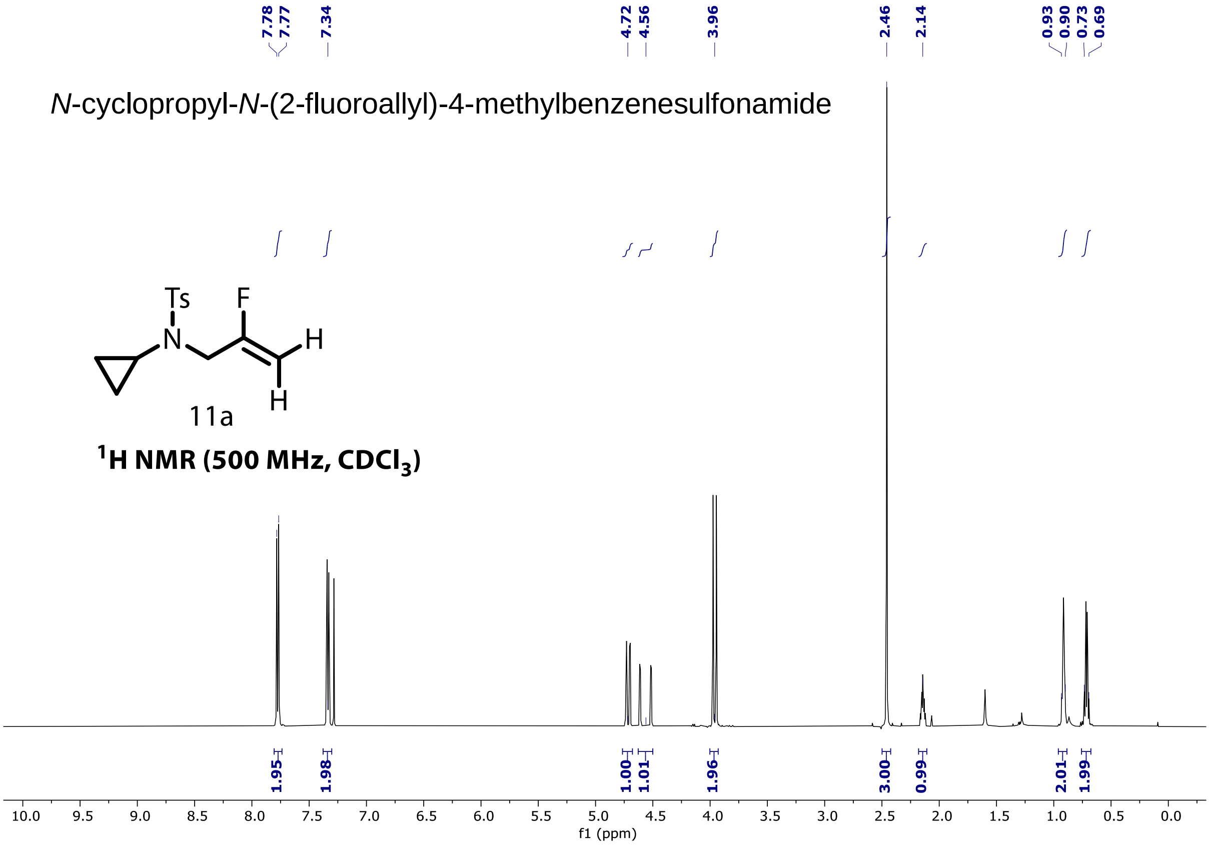
N-cyclopropyl-*N*-(2-fluoroallyl)-4-methylbenzenesulfonamide
Ts
F
N
H
H
11a
^{1}H NMR (500 MHz, $CDCl_3$)
7.78 7.77 7.34 4.72 4.56 3.96 2.46 2.14 0.93 0.90 0.73 0.69
1.95 1.98 1.00 1.01 1.96 3.00 0.99 2.01 1.99
f1 (ppm)

N-cyclopropyl-*N*-(2-fluoroallyl)-4-methylbenzenesulfonamide

^{13}C NMR (125 MHz, CDCl$_3$)

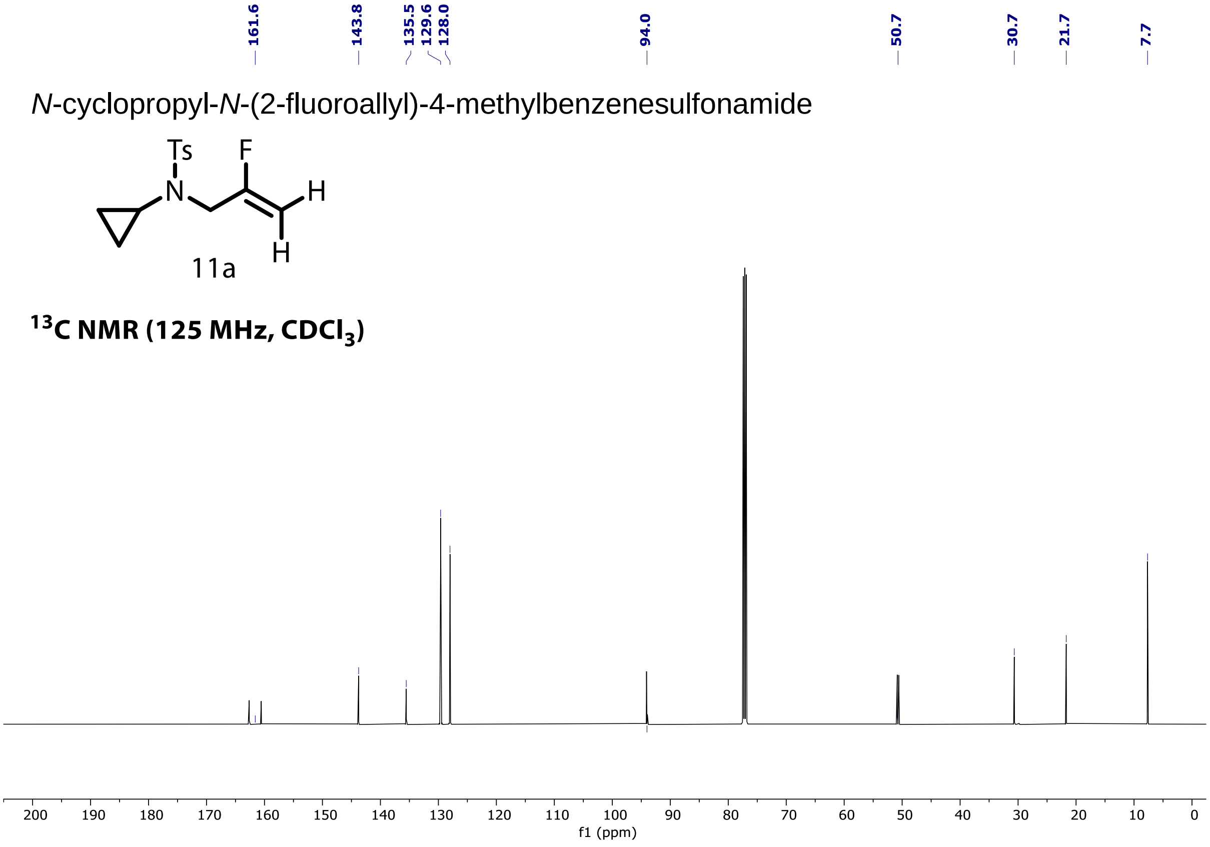

N-cyclopropyl-*N*-(2-fluoroallyl)-4-methylbenzenesulfonamide

^{19}F NMR (377 MHz, CDCl$_3$)

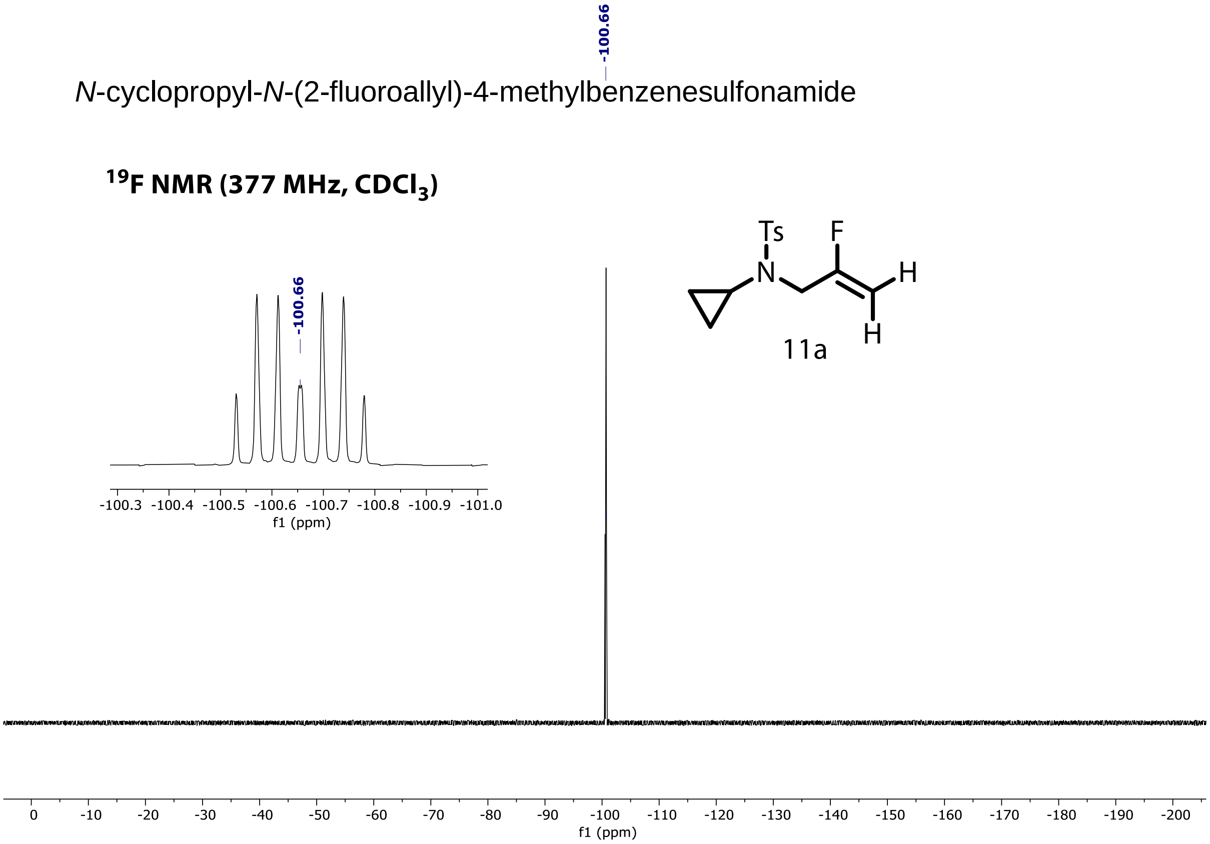

(*Z*)-2-(2-fluoro-5-phenylpent-1-en-1-yl)-1-methyl-1*H*-indole

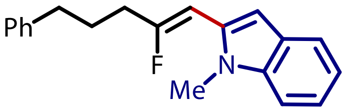

3a

^{1}H NMR (400 MHz, CDCl$_3$)

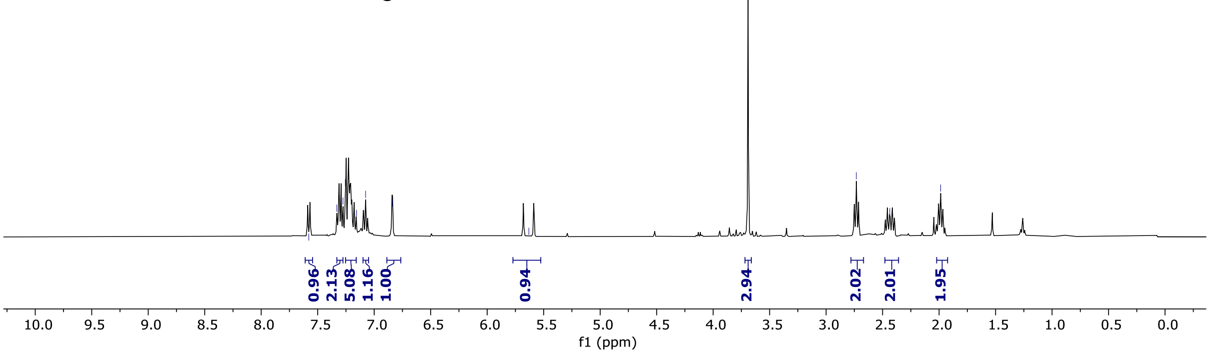

(*Z*)-2-(2-fluoro-5-phenylpent-1-en-1-yl)-1-methyl-1*H*-indole

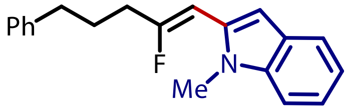

3a

^{13}C NMR (101 MHz, CDCl$_3$)

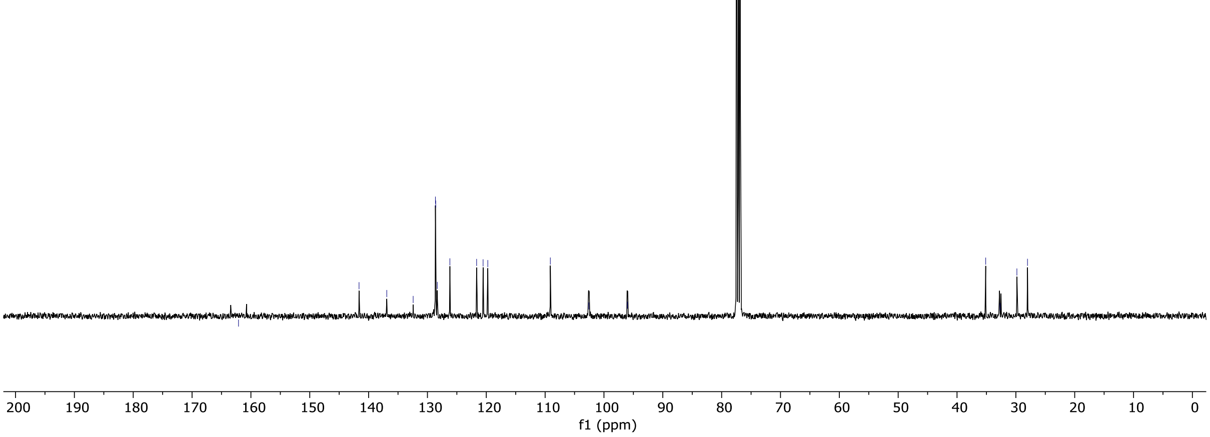

(*Z*)-2-(2-fluoro-5-phenylpent-1-en-1-yl)-1-methyl-1*H*-indole

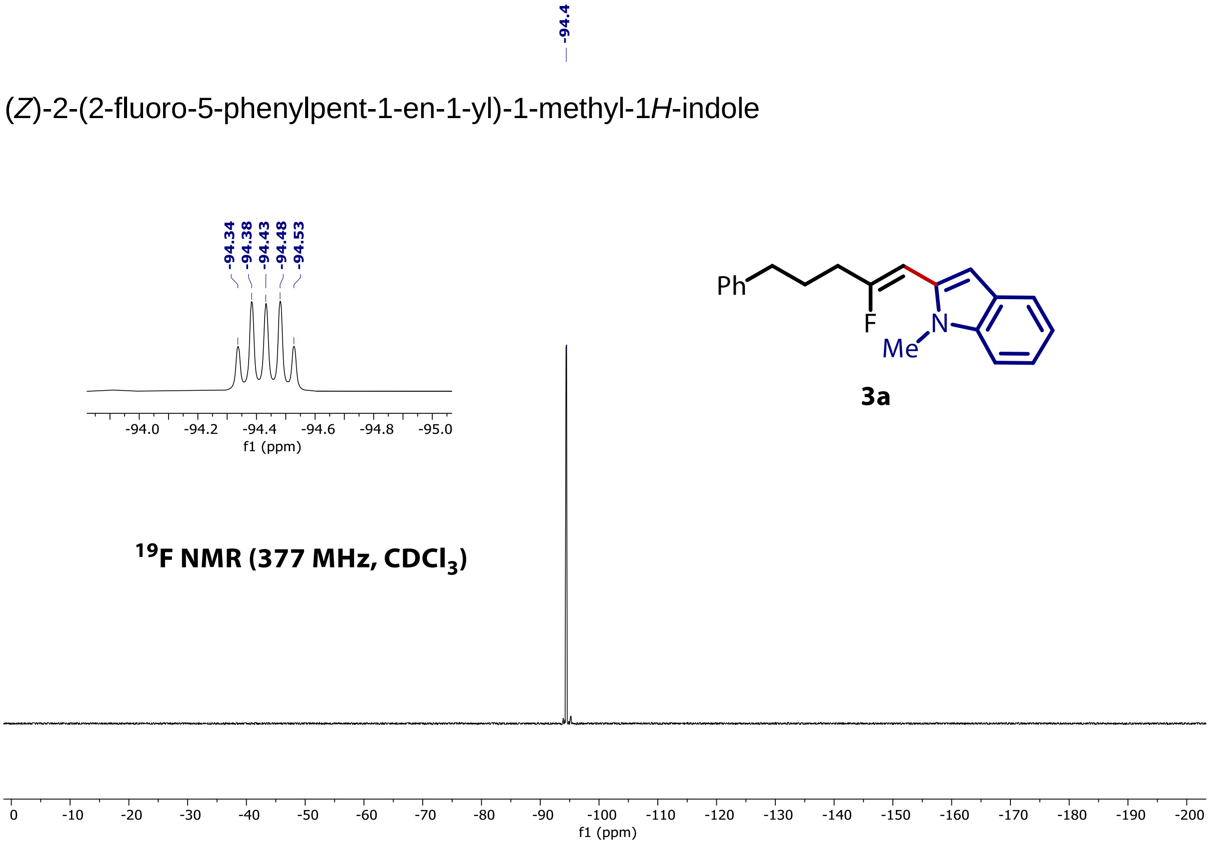

(*Z*)-1-benzyl-2-(2-fluoro-5-phenylpent-1-en-1-yl)-1*H*-indole

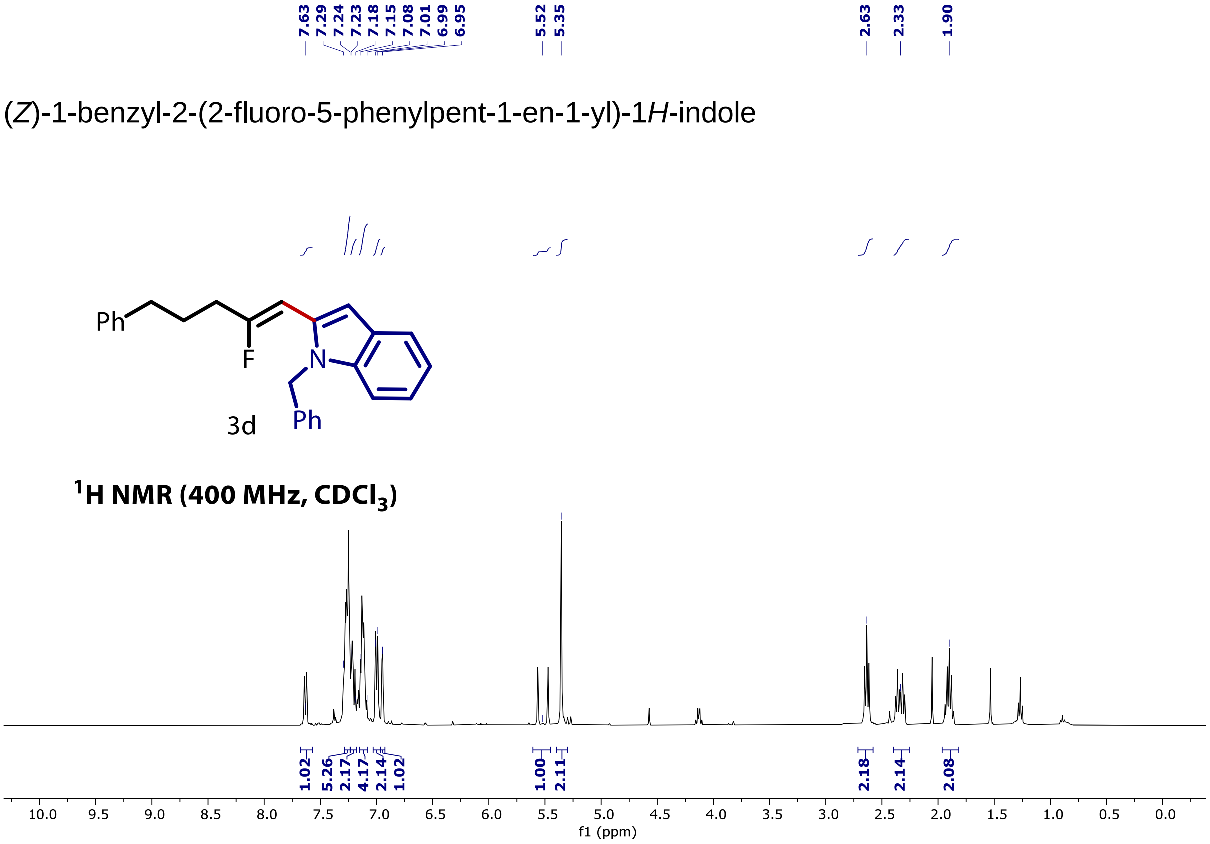

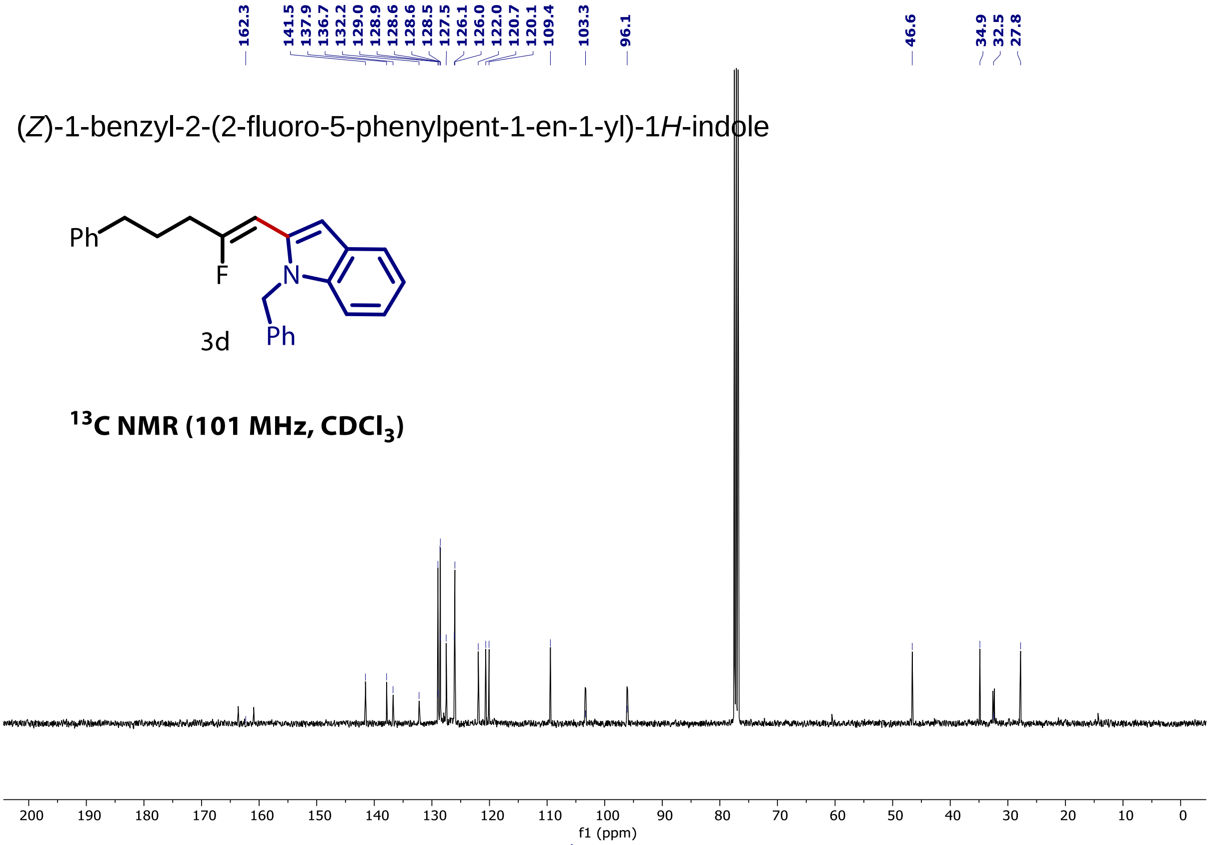
162.3
141.5
137.9
136.7
132.2
129.0
128.9
128.6
128.6
128.5
127.5
126.1
126.0
122.0
120.7
120.1
109.4
103.3
96.1
46.6
34.9
32.5
27.8
(Z)-1-benzyl-2-(2-fluoro-5-phenylpent-1-en-1-yl)-1H-indole
Ph
F
N
3d
Ph
13C NMR (101 MHz, CDCl3)
200 190 180 170 160 150 140 130 120 110 100 90 80 70 60 50 40 30 20 10 0
f1 (ppm)

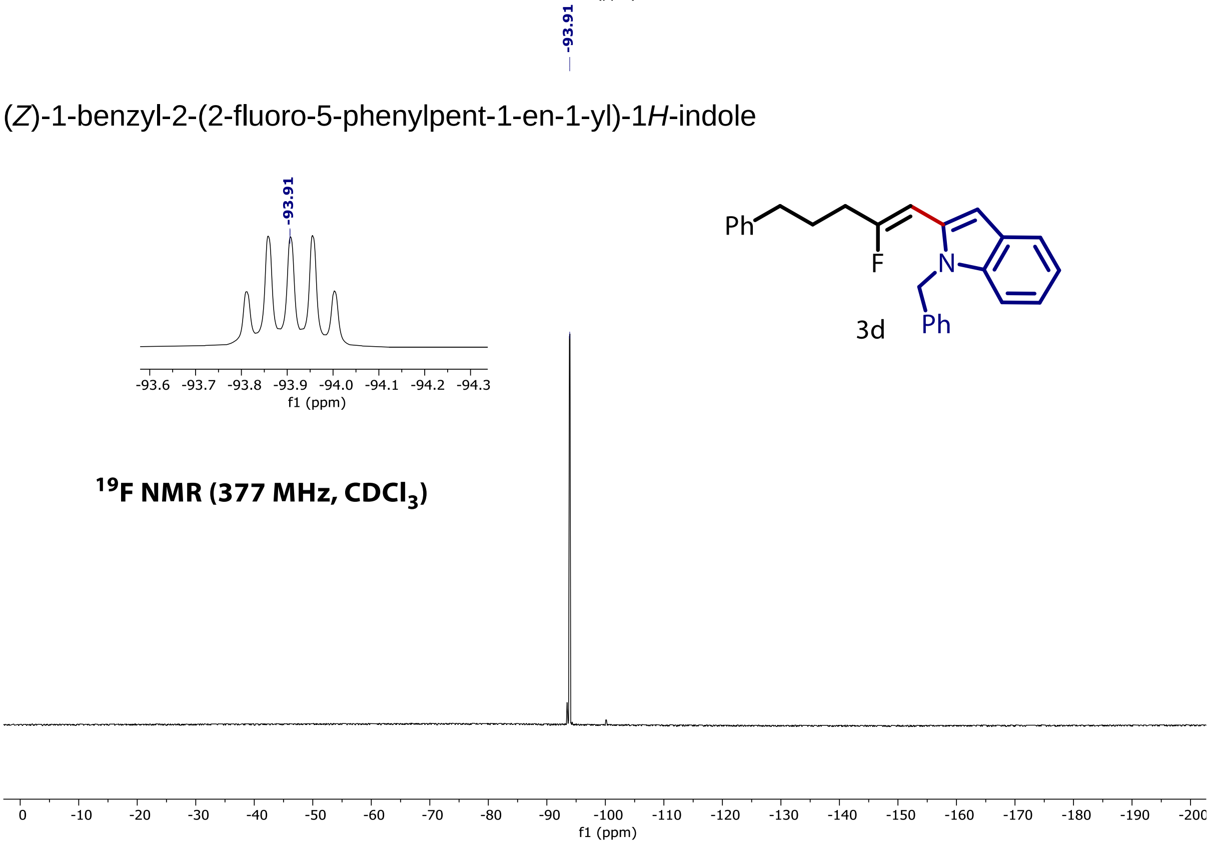
-93.91
(Z)-1-benzyl-2-(2-fluoro-5-phenylpent-1-en-1-yl)-1H-indole
-93.91
-93.6 -93.7 -93.8 -93.9 -94.0 -94.1 -94.2 -94.3
f1 (ppm)
Ph
F
N
3d
Ph
19F NMR (377 MHz, CDCl3)
0 -10 -20 -30 -40 -50 -60 -70 -80 -90 -100 -110 -120 -130 -140 -150 -160 -170 -180 -190 -200
f1 (ppm)

(*Z*)-2-(2-fluoro-5-phenylpent-1-en-1-yl)-1*H*-indole

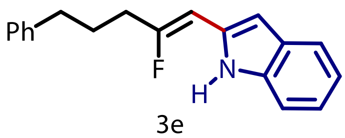

3e

^{1}H NMR (400 MHz, $CDCl_3$)

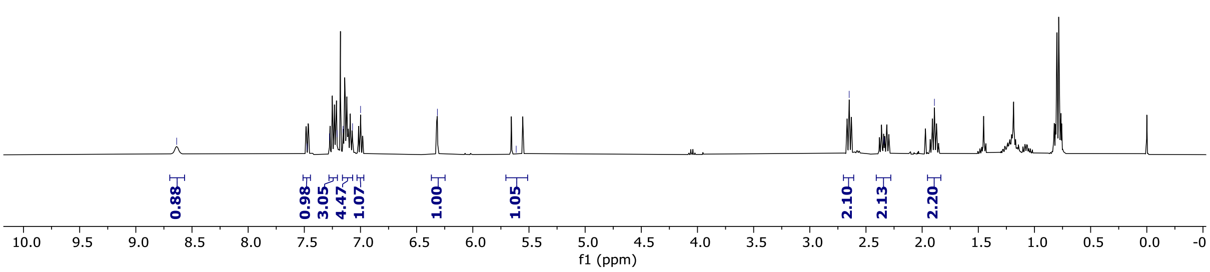

(*Z*)-2-(2-fluoro-5-phenylpent-1-en-1-yl)-1*H*-indole

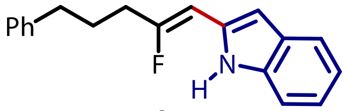

3e

^{13}C NMR (101 MHz, $CDCl_3$)

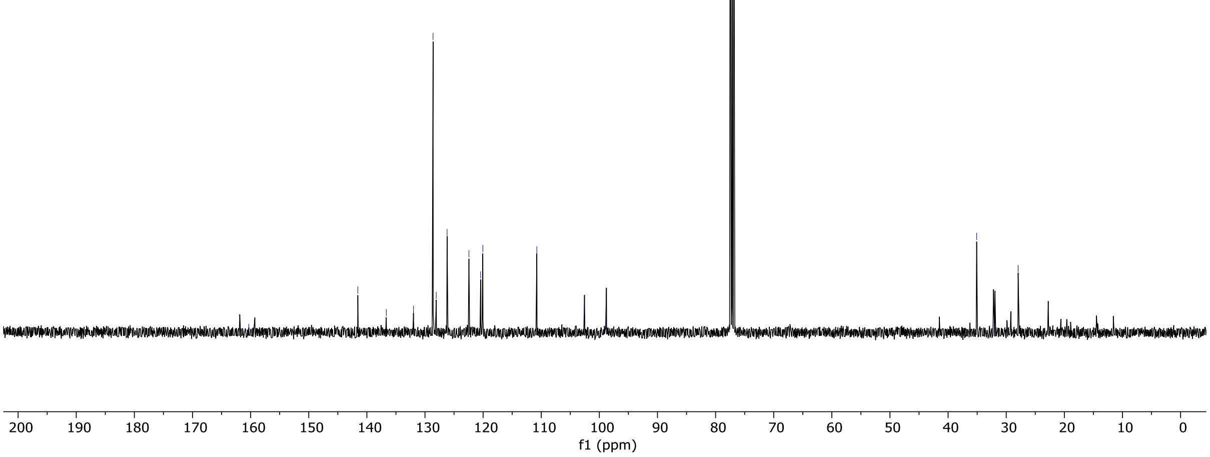

(*Z*)-2-(2-fluoro-5-phenylpent-1-en-1-yl)-1*H*-indole

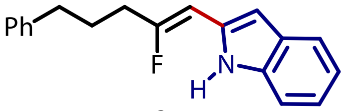

3e

^{19}F NMR (377 MHz, $CDCl_3$)

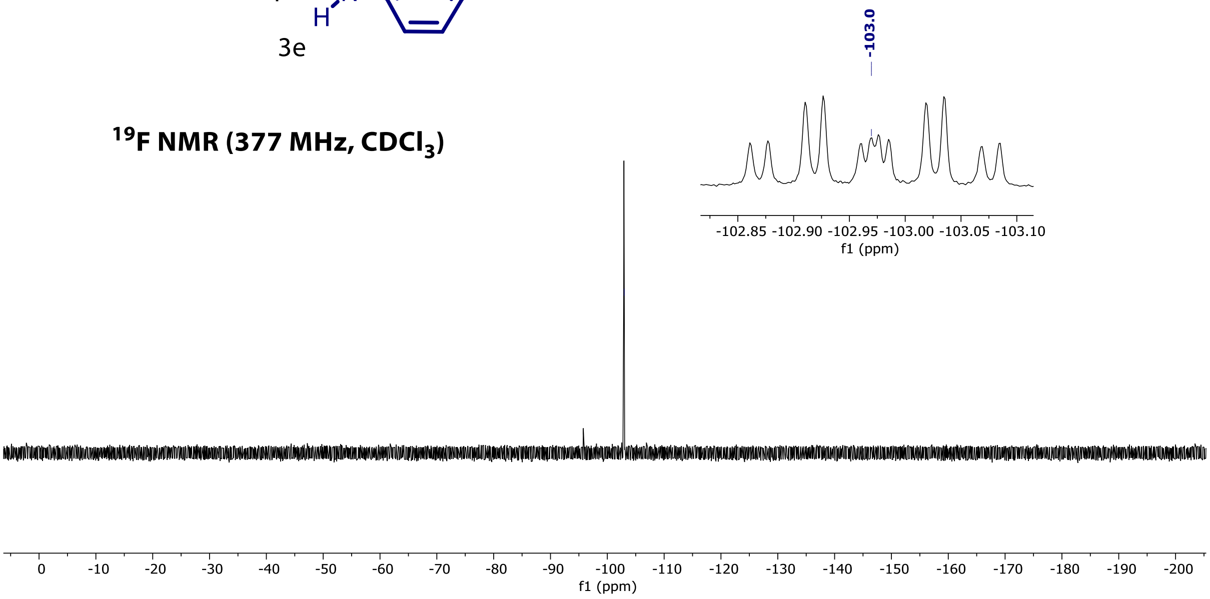

(*Z*)-2-(2-fluoro-5-phenylpent-1-en-1-yl)-5-(p-tolyl)-1*H*-indole

^{1}H NMR (400 MHz, $CDCl_3$)

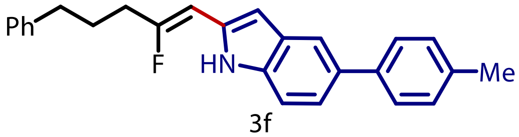

3f

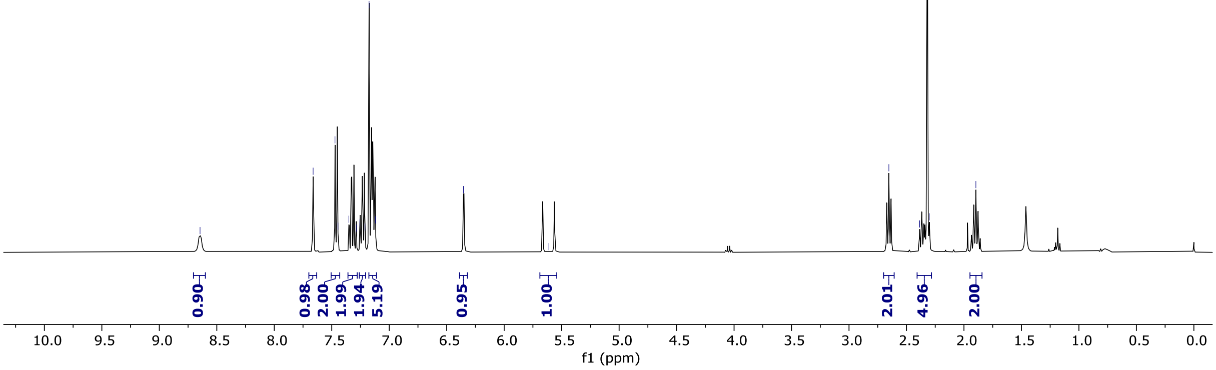

(*Z*)-2-(2-fluoro-5-phenylpent-1-en-1-yl)-5-(p-tolyl)-1*H*-indole

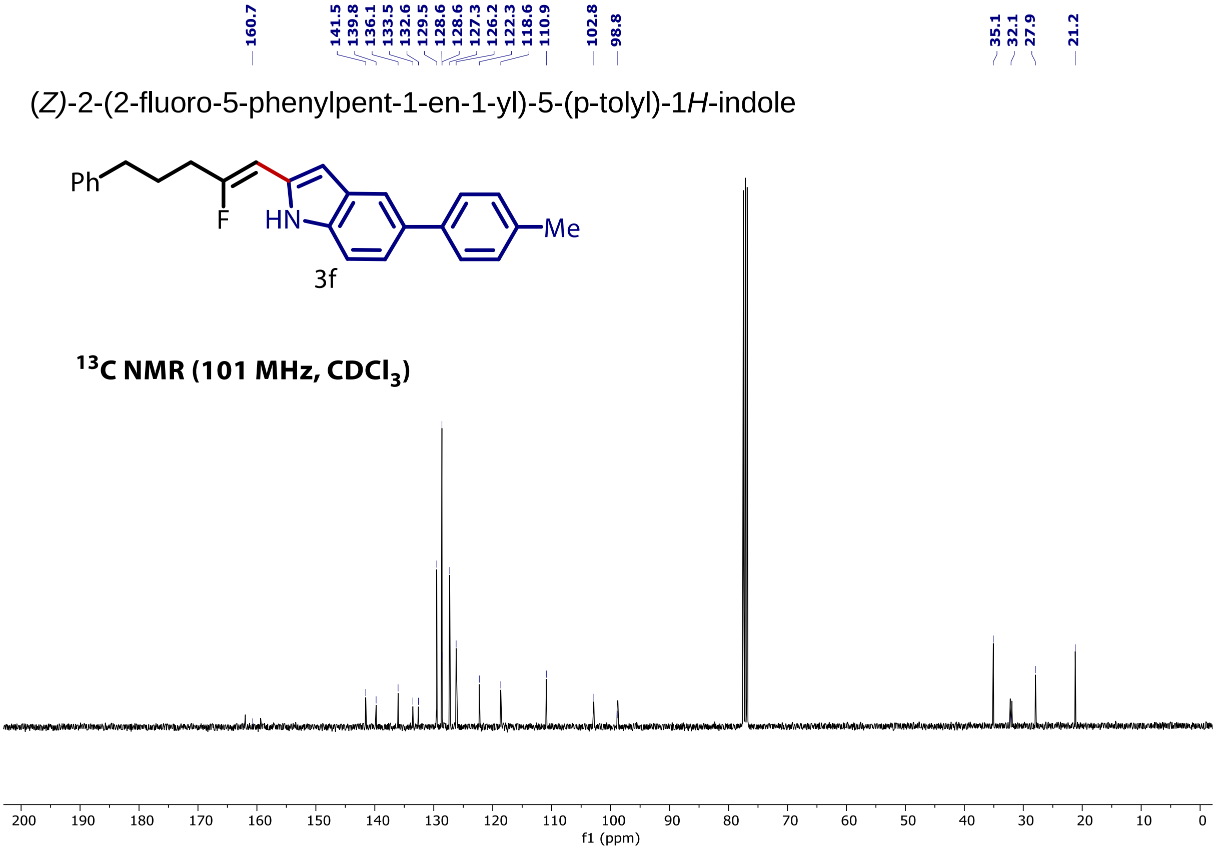

(*Z*)-2-(2-fluoro-5-phenylpent-1-en-1-yl)-5-(p-tolyl)-1*H*-indole

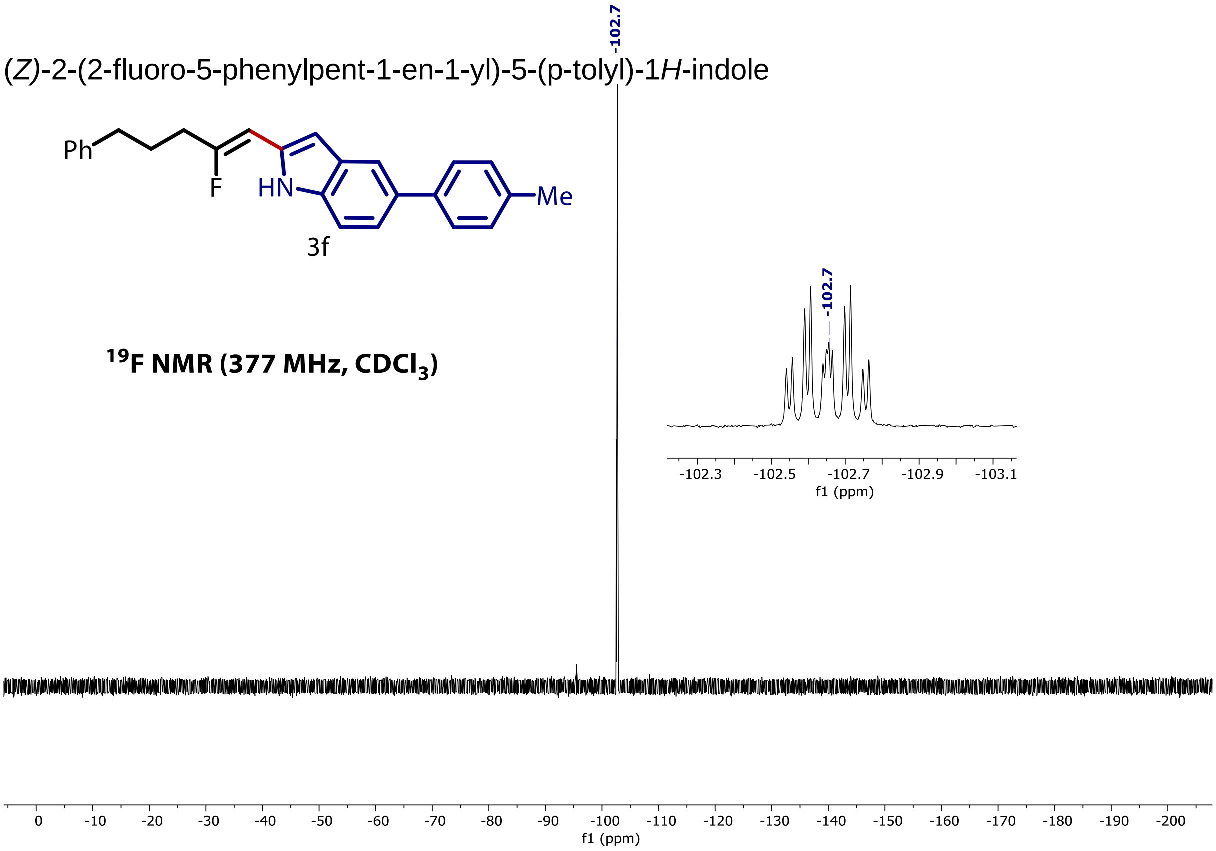

(Z)-2-(2-fluoro-5-phenylpent-1-en-1-yl)-5-methyl-1*H*-indole

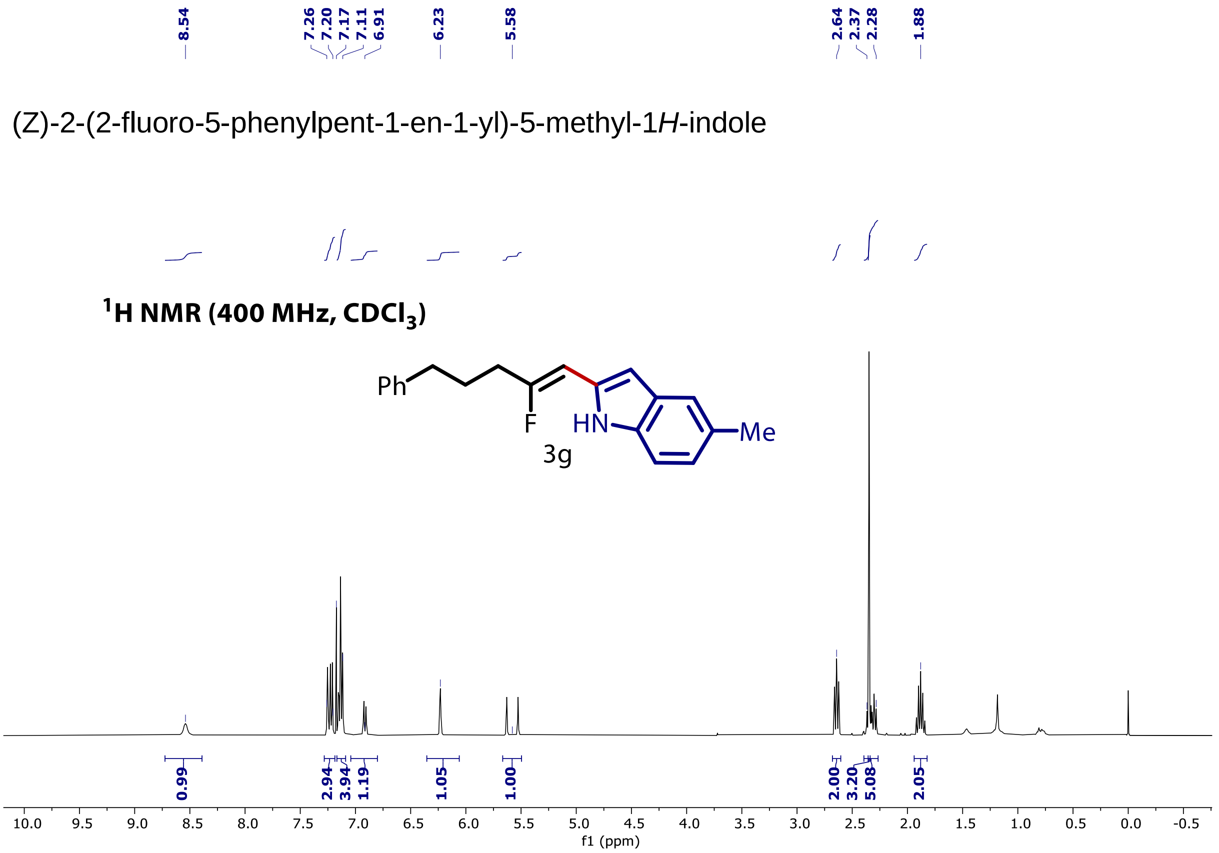

(Z)-2-(2-fluoro-5-phenylpent-1-en-1-yl)-5-methyl-1*H*-indole

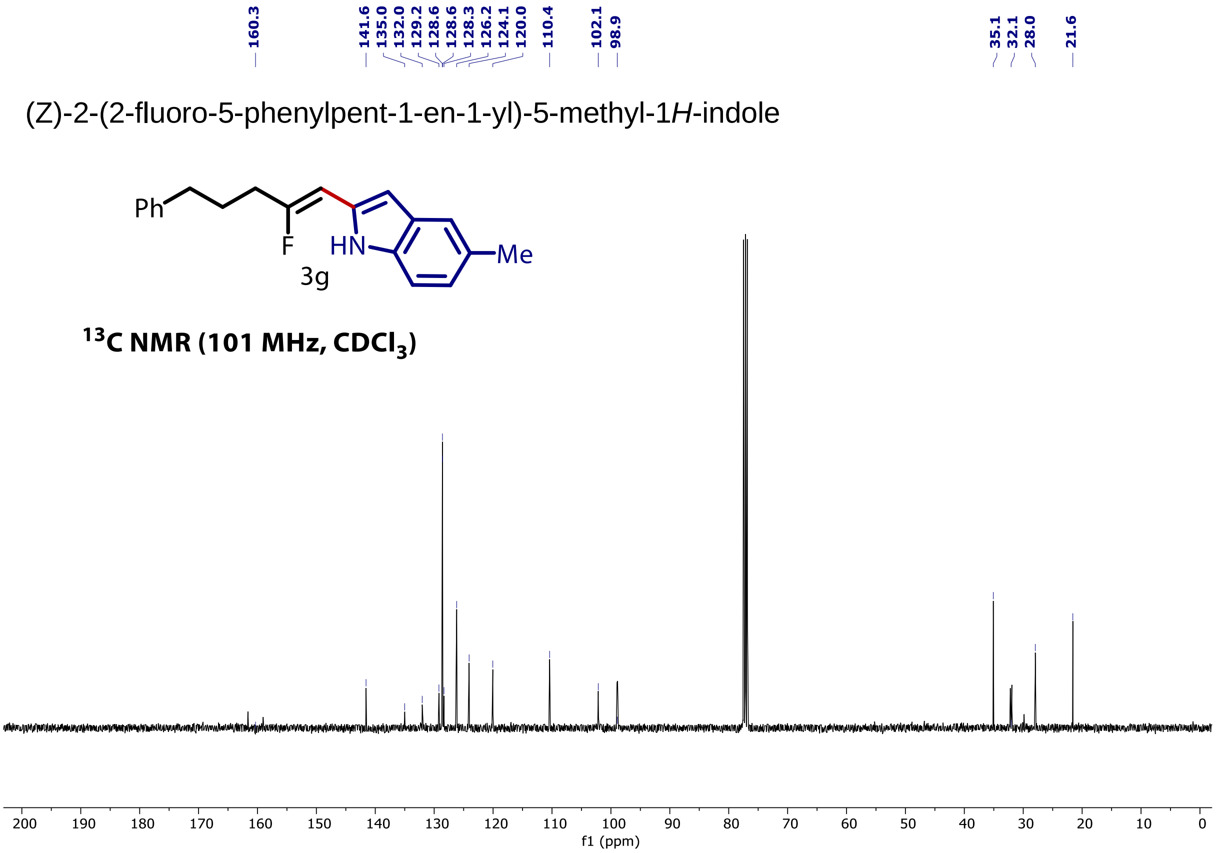

(Z)-2-(2-fluoro-5-phenylpent-1-en-1-yl)-5-methyl-1*H*-indole

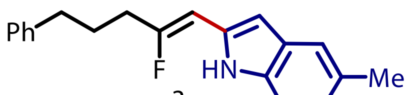

3g

^{19}F NMR (377 MHz, $CDCl_3$)

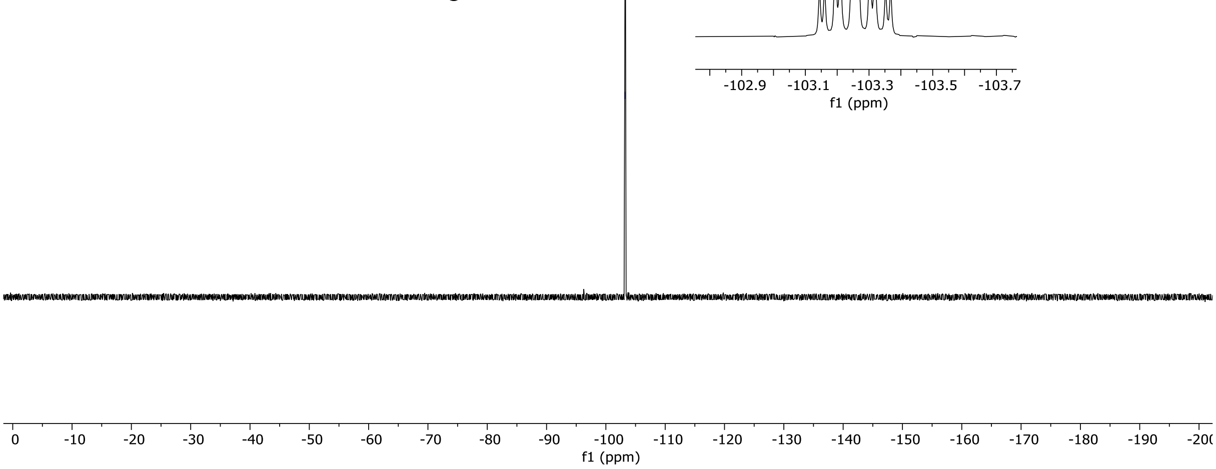

(*Z*)-2-(2-fluoro-5-phenylpent-1-en-1-yl)-5-methoxy-1*H*-indole

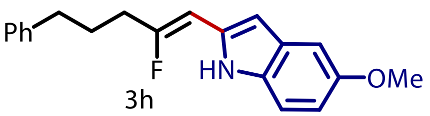

3h

^{1}H NMR (400 MHz, $CDCl_3$)

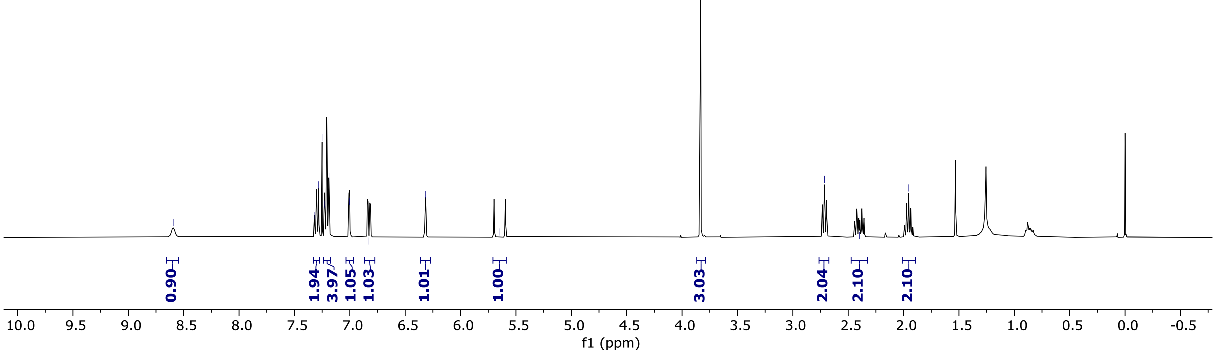

(*Z*)-2-(2-fluoro-5-phenylpent-1-en-1-yl)-5-methoxy-1*H*-indole

3h

^{13}C NMR (101 MHz, $CDCl_3$)

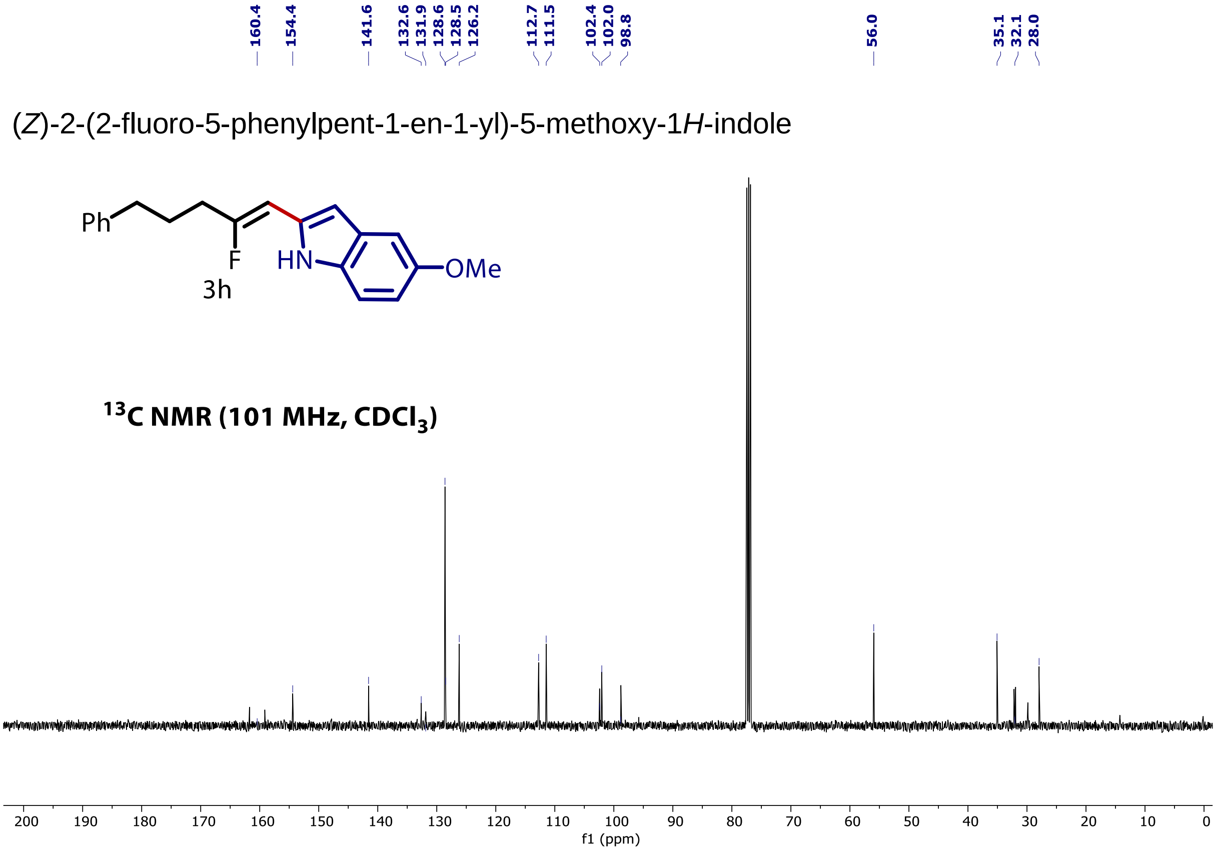

(*Z*)-2-(2-fluoro-5-phenylpent-1-en-1-yl)-5-methoxy-1*H*-indole

3h

^{19}F NMR (377 MHz, $CDCl_3$)

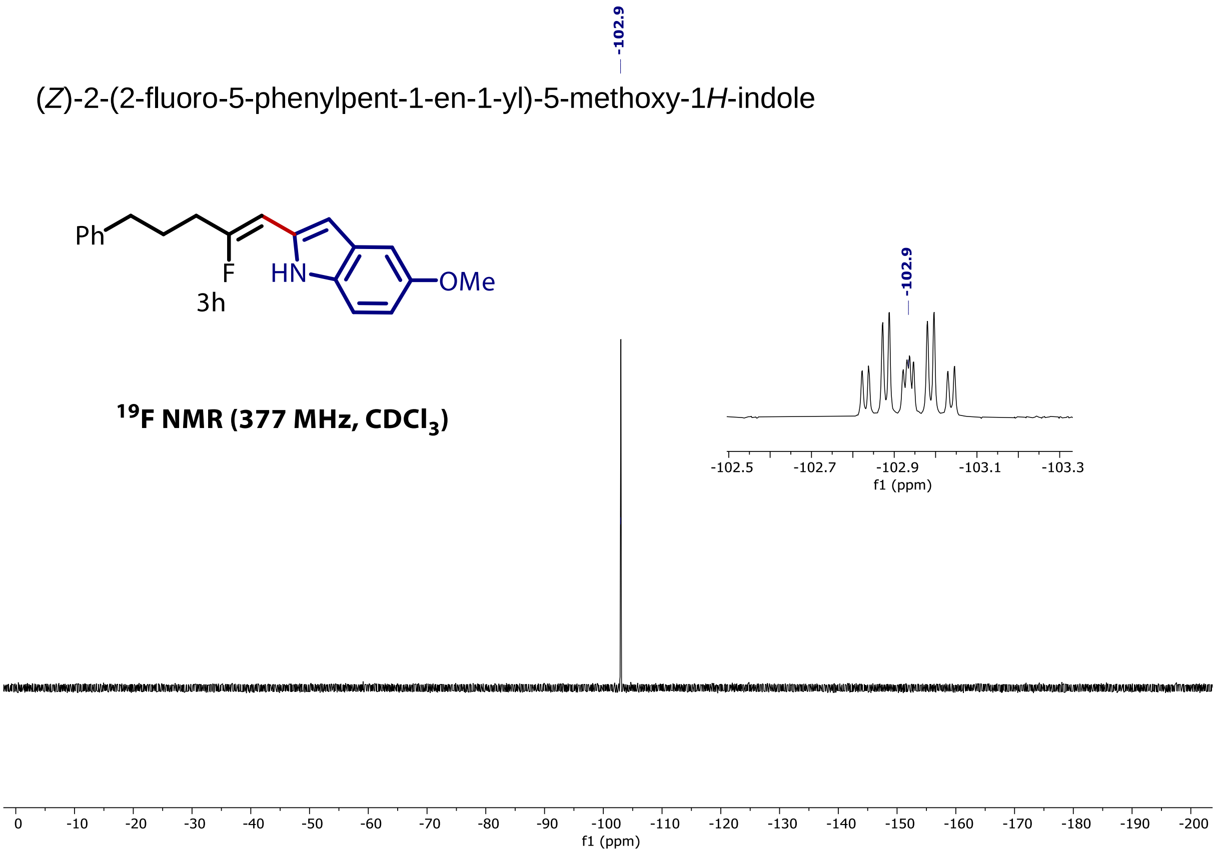

(*Z*)-4-(benzyloxy)-2-(2-fluoro-5-phenylpent-1-en-1-yl)-1*H*-indole

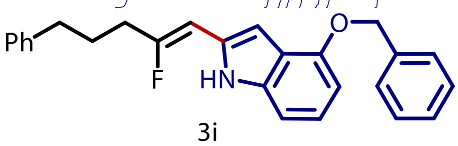

3i

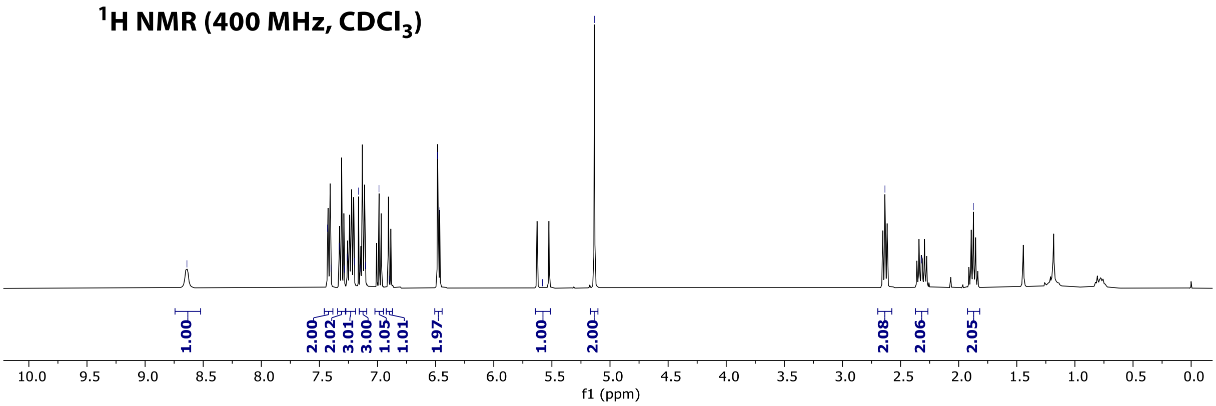

(*Z*)-4-(benzyloxy)-2-(2-fluoro-5-phenylpent-1-en-1-yl)-1*H*-indole

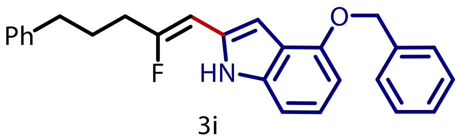

3i

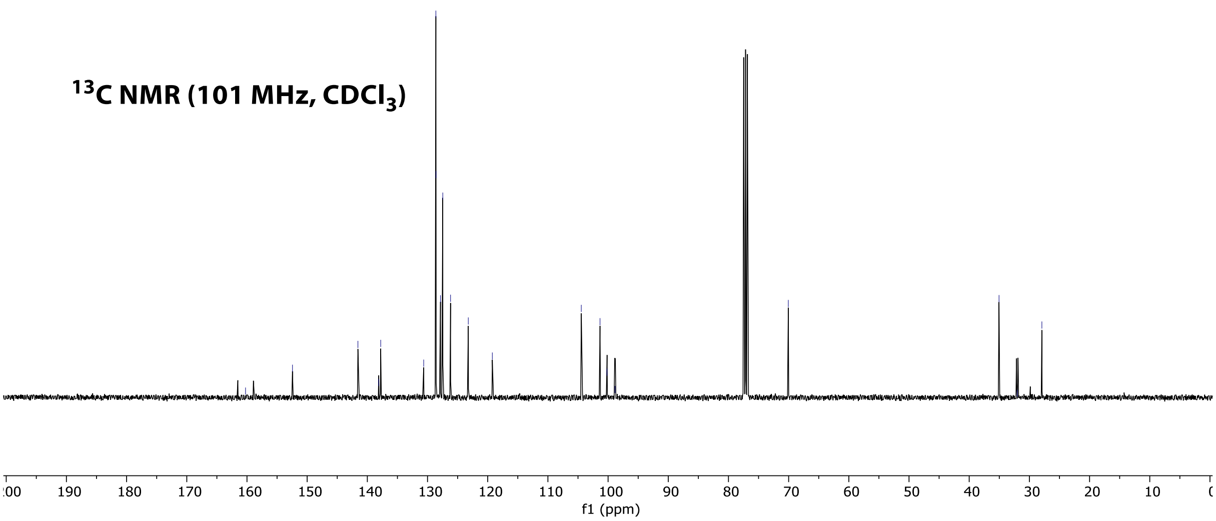

(*Z*)-4-(benzyloxy)-2-(2-fluoro-5-phenylpent-1-en-1-yl)-1*H*-indole

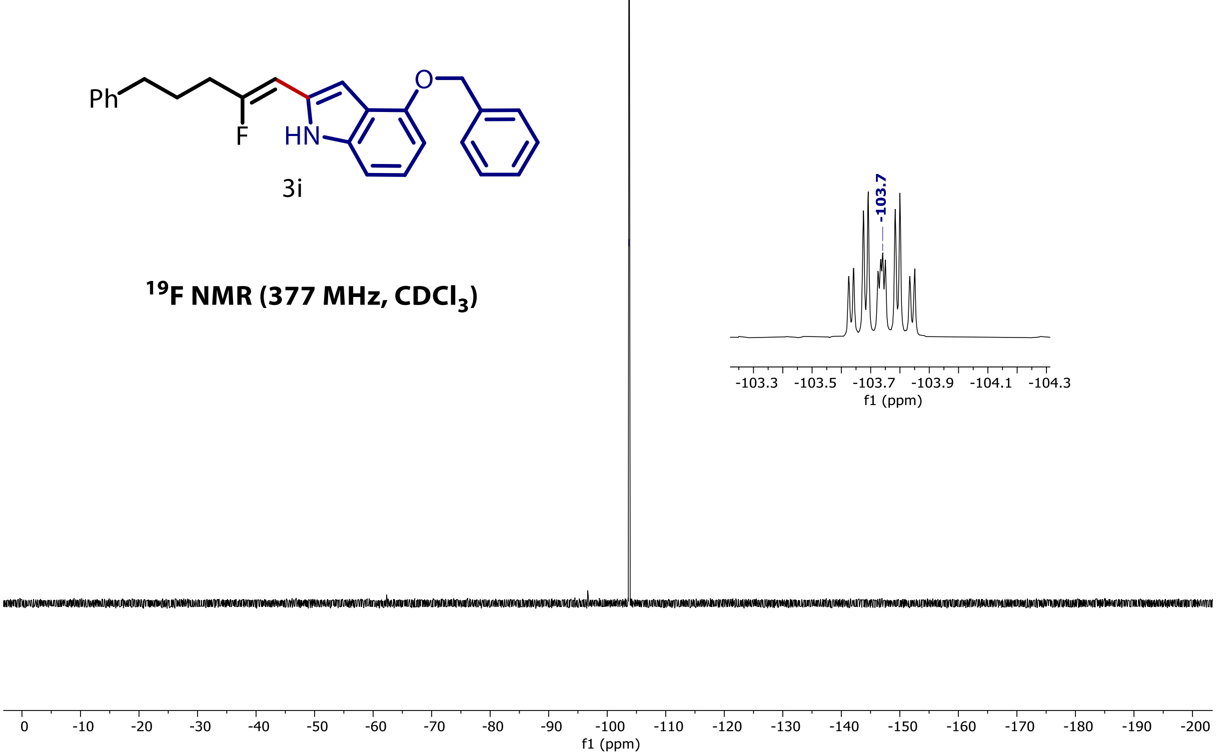

(*Z*)-2-(2-fluoro-5-phenylpent-1-en-1-yl)-7-methoxy-1*H*-indole

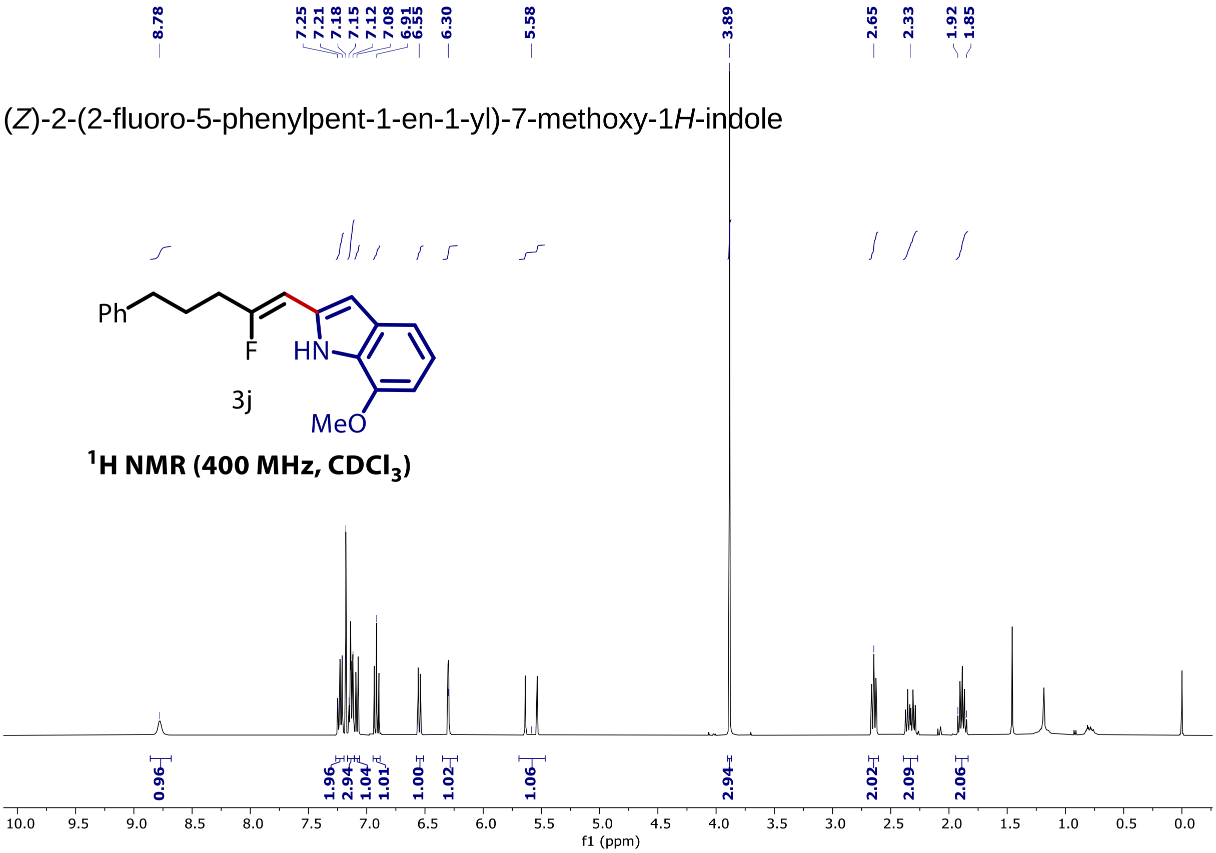

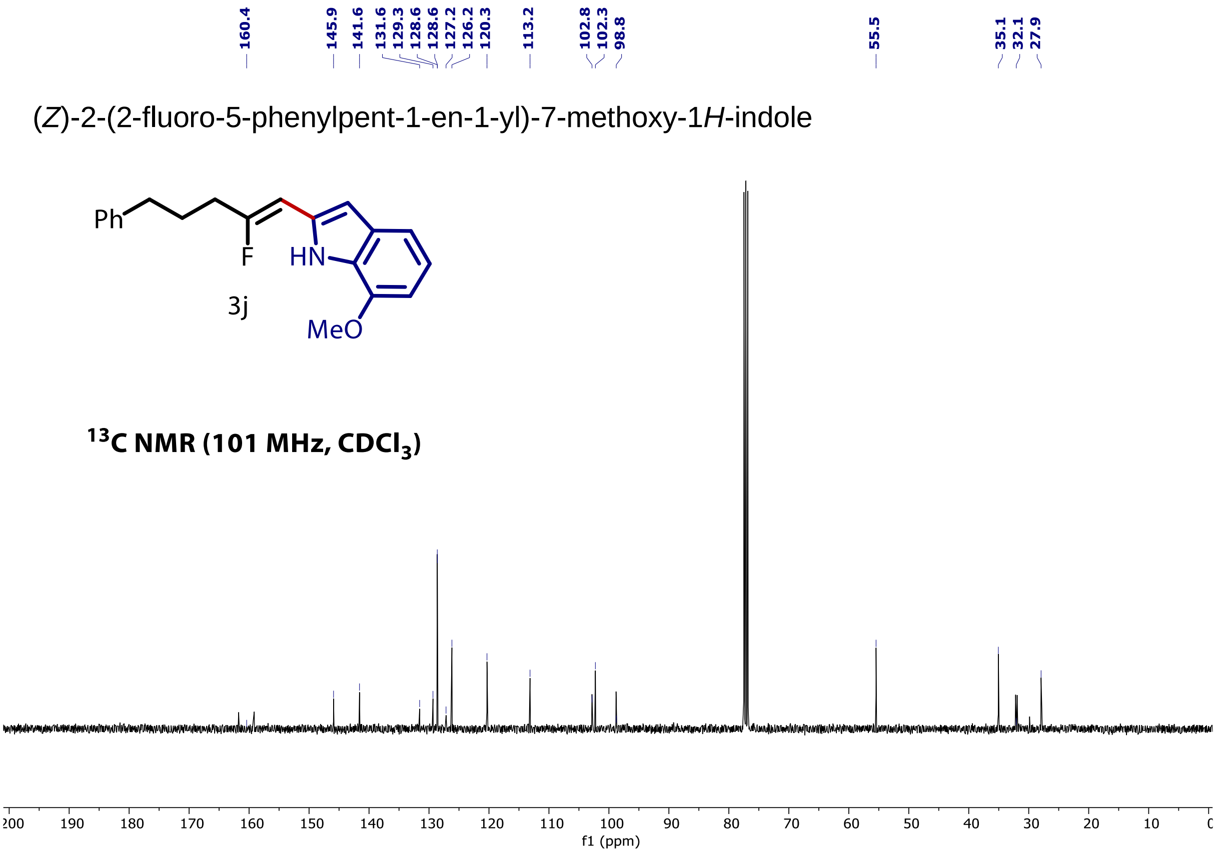
160.4
145.9
141.6
131.6
129.3
128.6
128.6
127.2
126.2
120.3
113.2
102.8
102.3
98.8
55.5
35.1
32.1
27.9
(Z)-2-(2-fluoro-5-phenylpent-1-en-1-yl)-7-methoxy-1H-indole
Ph
F
HN
3j
MeO
13C NMR (101 MHz, CDCl3)
f1 (ppm)

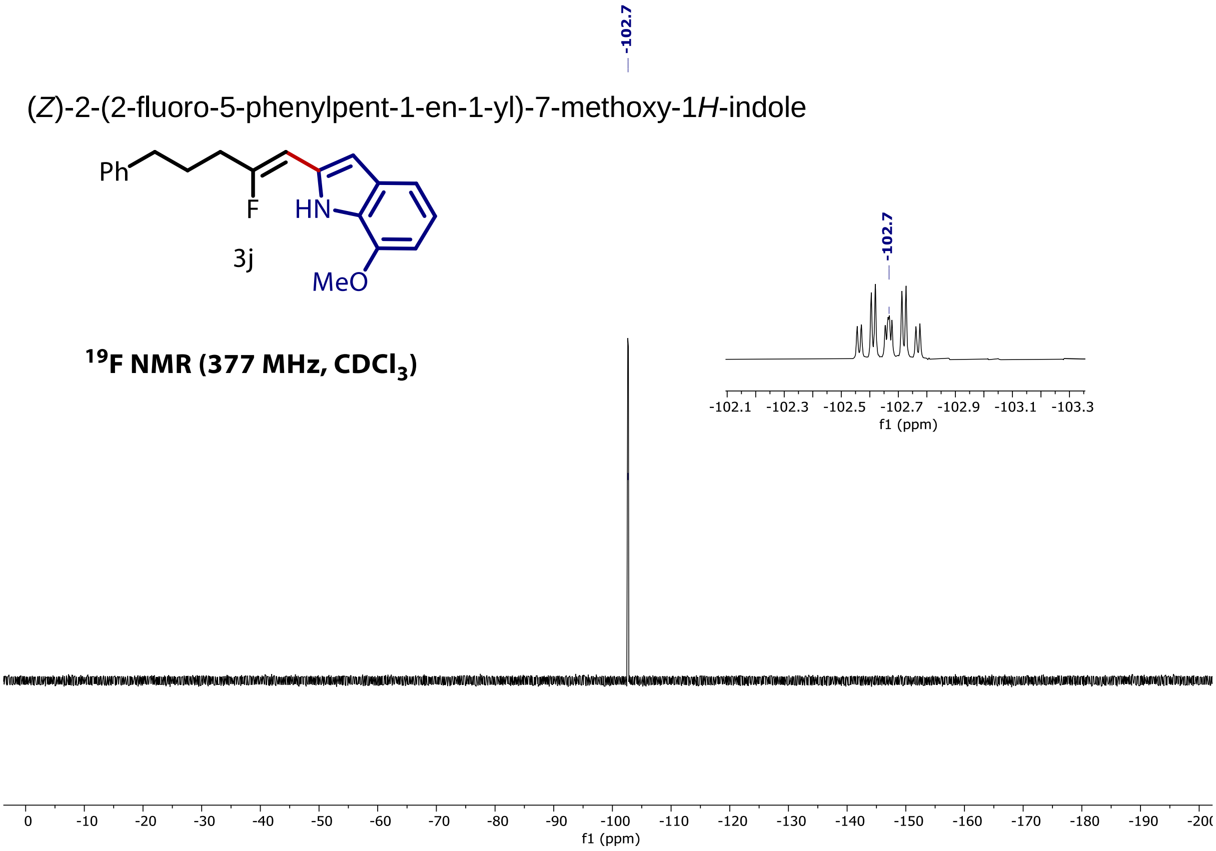
-102.7
(Z)-2-(2-fluoro-5-phenylpent-1-en-1-yl)-7-methoxy-1H-indole
Ph
F
HN
3j
MeO
19F NMR (377 MHz, CDCl3)
-102.7
f1 (ppm)
f1 (ppm)

(*Z*)-2-(2-fluoro-5-phenylpent-1-en-1-yl)-5-iodo-1*H*-indole

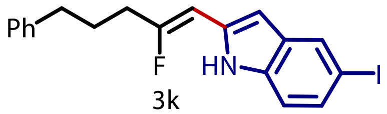

3k

^{1}H NMR (400 MHz, $CDCl_3$)

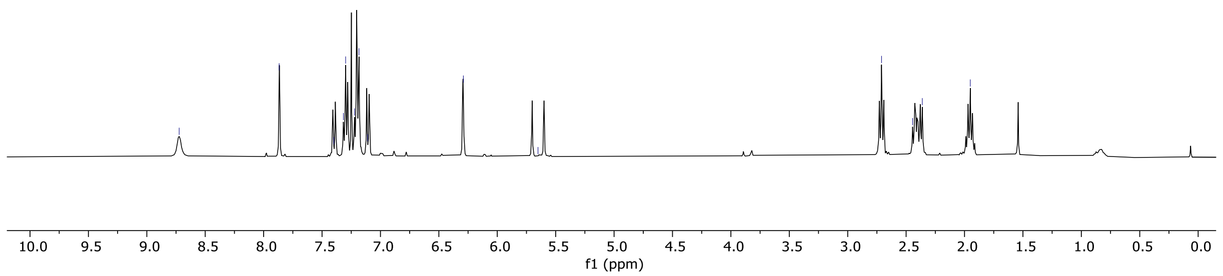

(*Z*)-2-(2-fluoro-5-phenylpent-1-en-1-yl)-5-iodo-1*H*-indole

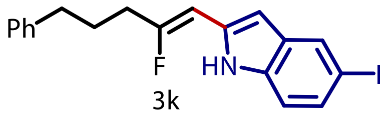

3k

^{13}C NMR (151 MHz, $CDCl_3$)

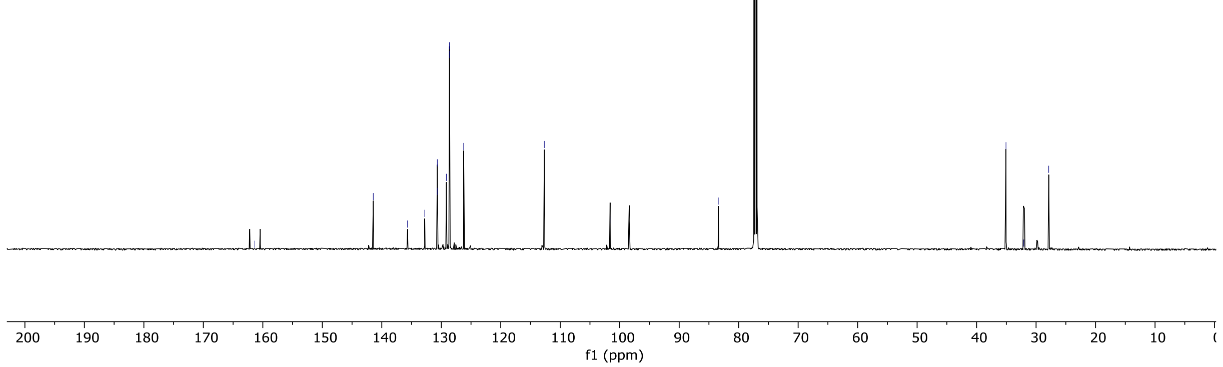

(*Z*)-2-(2-fluoro-5-phenylpent-1-en-1-yl)-5-iodo-1*H*-indole

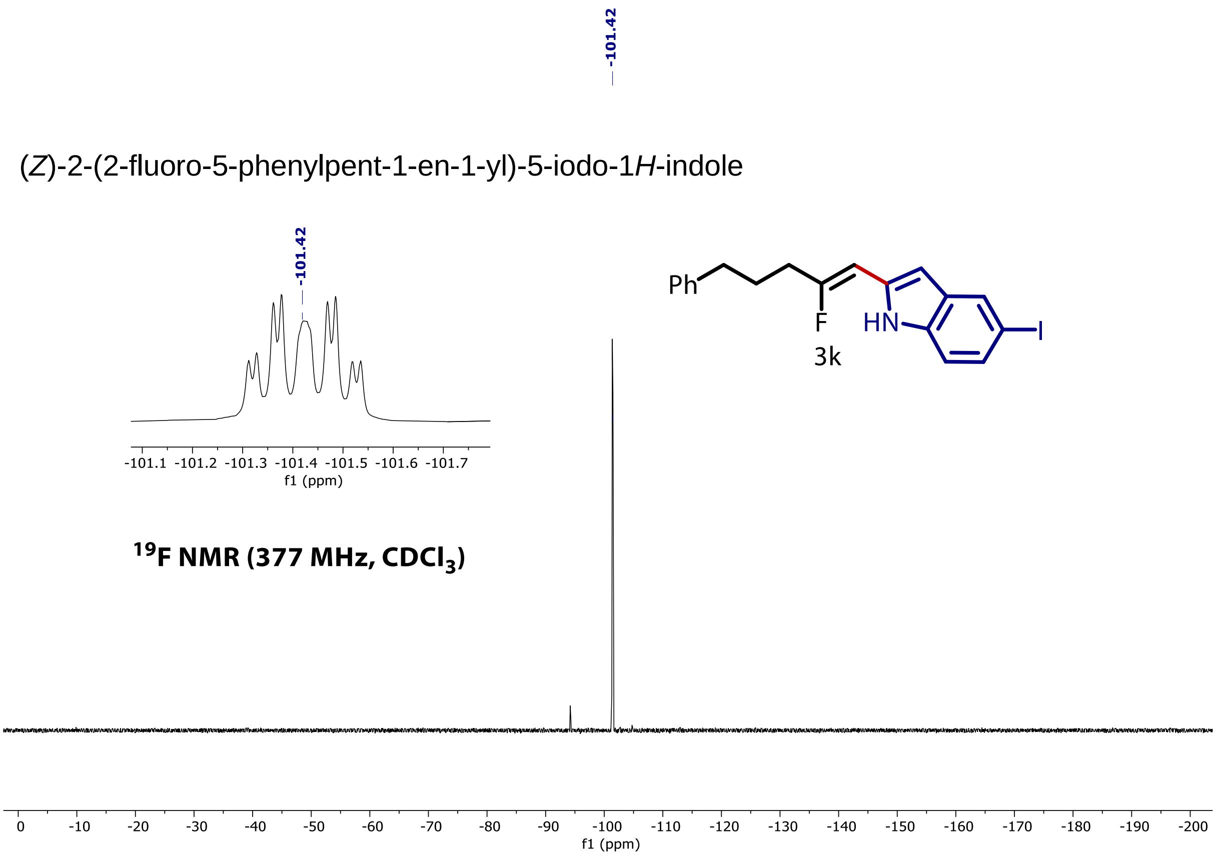

(*Z*)-5-bromo-2-(2-fluoro-5-phenylpent-1-en-1-yl)-1*H*-indole

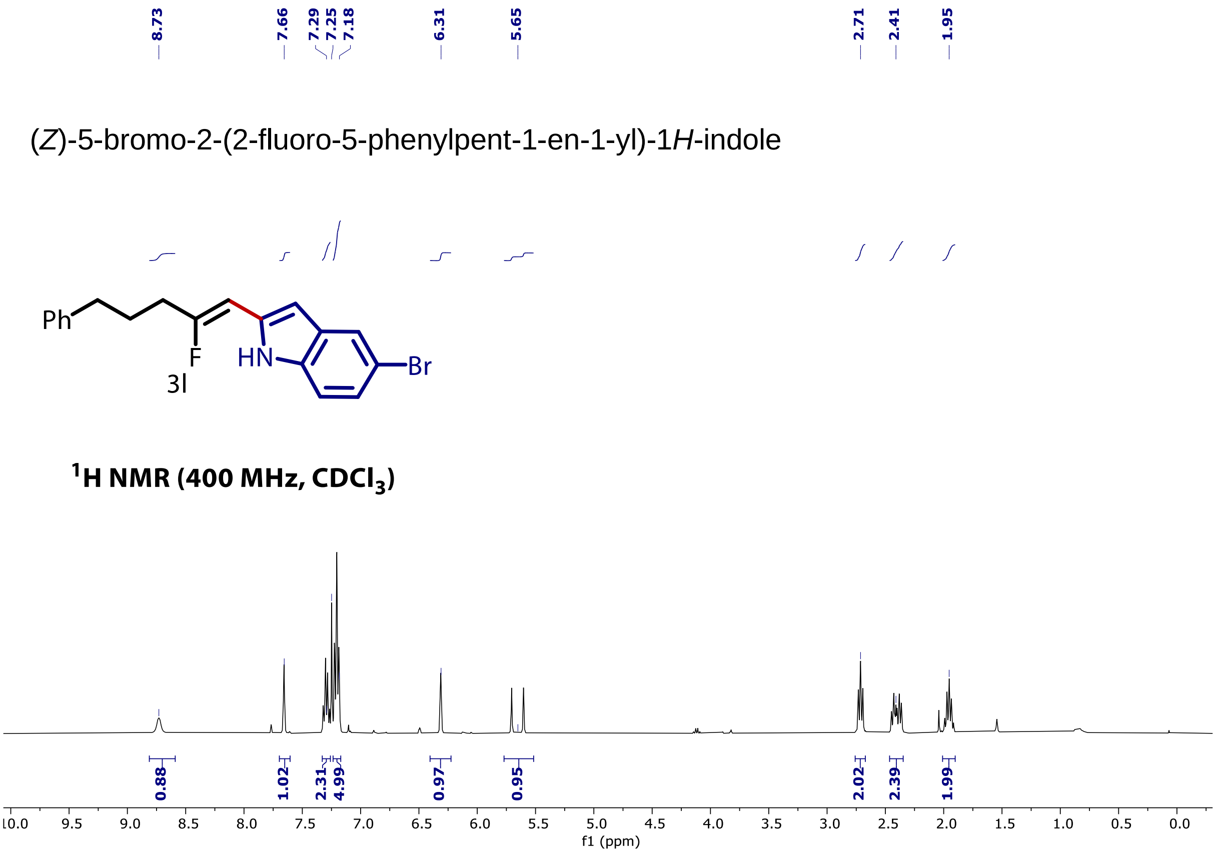

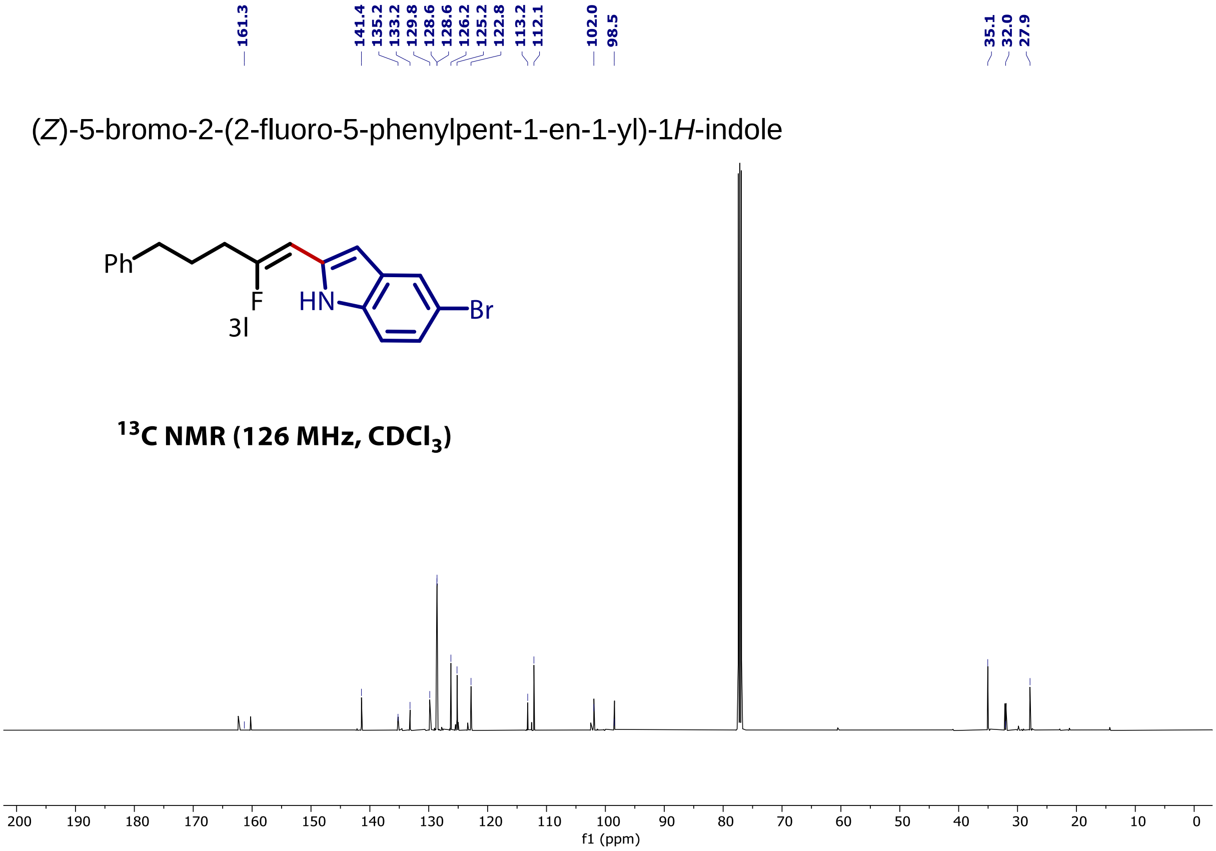
161.3
141.4
135.2
133.2
129.8
128.6
128.6
126.2
125.2
122.8
113.2
112.1
102.0
98.5
35.1
32.0
27.9
(Z)-5-bromo-2-(2-fluoro-5-phenylpent-1-en-1-yl)-1H-indole
Ph
F
HN
Br
3l
13C NMR (126 MHz, CDCl3)
f1 (ppm)

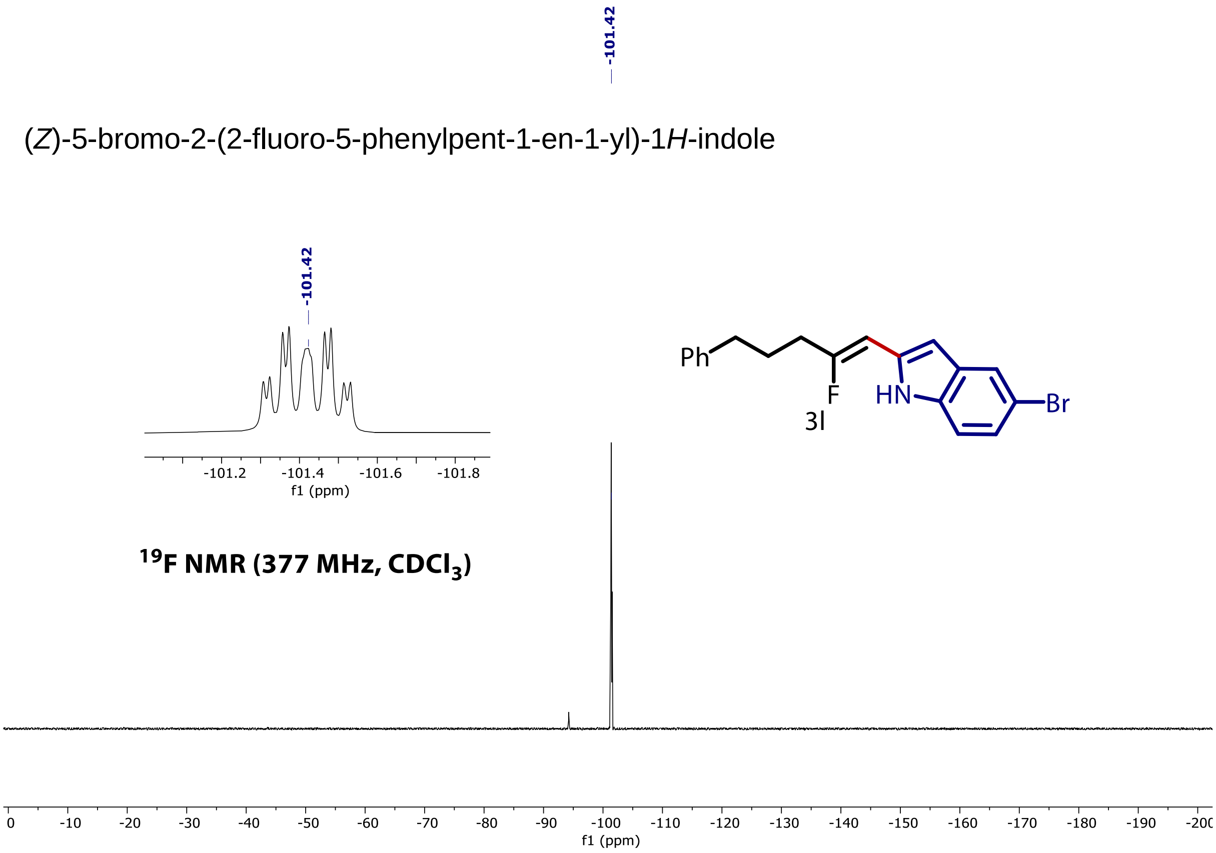
-101.42
(Z)-5-bromo-2-(2-fluoro-5-phenylpent-1-en-1-yl)-1H-indole
-101.42
-101.2
-101.4
-101.6
-101.8
f1 (ppm)
Ph
F
HN
Br
3l
19F NMR (377 MHz, CDCl3)
f1 (ppm)

(*Z*)-5-fluoro-2-(2-fluoro-5-phenylpent-1-en-1-yl)-1*H*-indole

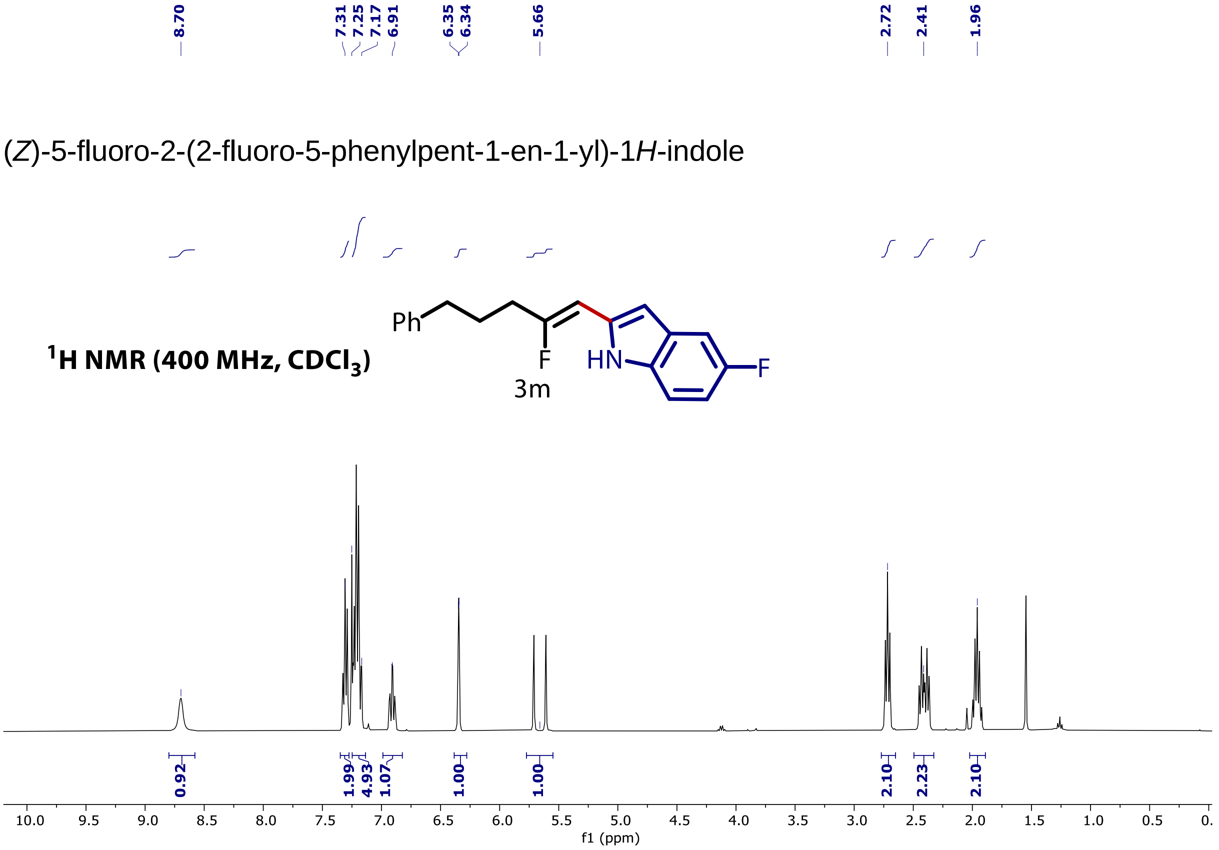

(*Z*)-5-fluoro-2-(2-fluoro-5-phenylpent-1-en-1-yl)-1*H*-indole

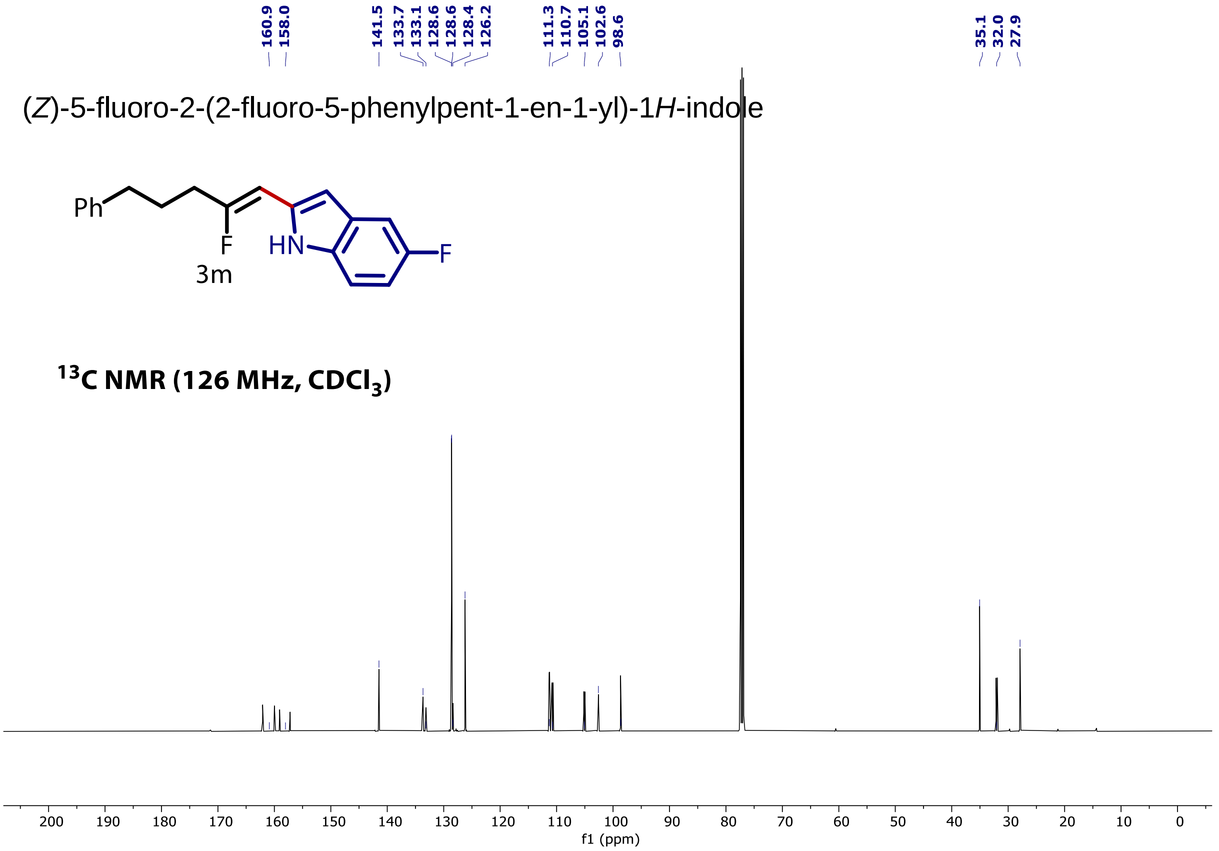

(*Z*)-5-fluoro-2-(2-fluoro-5-phenylpent-1-en-1-yl)-1*H*-indole

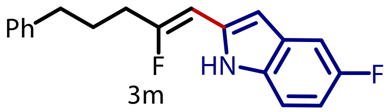

3m

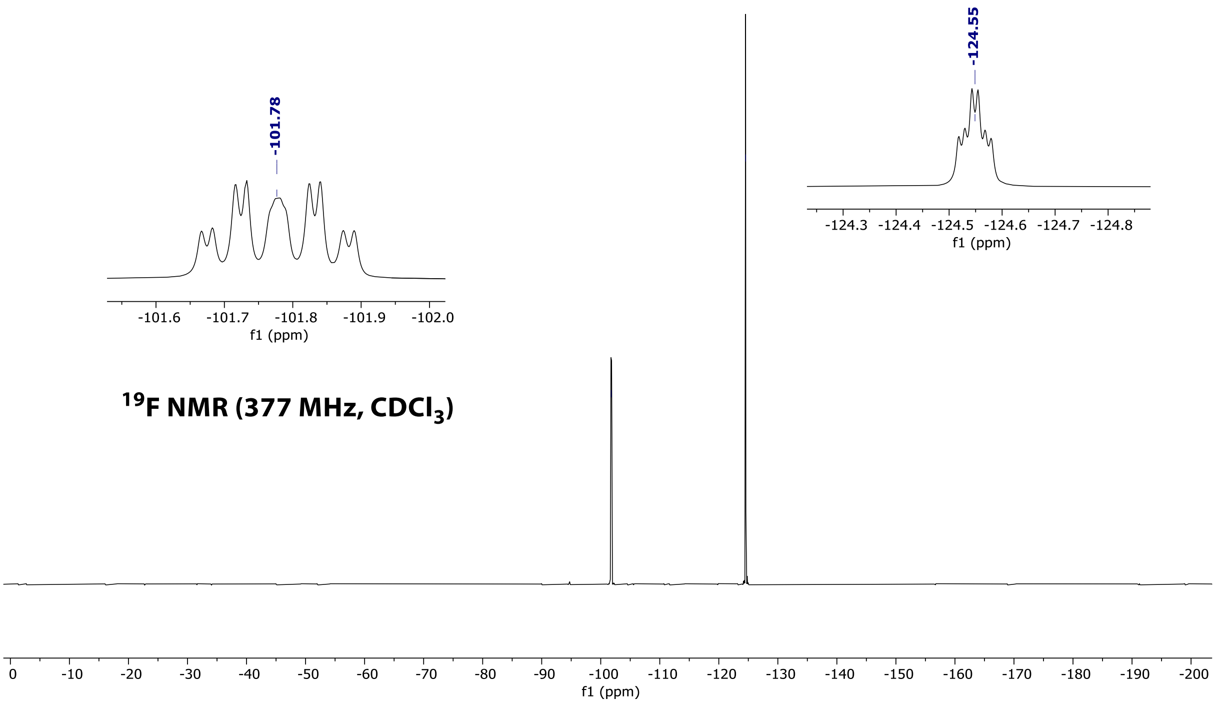

^{19}F NMR (377 MHz, $CDCl_3$)

(*Z*)-4-chloro-2-(2-fluoro-5-phenylpent-1-en-1-yl)-1*H*-indole

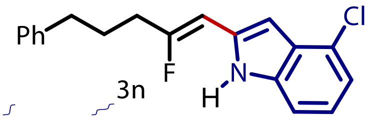

3n

^{1}H NMR (400 MHz, $CDCl_3$)

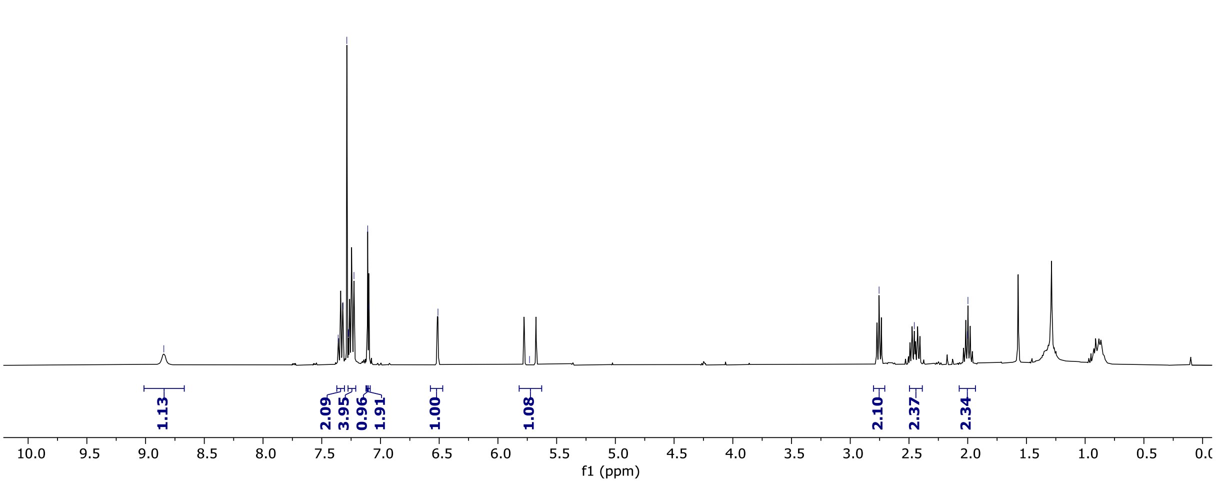

(*Z*)-4-chloro-2-(2-fluoro-5-phenylpent-1-en-1-yl)-1*H*-indole

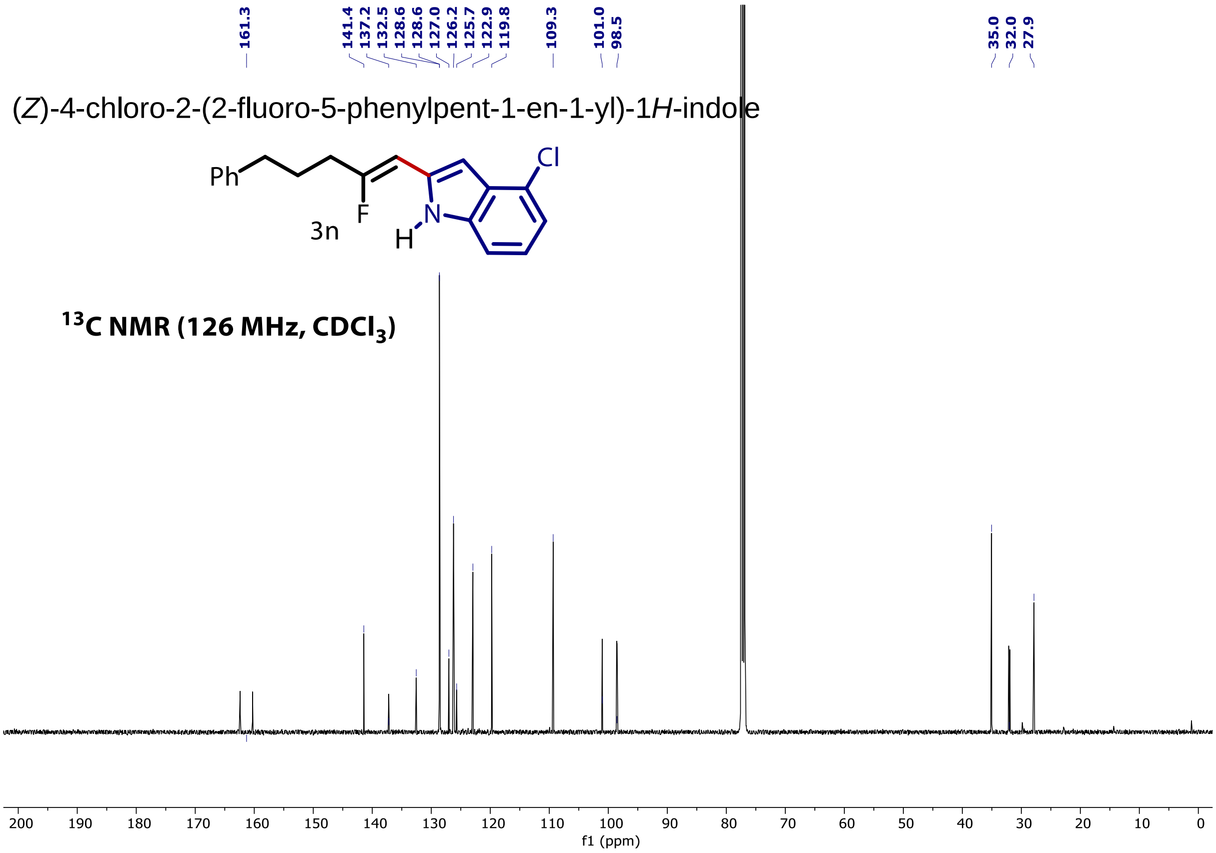

(*Z*)-4-chloro-2-(2-fluoro-5-phenylpent-1-en-1-yl)-1*H*-indole

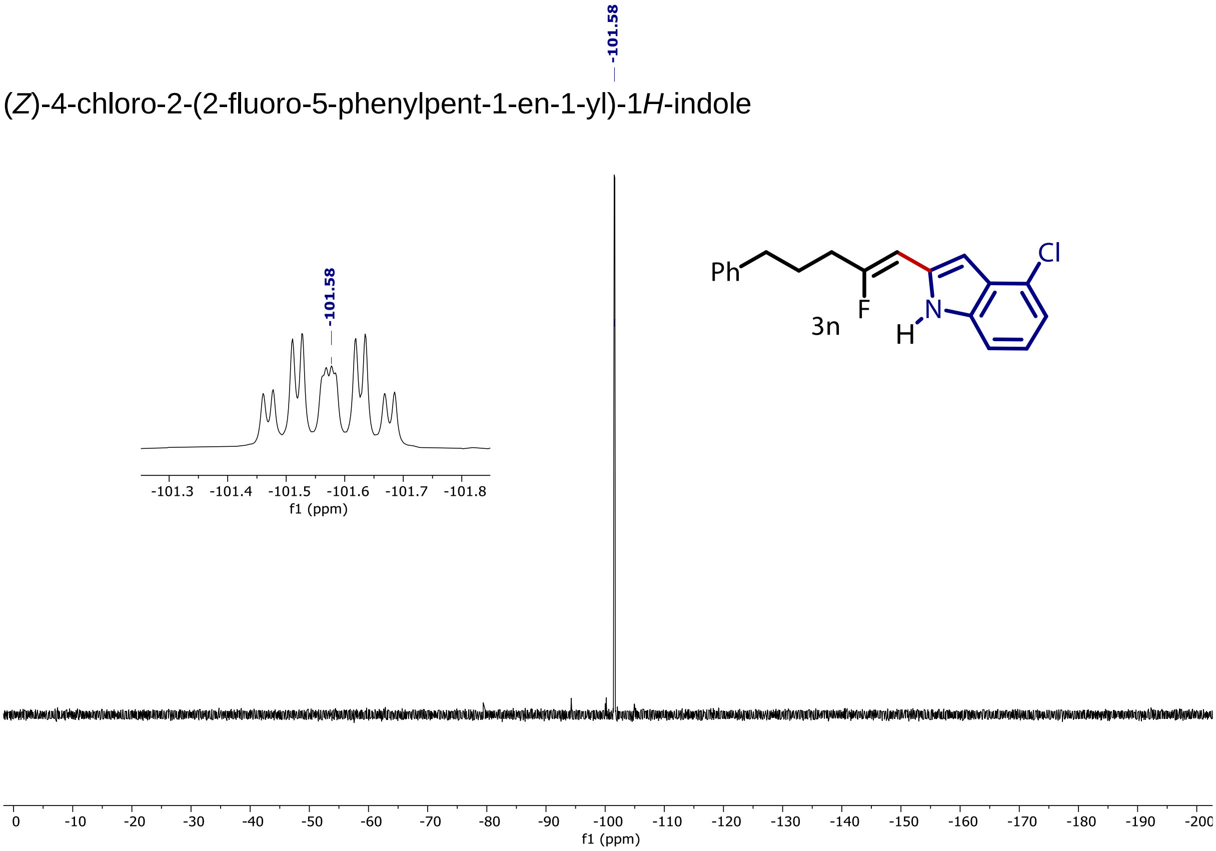

(*Z*)-4-chloro-2-(2-fluoro-5-phenylpent-1-en-1-yl)-1-methyl-1*H*-indole

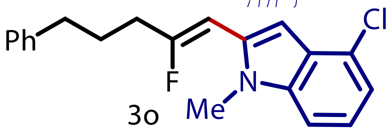

3o

^{1}H NMR (400 MHz, $CDCl_3$)

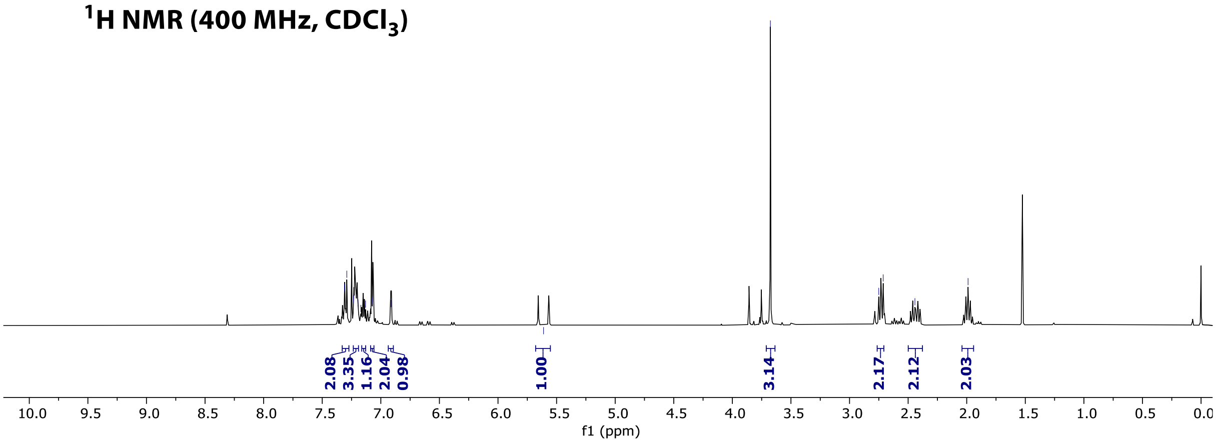

(*Z*)-4-chloro-2-(2-fluoro-5-phenylpent-1-en-1-yl)-1-methyl-1*H*-indole

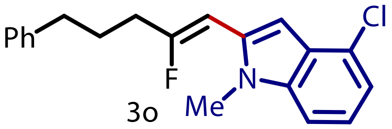

3o

^{13}C NMR (101 MHz, $CDCl_3$)

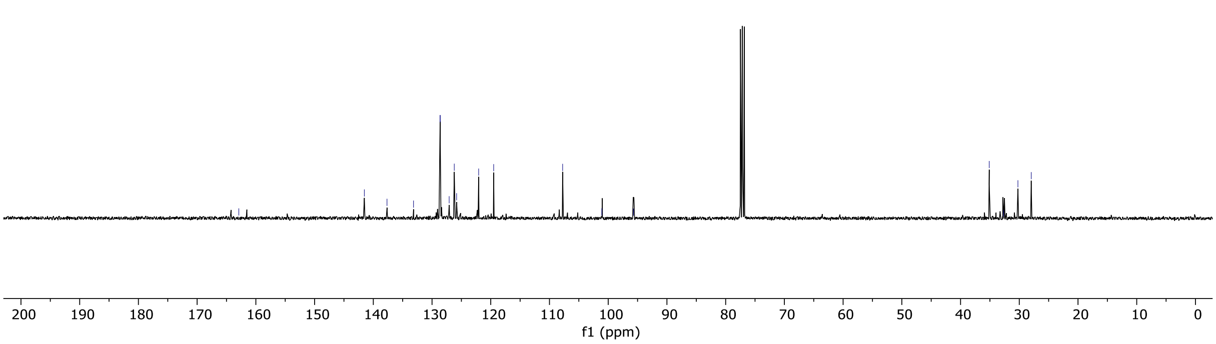

(*Z*)-4-chloro-2-(2-fluoro-5-phenylpent-1-en-1-yl)-1-methyl-1*H*-indole

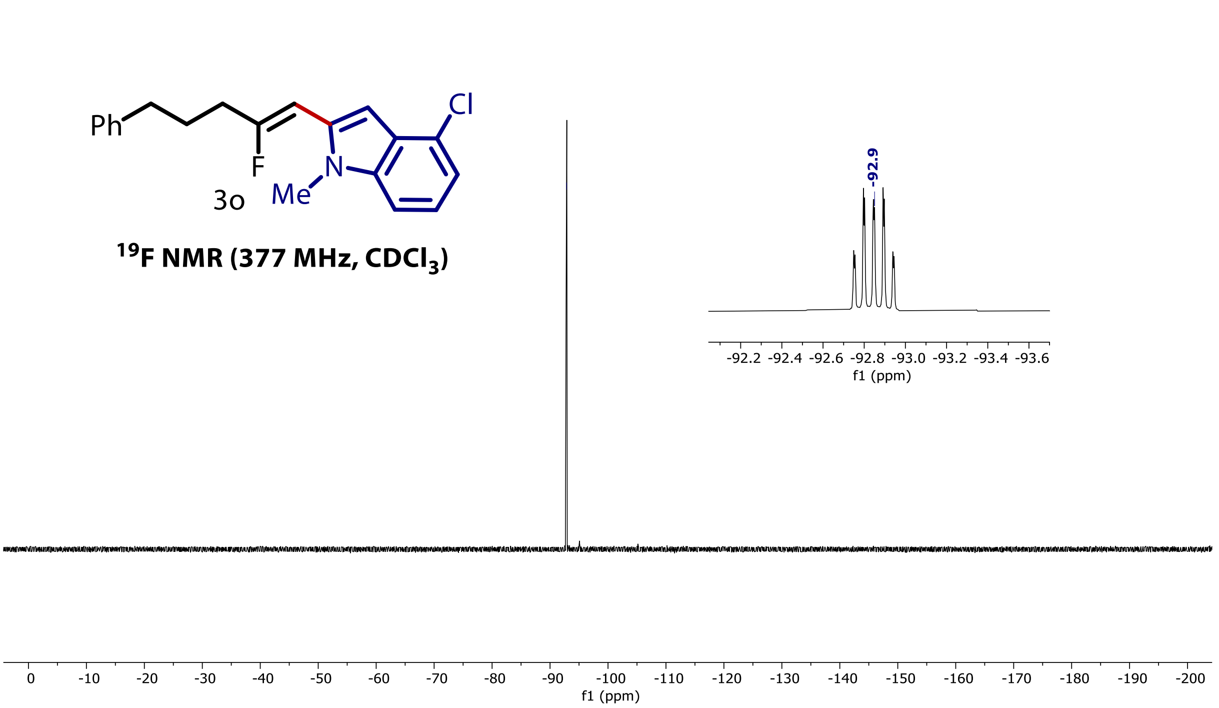

Methyl (*Z*)-2-(2-fluoro-5-phenylpent-1-en-1-yl)-1*H*-indole-5-carboxylate

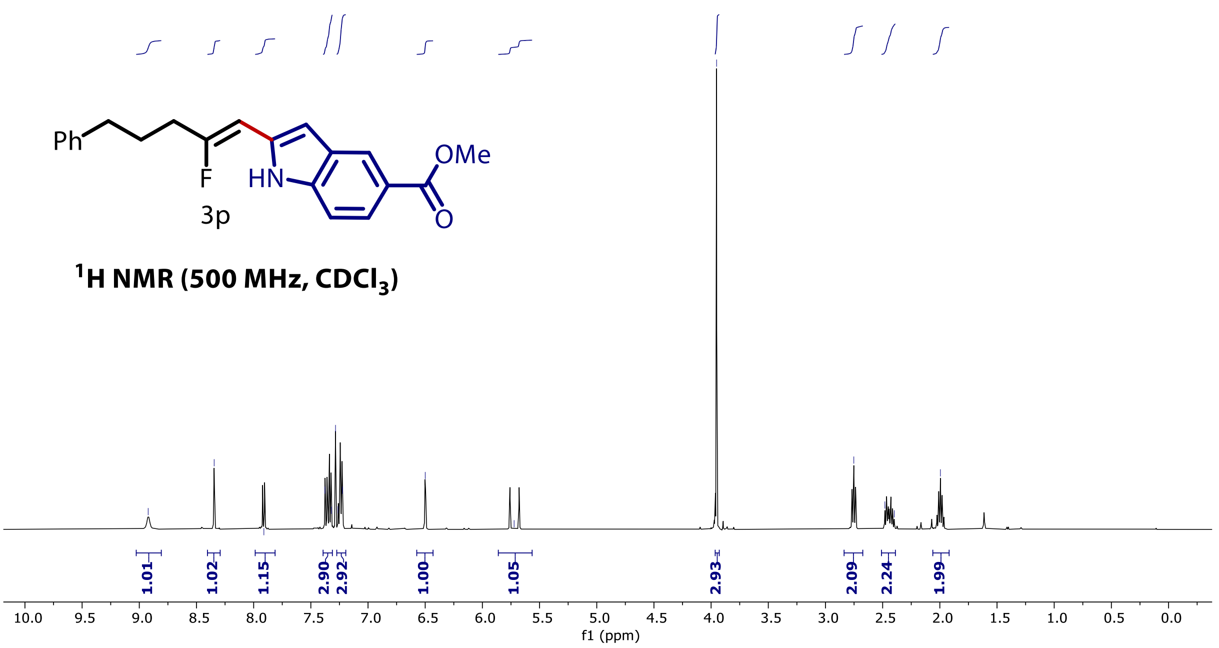

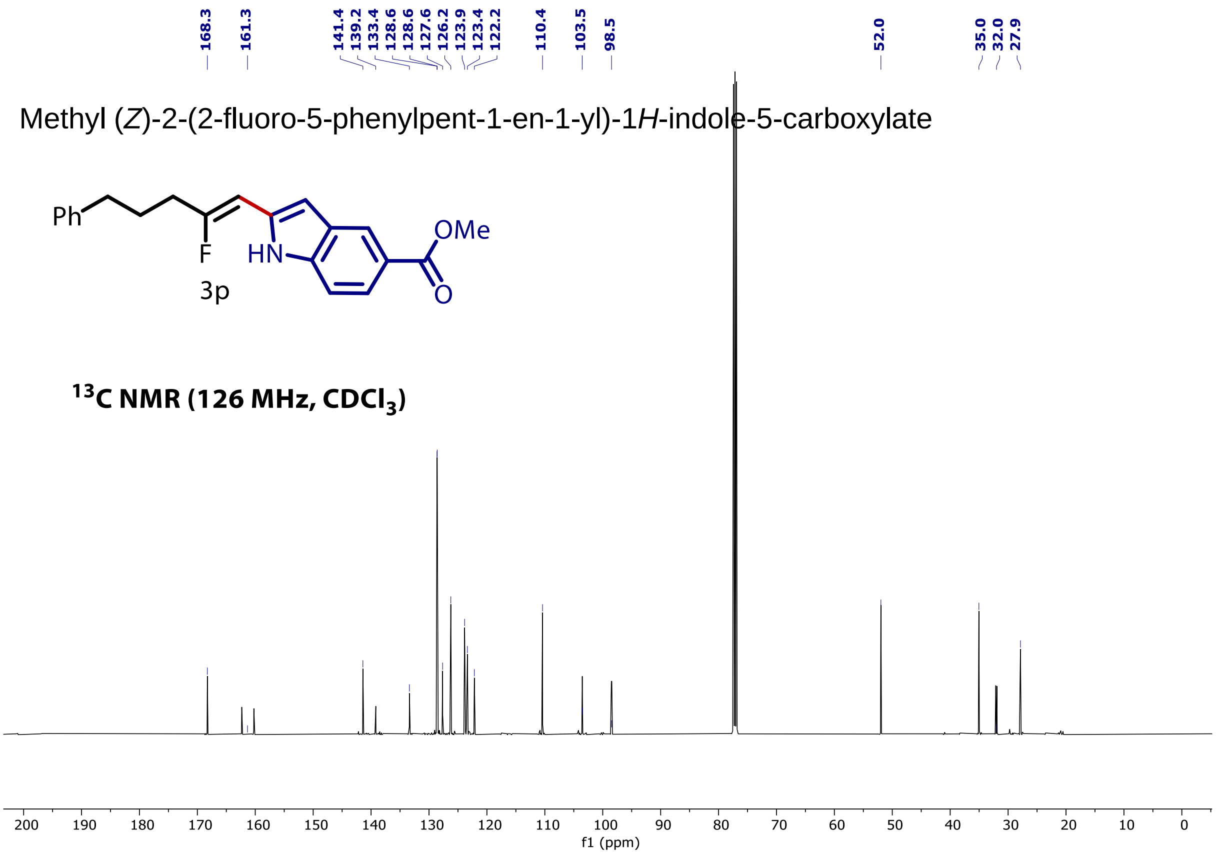
168.3
161.3
141.4
139.2
133.4
128.6
128.6
127.6
126.2
123.9
123.4
122.2
110.4
103.5
98.5
52.0
35.0
32.0
27.9
Methyl (Z)-2-(2-fluoro-5-phenylpent-1-en-1-yl)-1H-indole-5-carboxylate
Ph
F
HN
OMe
O
3p
13C NMR (126 MHz, CDCl3)
200 190 180 170 160 150 140 130 120 110 100 90 80 70 60 50 40 30 20 10 0
f1 (ppm)

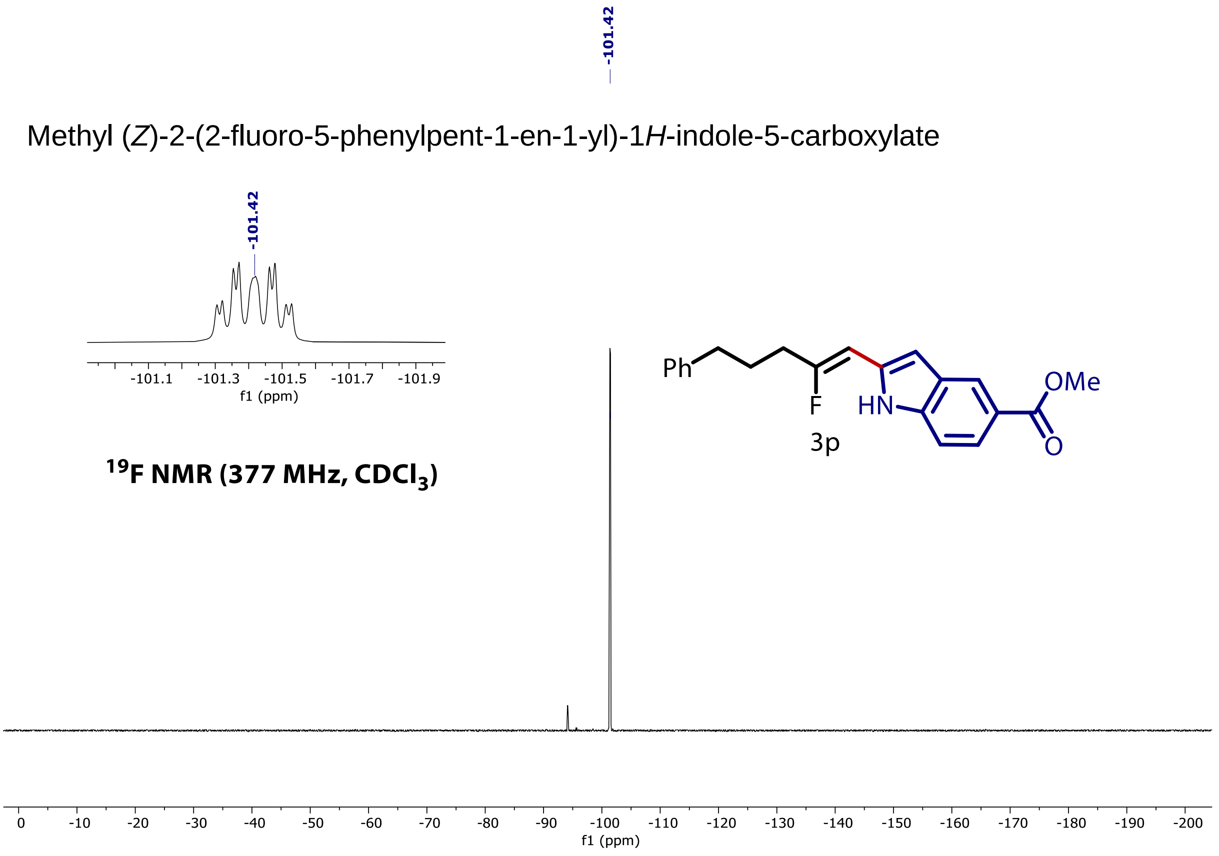
-101.42
Methyl (Z)-2-(2-fluoro-5-phenylpent-1-en-1-yl)-1H-indole-5-carboxylate
-101.42
-101.1 -101.3 -101.5 -101.7 -101.9
f1 (ppm)
19F NMR (377 MHz, CDCl3)
Ph
F
HN
OMe
O
3p
0 -10 -20 -30 -40 -50 -60 -70 -80 -90 -100 -110 -120 -130 -140 -150 -160 -170 -180 -190 -200
f1 (ppm)

Ethyl (*Z*)-2-(2-fluoro-5-phenylpent-1-en-1-yl)-1*H*-indole-4-carboxylate

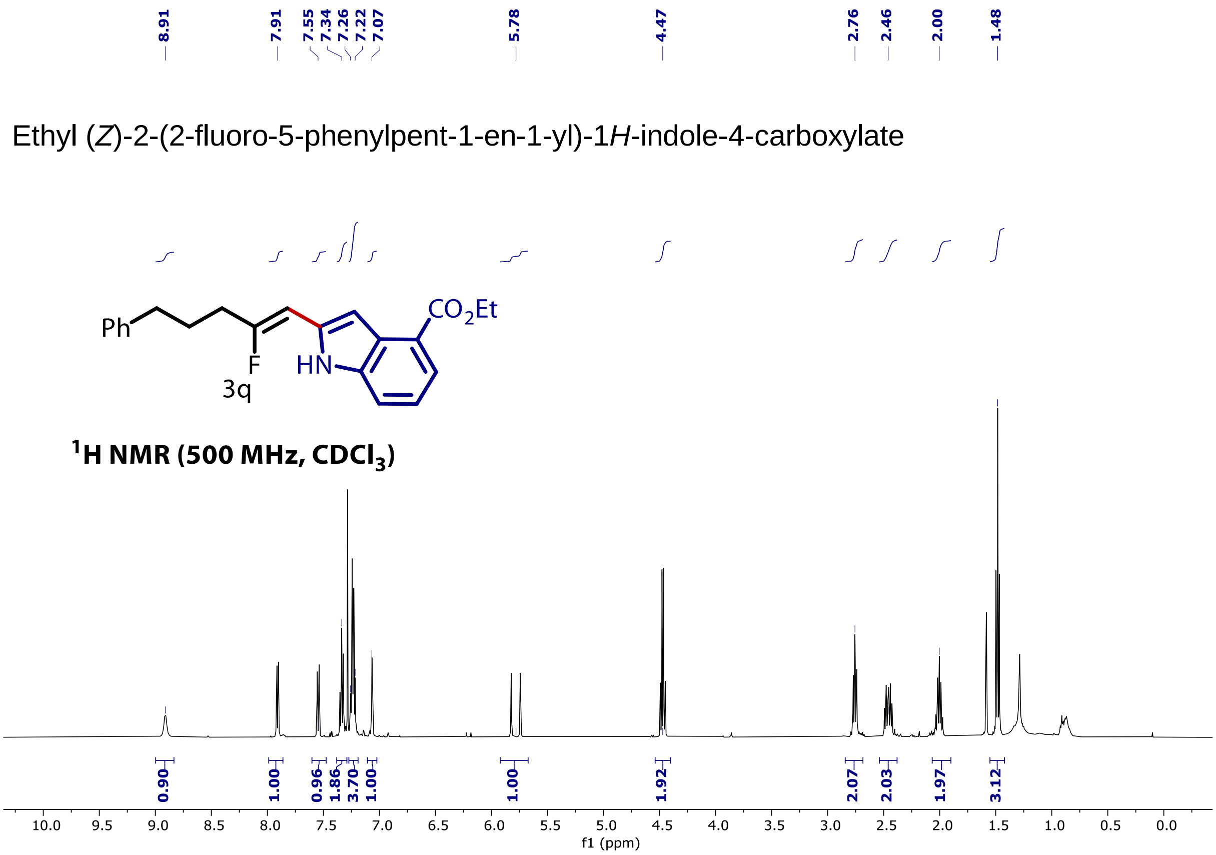

Ethyl (*Z*)-2-(2-fluoro-5-phenylpent-1-en-1-yl)-1*H*-indole-4-carboxylate

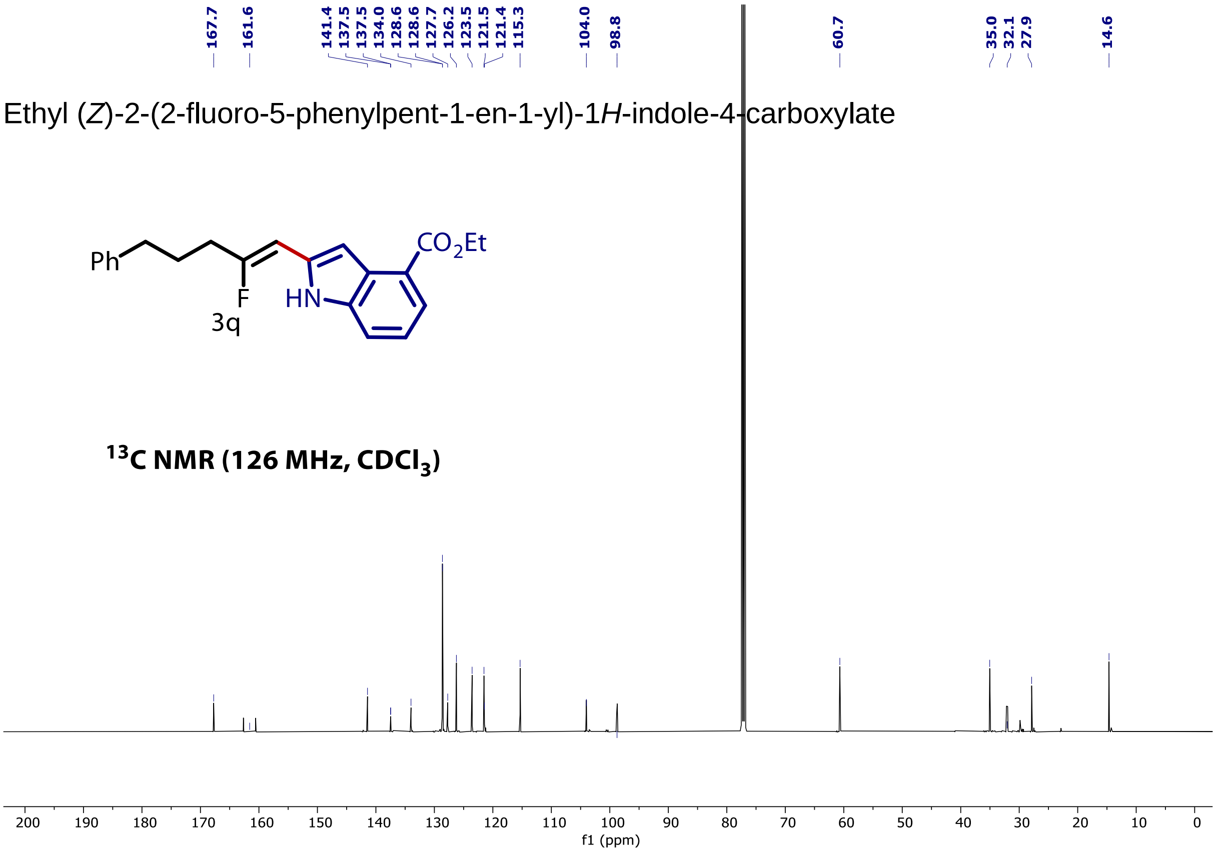

Ethyl (*Z*)-2-(2-fluoro-5-phenylpent-1-en-1-yl)-1*H*-indole-4-carboxylate

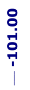

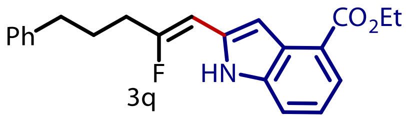

3q

^{19}F NMR (377 MHz, $CDCl_3$)

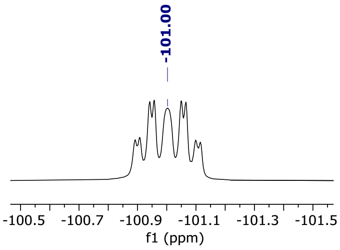

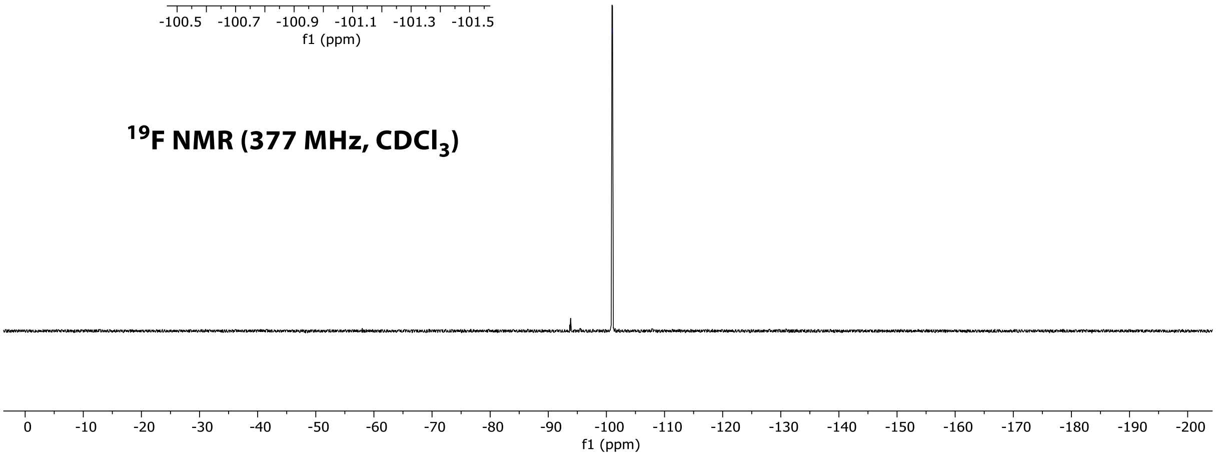

(*Z*)-2-(2-fluoro-5-phenylpent-1-en-1-yl)-1*H*-indol-5-ol

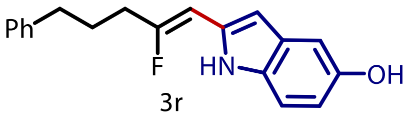

3r

^{1}H NMR (400 MHz, $CDCl_3$)

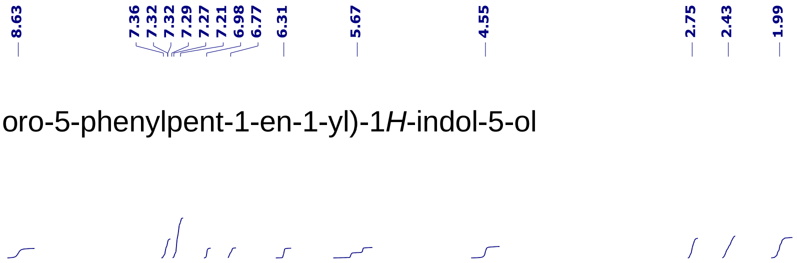

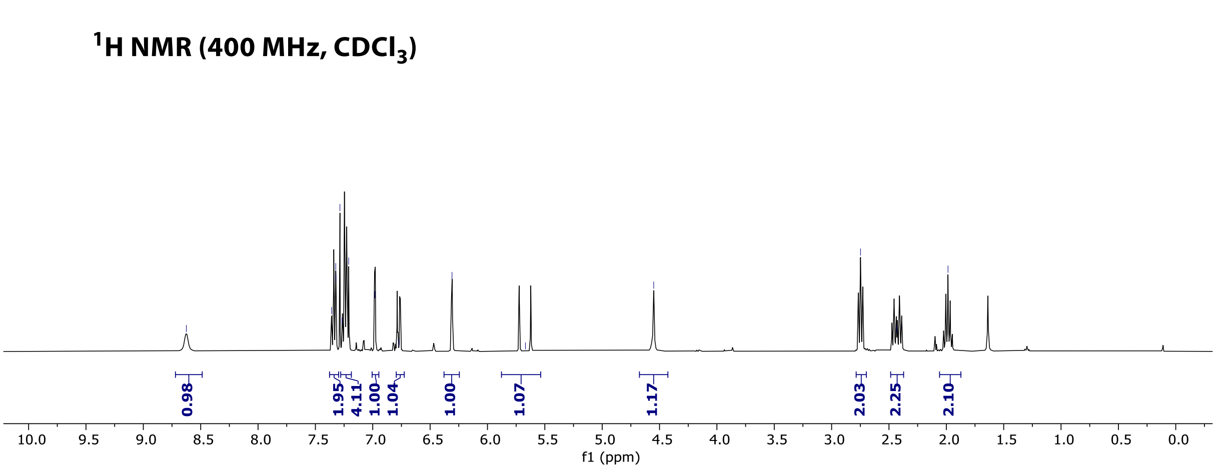

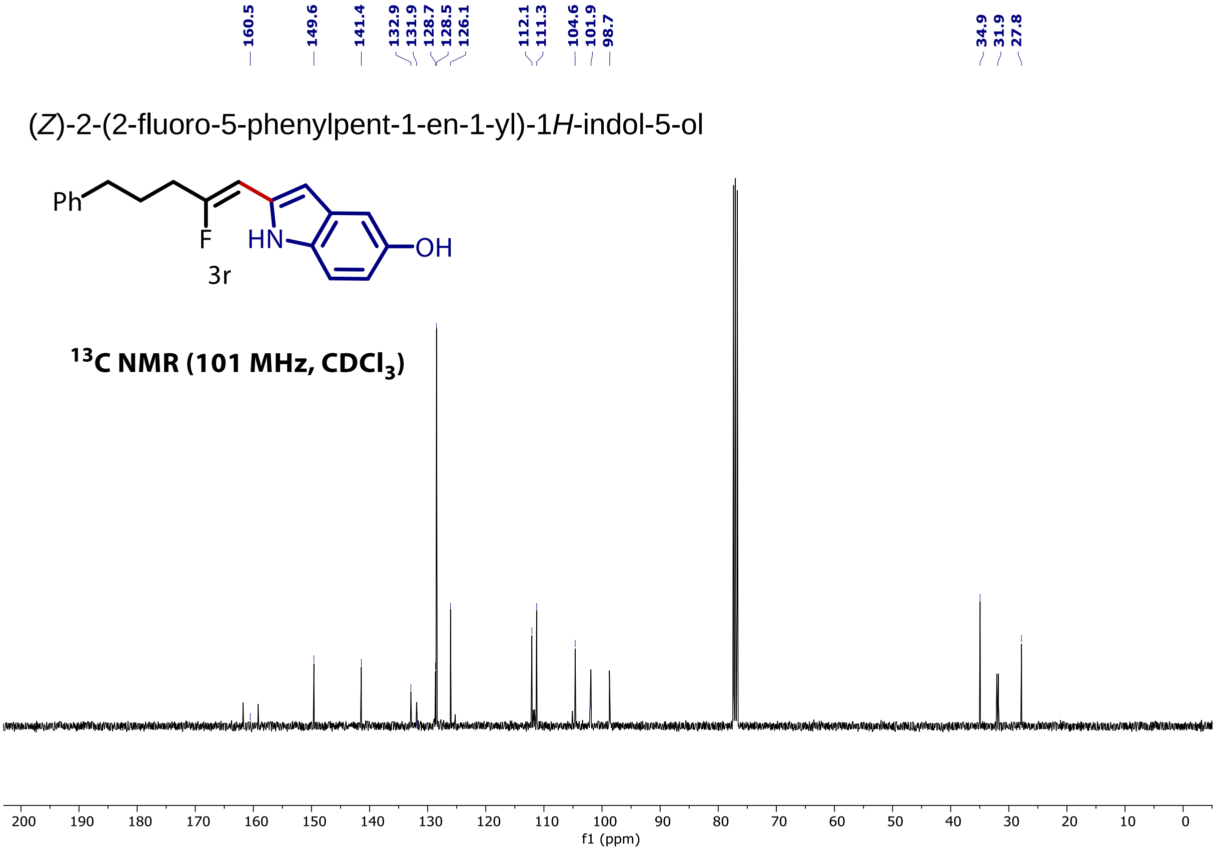

160.5
149.6
141.4
132.9
131.9
128.7
128.5
126.1
112.1
111.3
104.6
101.9
98.7
34.9
31.9
27.8
(Z)-2-(2-fluoro-5-phenylpent-1-en-1-yl)-1H-indol-5-ol
Ph
F
HN
OH
3r
13C NMR (101 MHz, CDCl3)
200 190 180 170 160 150 140 130 120 110 100 90 80 70 60 50 40 30 20 10 0
f1 (ppm)

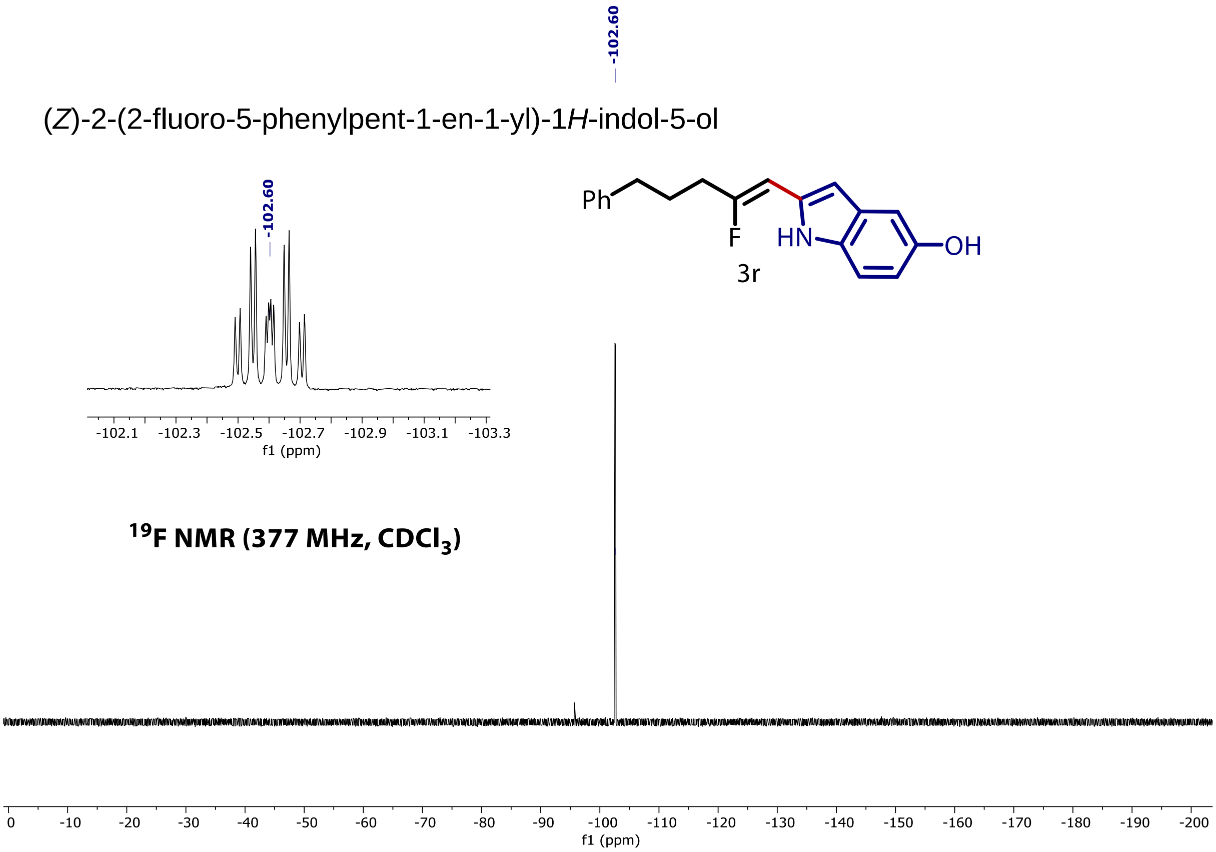

-102.60
(Z)-2-(2-fluoro-5-phenylpent-1-en-1-yl)-1H-indol-5-ol
-102.60
-102.1 -102.3 -102.5 -102.7 -102.9 -103.1 -103.3
f1 (ppm)
Ph
F
HN
OH
3r
19F NMR (377 MHz, CDCl3)
0 -10 -20 -30 -40 -50 -60 -70 -80 -90 -100 -110 -120 -130 -140 -150 -160 -170 -180 -190 -200
f1 (ppm)

(*Z*)-2-(2-fluoro-5-phenylpent-1-en-1-yl)-3-methyl-1*H*-indole

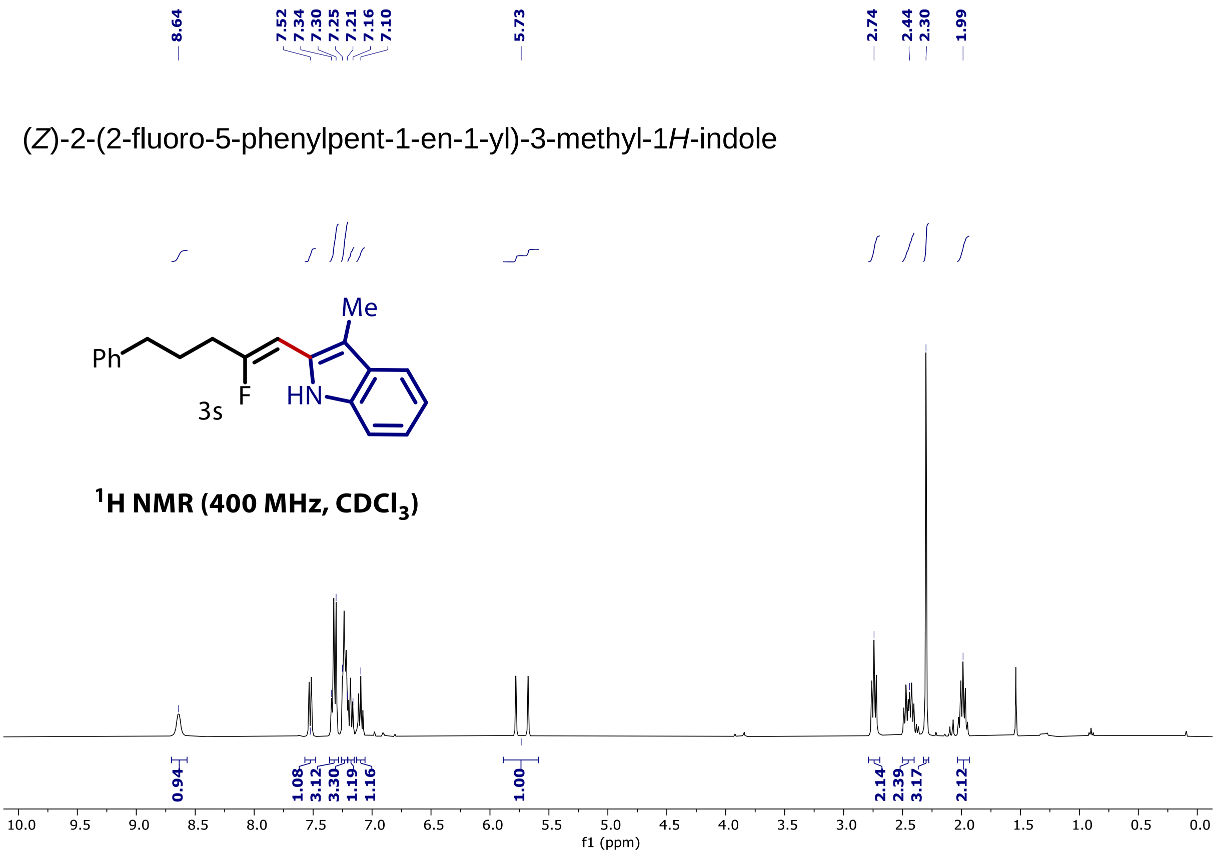

^{1}H NMR (400 MHz, $CDCl_3$)

(*Z*)-2-(2-fluoro-5-phenylpent-1-en-1-yl)-3-methyl-1*H*-indole

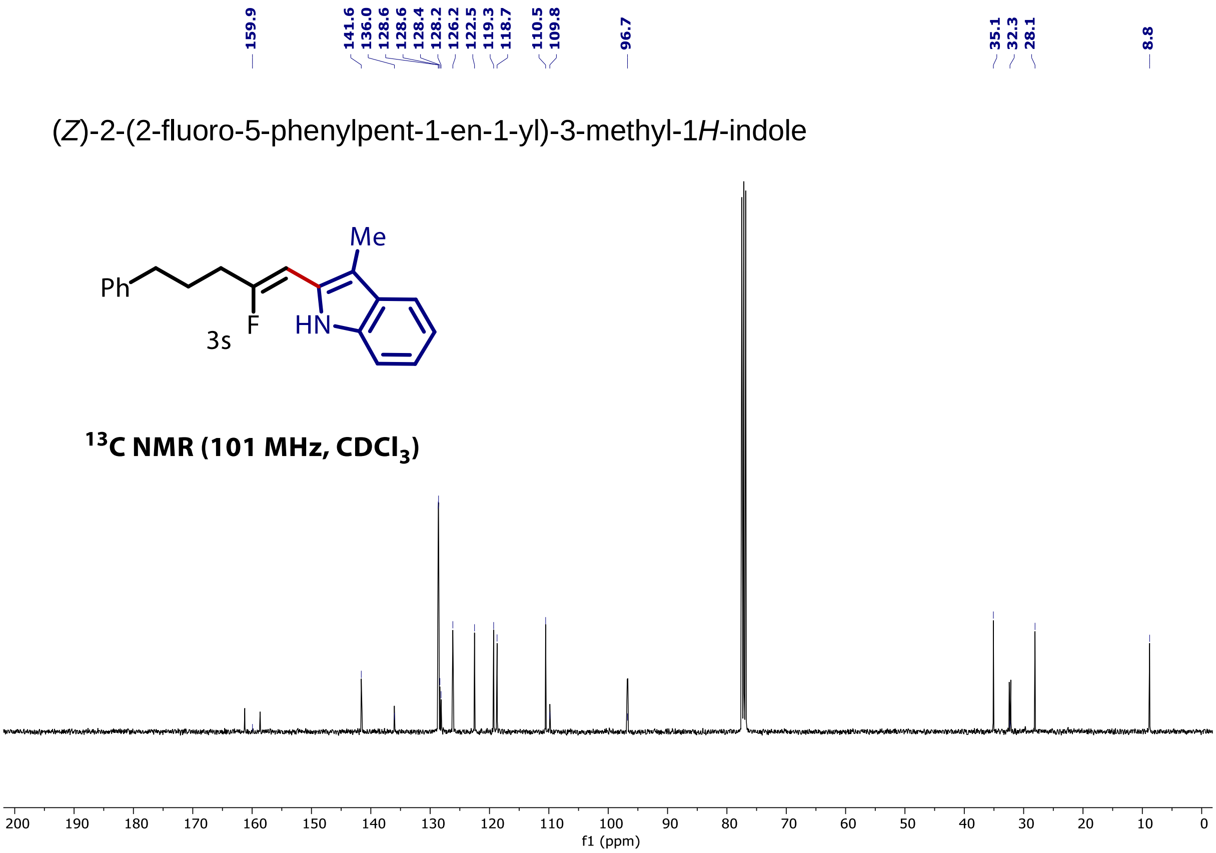

^{13}C NMR (101 MHz, $CDCl_3$)

-104.97

(*Z*)-2-(2-fluoro-5-phenylpent-1-en-1-yl)-3-methyl-1*H*-indole

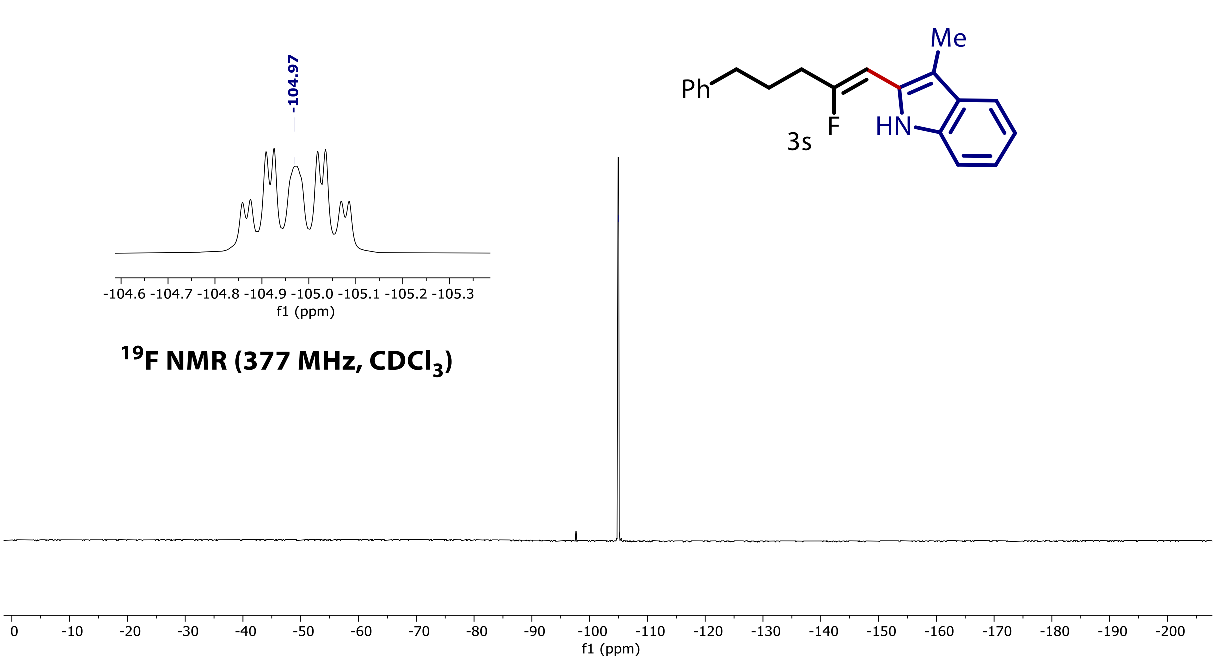

8.76 7.55 7.35 7.30 7.23 7.18 7.10 5.78 3.85 3.02 2.73 2.44 1.98

(*Z*)-2-(2-(2-fluoro-5-phenylpent-1-en-1-yl)-1*H*-indol-3-yl)ethan-1-ol

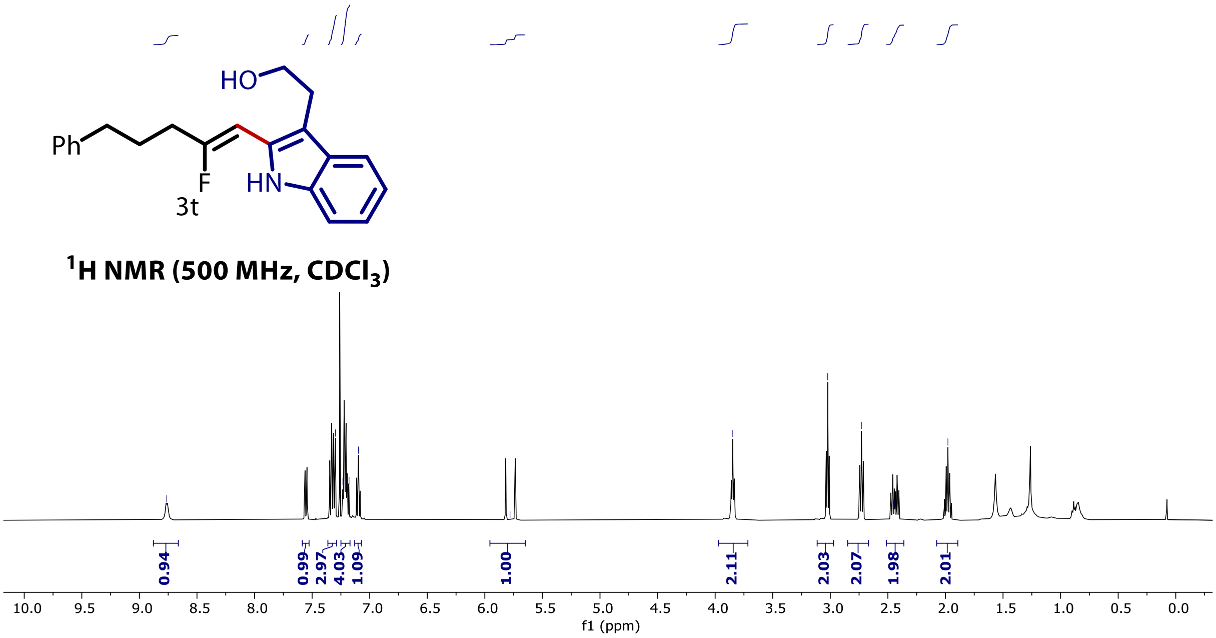

(*Z*)-2-(2-(2-fluoro-5-phenylpent-1-en-1-yl)-1*H*-indol-3-yl)ethan-1-ol

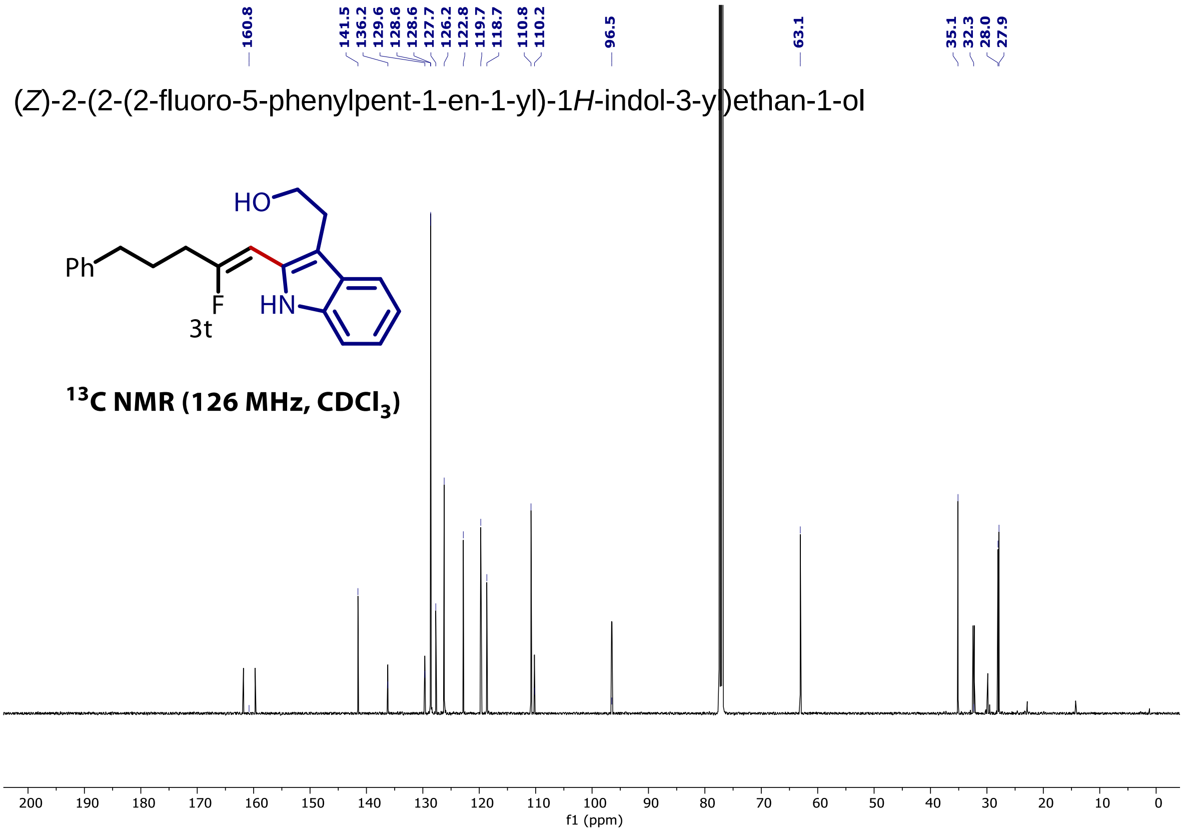

(*Z*)-2-(2-(2-fluoro-5-phenylpent-1-en-1-yl)-1*H*-indol-3-yl)ethan-1-ol

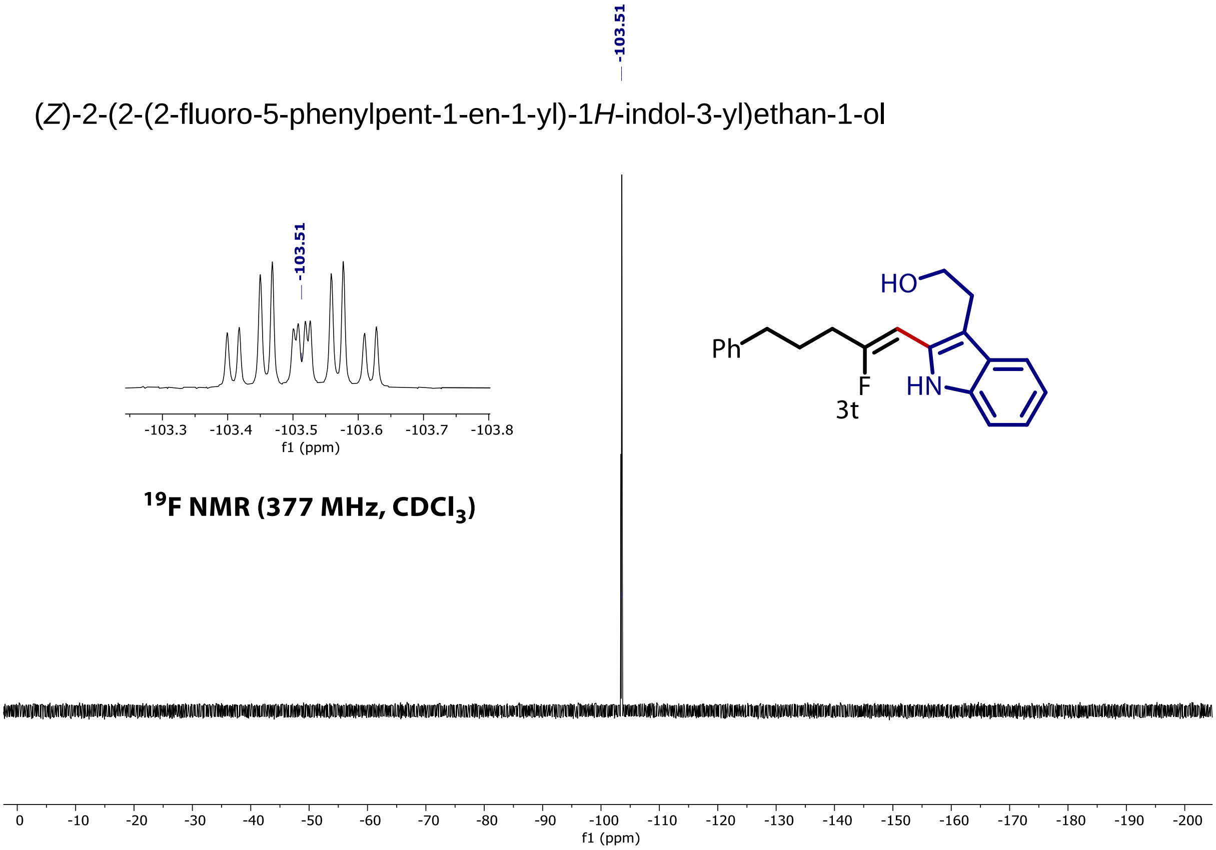

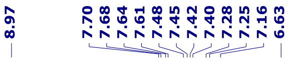

(*Z*)-2-(2-(2-fluoro-2-phenylvinyl)-1*H*-indol-3-yl)ethan-1-ol

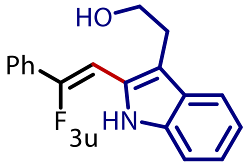

^{1}H NMR (500 MHz, $CDCl_3$)

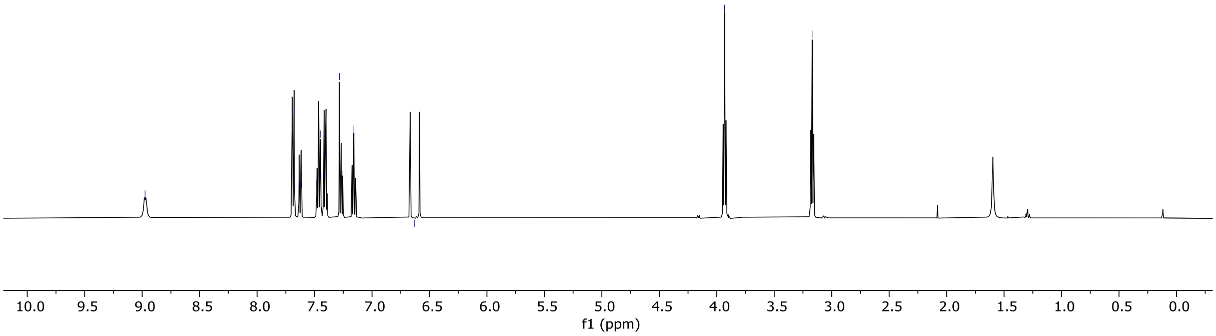

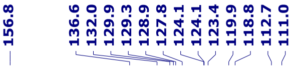

(*Z*)-2-(2-(2-fluoro-2-phenylvinyl)-1*H*-indol-3-yl)ethan-1-ol

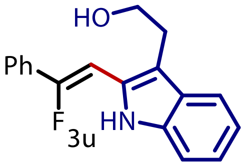

^{13}C NMR (126 MHz, $CDCl_3$)

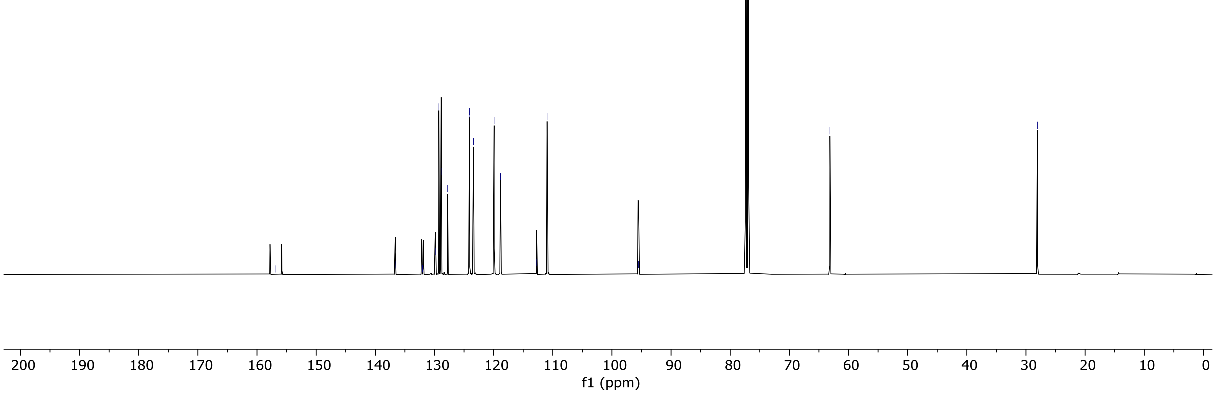

(*Z*)-2-(2-(2-fluoro-2-phenylvinyl)-1*H*-indol-3-yl)ethan-1-ol

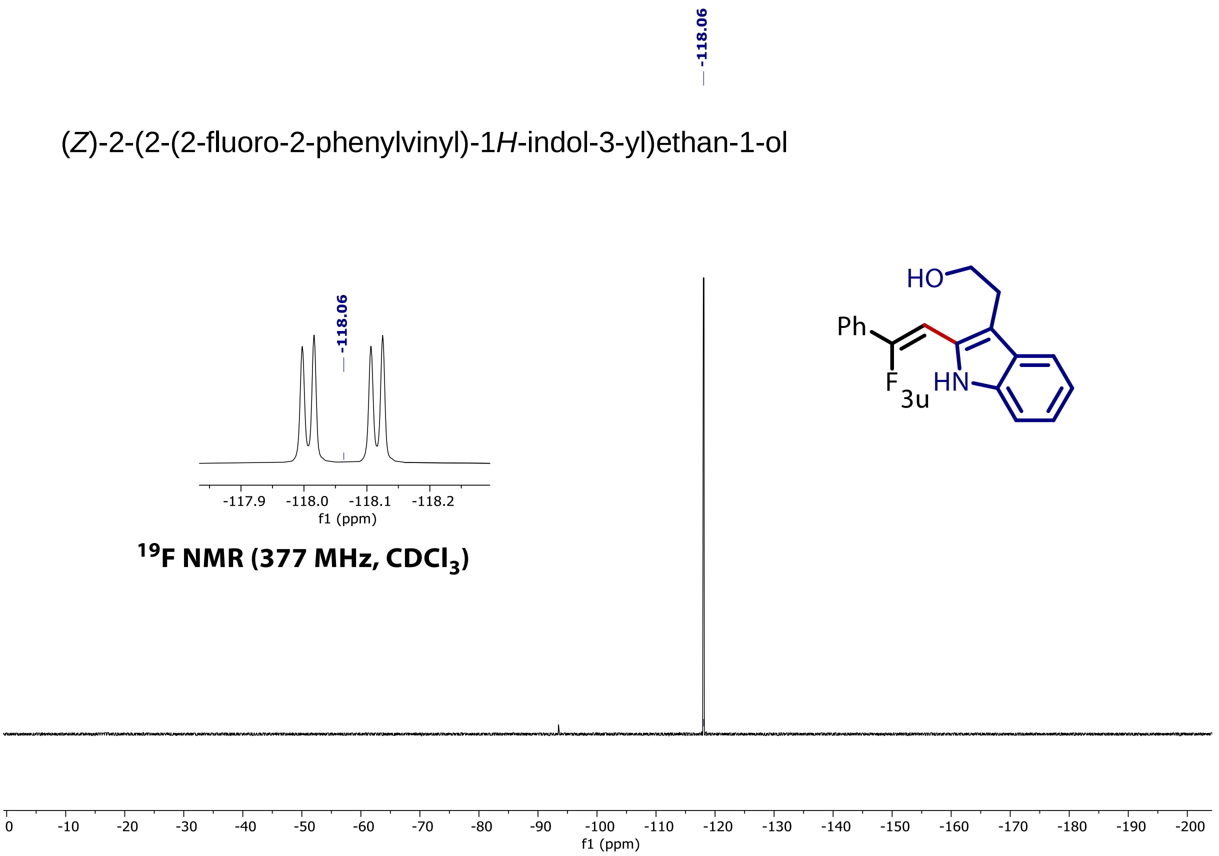

(*Z*)-2-(2-fluorododec-1-en-1-yl)-1-methyl-1*H*-indole

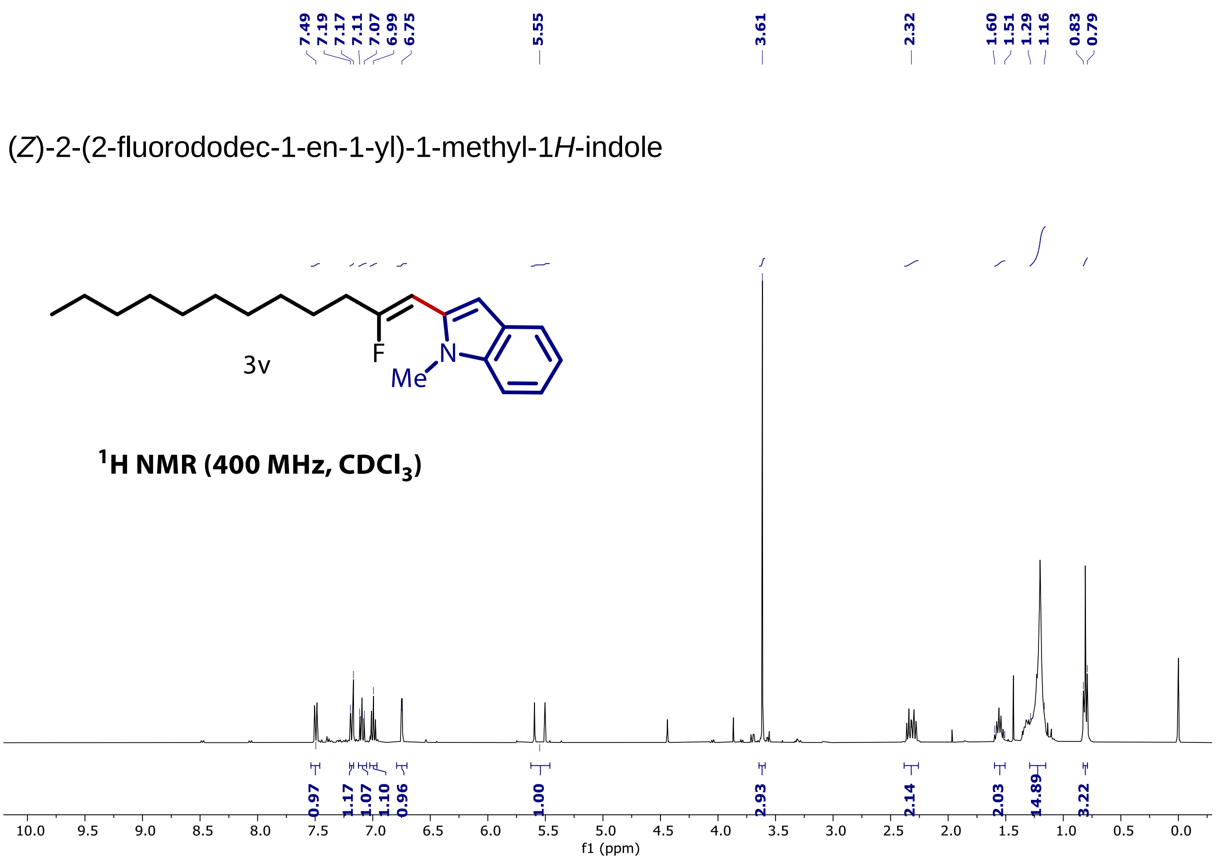

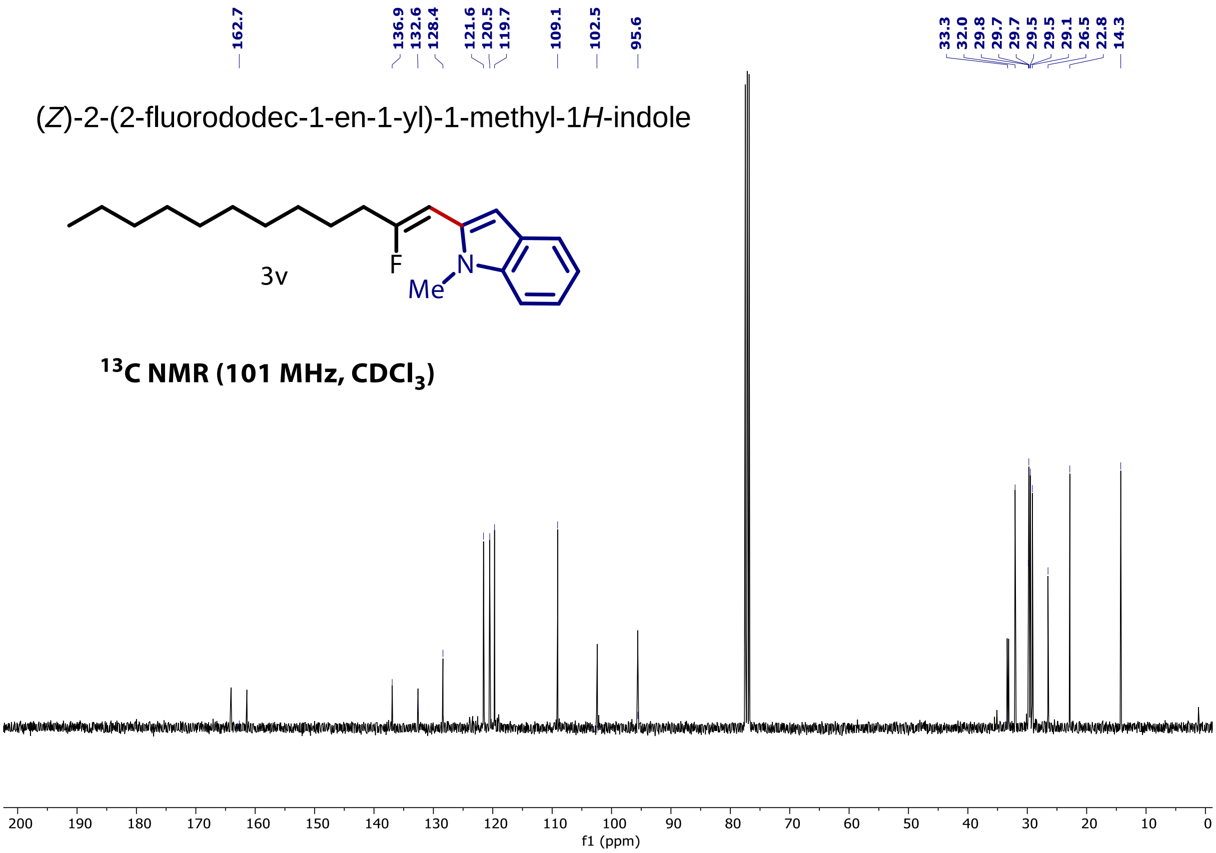
162.7
136.9
132.6
128.4
121.6
120.5
119.7
109.1
102.5
95.6
33.3
32.0
29.8
29.7
29.7
29.5
29.5
29.1
26.5
22.8
14.3
(Z)-2-(2-fluorododec-1-en-1-yl)-1-methyl-1H-indole
3v
F
N
Me
13C NMR (101 MHz, CDCl3)
f1 (ppm)

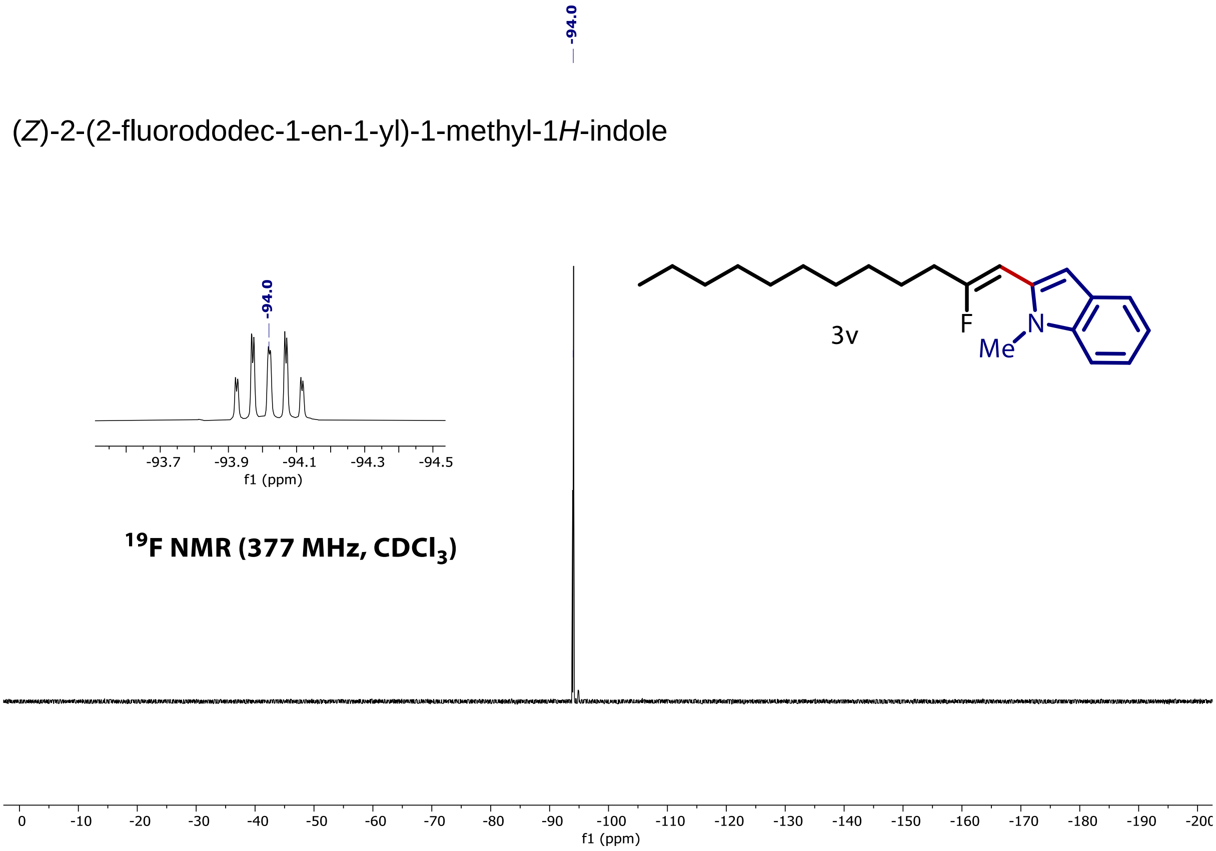
-94.0
(Z)-2-(2-fluorododec-1-en-1-yl)-1-methyl-1H-indole
3v
F
N
Me
-93.7
-93.9
-94.1
-94.3
-94.5
f1 (ppm)
19F NMR (377 MHz, CDCl3)
f1 (ppm)

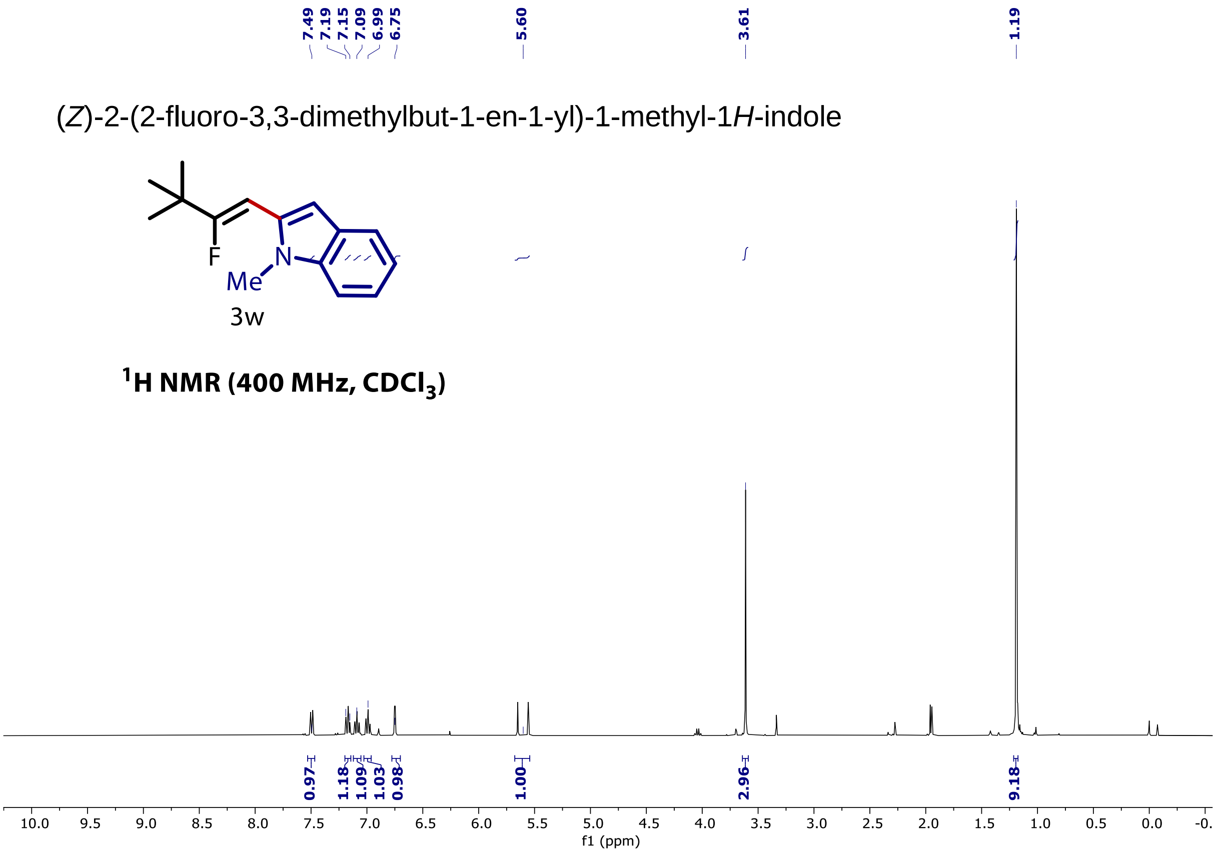

(Z)-2-(2-fluoro-3,3-dimethylbut-1-en-1-yl)-1-methyl-1H-indole
3w
1H NMR (400 MHz, CDCl3)
7.49
7.19
7.15
7.09
6.99
6.75
5.60
3.61
1.19
0.97
1.18
1.09
1.03
0.98
1.00
2.96
9.18
10.0 9.5 9.0 8.5 8.0 7.5 7.0 6.5 6.0 5.5 5.0 4.5 4.0 3.5 3.0 2.5 2.0 1.5 1.0 0.5 0.0
f1 (ppm)

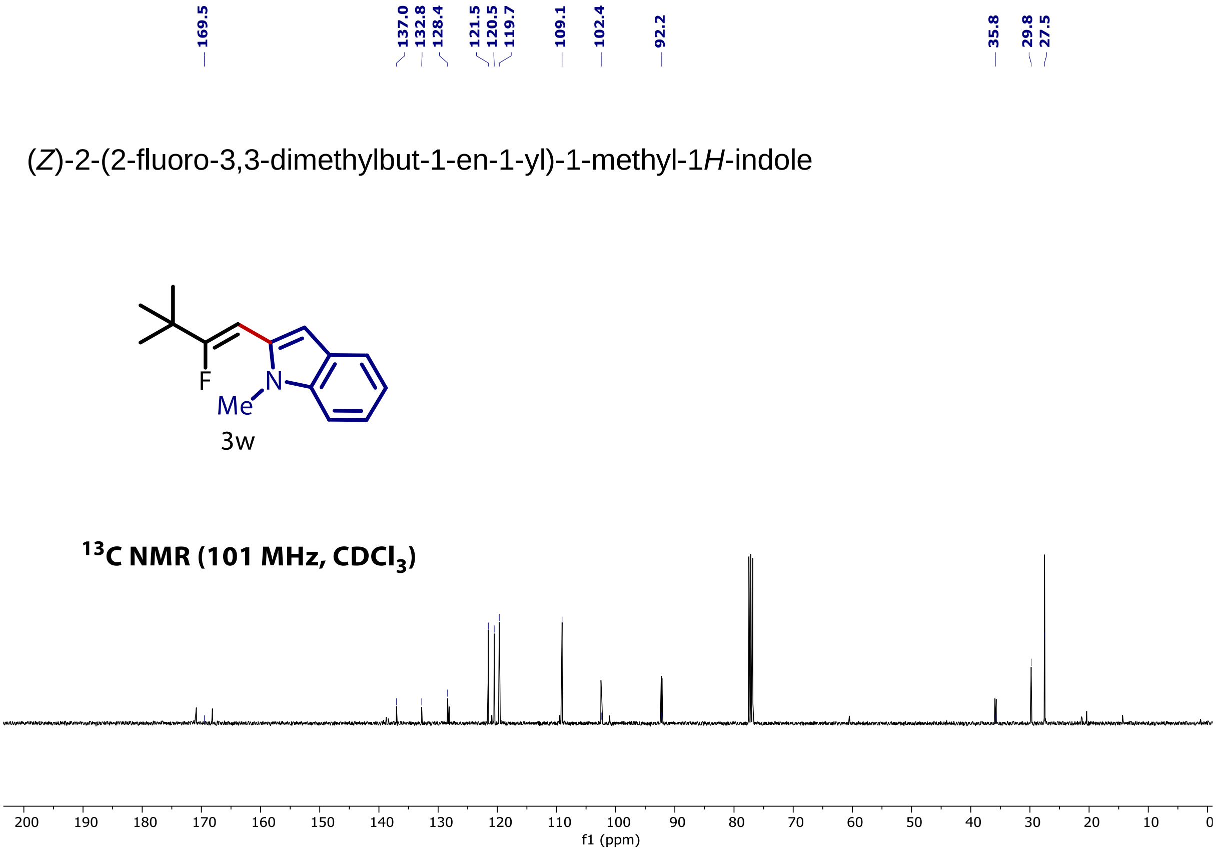

169.5
137.0
132.8
128.4
121.5
120.5
119.7
109.1
102.4
92.2
35.8
29.8
27.5
(Z)-2-(2-fluoro-3,3-dimethylbut-1-en-1-yl)-1-methyl-1H-indole
3w
13C NMR (101 MHz, CDCl3)
200 190 180 170 160 150 140 130 120 110 100 90 80 70 60 50 40 30 20 10 0
f1 (ppm)

(*Z*)-2-(2-fluoro-3,3-dimethylbut-1-en-1-yl)-1-methyl-1*H*-indole

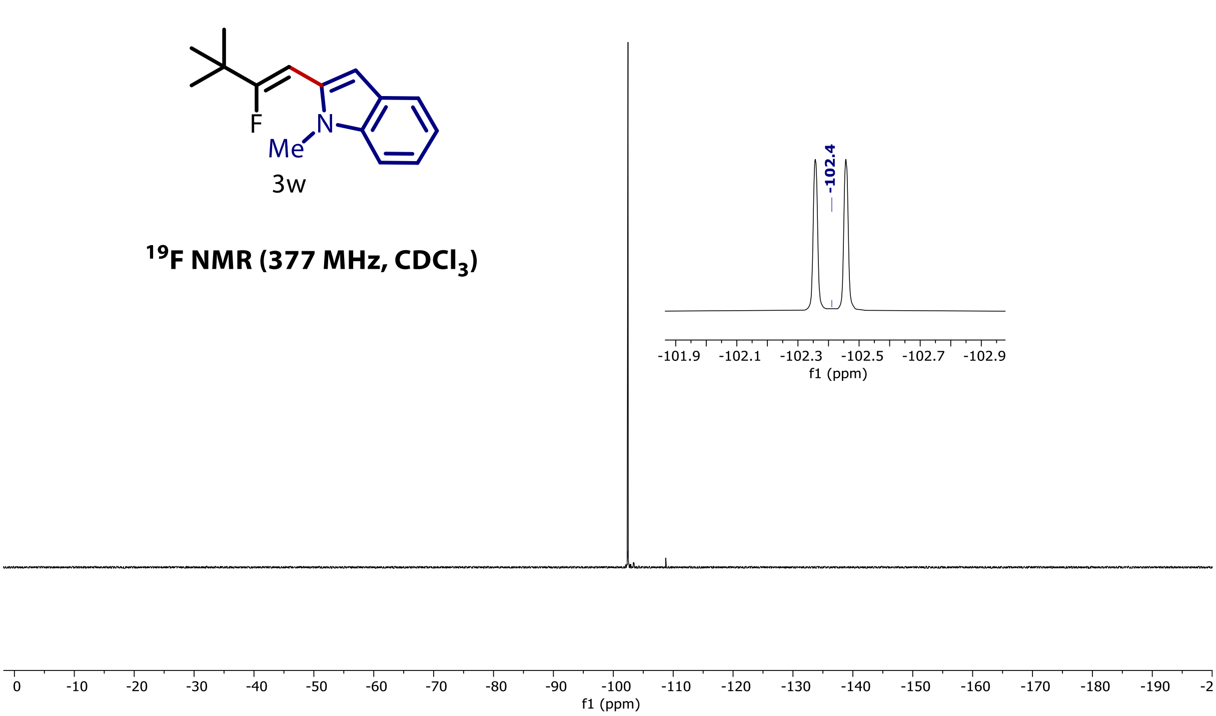

^{19}F NMR (377 MHz, $CDCl_3$)

(*Z*)-2-(2-cyclopropyl-2-fluorovinyl)-1-methyl-1*H*-indole

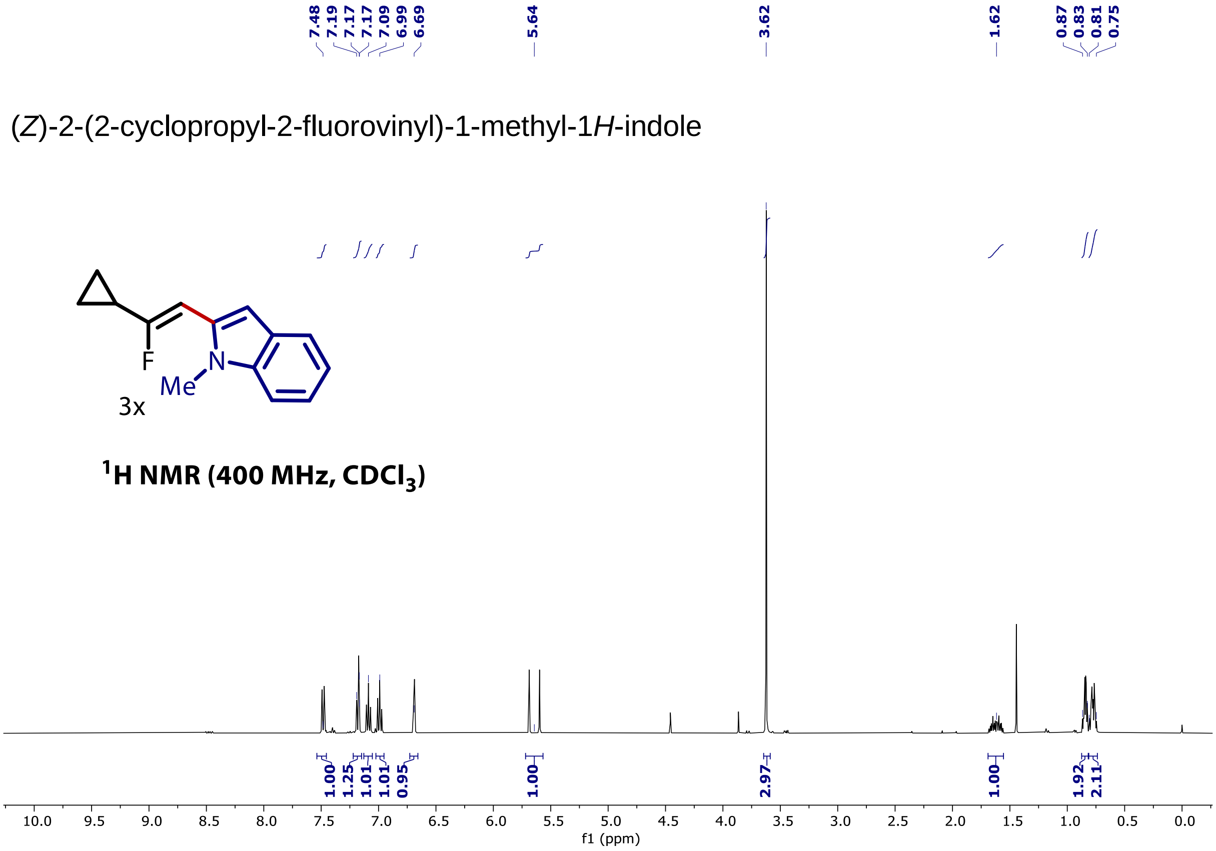

^{1}H NMR (400 MHz, $CDCl_3$)

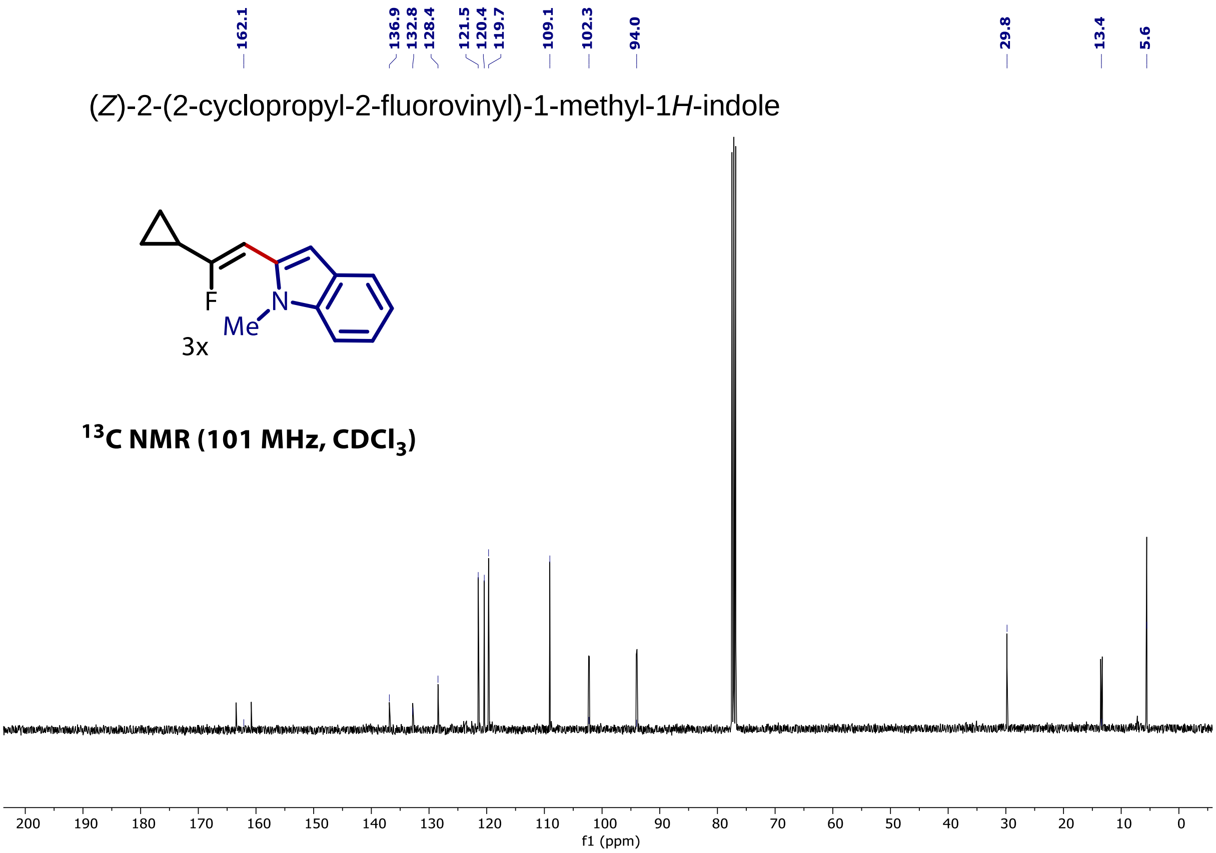
162.1
136.9
132.8
128.4
121.5
120.4
119.7
109.1
102.3
94.0
29.8
13.4
5.6
(Z)-2-(2-cyclopropyl-2-fluorovinyl)-1-methyl-1H-indole
F
N
Me
3x
13C NMR (101 MHz, CDCl3)
200
190
180
170
160
150
140
130
120
110
100
90
80
70
60
50
40
30
20
10
0
f1 (ppm)

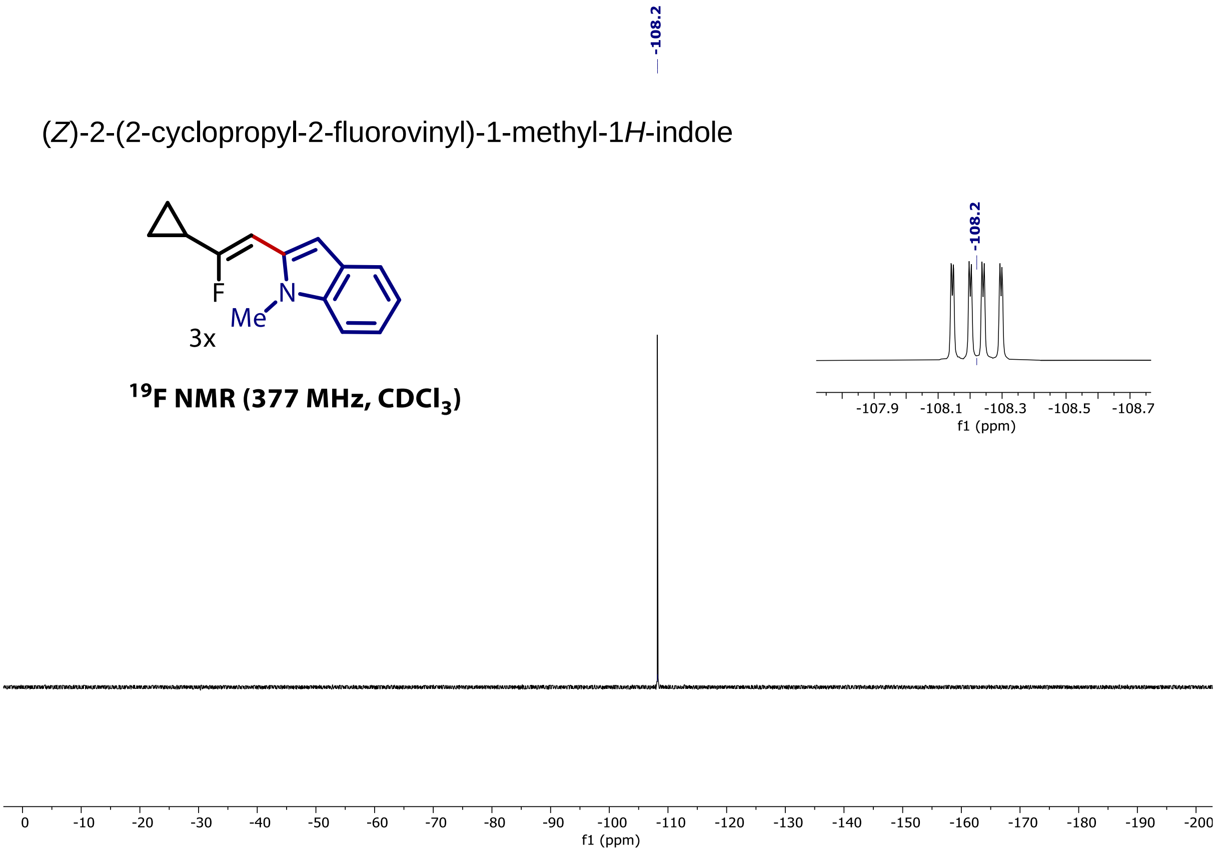
-108.2
(Z)-2-(2-cyclopropyl-2-fluorovinyl)-1-methyl-1H-indole
F
N
Me
3x
19F NMR (377 MHz, CDCl3)
-108.2
-107.9
-108.1
-108.3
-108.5
-108.7
f1 (ppm)
0
-10
-20
-30
-40
-50
-60
-70
-80
-90
-100
-110
-120
-130
-140
-150
-160
-170
-180
-190
-200
f1 (ppm)

(*Z*)-2-(2-cyclohexyl-2-fluorovinyl)-1-methyl-1*H*-indole

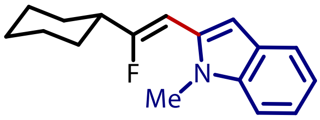

3y

^{1}H NMR (400 MHz, $CDCl_3$)

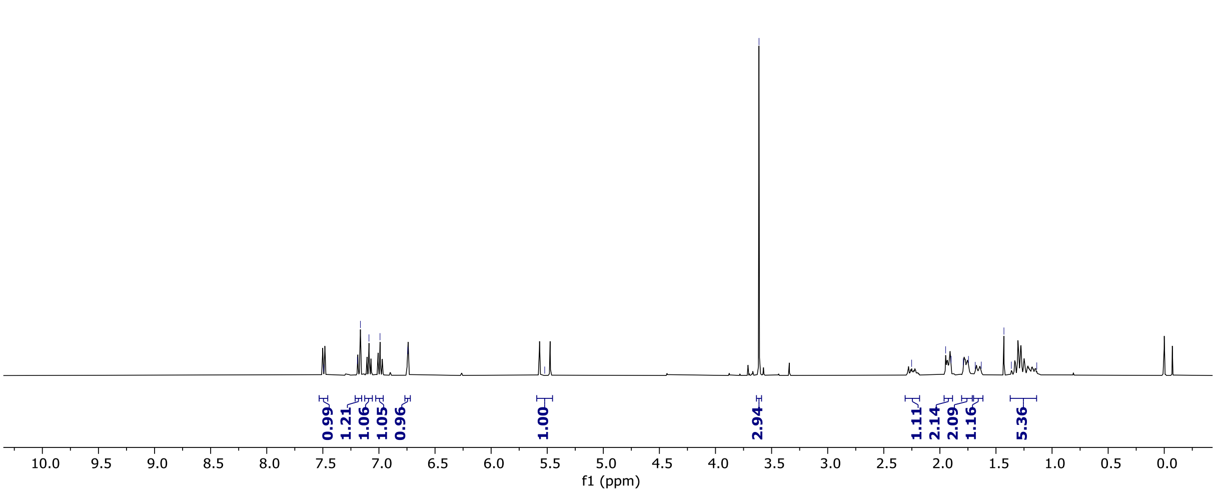

(*Z*)-2-(2-cyclohexyl-2-fluorovinyl)-1-methyl-1*H*-indole

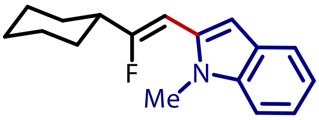

3y

^{13}C NMR (101 MHz, $CDCl_3$)

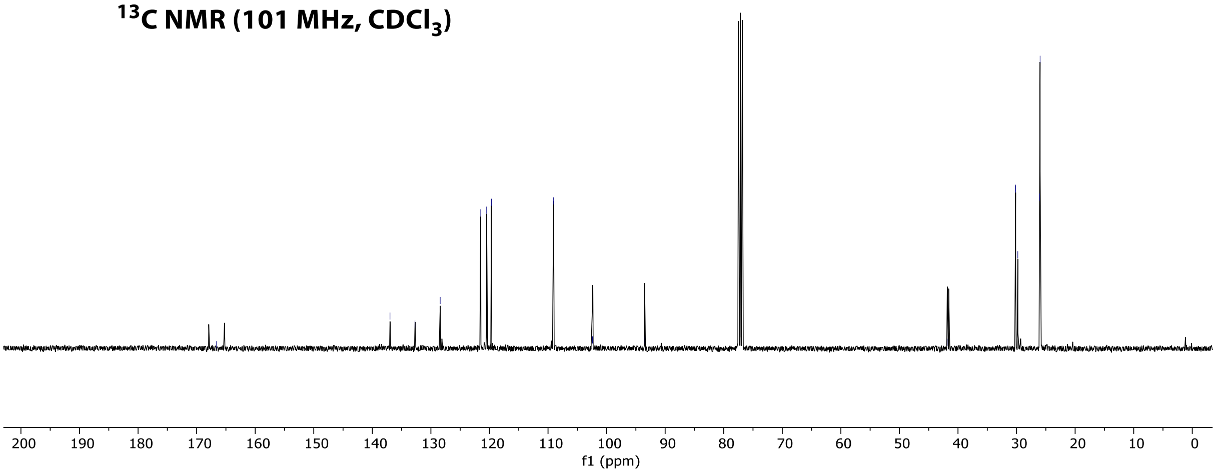

(*Z*)-2-(2-cyclohexyl-2-fluorovinyl)-1-methyl-1*H*-indole

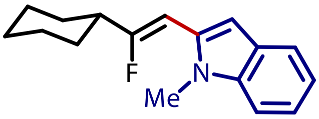

3y

^{19}F NMR (377 MHz, CDCl$_3$)

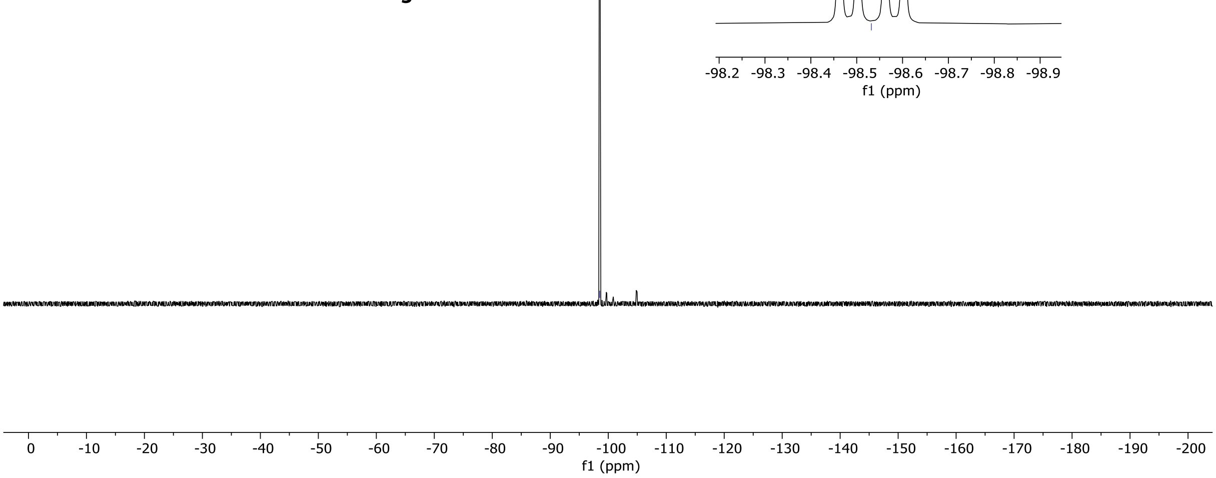

(*Z*)-2-(4-(benzyloxy)-2-fluorobut-1-en-1-yl)-1-methyl-1*H*-indole

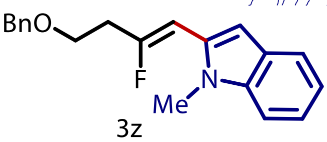

3z

^{1}H NMR (400 MHz, CDCl$_3$)

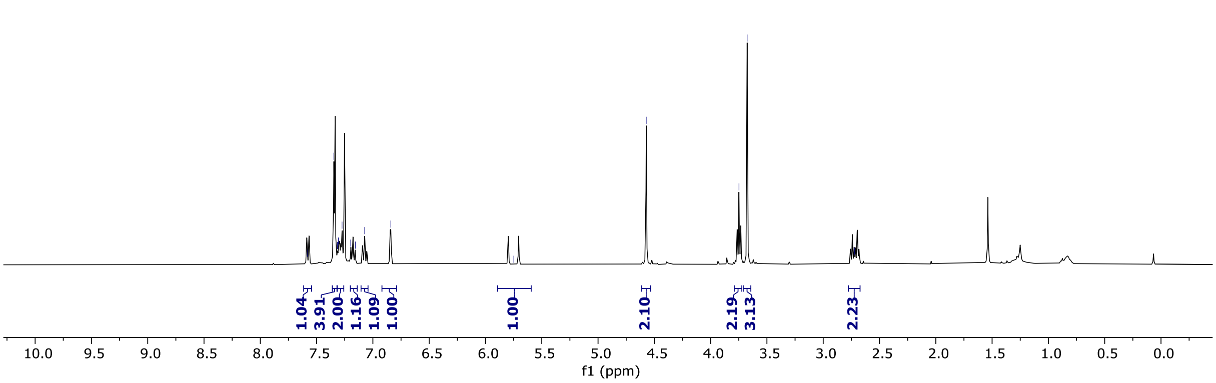

(*Z*)-2-(4-(benzyloxy)-2-fluorobut-1-en-1-yl)-1-methyl-1*H*-indole

3z

^{13}C NMR (101 MHz, $CDCl_3$)

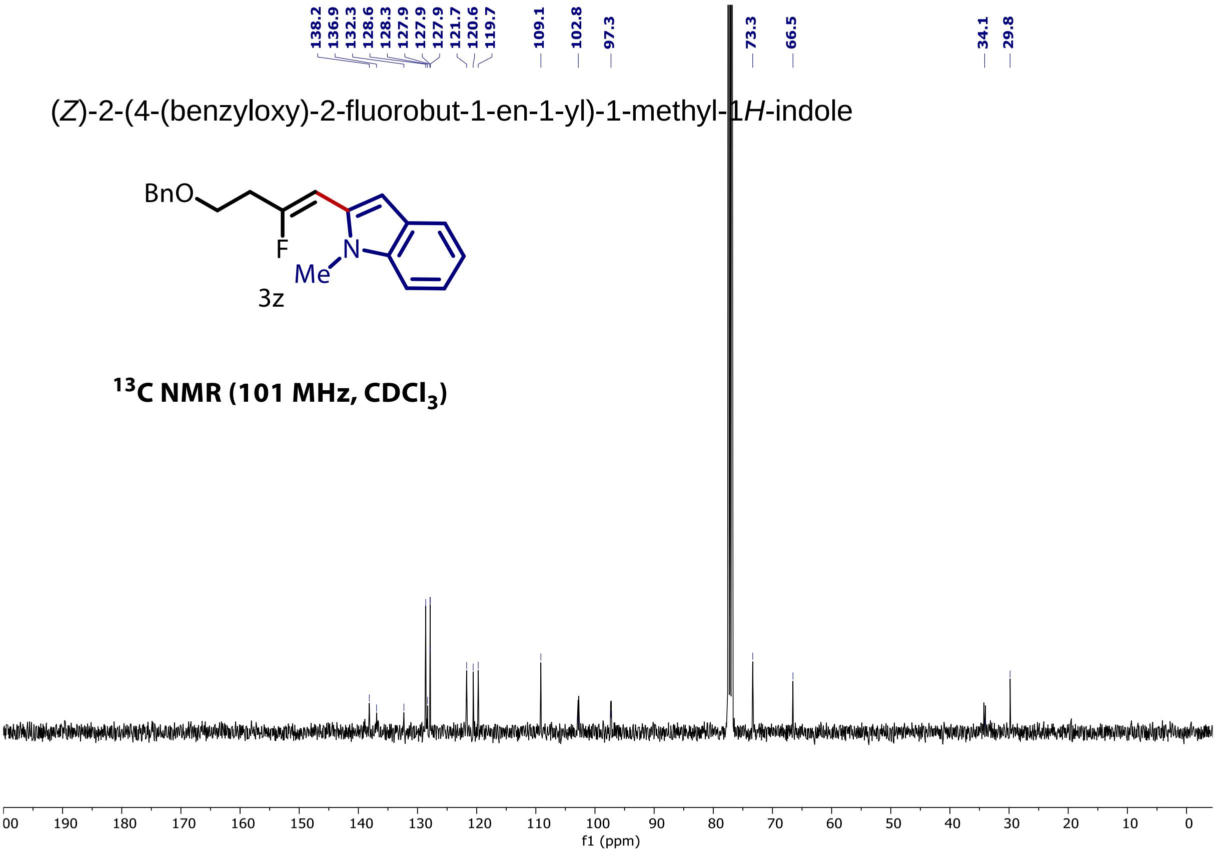

(*Z*)-2-(4-(benzyloxy)-2-fluorobut-1-en-1-yl)-1-methyl-1*H*-indole

3z

^{19}F NMR (377 MHz, $CDCl_3$)

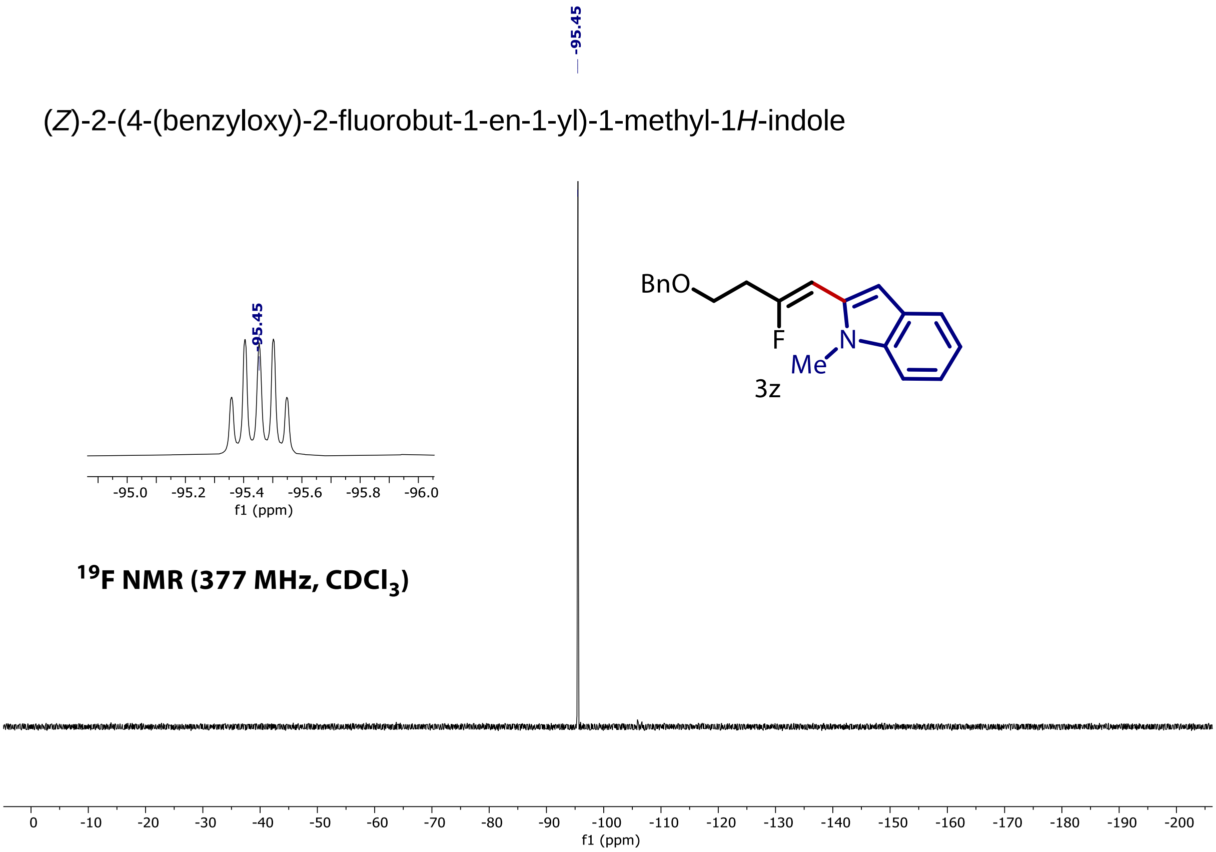

2-((*Z*)-2-fluoro-3-(((1*R*,2*S*,5*R*)-2-isopropyl-5-methylcyclohexyl)oxy)prop-1-en-1-yl)-1-methyl-1*H*-indole

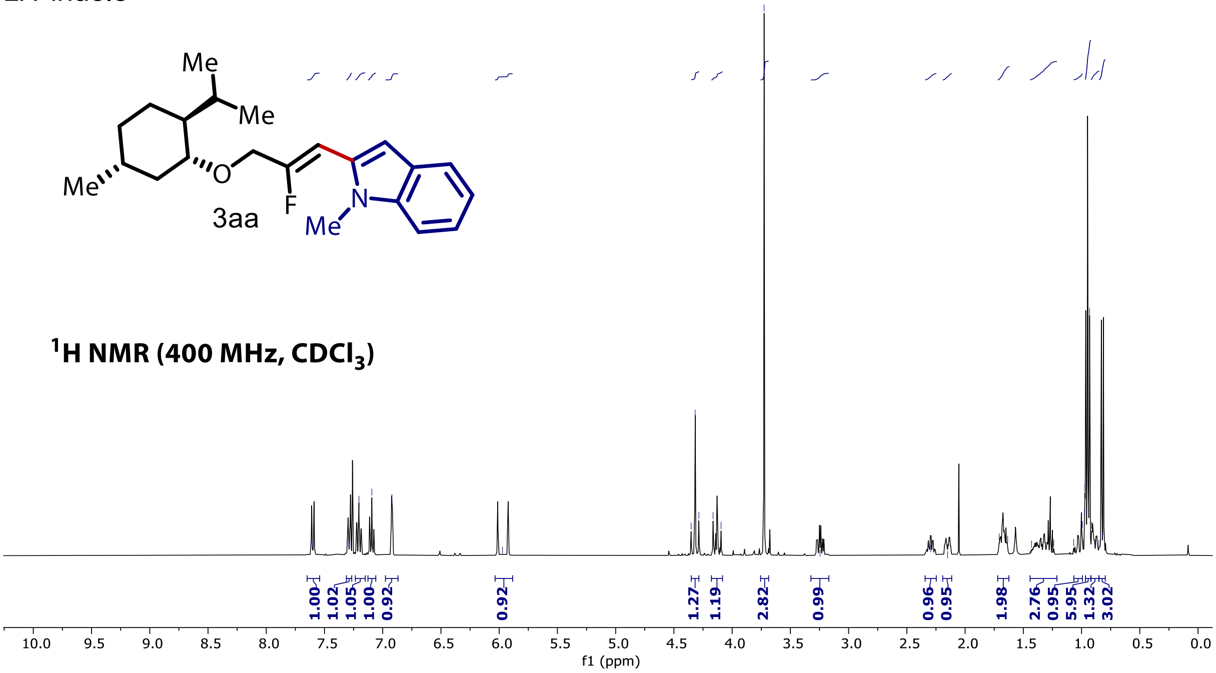

2-((*Z*)-2-fluoro-3-(((1*R*,2*S*,5*R*)-2-isopropyl-5-methylcyclohexyl)oxy)prop-1-en-1-yl)-1-methyl-1*H*-indole

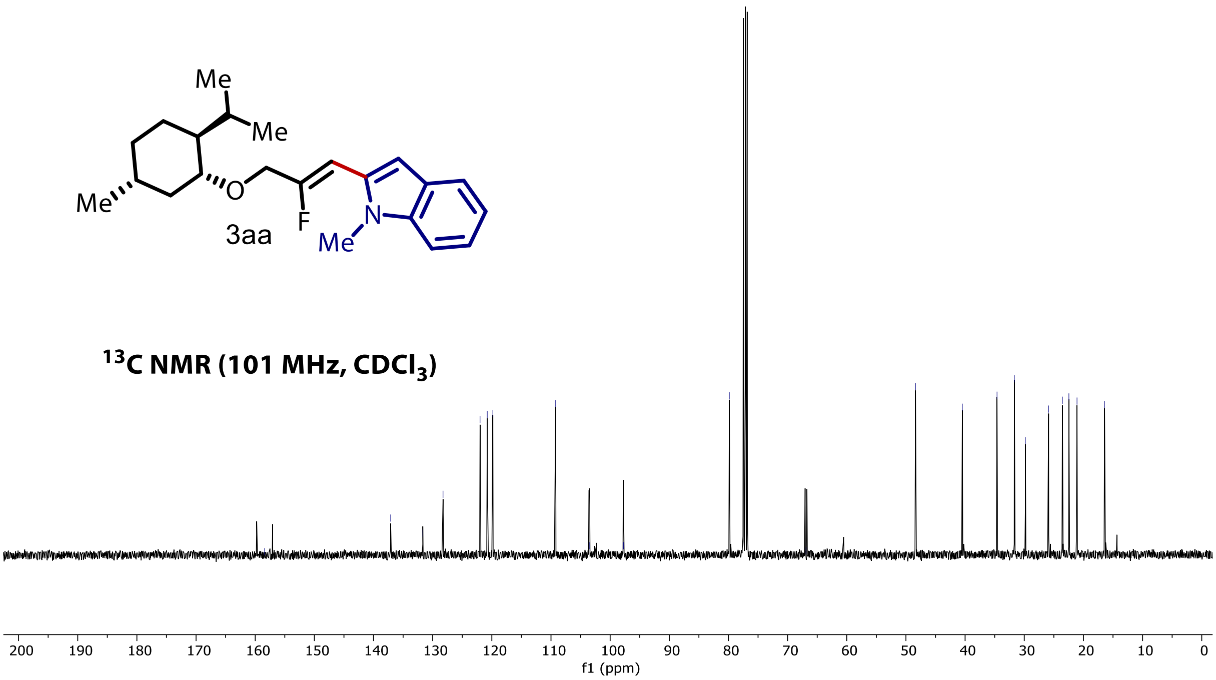

2-((*Z*)-2-fluoro-3-(((1*R*,2*S*,5*R*)-2-isopropyl-5-methylcyclohexyl)oxy)prop-1-en-1-yl)-1-methyl-1*H*-indole

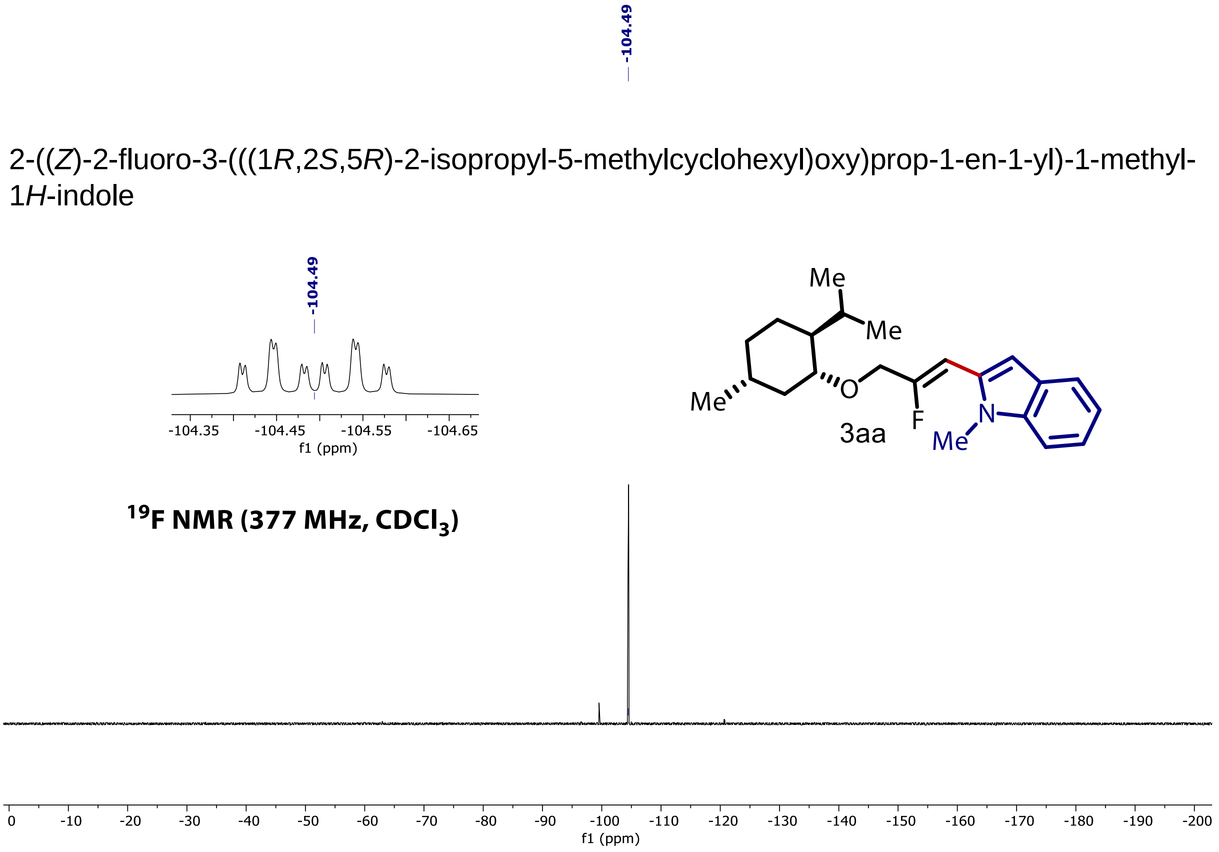

(*Z*)-2-(2-fluoro-3-(1-methyl-1*H*-indol-2-yl)allyl)isoindoline-1,3-dione

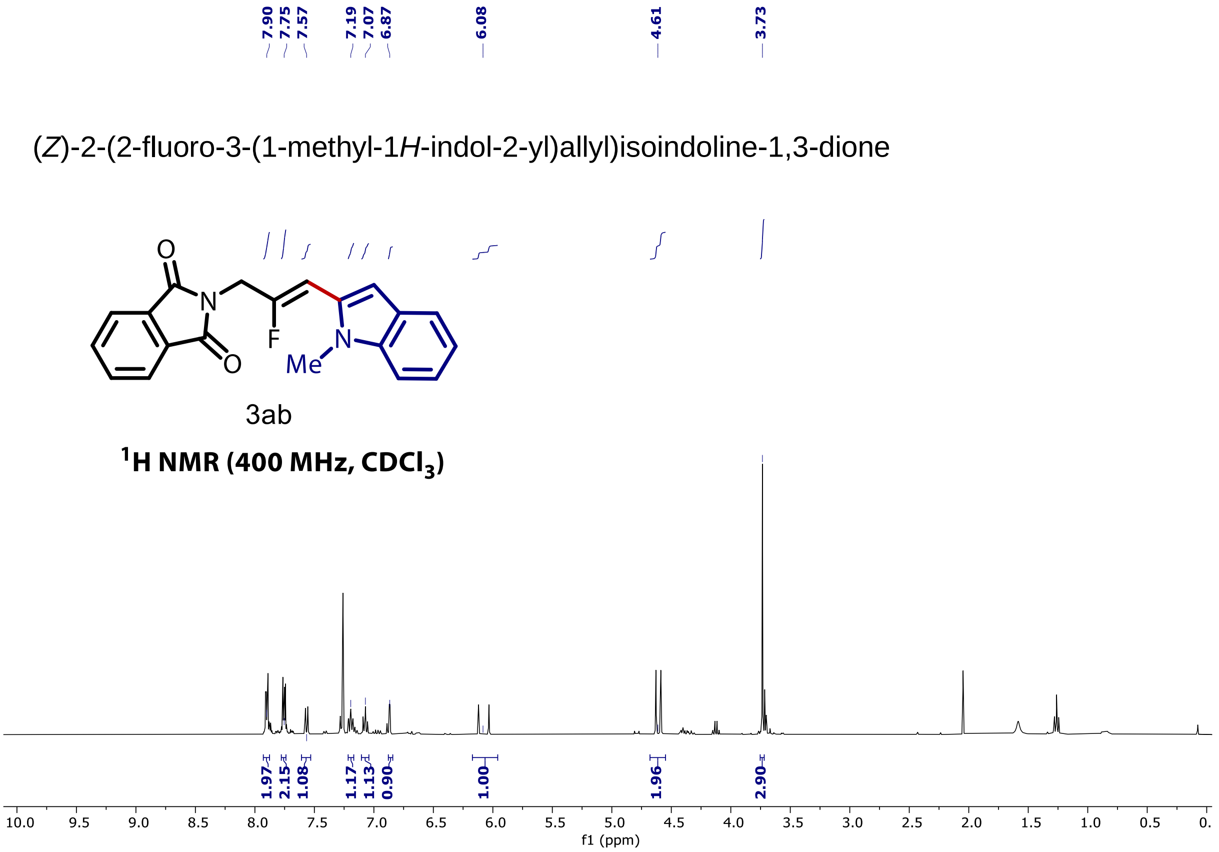

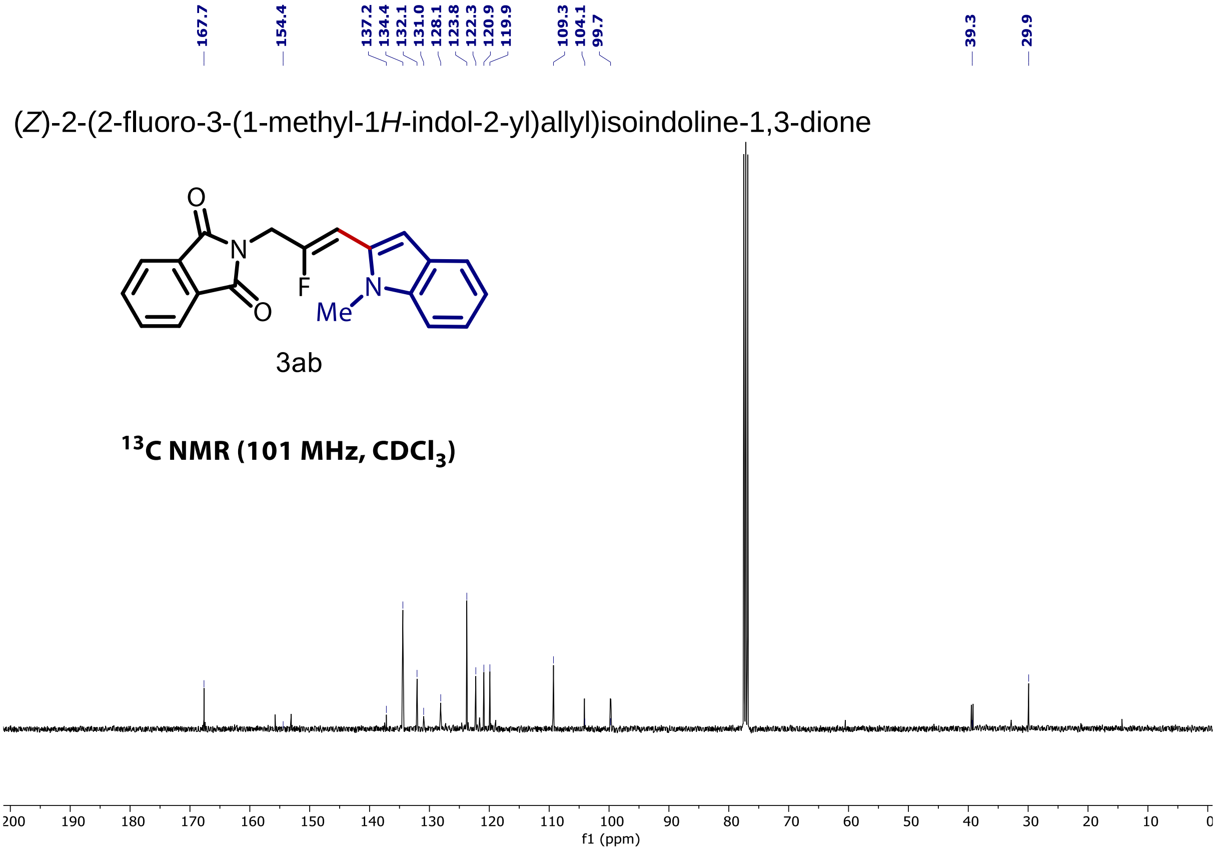

(*Z*)-2-(2-fluoro-3-(1-methyl-1*H*-indol-2-yl)allyl)isoindoline-1,3-dione

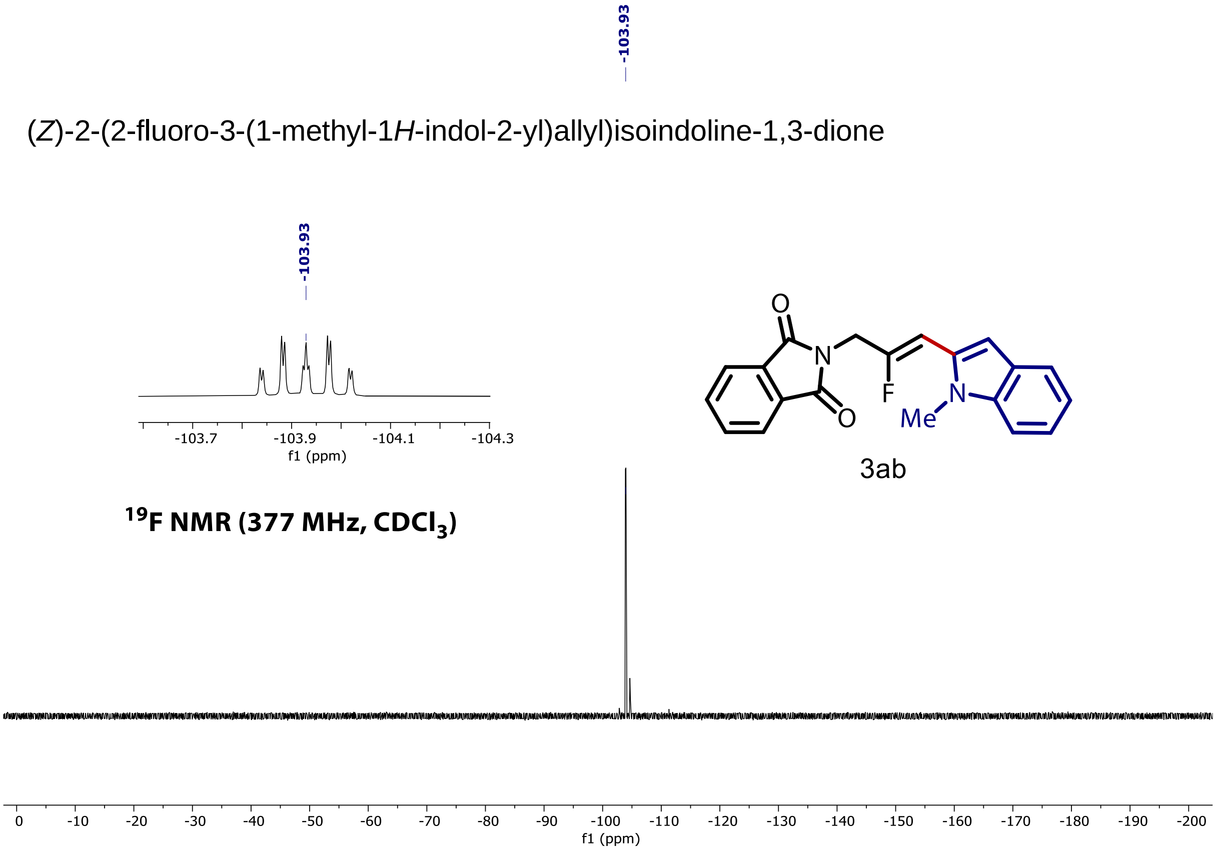

(*Z*)-2-(2-fluoro-3-(1-methyl-1*H*-indol-2-yl)allyl)isoindoline-1,3-dione

(Z)-*N*-(2-fluoro-3-(1-methyl-1*H*-indol-2-yl)allyl)-*N*-(4-fluorobenzyl)-4-nitrobenzenesulfonamide

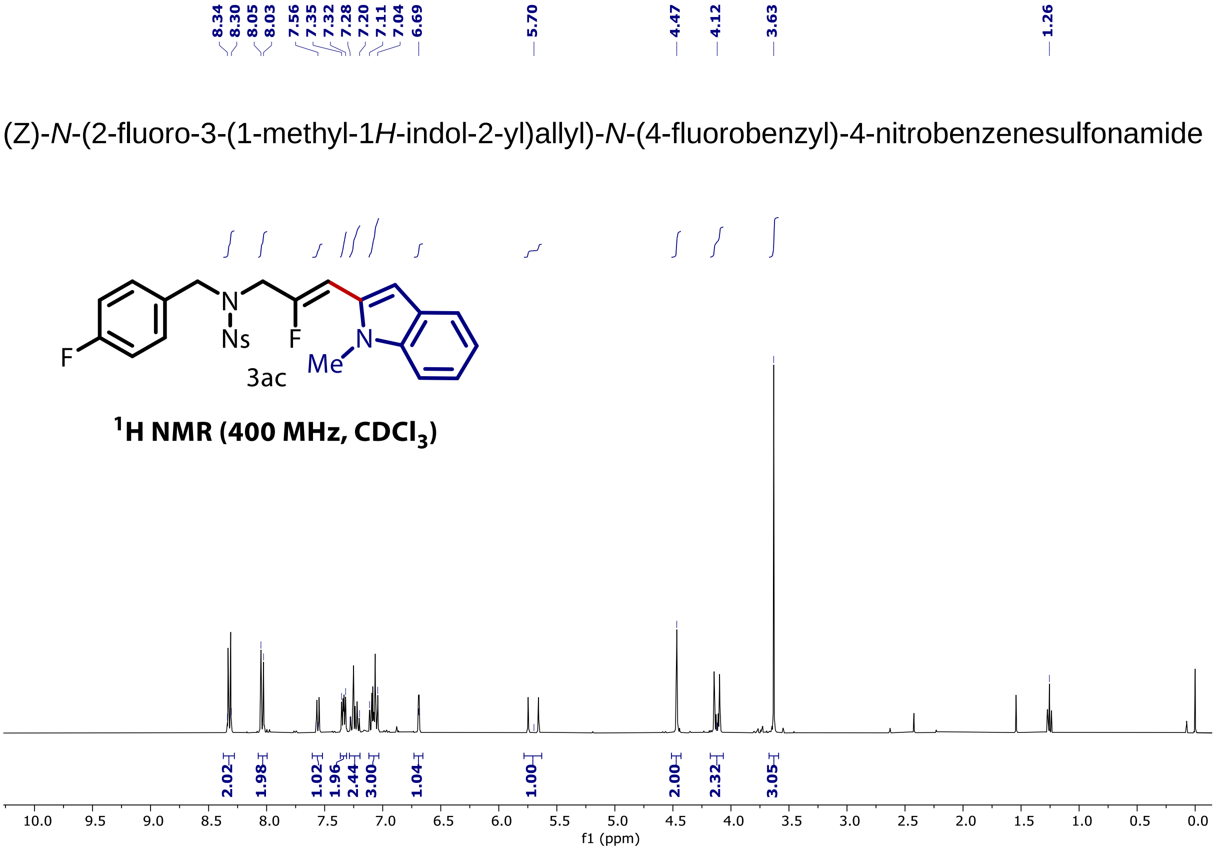

(Z)-*N*-(2-fluoro-3-(1-methyl-1*H*-indol-2-yl)allyl)-*N*-(4-fluorobenzyl)-4-nitrobenzenesulfonamide

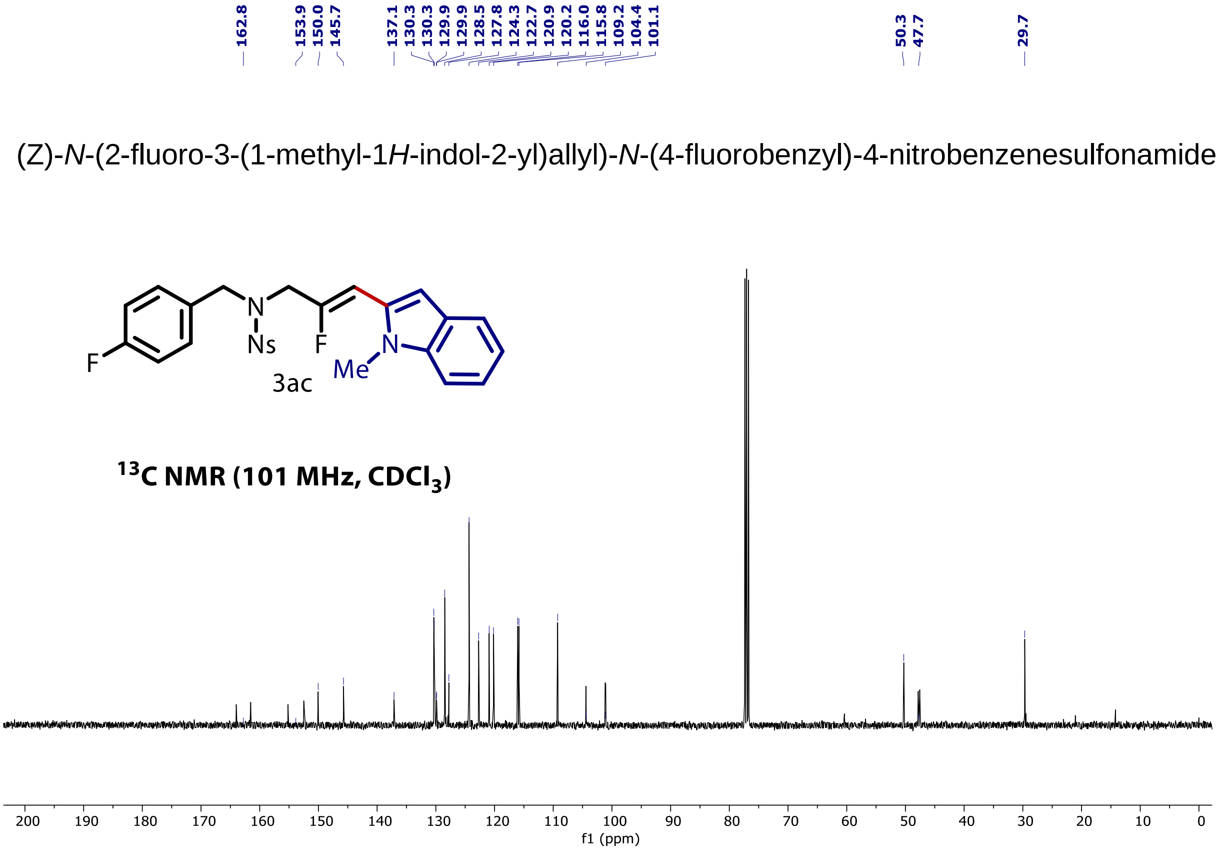

(Z)-*N*-(2-fluoro-3-(1-methyl-1*H*-indol-2-yl)allyl)-*N*-(4-fluorobenzyl)-4-nitrobenzenesulfonamide

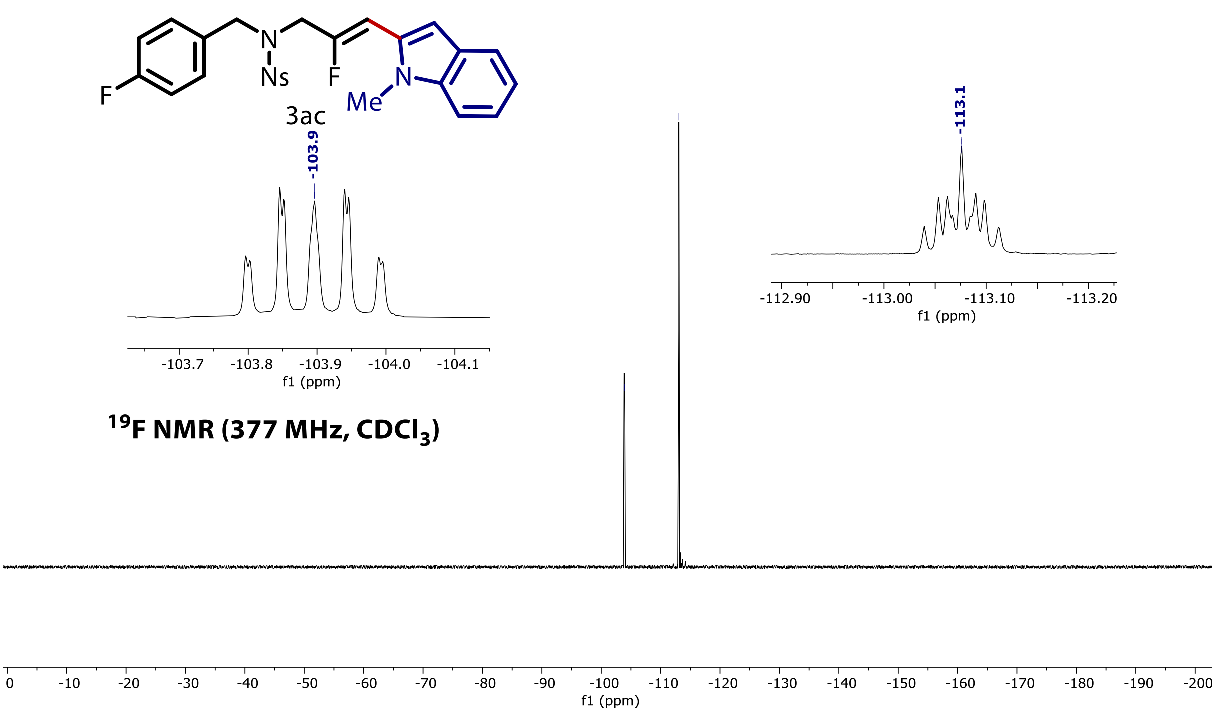

(*Z*)-*N*-(2-fluoro-3-(1-methyl-1*H*-indol-2-yl)allyl)-4-methylbenzenesulfonamide

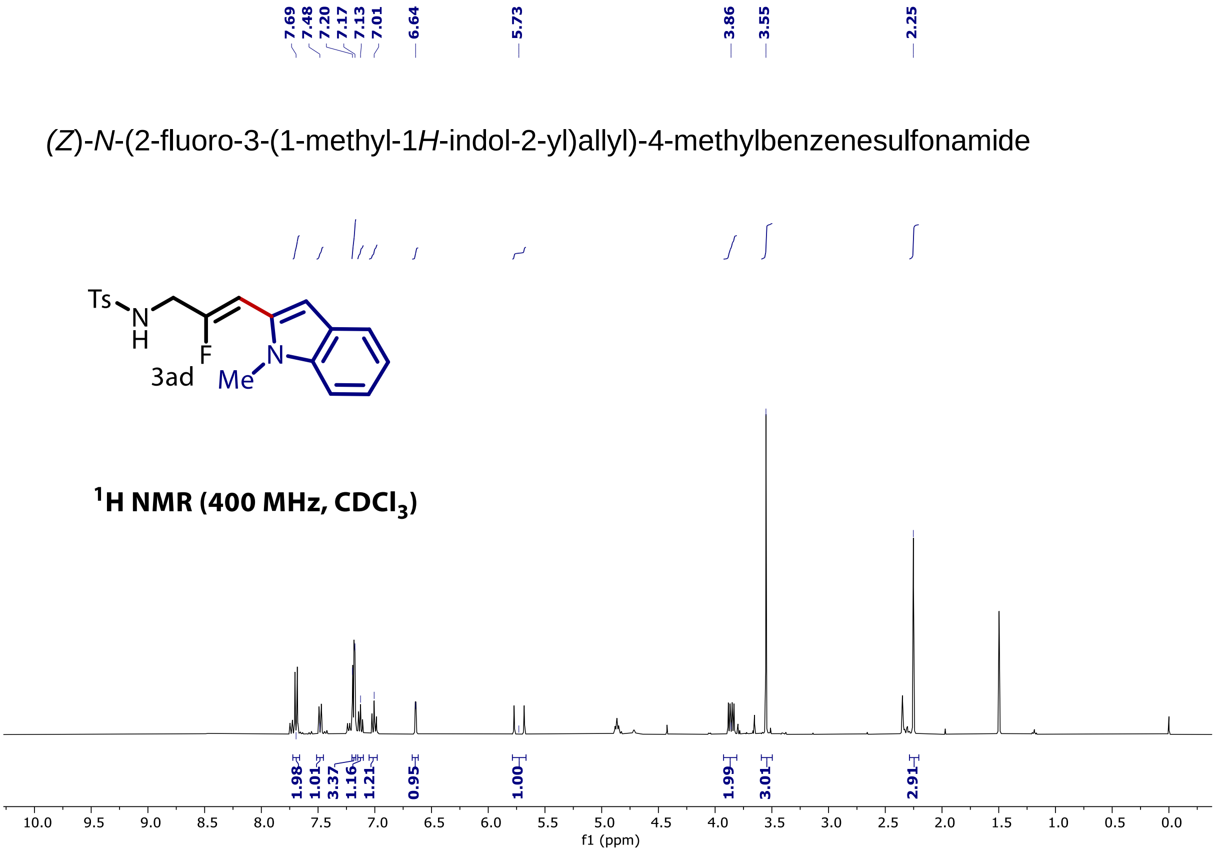

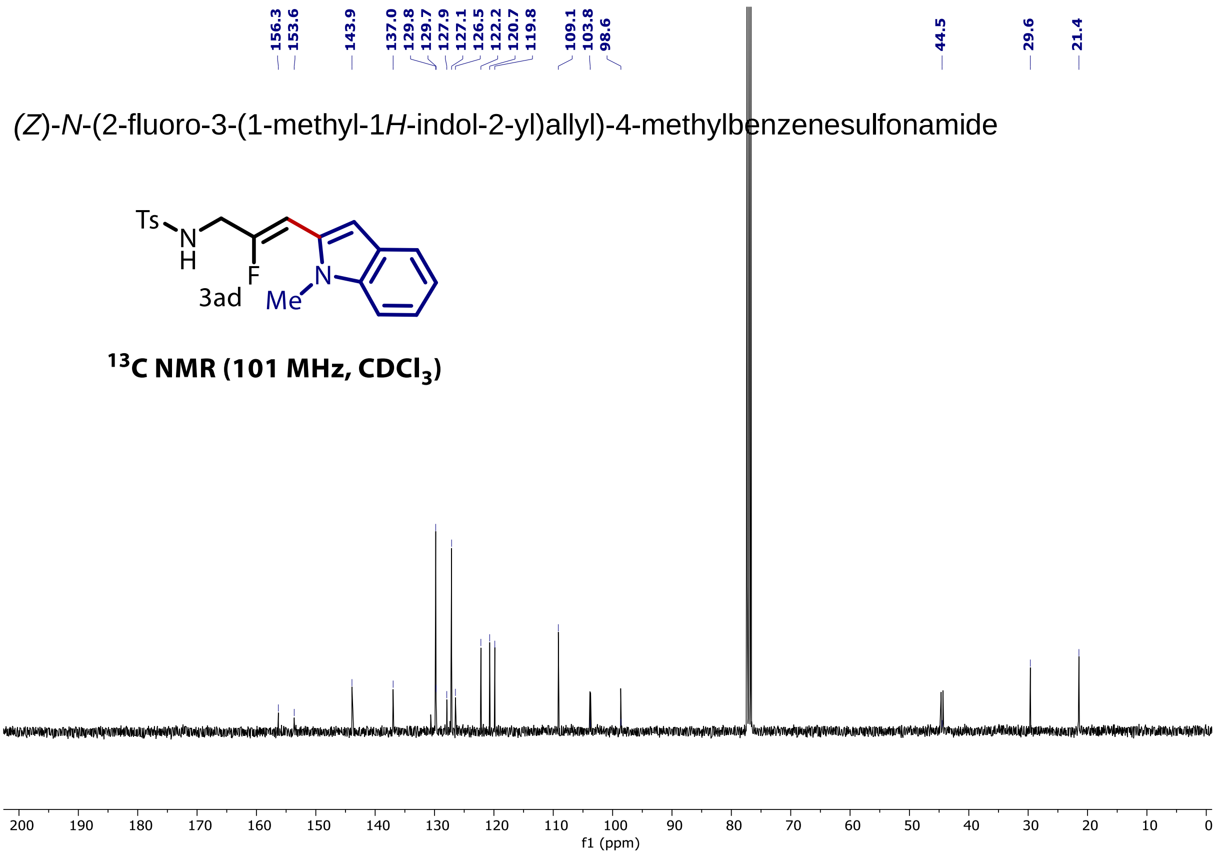
156.3
153.6
143.9
137.0
129.8
129.7
127.9
127.1
126.5
122.2
120.7
119.8
109.1
103.8
98.6
44.5
29.6
21.4
(Z)-N-(2-fluoro-3-(1-methyl-1H-indol-2-yl)allyl)-4-methylbenzenesulfonamide
Ts
N
H
F
3ad
N
Me
13C NMR (101 MHz, CDCl3)
f1 (ppm)

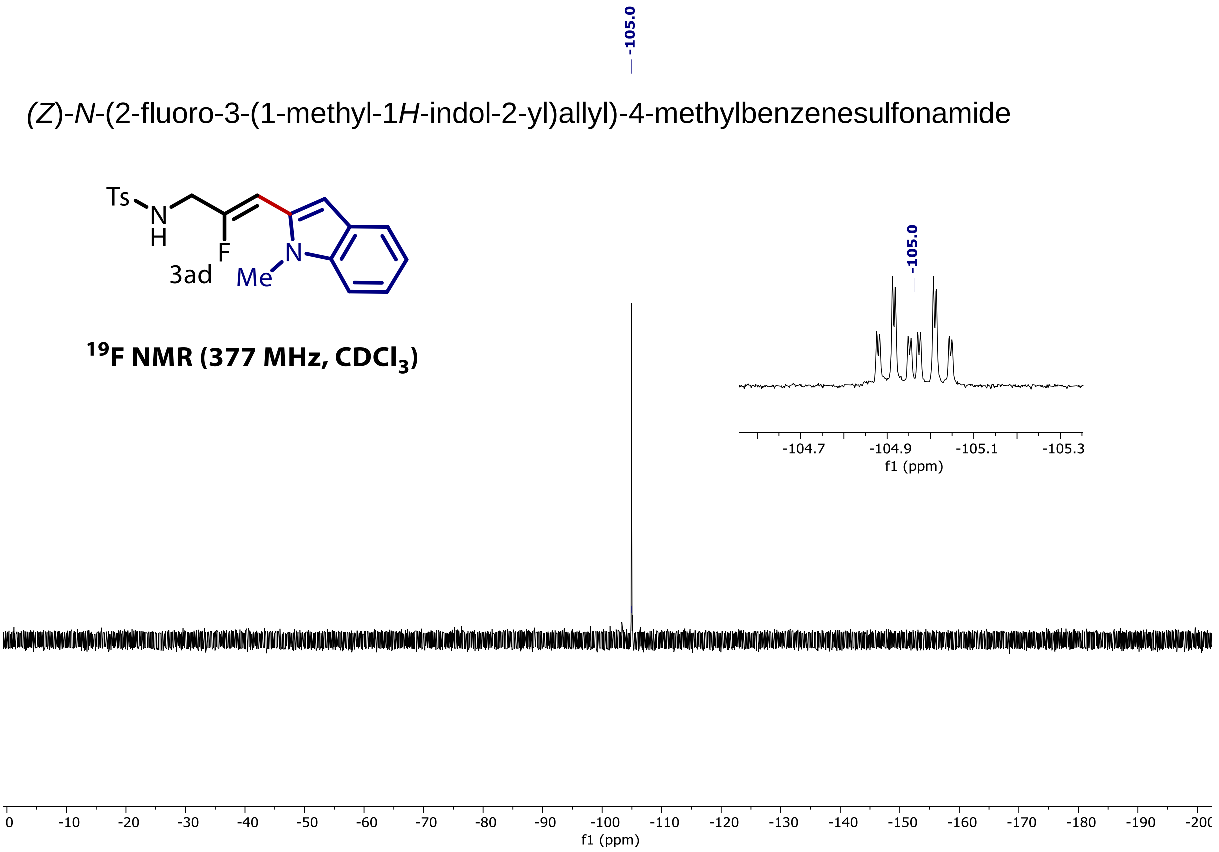
-105.0
(Z)-N-(2-fluoro-3-(1-methyl-1H-indol-2-yl)allyl)-4-methylbenzenesulfonamide
Ts
N
H
F
3ad
N
Me
19F NMR (377 MHz, CDCl3)
-105.0
-104.7
-104.9
-105.1
-105.3
f1 (ppm)
f1 (ppm)

(*Z*)-N-(2-fluoro-3-(1-methyl-1*H*-indol-2-yl)allyl)-*N*-(4-fluorophenyl)-4-methylbenzenesulfonamide

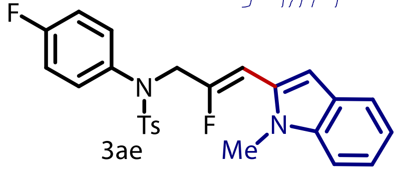

3ae

^{1}H NMR (400 MHz, CDCl$_3$)

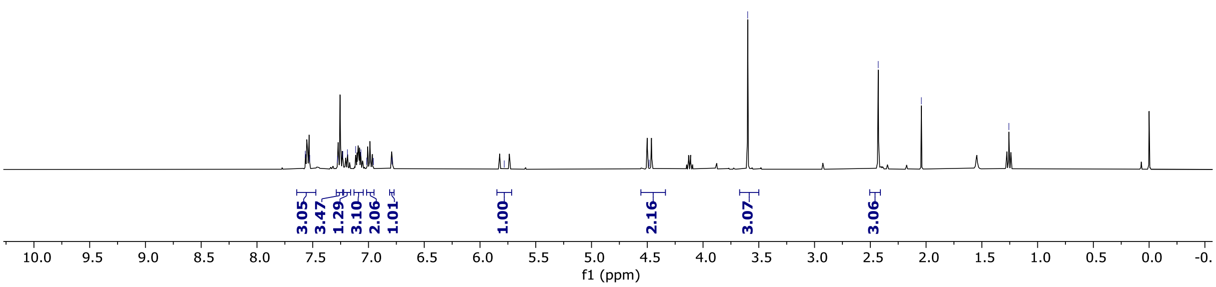

(*Z*)-N-(2-fluoro-3-(1-methyl-1*H*-indol-2-yl)allyl)-*N*-(4-fluorophenyl)-4-methylbenzenesulfonamide

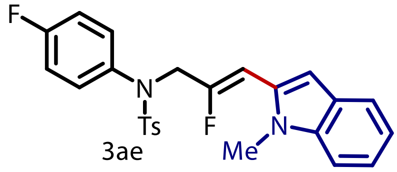

3ae

^{13}C NMR (101 MHz, CDCl$_3$)

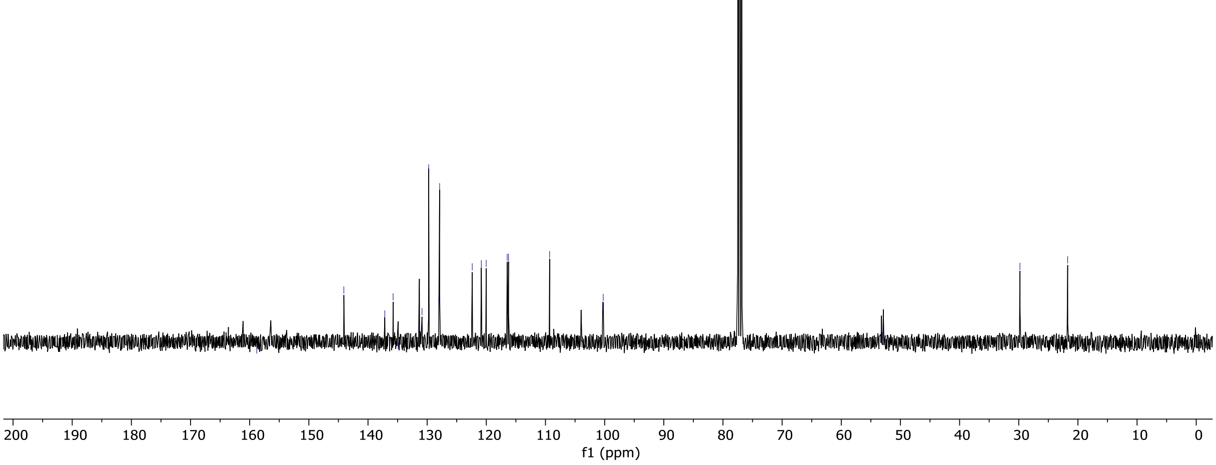

(*Z*)-N-(2-fluoro-3-(1-methyl-1*H*-indol-2-yl)allyl)-*N*-(4-fluorophenyl)-4-methylbenzenesulfonamide

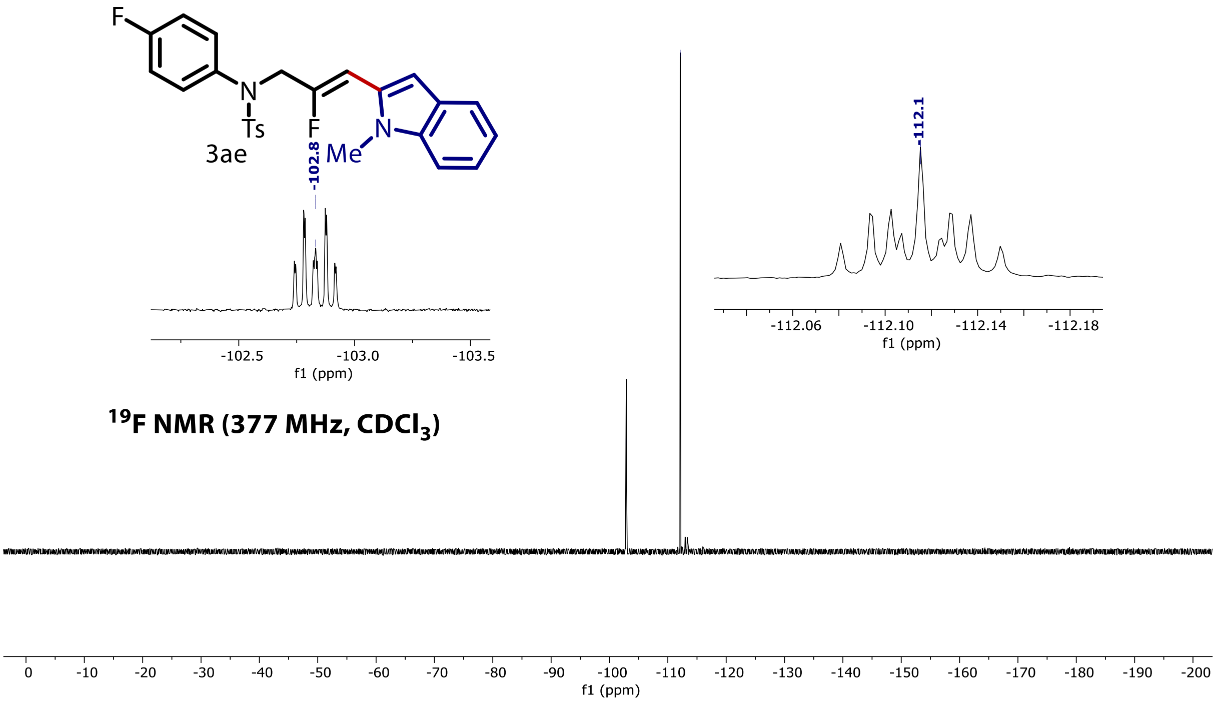

(*Z*)-N-(4-bromophenyl)-*N*-(2-fluoro-3-(1-methyl-1*H*-indol-2-yl)allyl)-4-methylbenzenesulfonamide

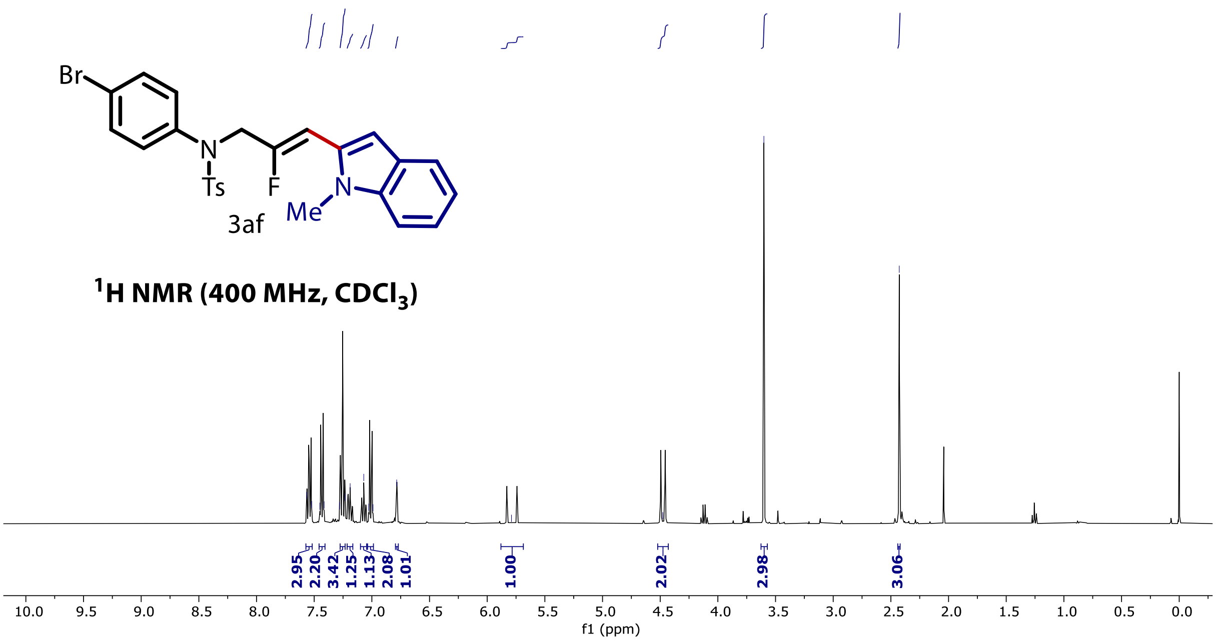

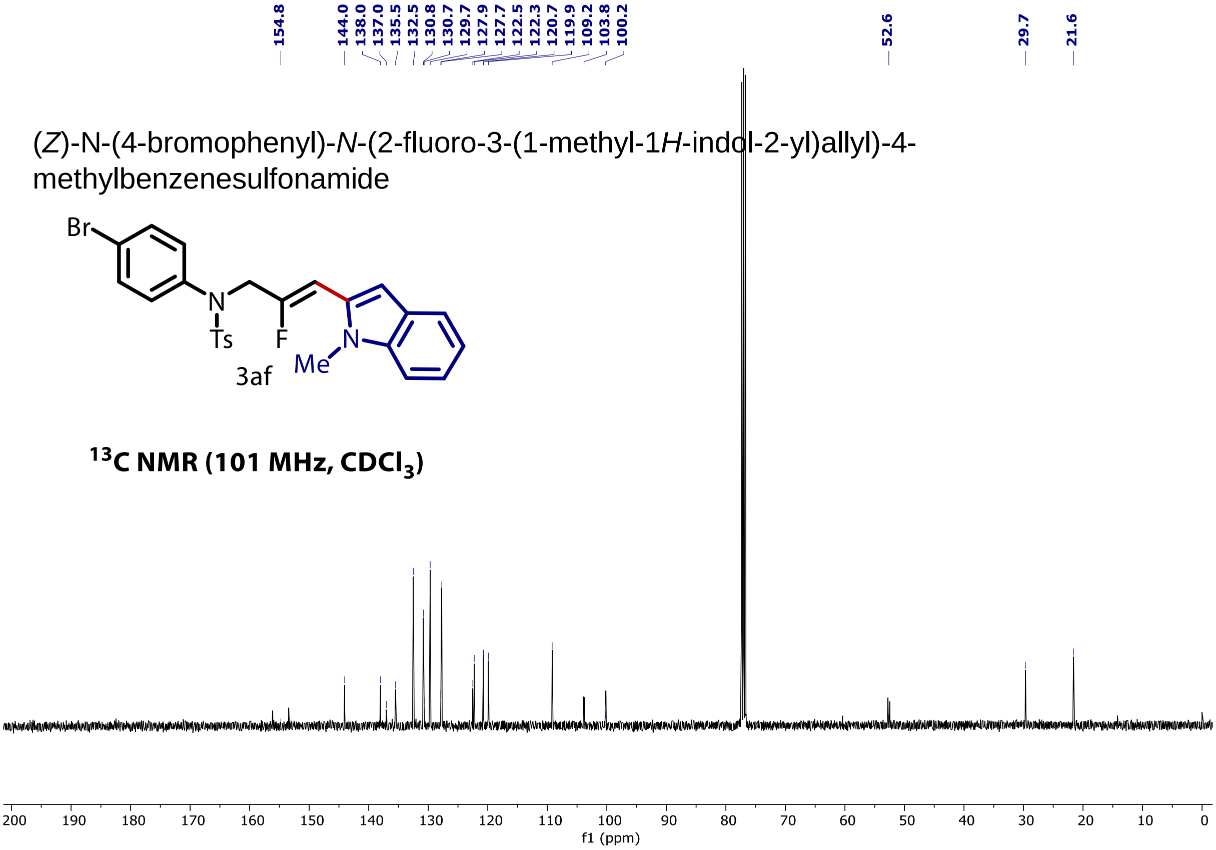
154.8
144.0
138.0
137.0
135.5
132.5
130.8
130.7
129.7
127.9
127.7
122.5
122.3
120.7
119.9
109.2
103.8
100.2
52.6
29.7
21.6
(Z)-N-(4-bromophenyl)-N-(2-fluoro-3-(1-methyl-1H-indol-2-yl)allyl)-4-methylbenzenesulfonamide
Br
N
Ts
F
N
Me
3af
13C NMR (101 MHz, CDCl3)
200 190 180 170 160 150 140 130 120 110 100 90 80 70 60 50 40 30 20 10 0
f1 (ppm)

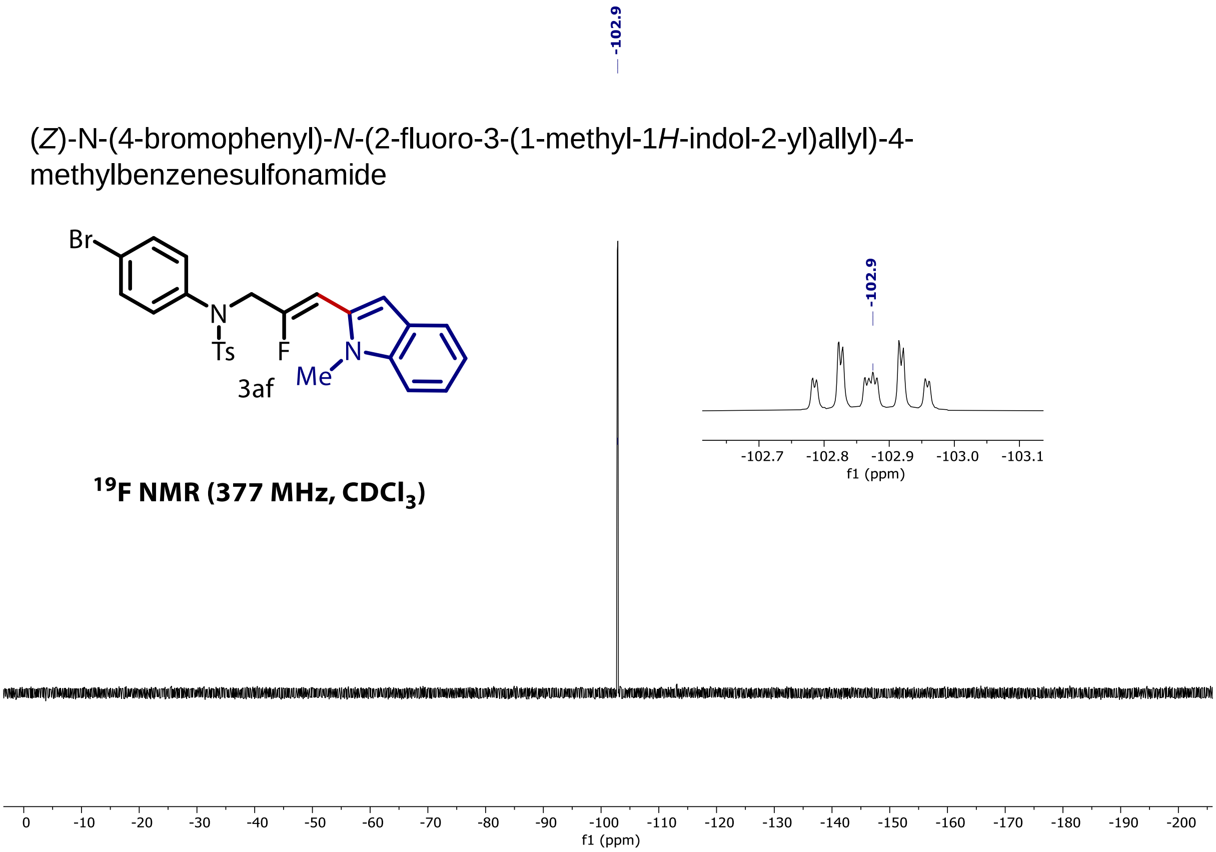
-102.9
(Z)-N-(4-bromophenyl)-N-(2-fluoro-3-(1-methyl-1H-indol-2-yl)allyl)-4-methylbenzenesulfonamide
Br
N
Ts
F
N
Me
3af
19F NMR (377 MHz, CDCl3)
-102.9
-102.7 -102.8 -102.9 -103.0 -103.1
f1 (ppm)
0 -10 -20 -30 -40 -50 -60 -70 -80 -90 -100 -110 -120 -130 -140 -150 -160 -170 -180 -190 -200
f1 (ppm)

(*Z*)-*N*-cyclopropyl-*N*-(2-fluoro-3-(1-methyl-1*H*-indol-2-yl)allyl)-4-methylbenzenesulfonamide

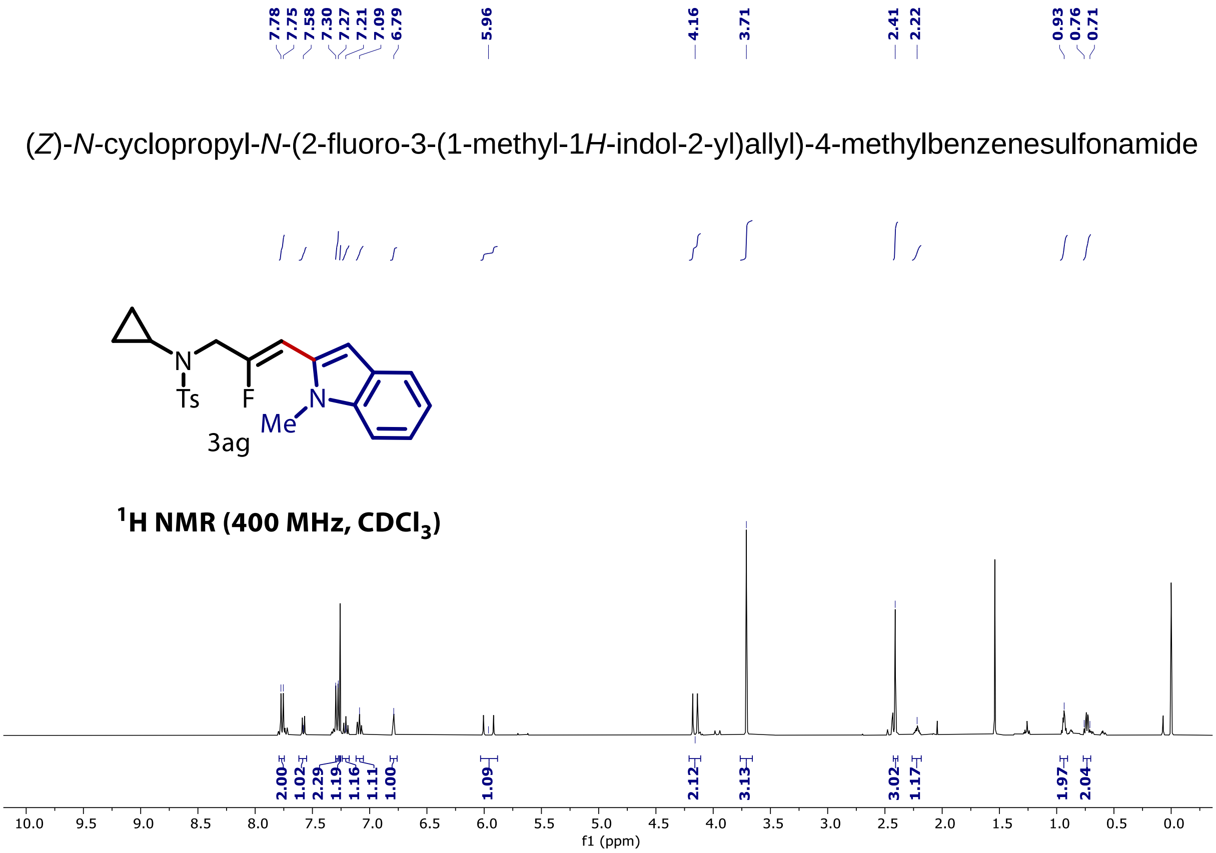

^{1}H NMR (400 MHz, $CDCl_3$)

(*Z*)-*N*-cyclopropyl-*N*-(2-fluoro-3-(1-methyl-1*H*-indol-2-yl)allyl)-4-methylbenzenesulfonamide

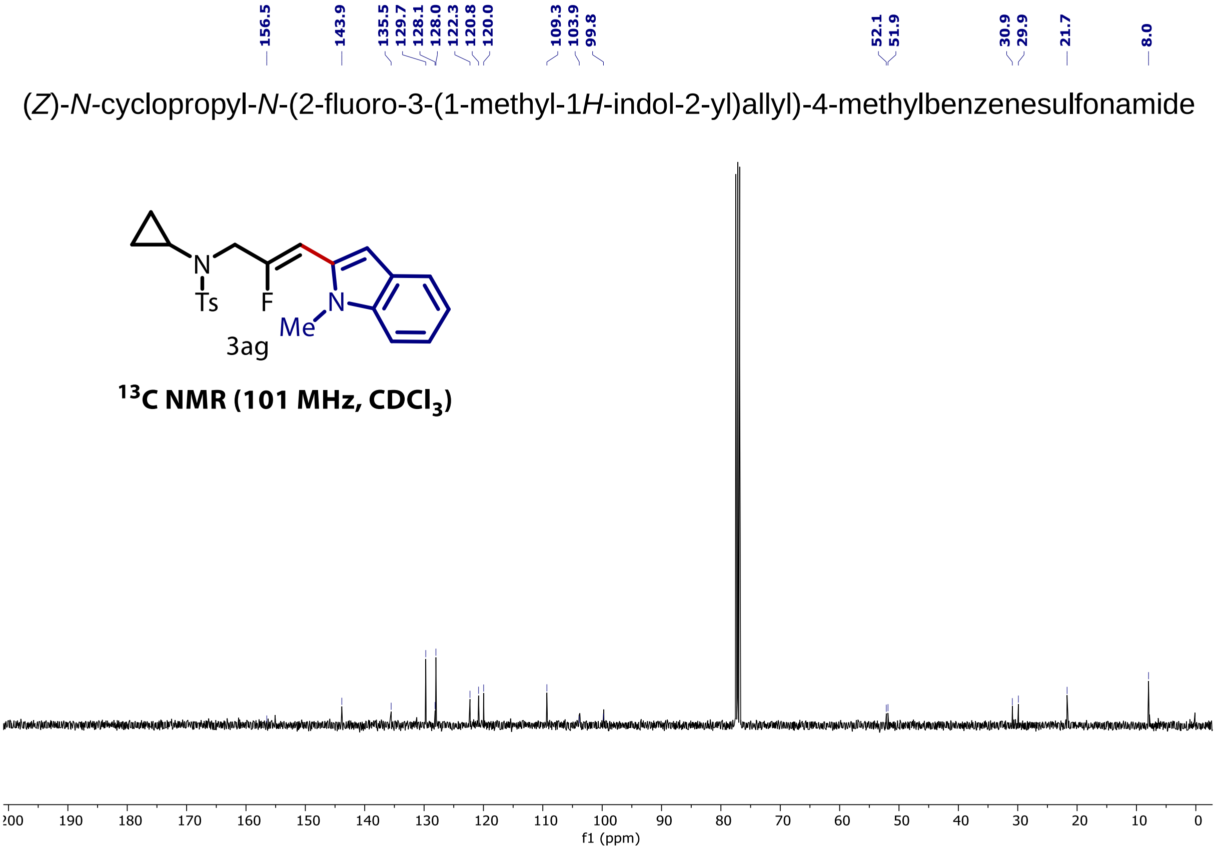

^{13}C NMR (101 MHz, $CDCl_3$)

(*Z*)-*N*-cyclopropyl-*N*-(2-fluoro-3-(1-methyl-1*H*-indol-2-yl)allyl)-4-methylbenzenesulfonamide

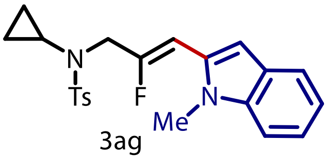

3ag

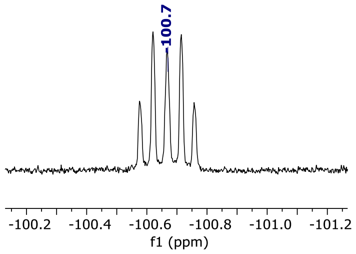

^{19}F NMR (377 MHz, CDCl$_3$)

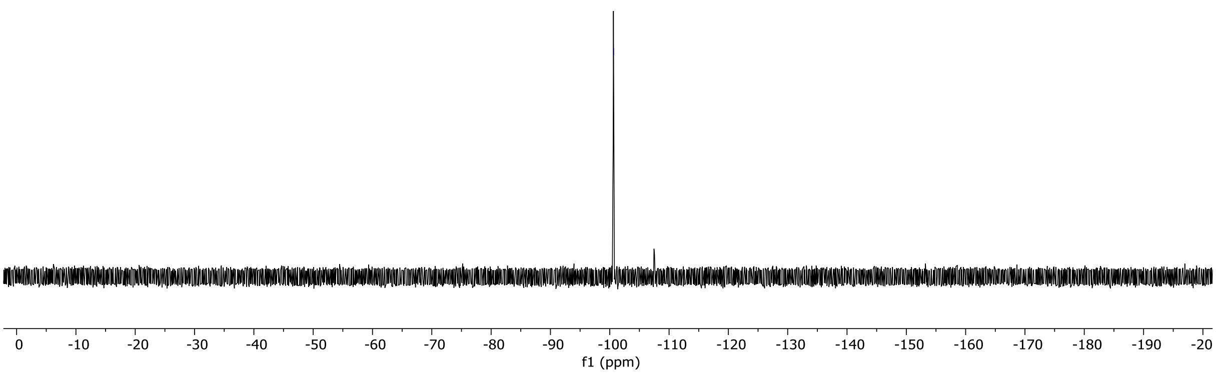

(*Z*)-3-fluoro-4-(1-methyl-1*H*-indol-2-yl)but-3-en-2-one

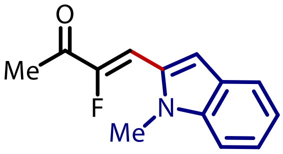

3ah

^{1}H NMR (400 MHz, CDCl$_3$)

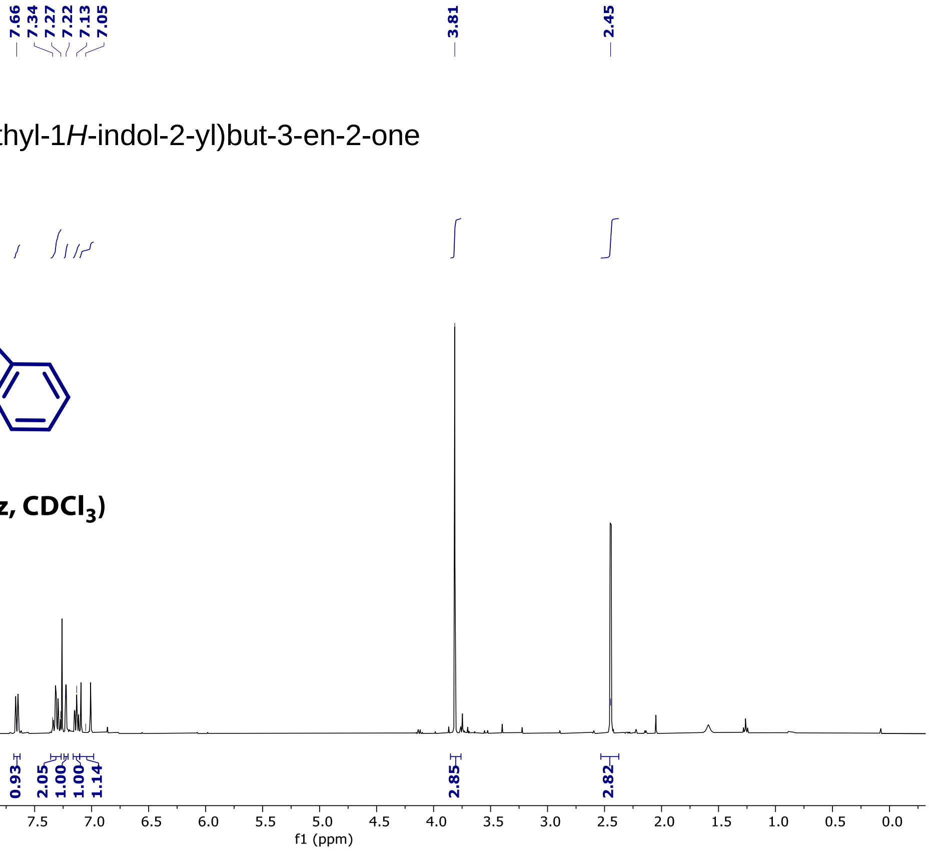

(*Z*)-3-fluoro-4-(1-methyl-1*H*-indol-2-yl)but-3-en-2-one

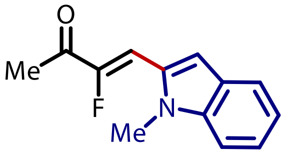

3ah

^{13}C NMR (101 MHz, $CDCl_3$)

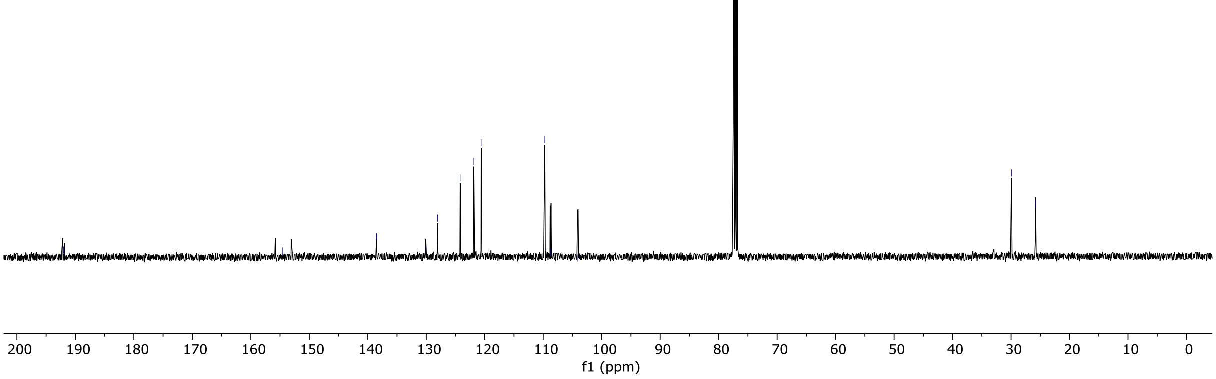

(*Z*)-3-fluoro-4-(1-methyl-1*H*-indol-2-yl)but-3-en-2-one

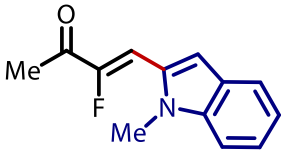

3ah

^{19}F NMR (377 MHz, $CDCl_3$)

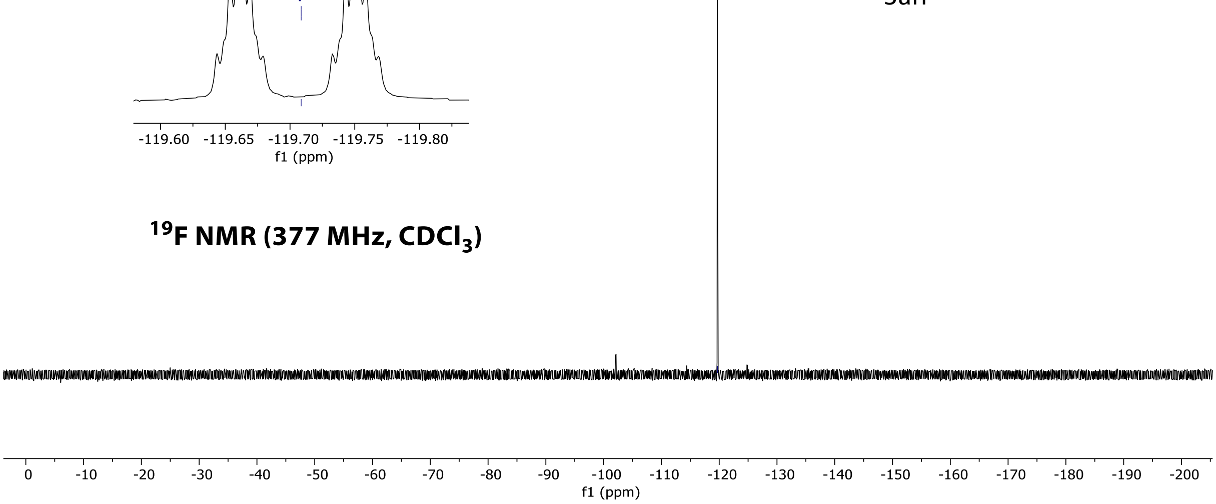

Ethyl (*z*)-2-fluoro-3-(1-methyl-1*H*-indol-2-yl)acrylate

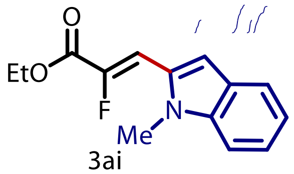

^{1}H NMR (400 MHz, $CDCl_3$)

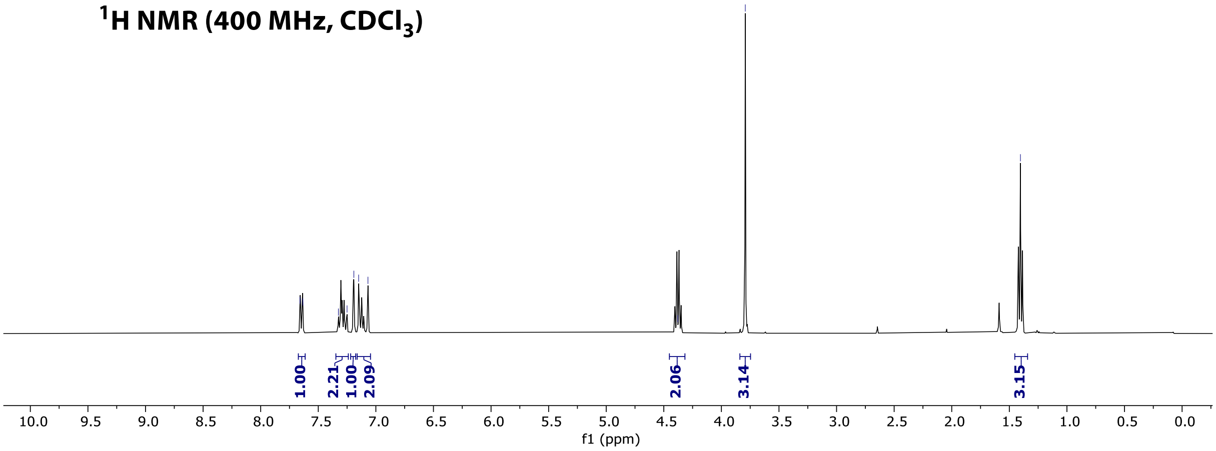

Ethyl (*z*)-2-fluoro-3-(1-methyl-1*H*-indol-2-yl)acrylate

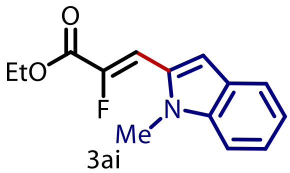

^{13}C NMR (101 MHz, $CDCl_3$)

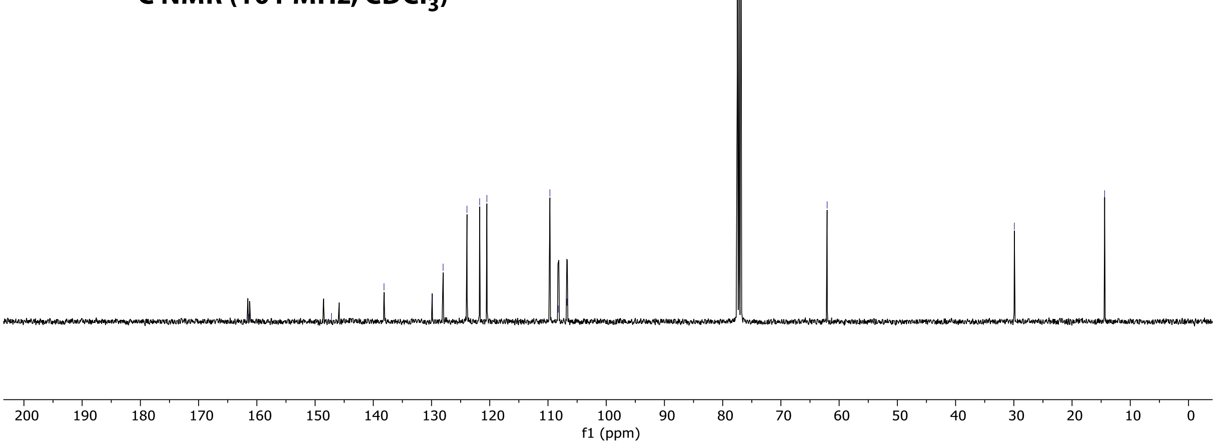

Ethyl (*z*)-2-fluoro-3-(1-methyl-1*H*-indol-2-yl)acrylate

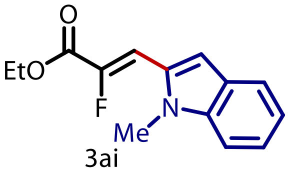

3ai

^{19}F NMR (377 MHz, $CDCl_3$)

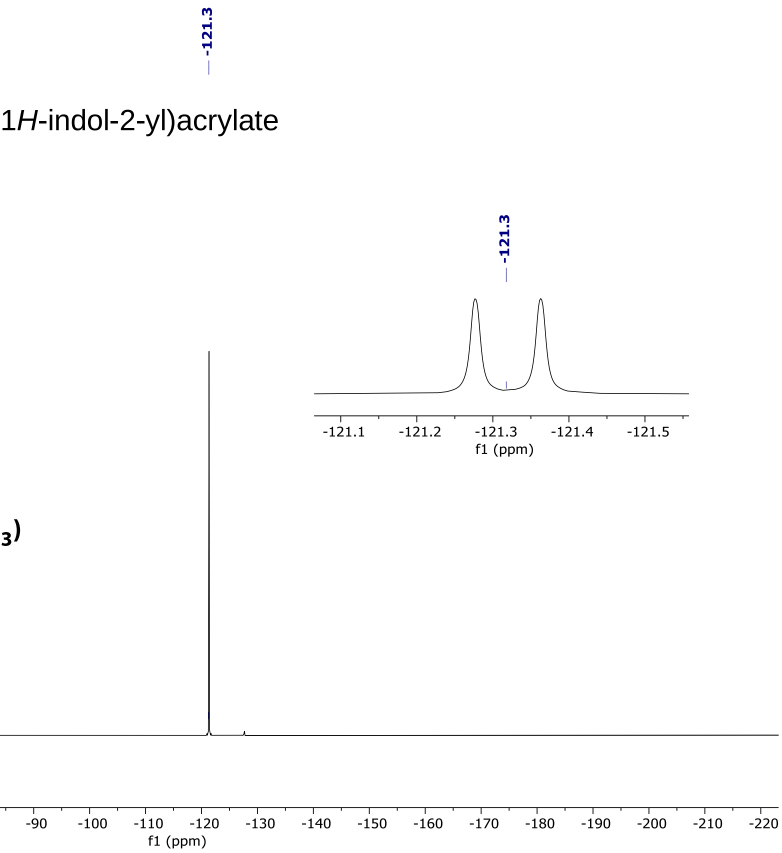

(*Z*)-2-(2-fluoro-2-phenylvinyl)-1-methyl-1*H*-indole

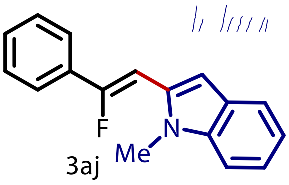

3aj

^{1}H NMR (400 MHz, $CDCl_3$)

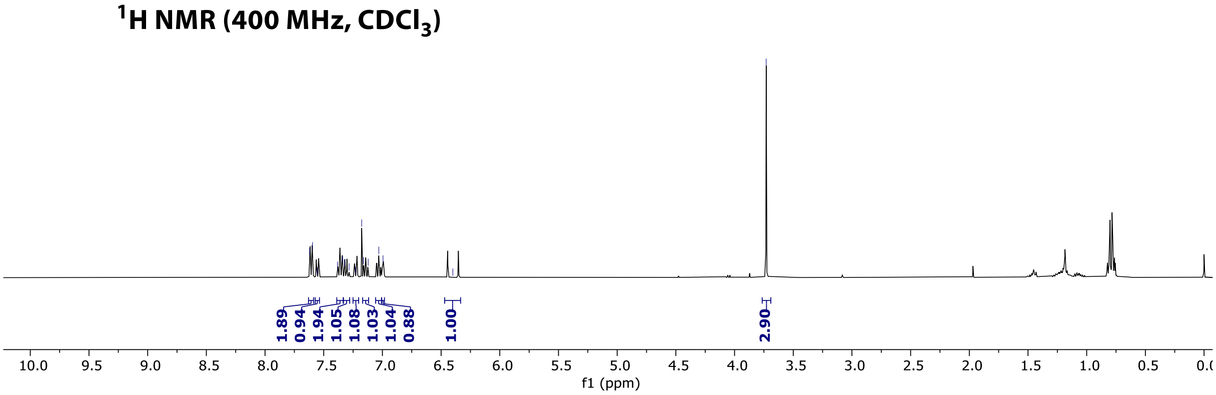

(*Z*)-2-(2-fluoro-2-phenylvinyl)-1-methyl-1*H*-indole

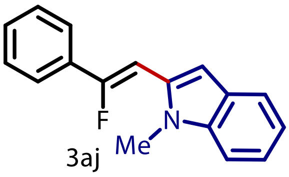

^{13}C NMR (101 MHz, CDCl$_3$)

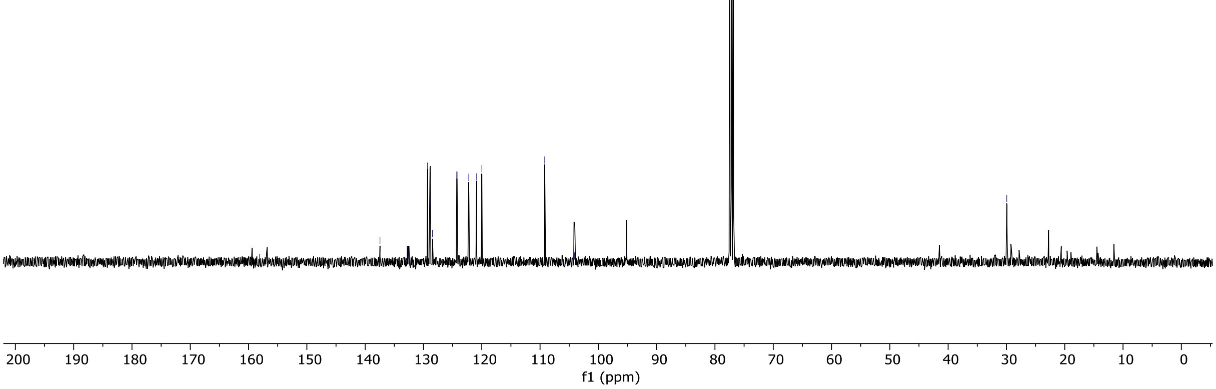

(*Z*)-2-(2-fluoro-2-phenylvinyl)-1-methyl-1*H*-indole

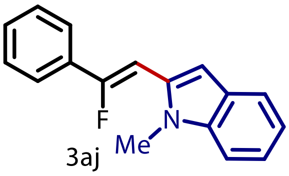

^{19}F NMR (377 MHz, CDCl$_3$)

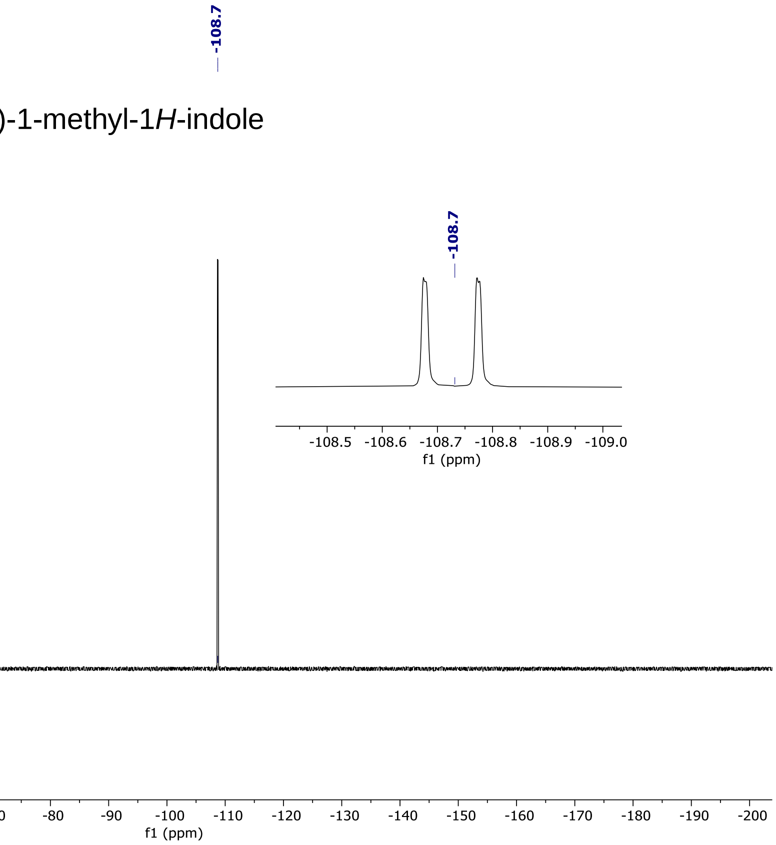

(*Z*)-2-(2-fluoro-2-phenylvinyl)-1*H*-indole

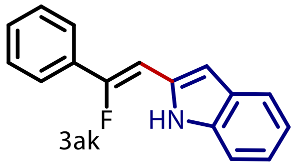

3ak

^{1}H NMR (400 MHz, $CDCl_3$)

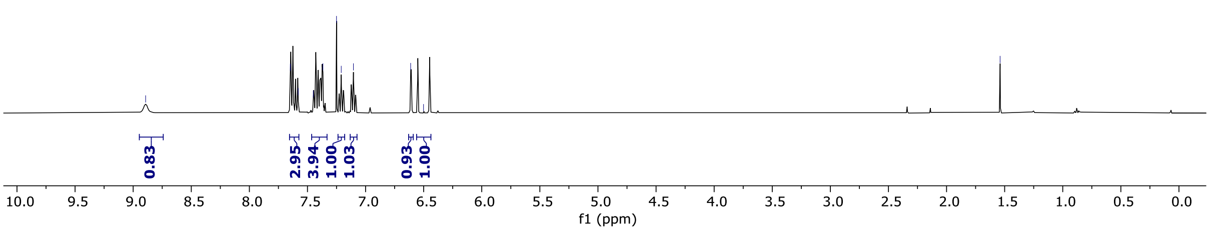

(*Z*)-2-(2-fluoro-2-phenylvinyl)-1*H*-indole

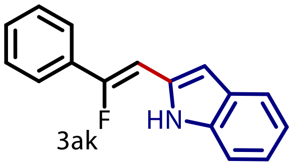

3ak

^{13}C NMR (101 MHz, $CDCl_3$)

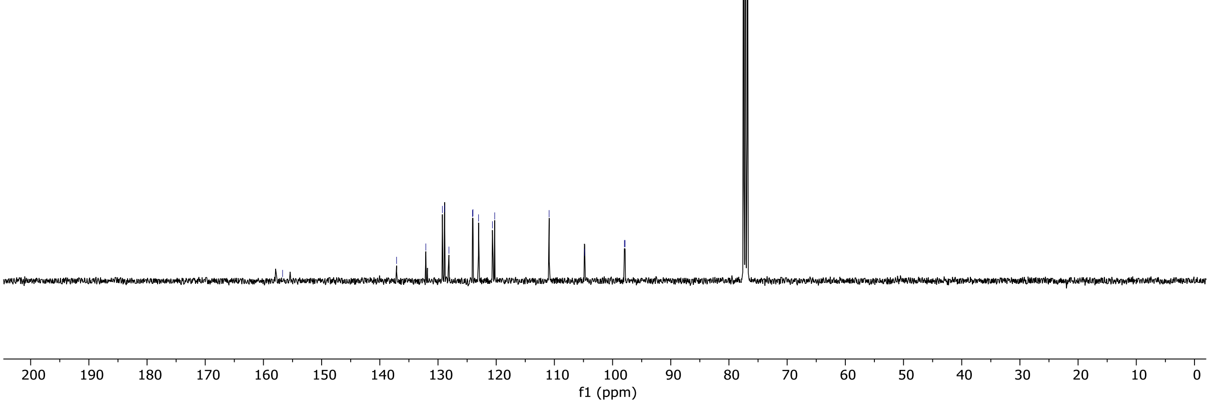

(*Z*)-2-(2-fluoro-2-phenylvinyl)-1*H*-indole

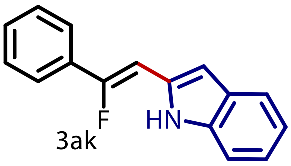

3ak

^{19}F NMR (377 MHz, CDCl$_3$)

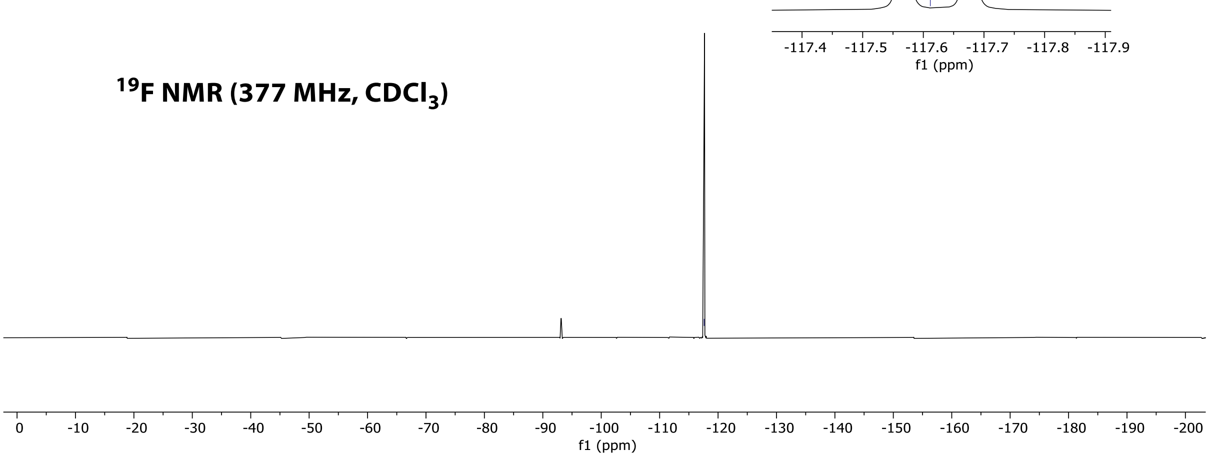

(*Z*)-2-(2-fluoro-2-(4-fluorophenyl)vinyl)-1-methyl-1*H*-indole

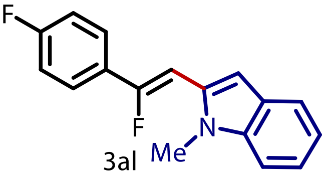

3al

^{1}H NMR (400 MHz, CDCl$_3$)

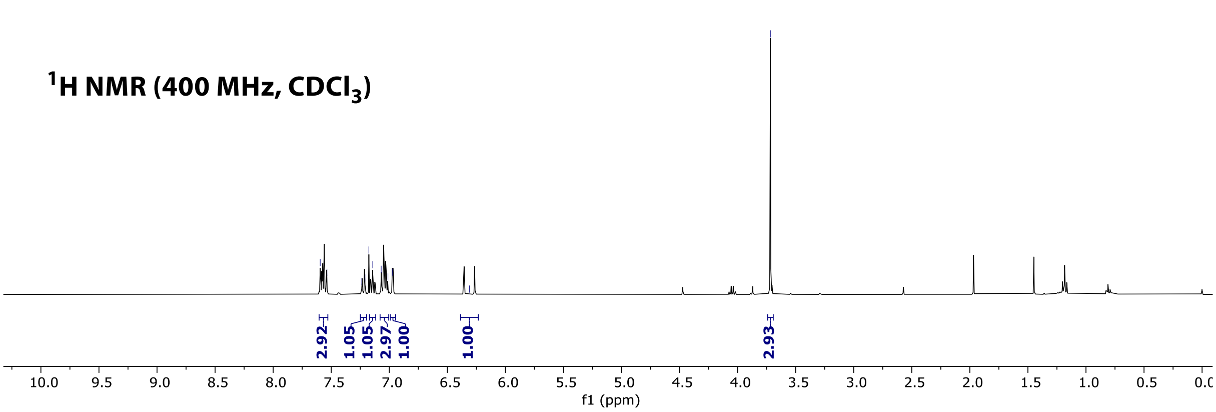

(*Z*)-2-(2-fluoro-2-(4-fluorophenyl)vinyl)-1-methyl-1*H*-indole

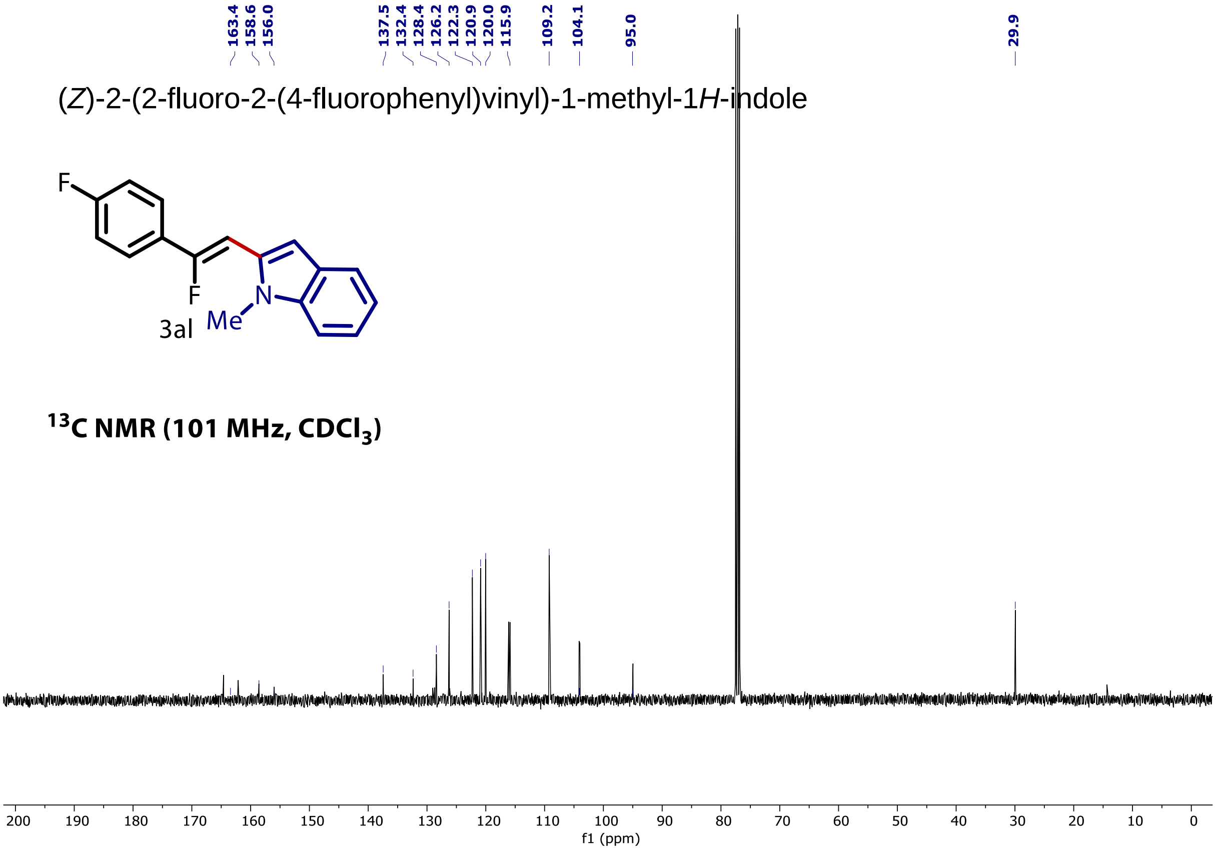

(*Z*)-2-(2-fluoro-2-(4-fluorophenyl)vinyl)-1-methyl-1*H*-indole

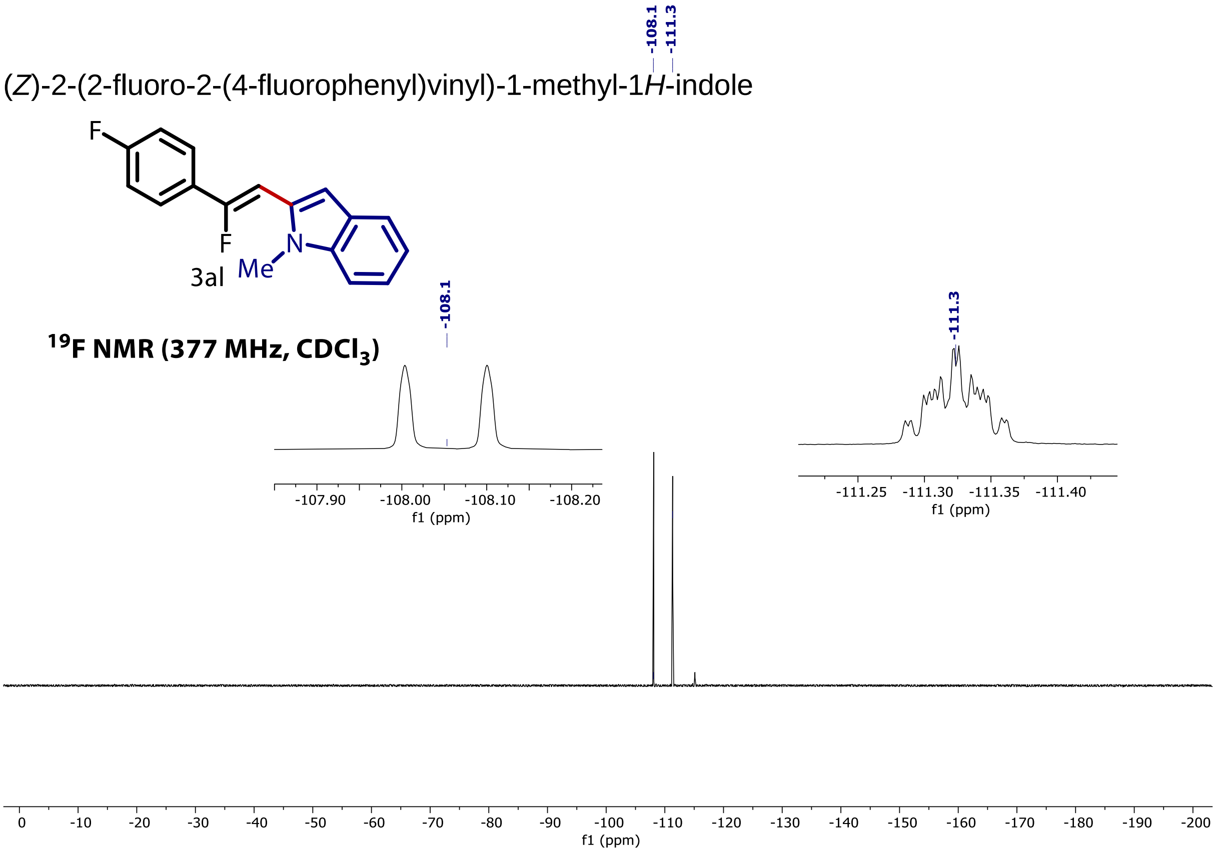

(*Z*)-2-(2-fluoro-2-(4-(trifluoromethyl)phenyl)vinyl)-1-methyl-1*H*-indole

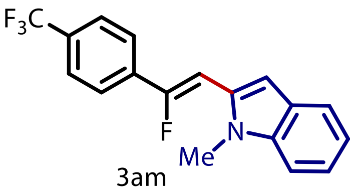

3am

^{1}H NMR (400 MHz, $CDCl_3$)

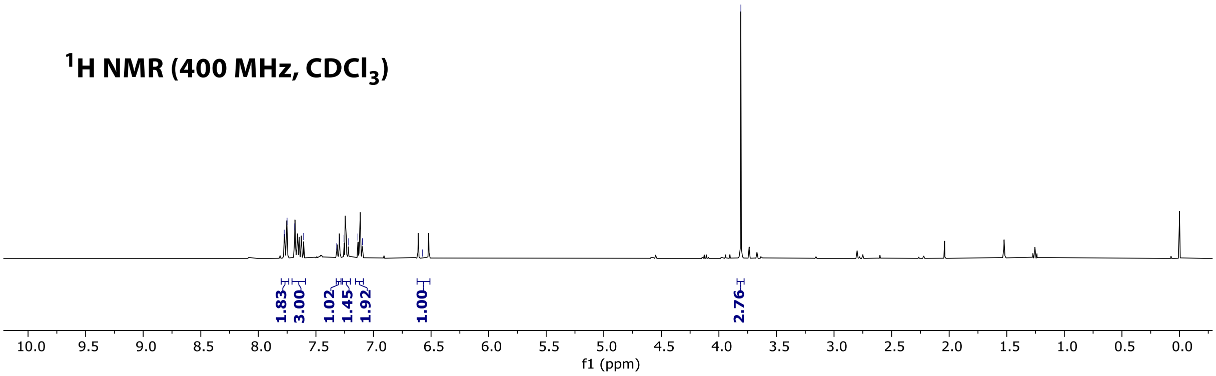

(*Z*)-2-(2-fluoro-2-(4-(trifluoromethyl)phenyl)vinyl)-1-methyl-1*H*-indole

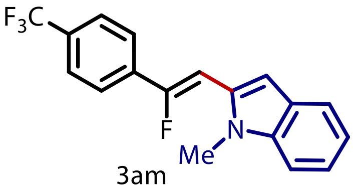

3am

^{13}C NMR (101 MHz, $CDCl_3$)

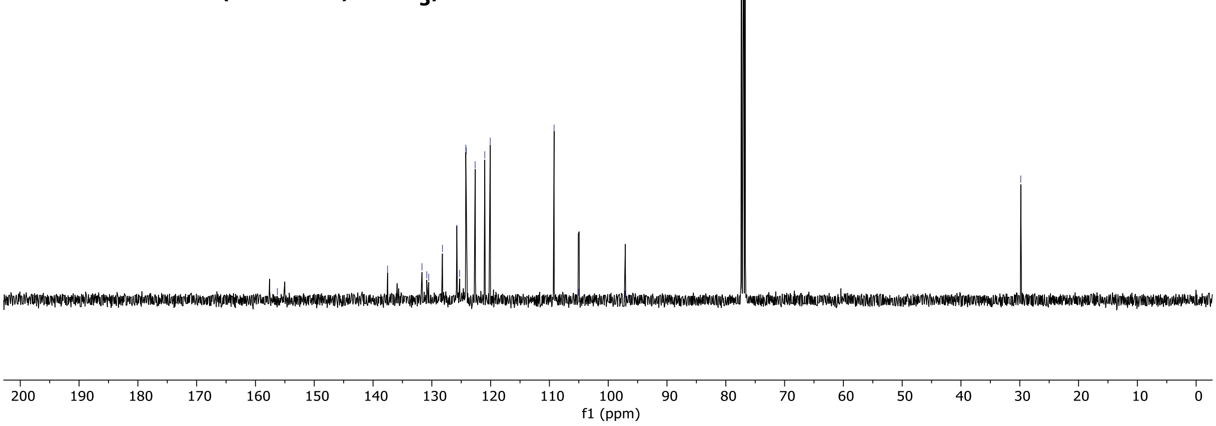

(*Z*)-2-(2-fluoro-2-(4-(trifluoromethyl)phenyl)vinyl)-1-methyl-1*H*-indole

^{19}F NMR (377 MHz, $CDCl_3$)

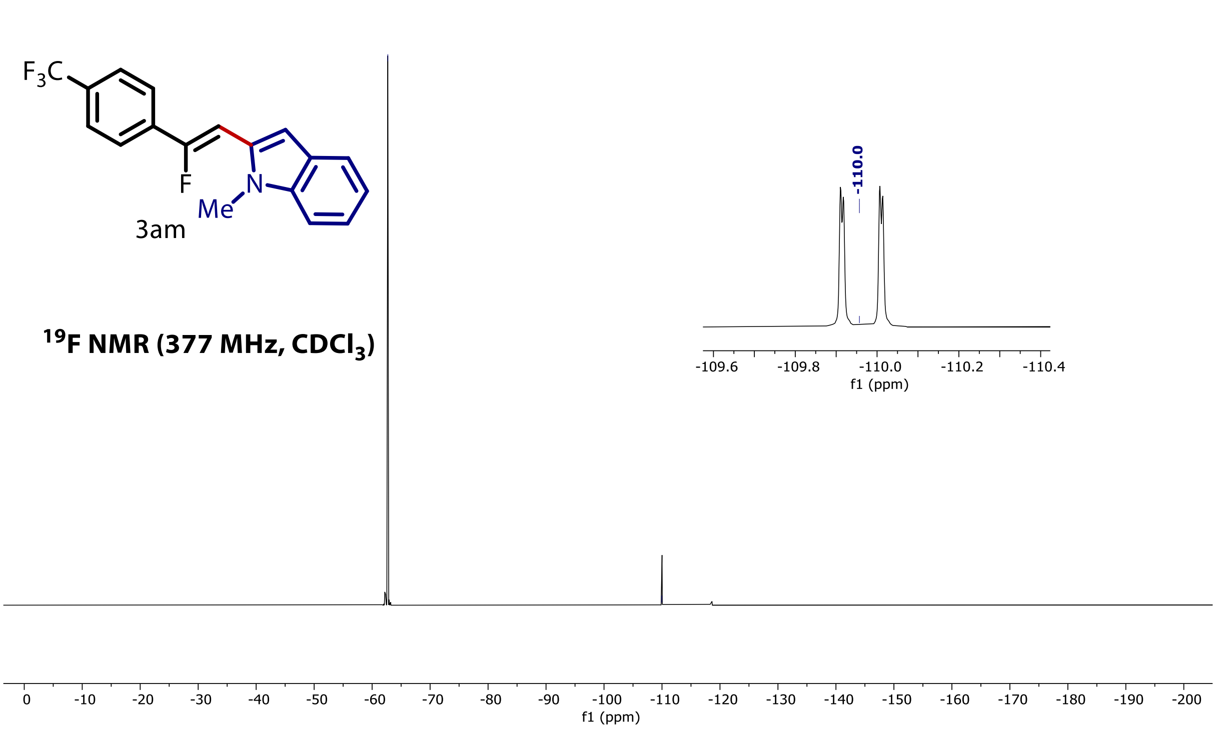

(*Z*)-2-(2-fluoro-2-(thiophen-3-yl)vinyl)-1-methyl-1*H*-indole

^{1}H NMR (400 MHz, $CDCl_3$)

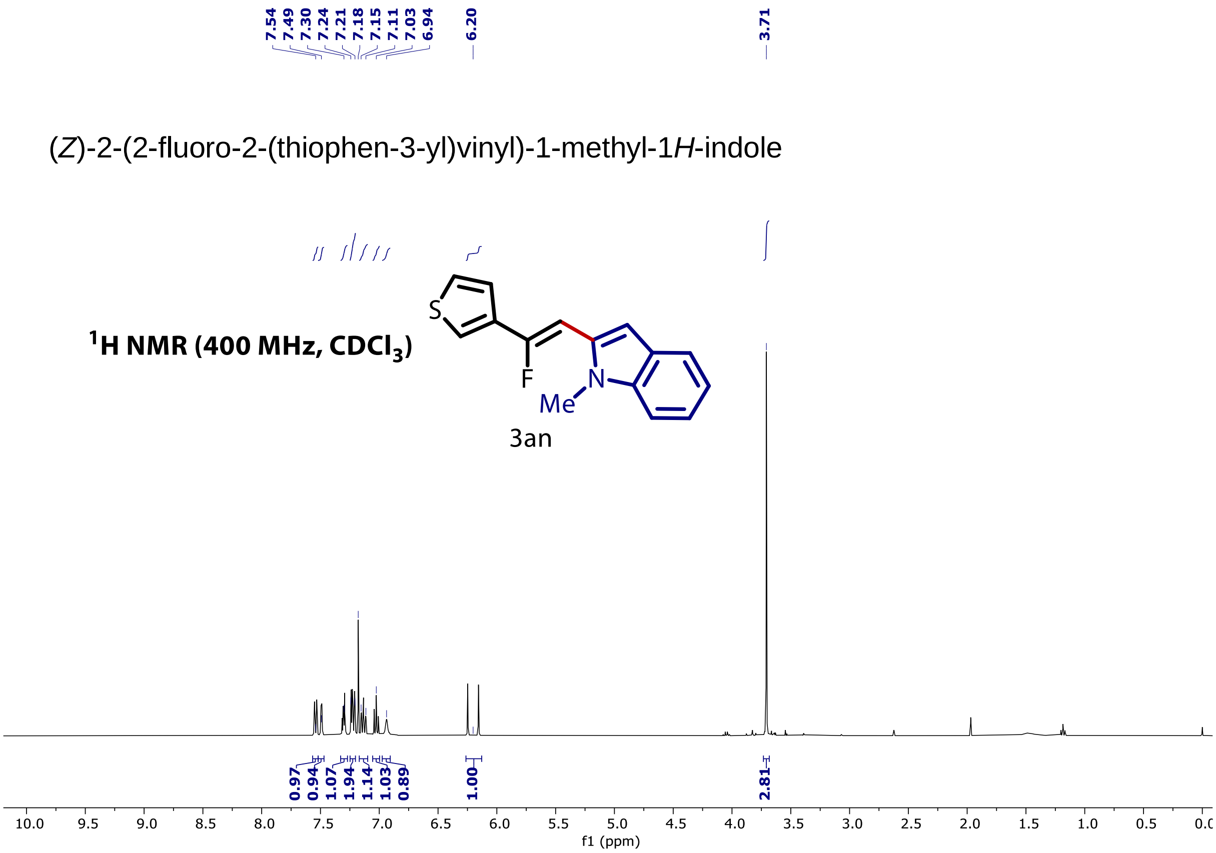

(*Z*)-2-(2-fluoro-2-(thiophen-3-yl)vinyl)-1-methyl-1*H*-indole

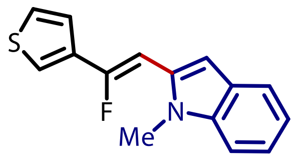

3an

^{13}C NMR (101 MHz, CDCl$_3$)

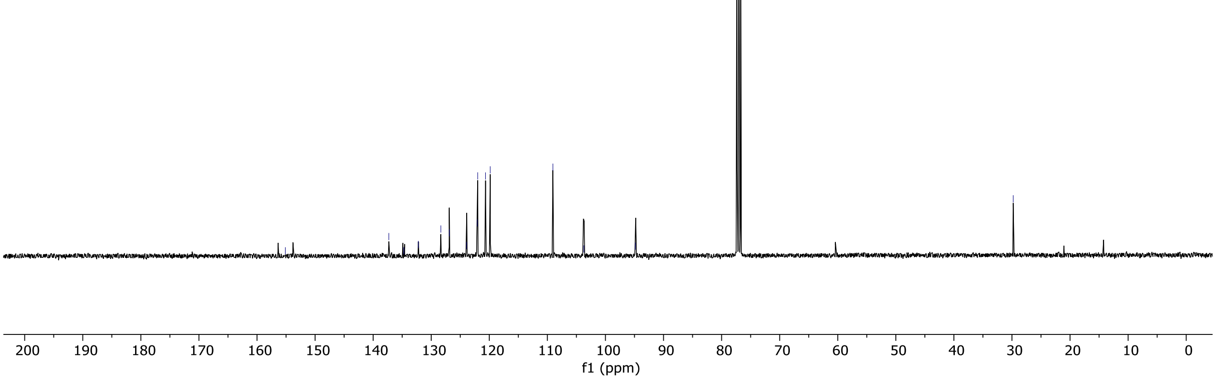

(*Z*)-2-(2-fluoro-2-(thiophen-3-yl)vinyl)-1-methyl-1*H*-indole

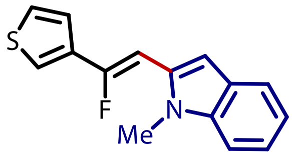

3an

^{19}F NMR (377 MHz, CDCl$_3$)

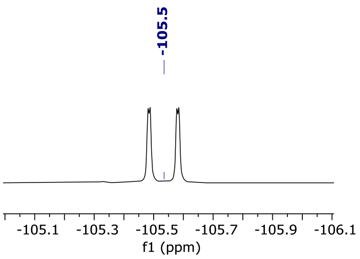

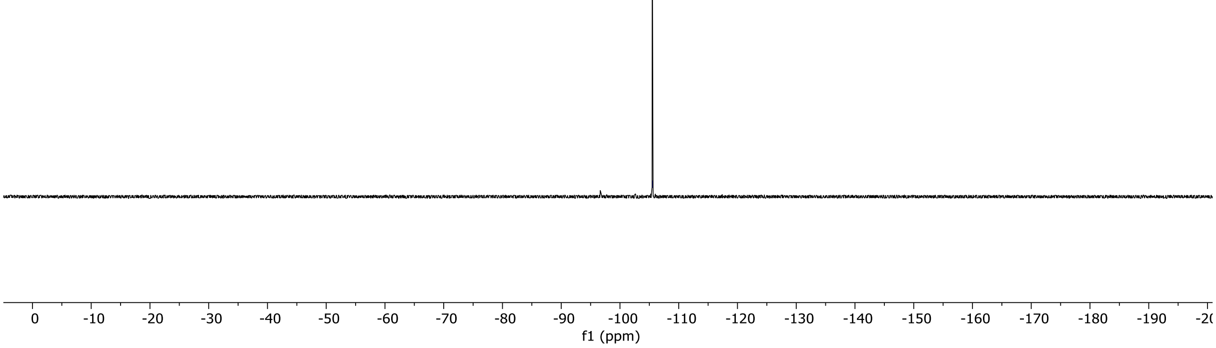

(*E*)-2-(2-fluorododec-1-en-1-yl)-1*H*-indole

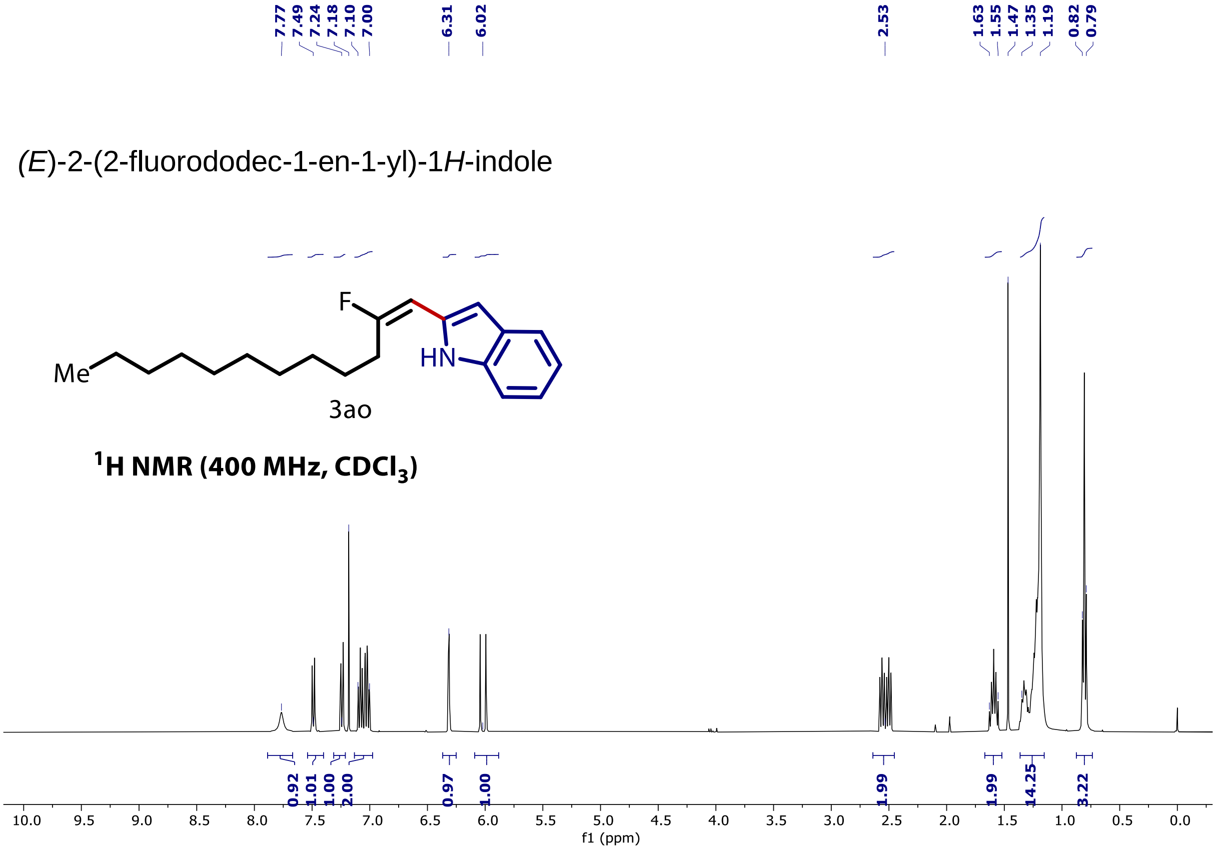

(*E*)-2-(2-fluorododec-1-en-1-yl)-1*H*-indole

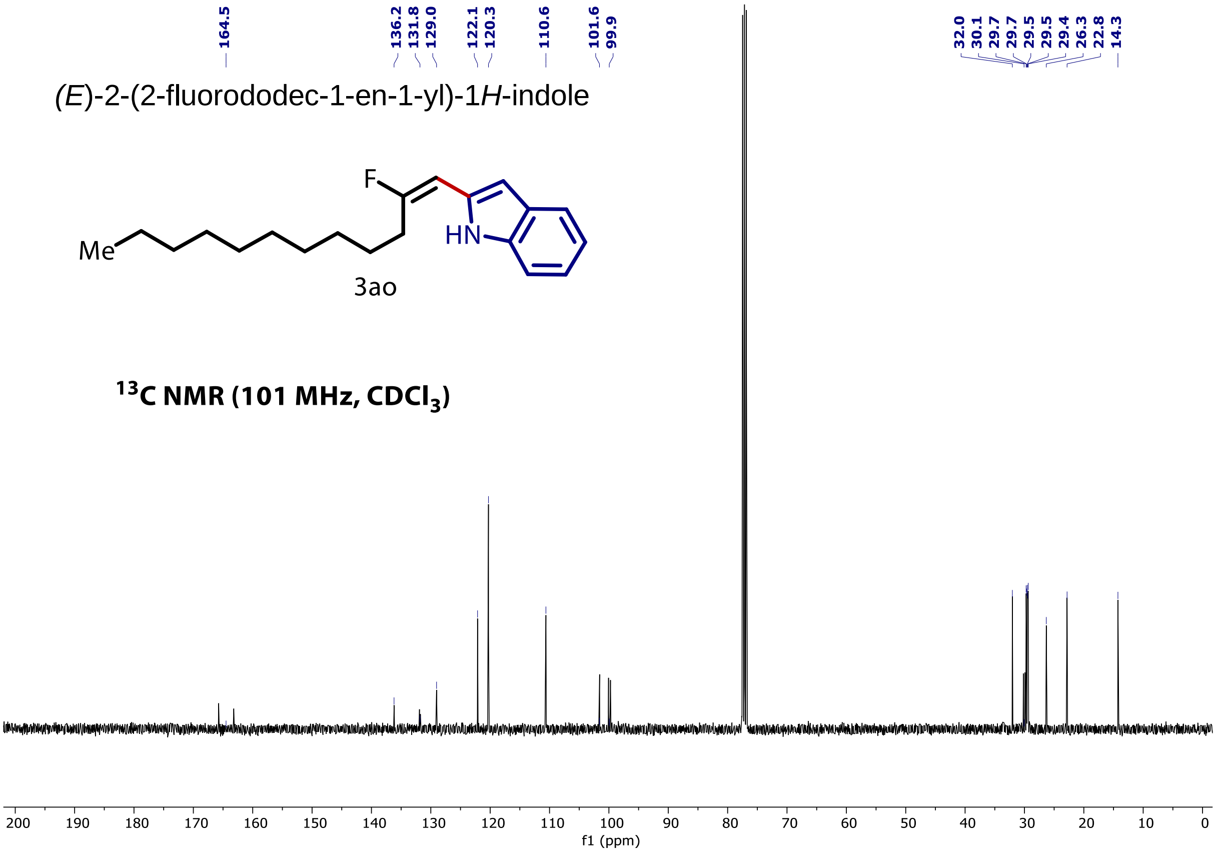

(*E*)-2-(2-fluorododec-1-en-1-yl)-1*H*-indole

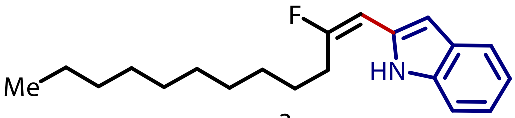

3ao

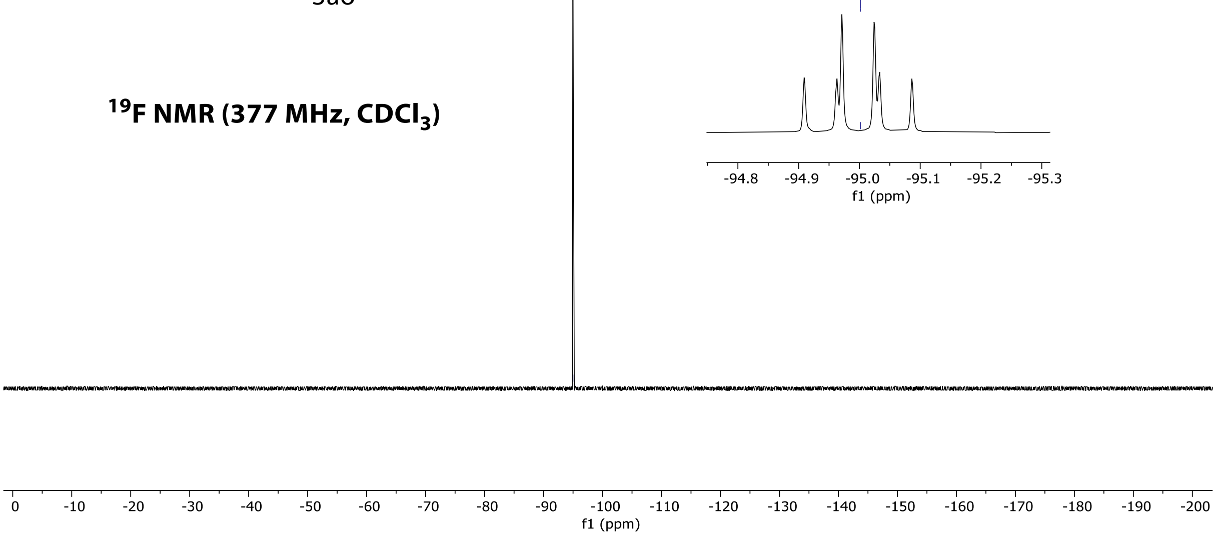

(*Z*)-3-(2-fluoro-5-phenylpent-1-en-1-yl)-1,2,5-trimethyl-1*H*-pyrrole

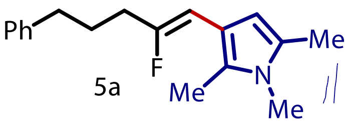

5a

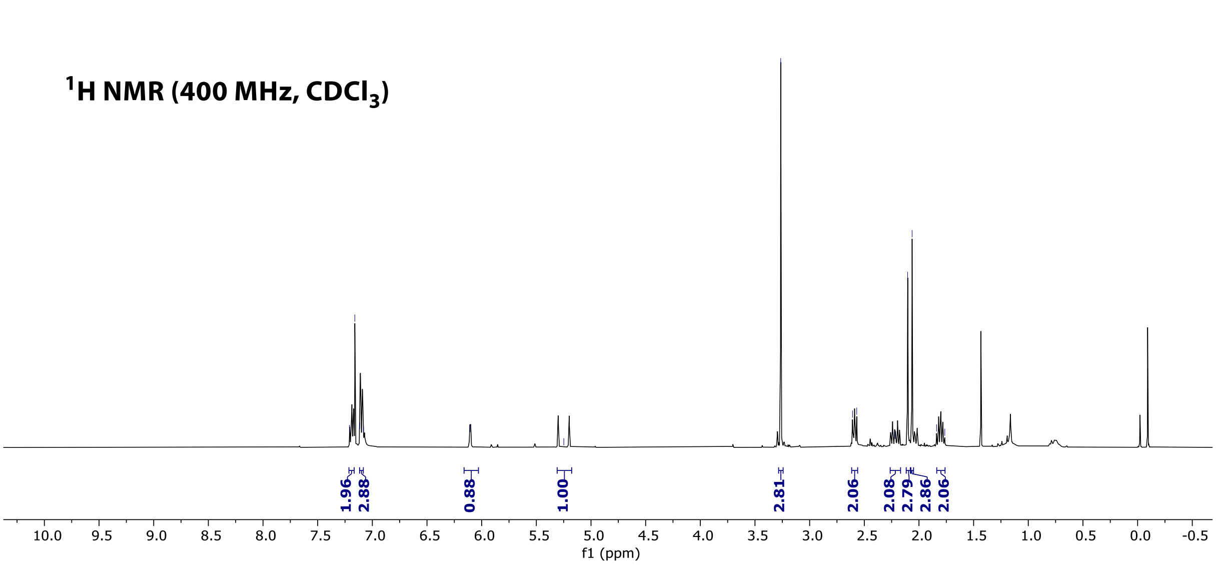

(*Z*)-3-(2-fluoro-5-phenylpent-1-en-1-yl)-1,2,5-trimethyl-1*H*-pyrrole

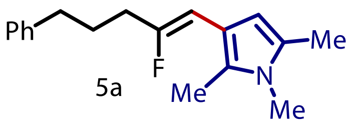

^{13}C NMR (101 MHz, CDCl$_3$)

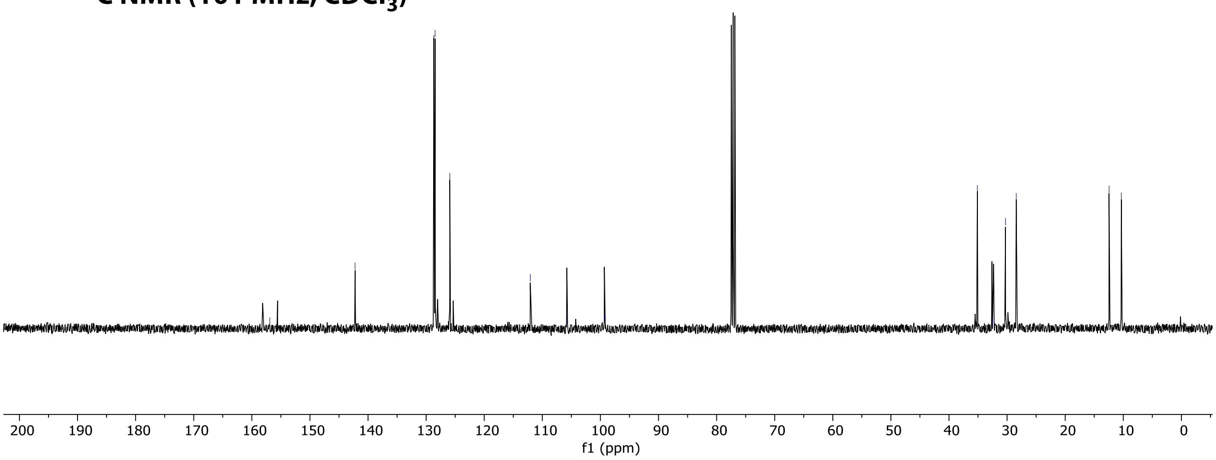

(*Z*)-3-(2-fluoro-5-phenylpent-1-en-1-yl)-1,2,5-trimethyl-1*H*-pyrrole

^{19}F NMR (377 MHz, CDCl$_3$)

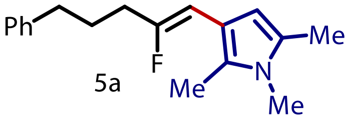

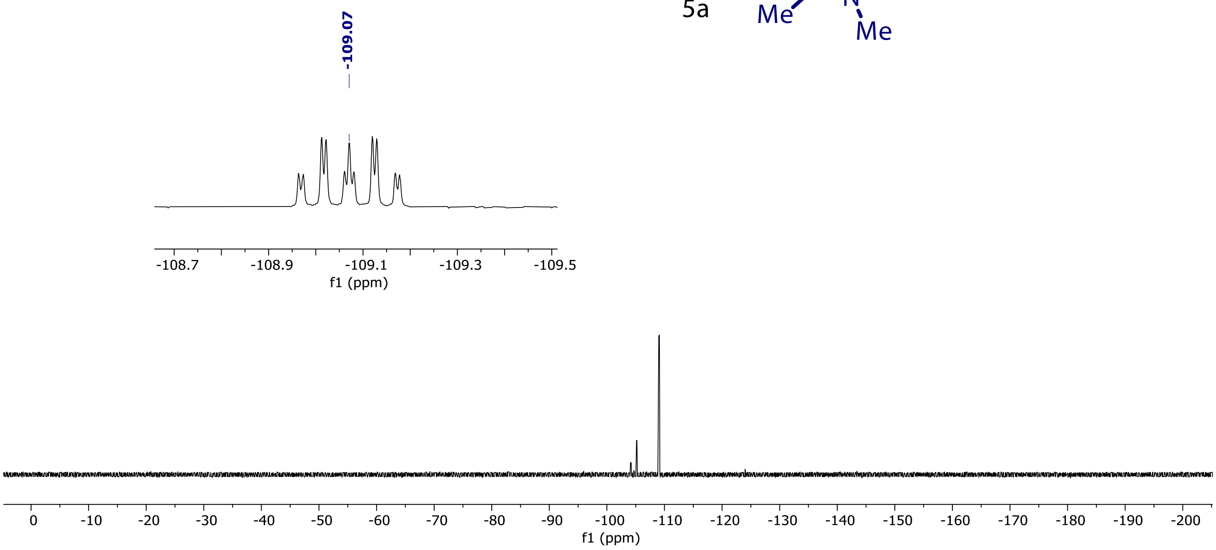

(*Z*)-3-(2-fluoro-5-phenylpent-1-en-1-yl)-1-(4-methoxyphenyl)-2,5-dimethyl-1*H*-pyrrole

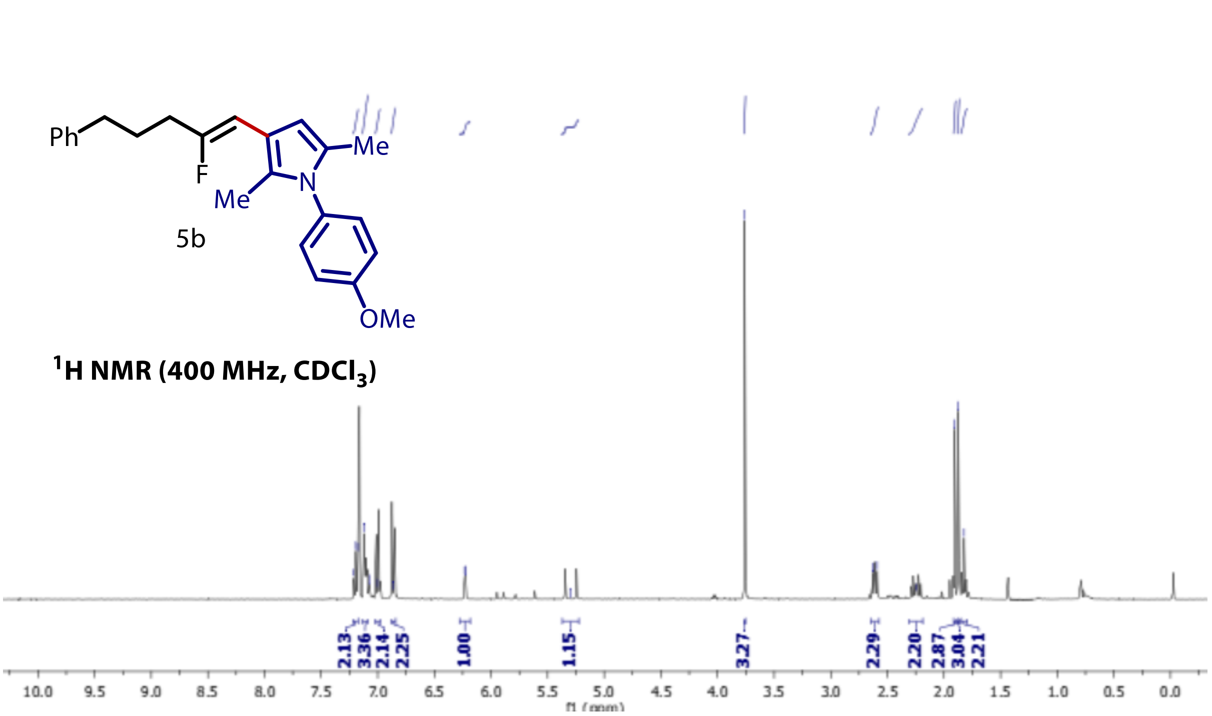

(*Z*)-3-(2-fluoro-5-phenylpent-1-en-1-yl)-1-(4-methoxyphenyl)-2,5-dimethyl-1*H*-pyrrole

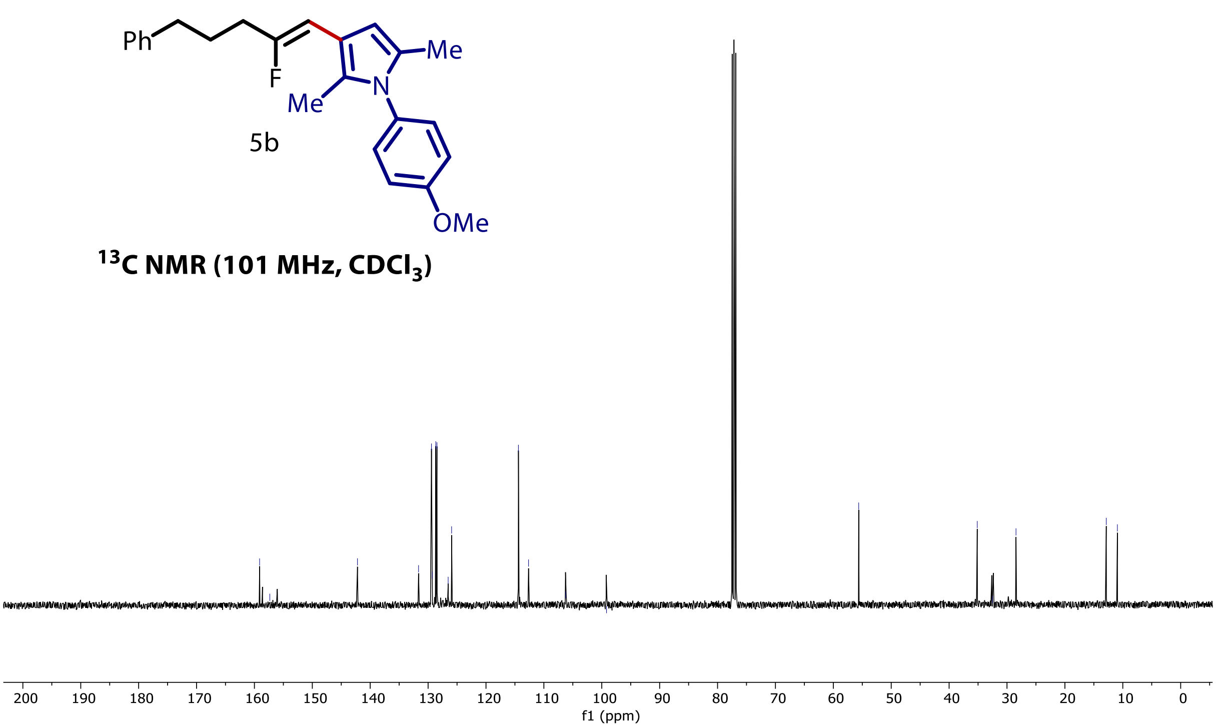

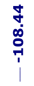

(*Z*)-3-(2-fluoro-5-phenylpent-1-en-1-yl)-1-(4-methoxyphenyl)-2,5-dimethyl-1*H*-pyrrole

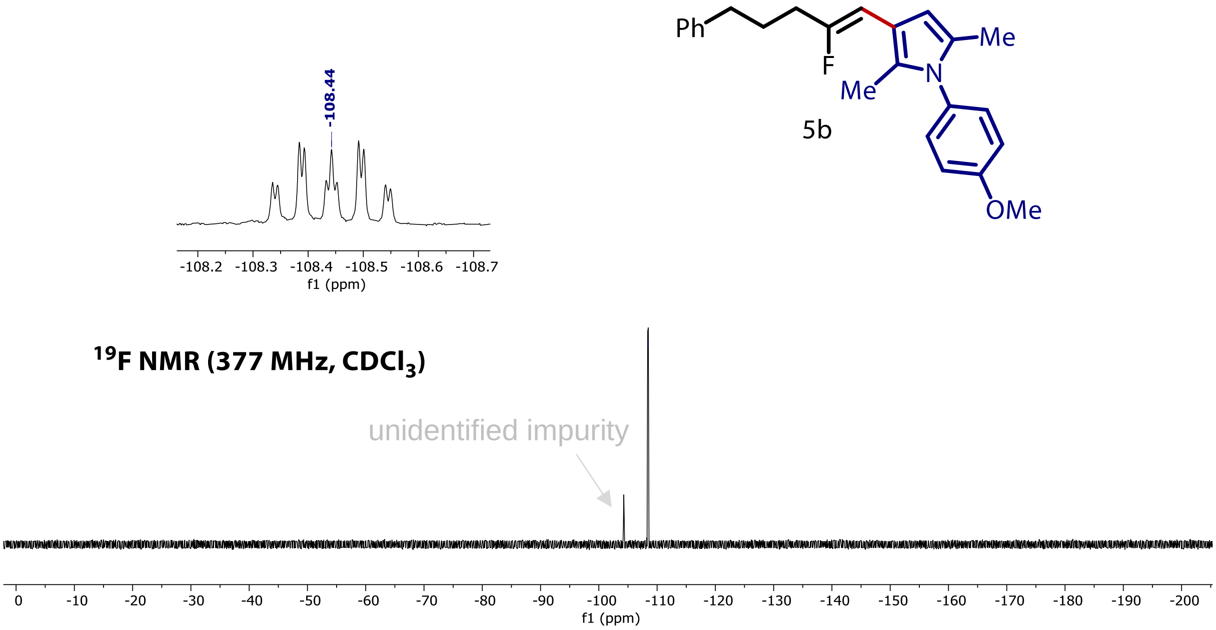

(*Z*)-1-cyclopropyl-3-(2-fluoro-5-phenylpent-1-en-1-yl)-2,5-dimethyl-1*H*-pyrrole

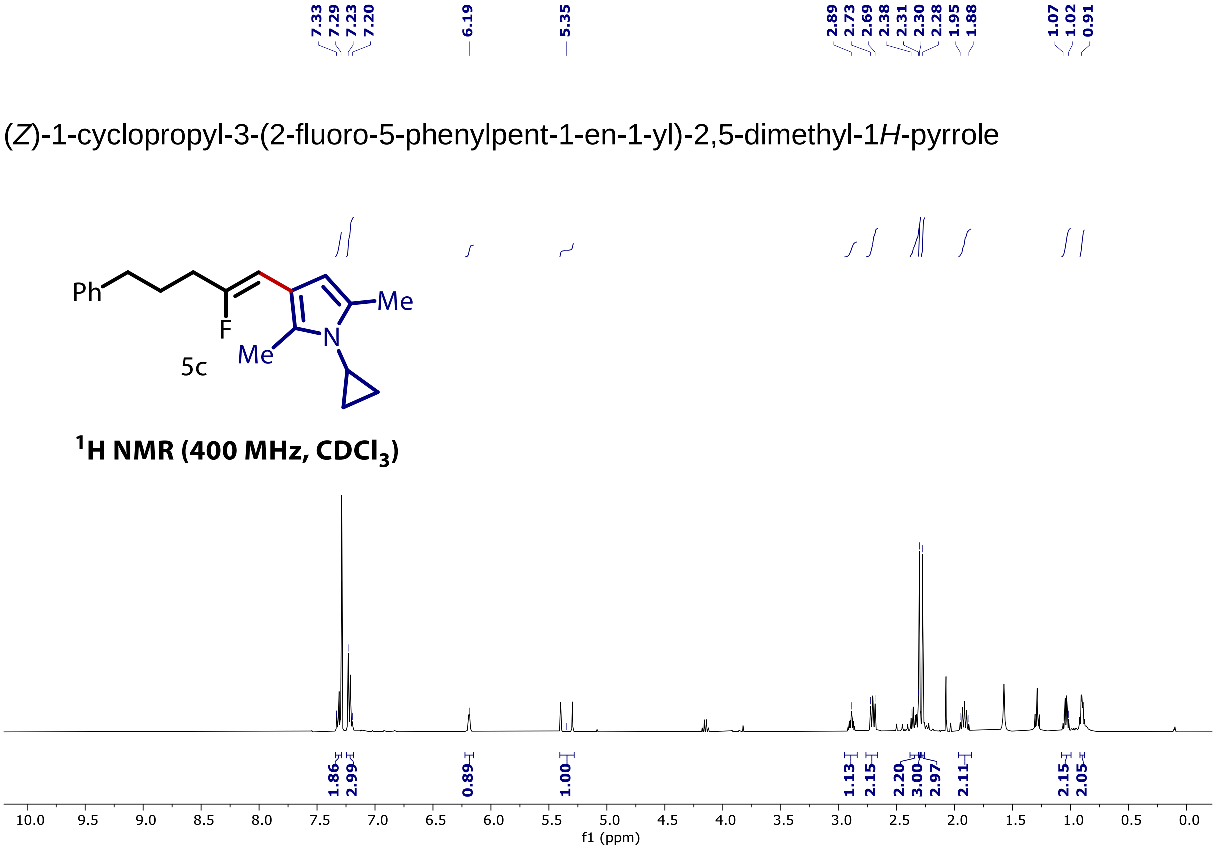

(*Z*)-1-cyclopropyl-3-(2-fluoro-5-phenylpent-1-en-1-yl)-2,5-dimethyl-1*H*-pyrrole

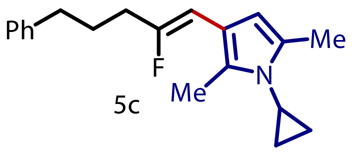

^{13}C NMR (101 MHz, $CDCl_3$)

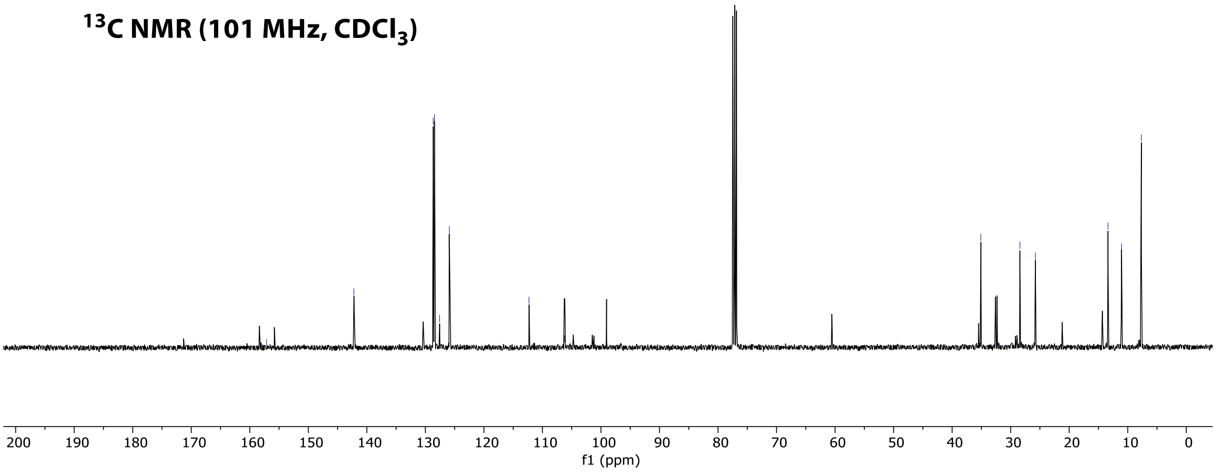

(*Z*)-1-cyclopropyl-3-(2-fluoro-5-phenylpent-1-en-1-yl)-2,5-dimethyl-1*H*-pyrrole

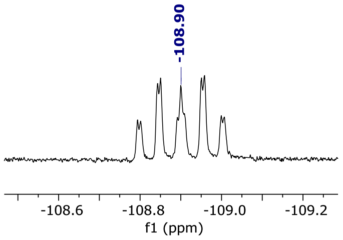

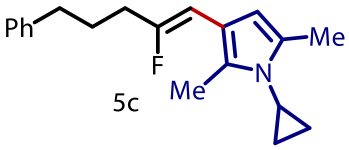

^{19}F NMR (377 MHz, $CDCl_3$)

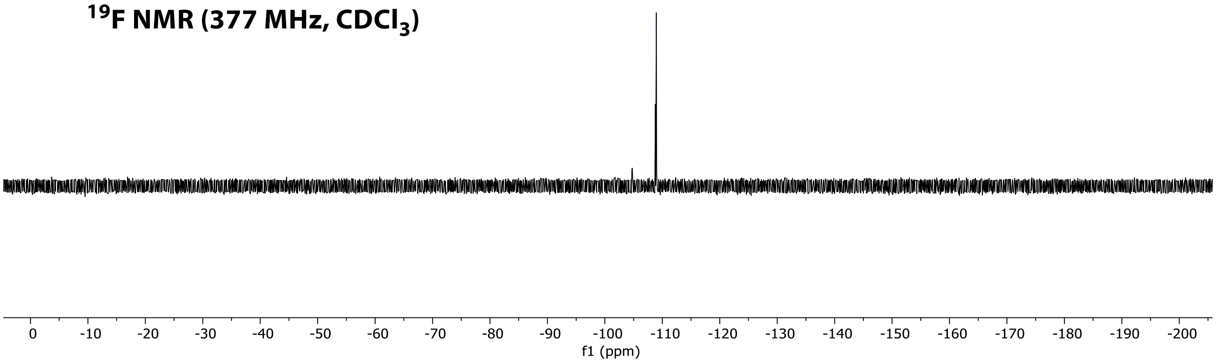

(*Z*)-3-(2-fluoro-5-phenylpent-1-en-1-yl)-2,5-dimethyl-1*H*-pyrrole

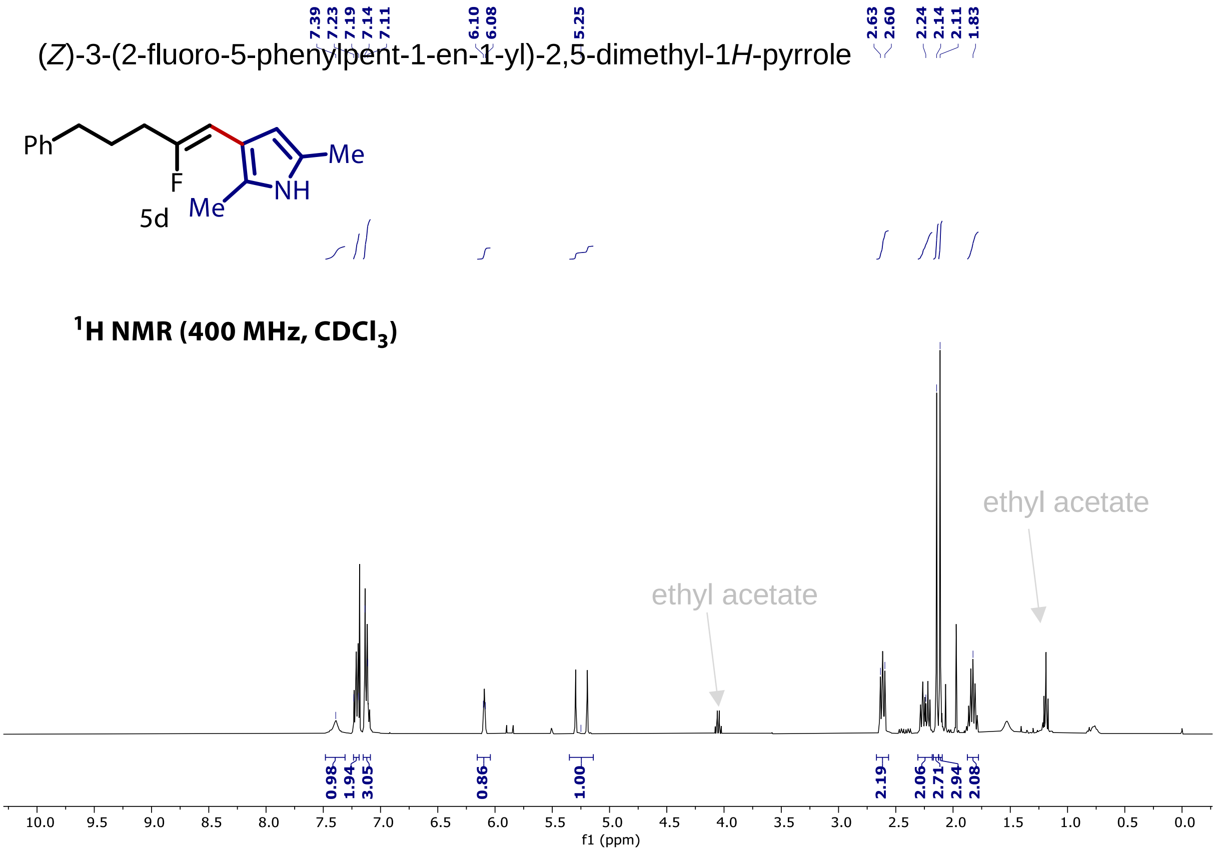

(*Z*)-3-(2-fluoro-5-phenylpent-1-en-1-yl)-2,5-dimethyl-1*H*-pyrrole

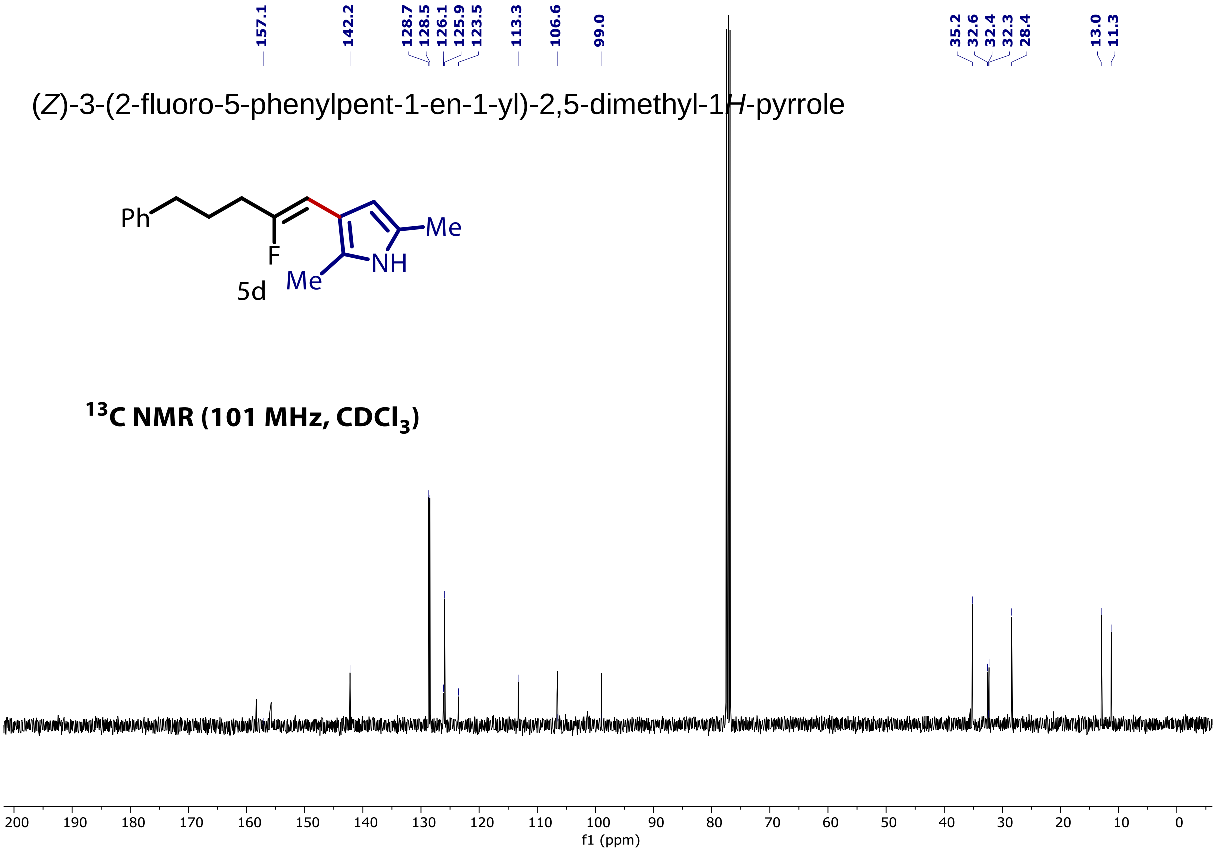

(*Z*)-3-(2-fluoro-5-phenylpent-1-en-1-yl)-2,5-dimethyl-1*H*-pyrrole

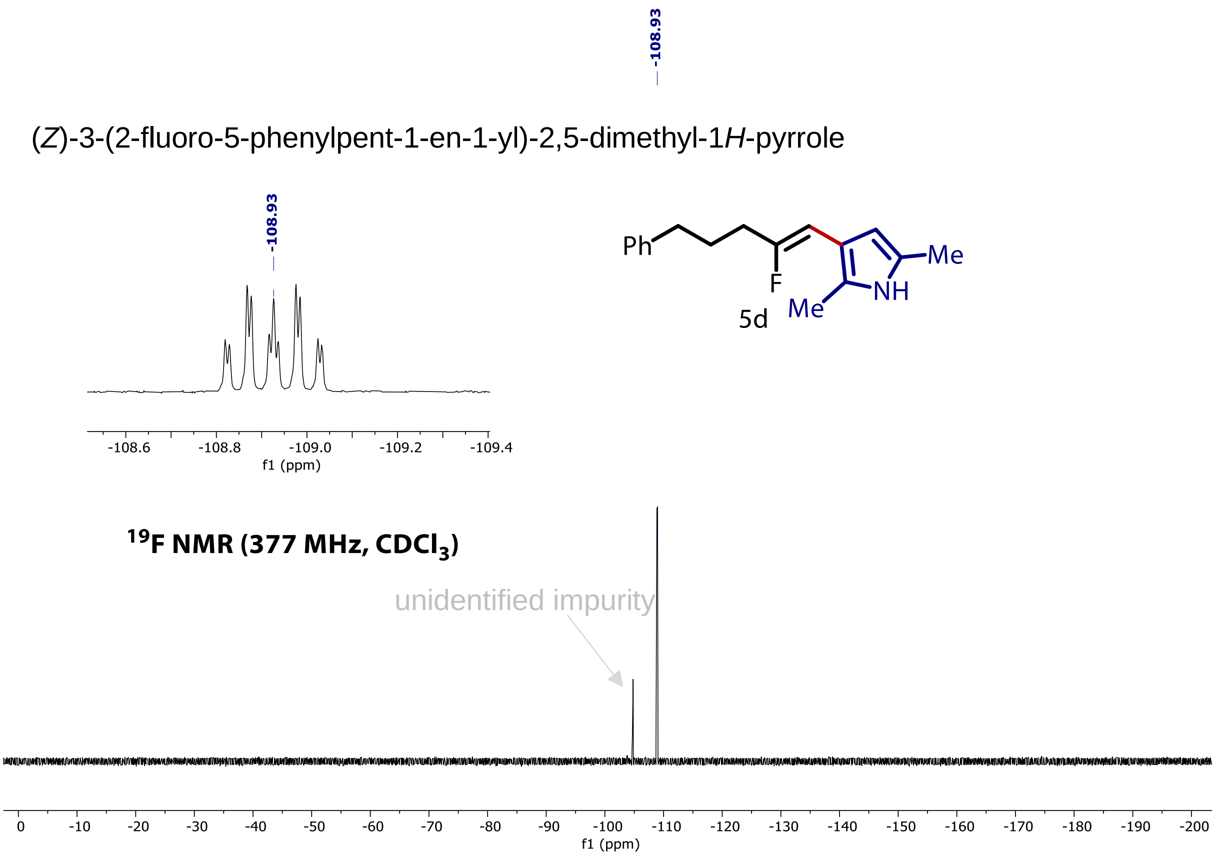

(*Z*)-3-(2-fluoro-3,3-dimethylbut-1-en-1-yl)-2,5-dimethyl-1*H*-pyrrole

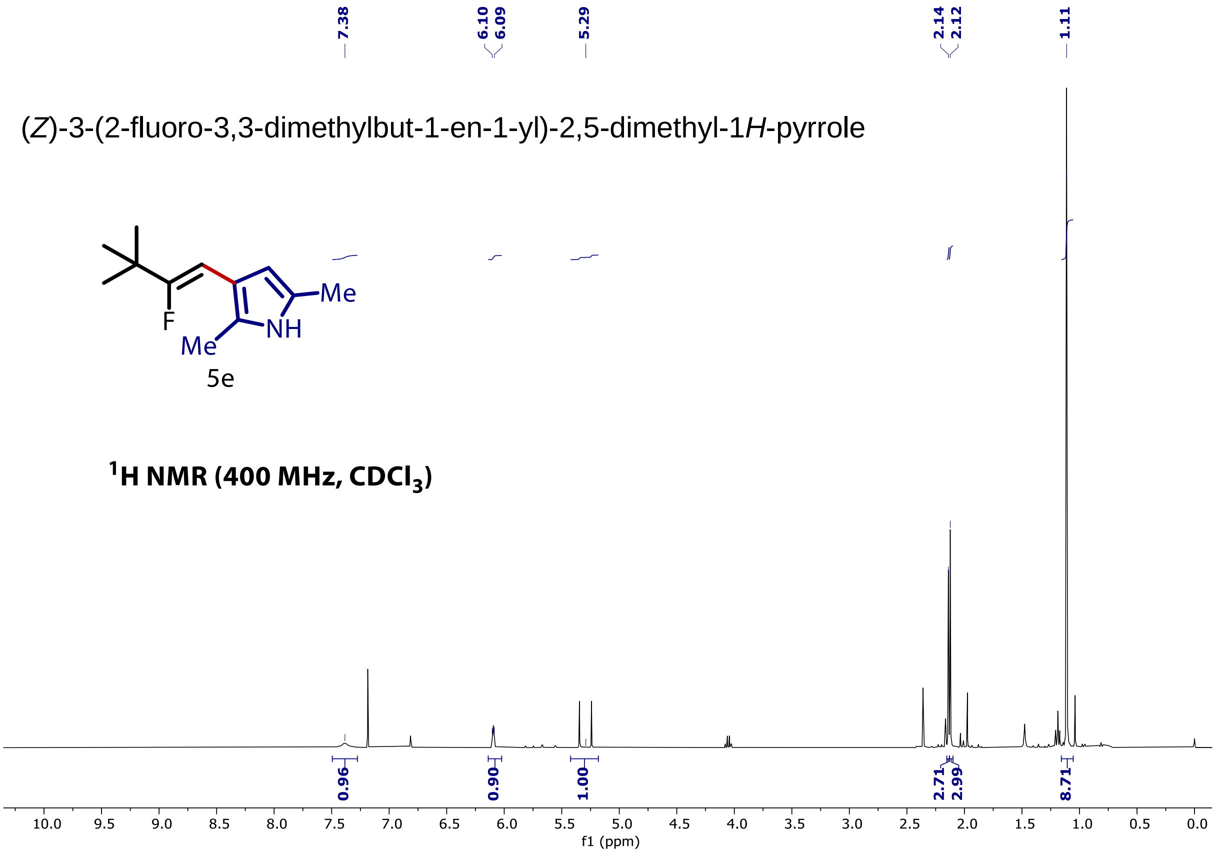

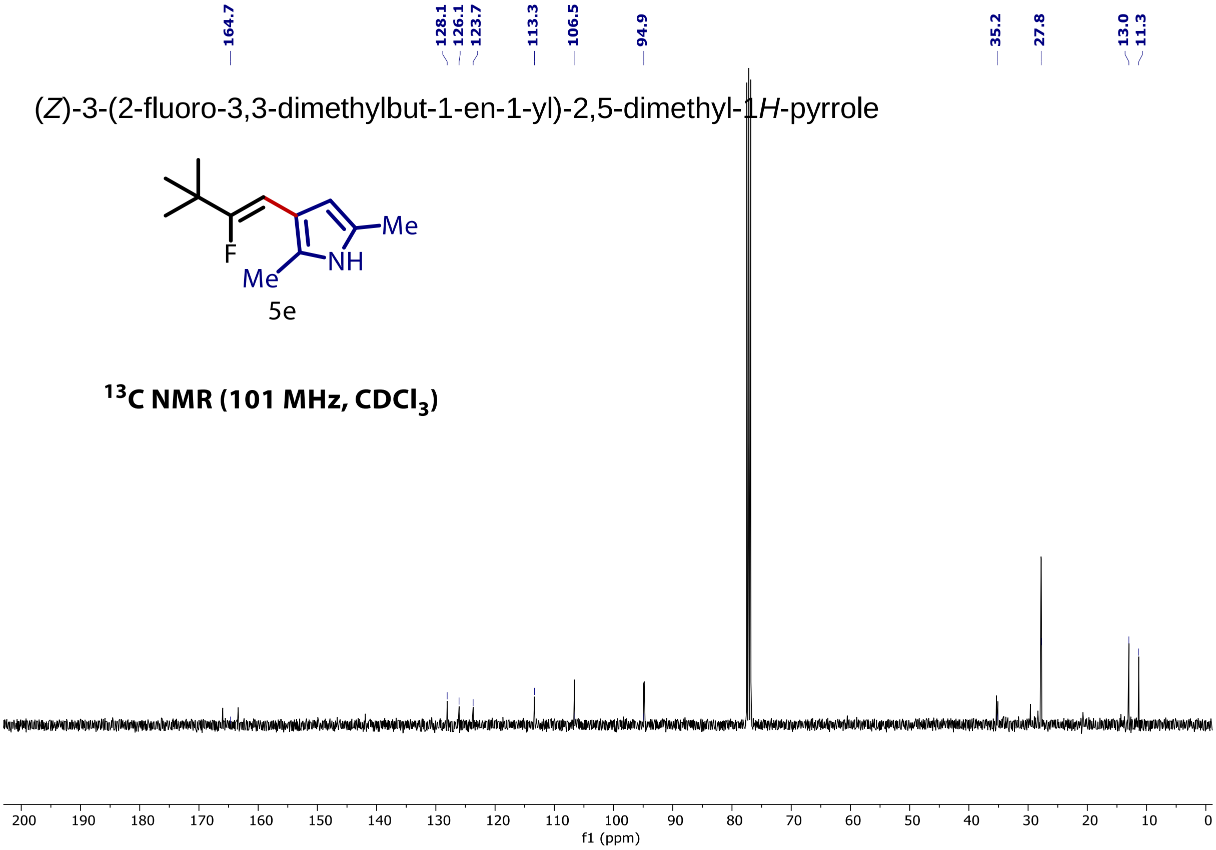
(Z)-3-(2-fluoro-3,3-dimethylbut-1-en-1-yl)-2,5-dimethyl-1H-pyrrole
F
Me
NH
Me
5e
13C NMR (101 MHz, CDCl3)
164.7
128.1
126.1
123.7
113.3
106.5
94.9
35.2
27.8
13.0
11.3
f1 (ppm)

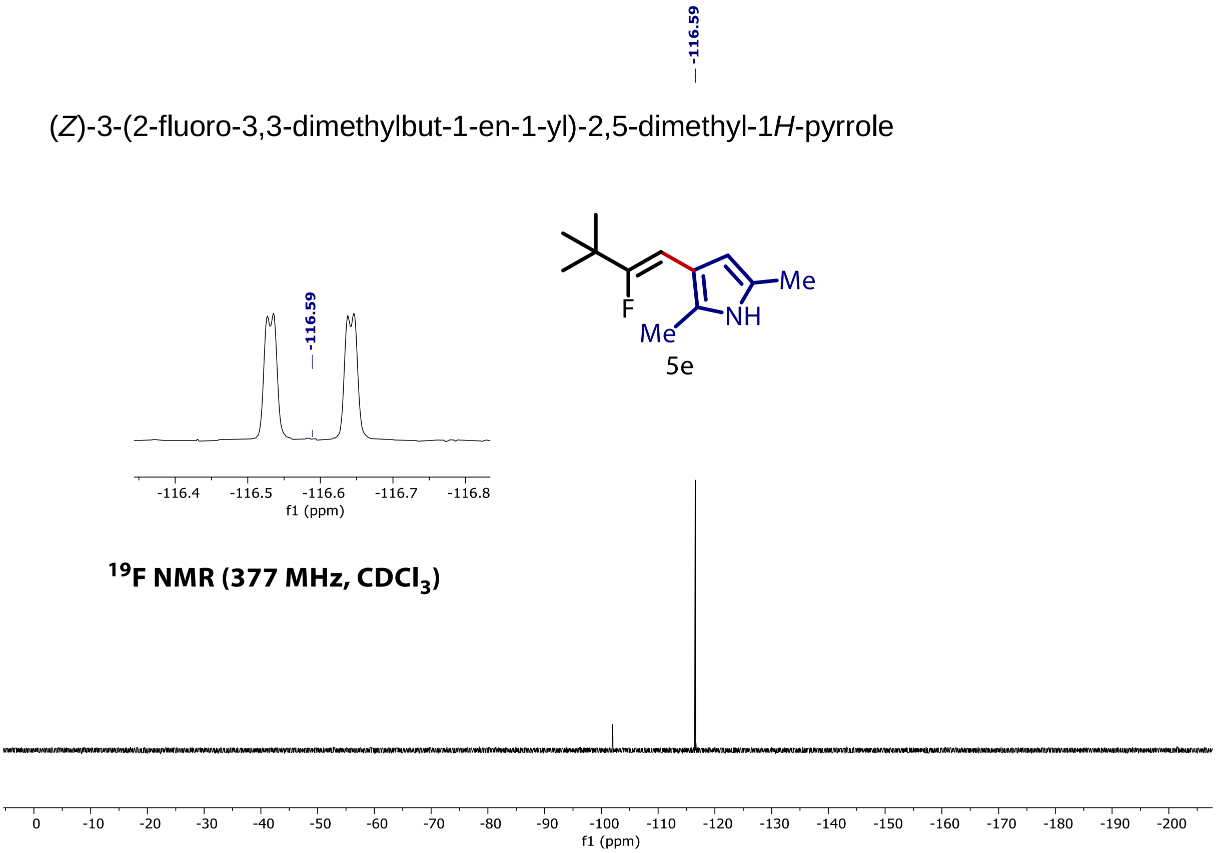
(Z)-3-(2-fluoro-3,3-dimethylbut-1-en-1-yl)-2,5-dimethyl-1H-pyrrole
F
Me
NH
Me
5e
19F NMR (377 MHz, CDCl3)
-116.59
-116.59
f1 (ppm)
f1 (ppm)

(*Z*)-3-(2-cyclohexyl-2-fluorovinyl)-2,5-dimethyl-1*H*-pyrrole

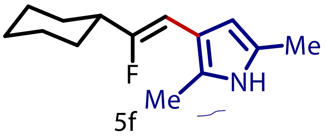

5f

^{1}H NMR (400 MHz, CDCl$_3$)

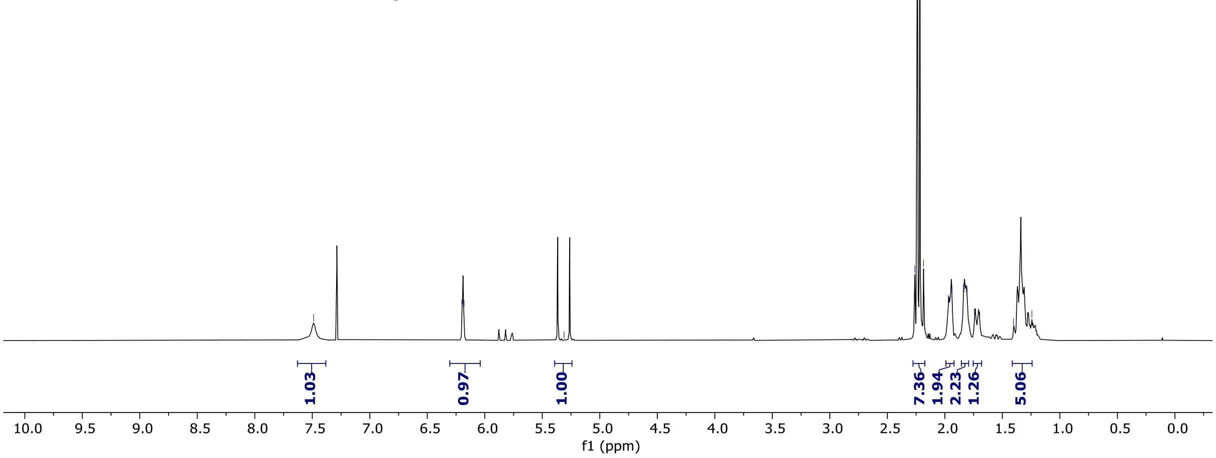

\

(*Z*)-3-(2-cyclohexyl-2-fluorovinyl)-2,5-dimethyl-1*H*-pyrrole

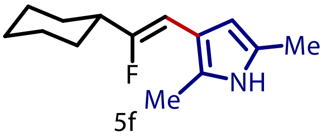

5f

^{13}C NMR (101 MHz, CDCl$_3$)

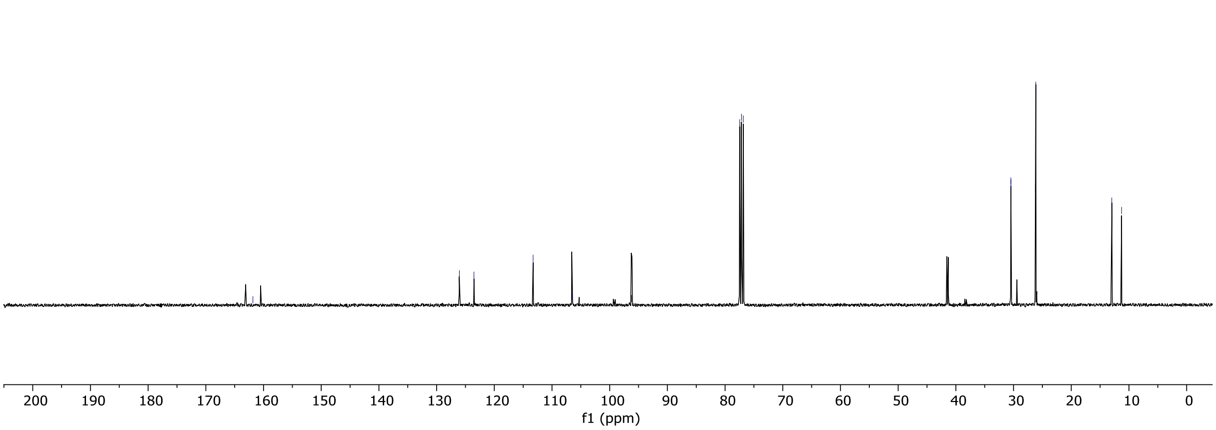

(*Z*)-3-(2-cyclohexyl-2-fluorovinyl)-2,5-dimethyl-1*H*-pyrrole

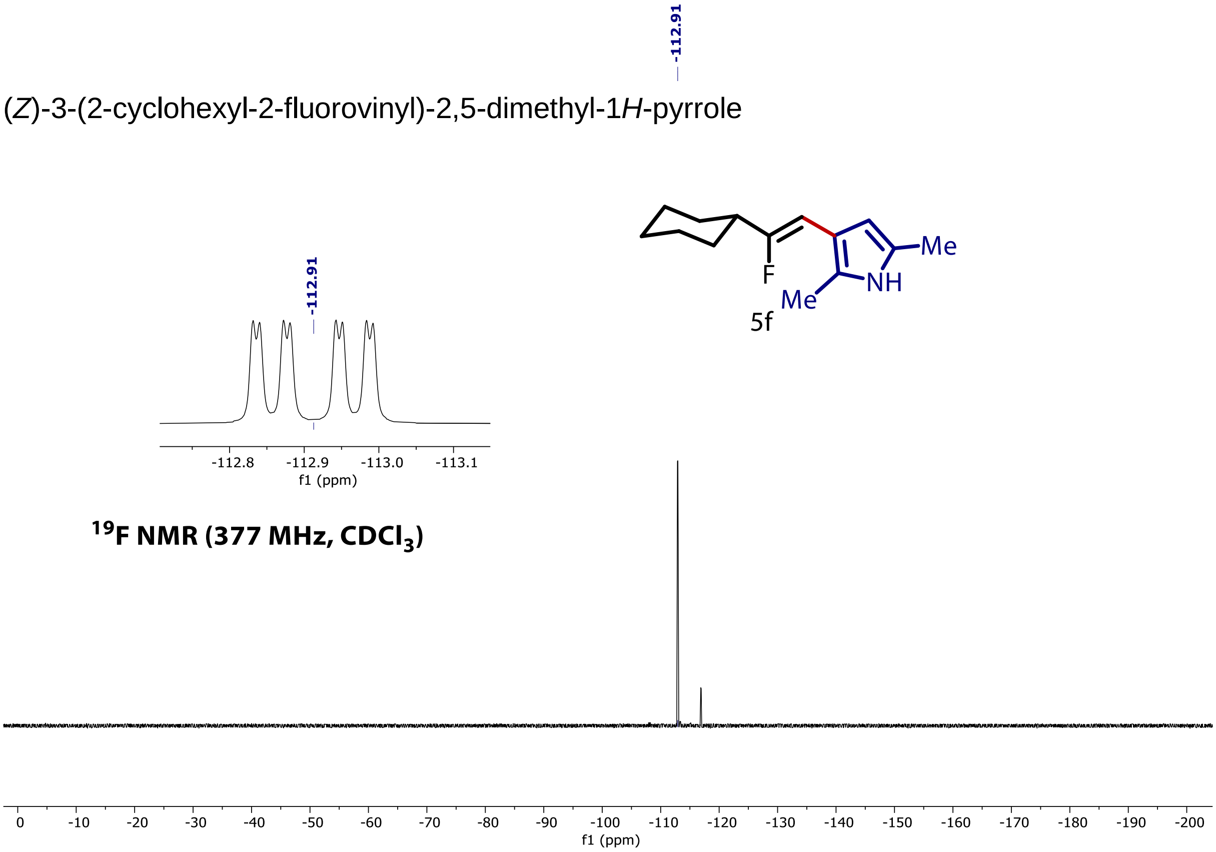

5f

^{19}F NMR (377 MHz, $CDCl_3$)

(*Z*)-3-(2-fluoro-2-(4-fluorophenyl)vinyl)-2,5-dimethyl-1*H*-pyrrole

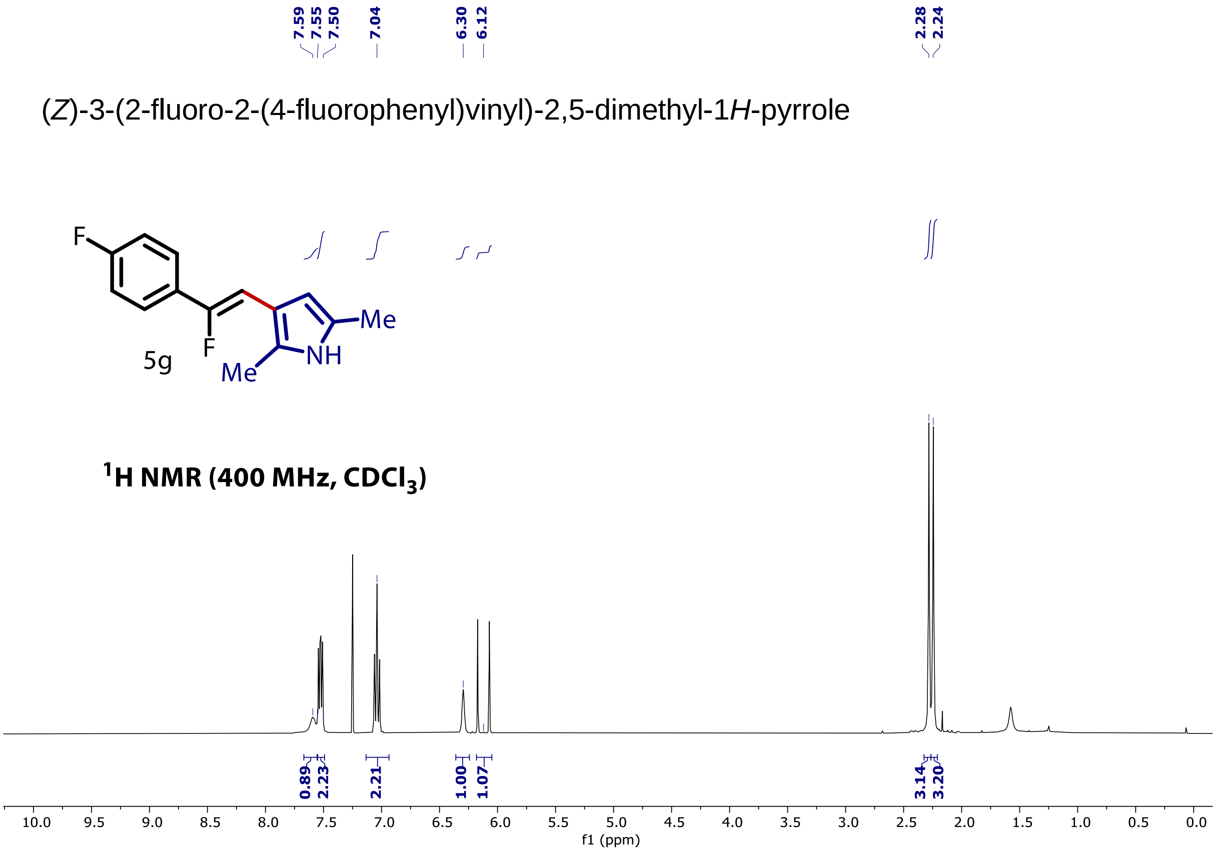

5g

^{1}H NMR (400 MHz, $CDCl_3$)

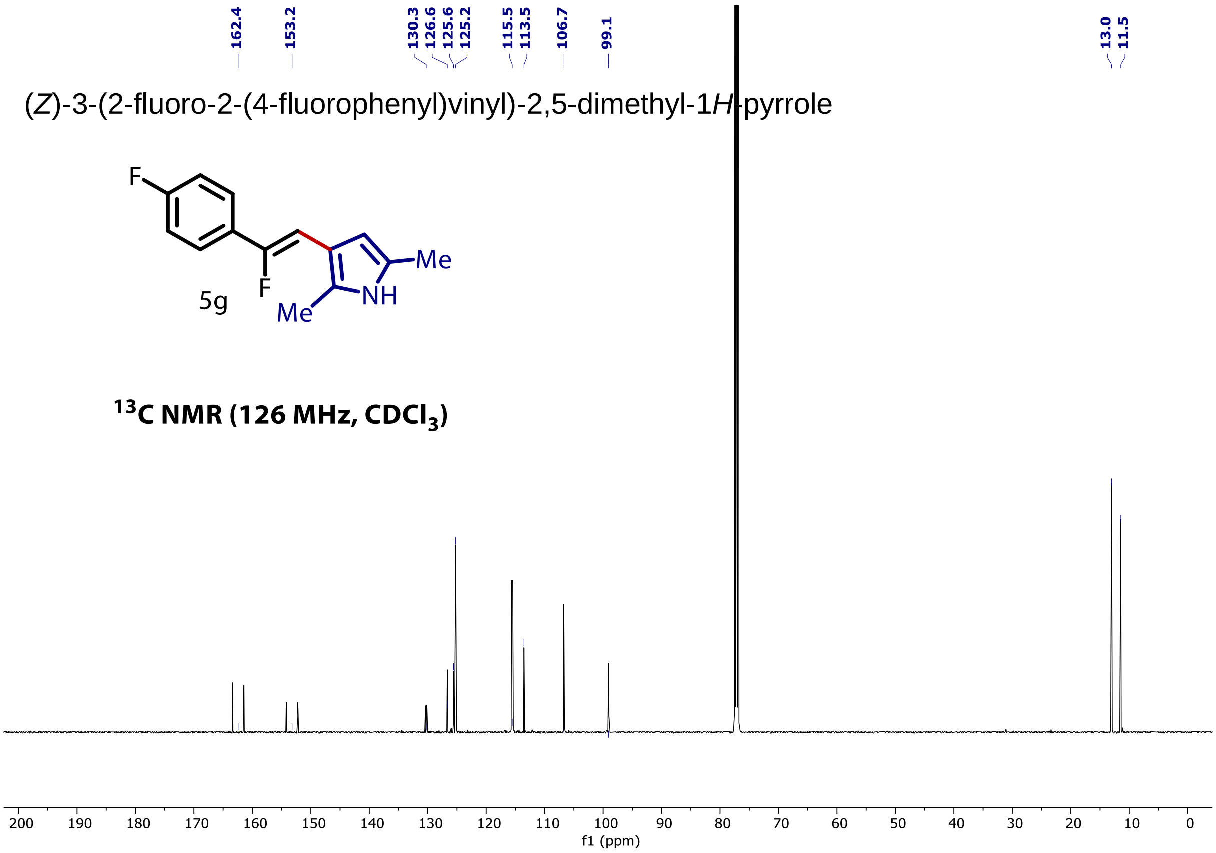
162.4
153.2
130.3
126.6
125.6
125.2
115.5
113.5
106.7
99.1
13.0
11.5
(Z)-3-(2-fluoro-2-(4-fluorophenyl)vinyl)-2,5-dimethyl-1H-pyrrole
F
F
Me
NH
Me
5g
13C NMR (126 MHz, CDCl3)
200 190 180 170 160 150 140 130 120 110 100 90 80 70 60 50 40 30 20 10 0
f1 (ppm)

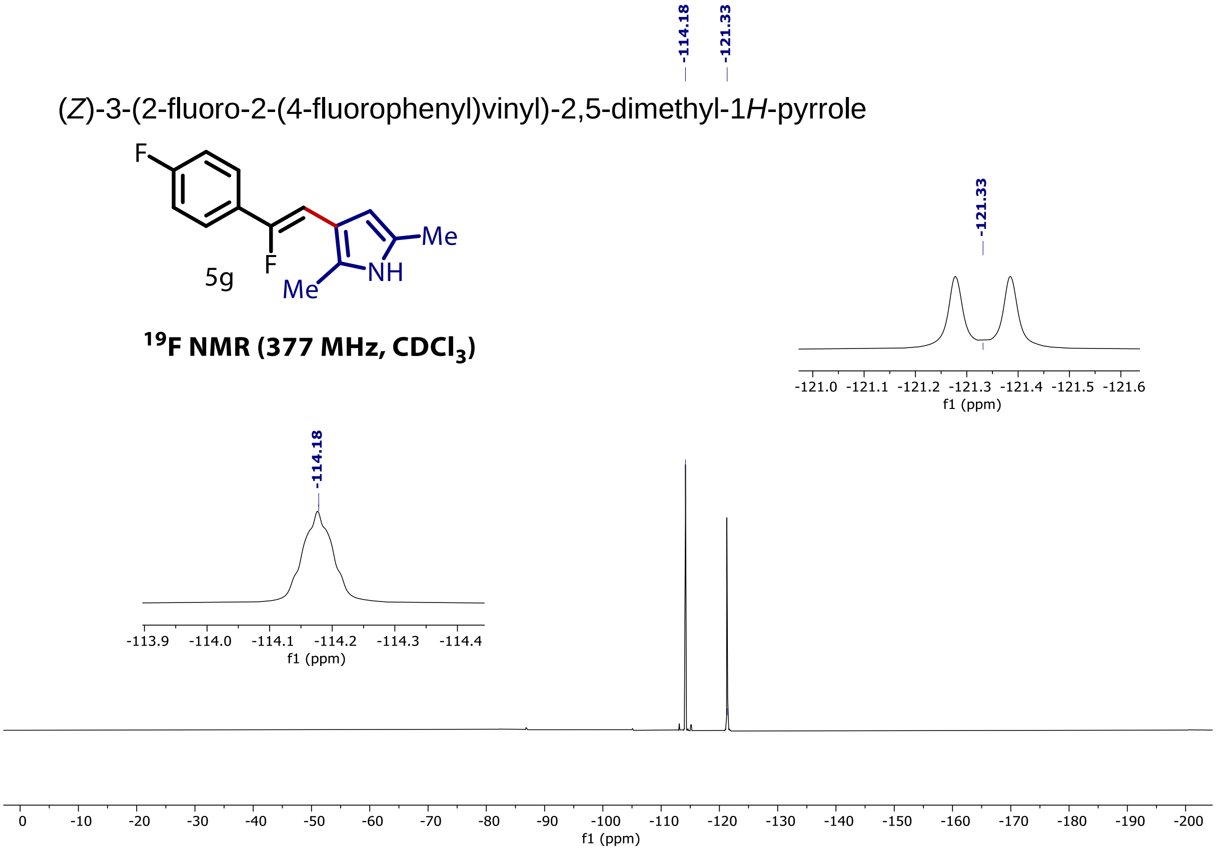
-114.18
-121.33
(Z)-3-(2-fluoro-2-(4-fluorophenyl)vinyl)-2,5-dimethyl-1H-pyrrole
F
F
Me
NH
Me
5g
19F NMR (377 MHz, CDCl3)
-121.33
-121.0 -121.1 -121.2 -121.3 -121.4 -121.5 -121.6
f1 (ppm)
-114.18
-113.9 -114.0 -114.1 -114.2 -114.3 -114.4
f1 (ppm)
0 -10 -20 -30 -40 -50 -60 -70 -80 -90 -100 -110 -120 -130 -140 -150 -160 -170 -180 -190 -200
f1 (ppm)

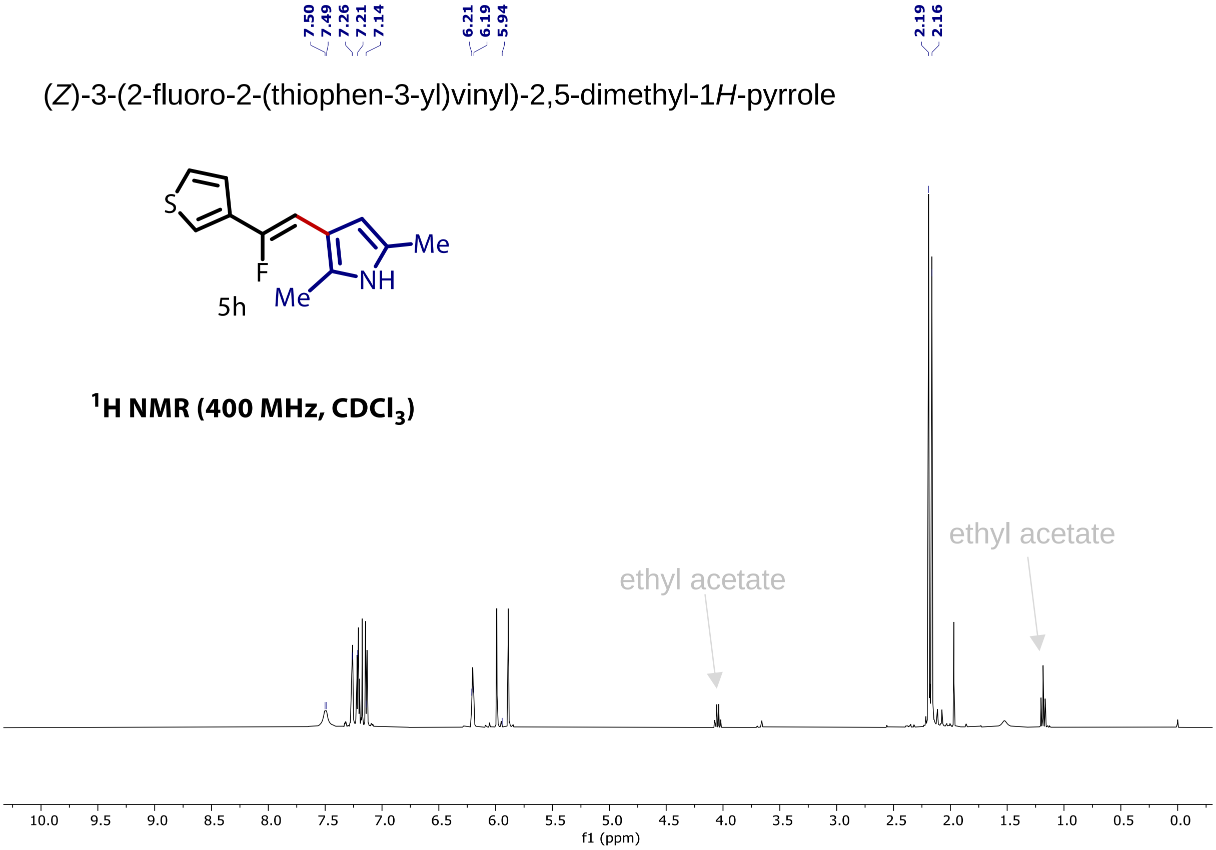
7.50
7.49
7.26
7.21
7.14
6.21
6.19
5.94
2.19
2.16
(Z)-3-(2-fluoro-2-(thiophen-3-yl)vinyl)-2,5-dimethyl-1H-pyrrole
S
F
Me
NH
Me
5h
¹H NMR (400 MHz, CDCl₃)
ethyl acetate
ethyl acetate
10.0 9.5 9.0 8.5 8.0 7.5 7.0 6.5 6.0 5.5 5.0 4.5 4.0 3.5 3.0 2.5 2.0 1.5 1.0 0.5 0.0
f1 (ppm)

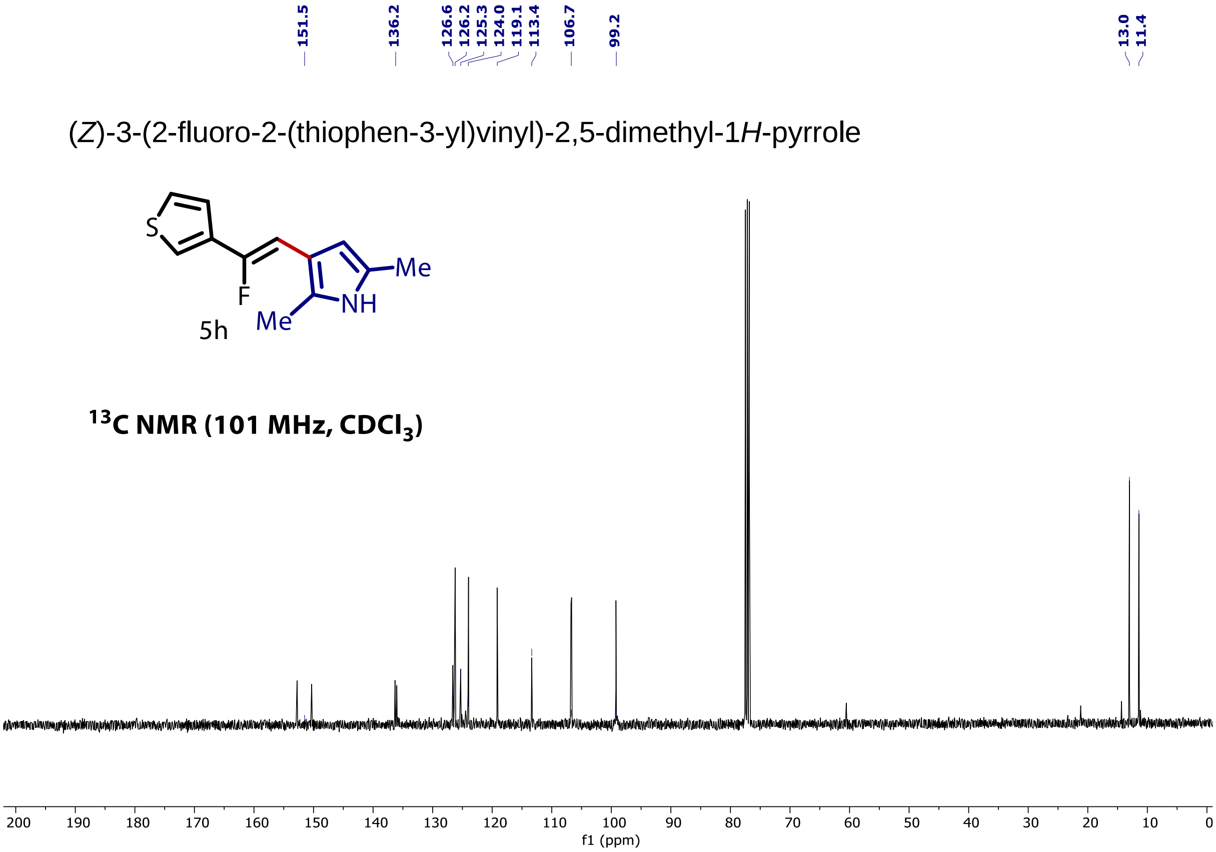
151.5
136.2
126.6
126.2
125.3
124.0
119.1
113.4
106.7
99.2
13.0
11.4
(Z)-3-(2-fluoro-2-(thiophen-3-yl)vinyl)-2,5-dimethyl-1H-pyrrole
S
F
Me
NH
Me
5h
¹³C NMR (101 MHz, CDCl₃)
200 190 180 170 160 150 140 130 120 110 100 90 80 70 60 50 40 30 20 10 0
f1 (ppm)

(*Z*)-3-(2-fluoro-2-(thiophen-3-yl)vinyl)-2,5-dimethyl-1*H*-pyrrole

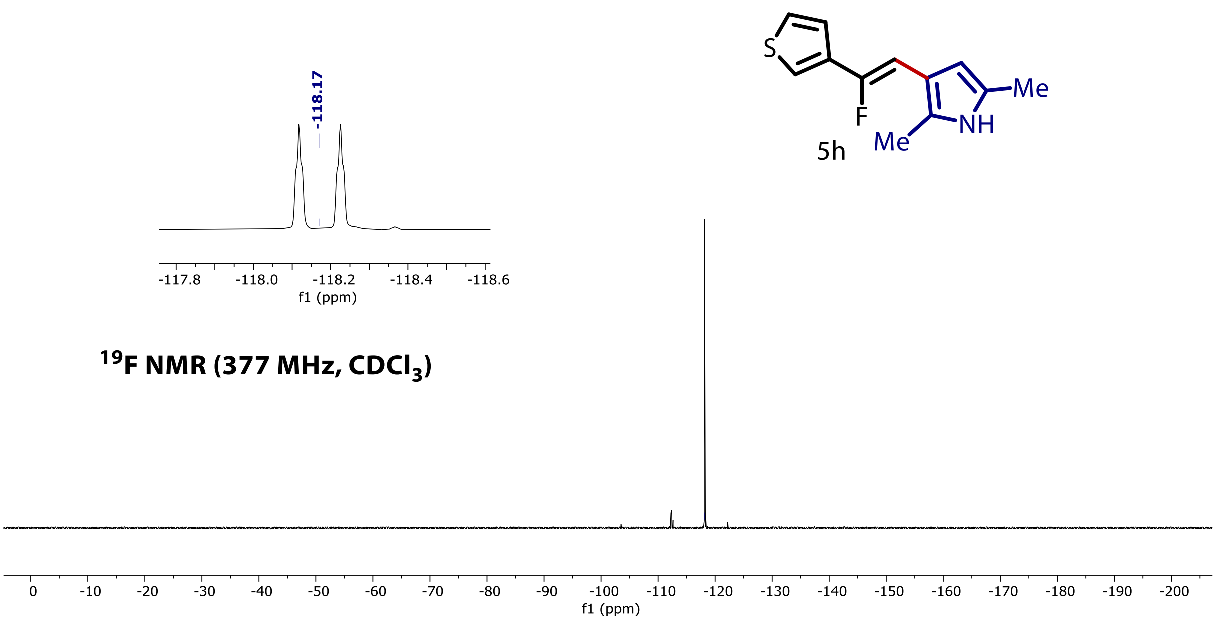

(*E*)-3-(2-fluorododec-1-en-1-yl)-2,5-dimethyl-1*H*-pyrrole

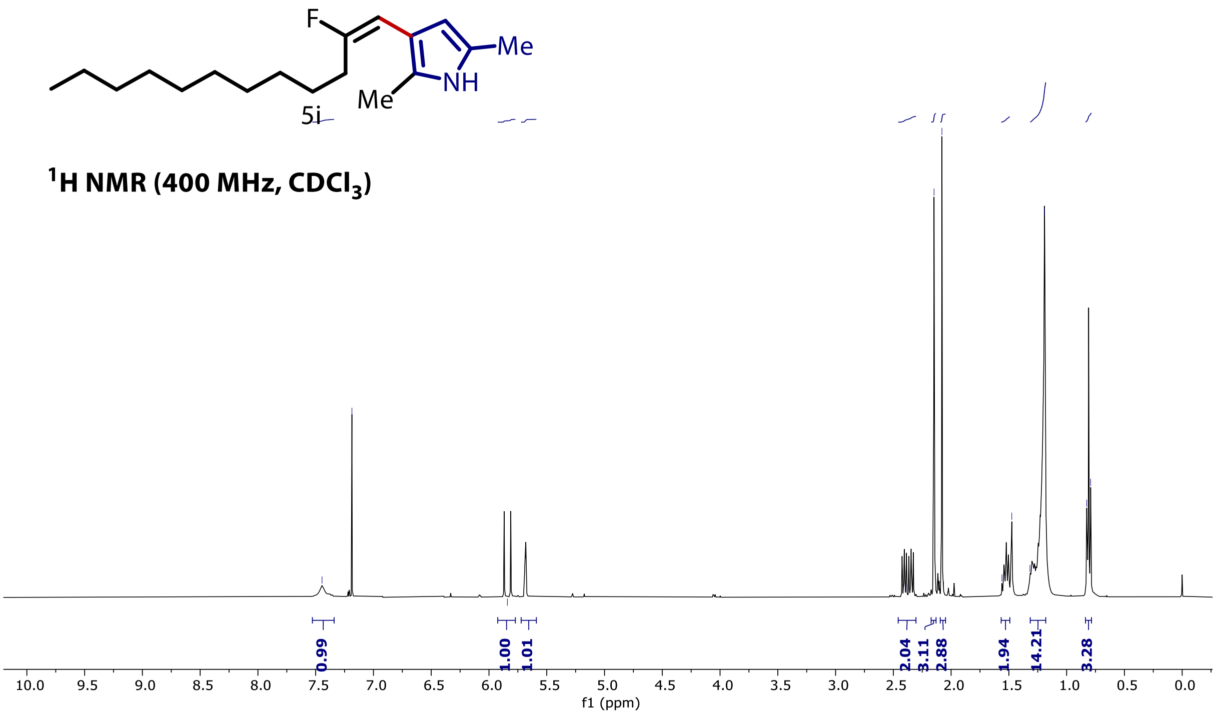

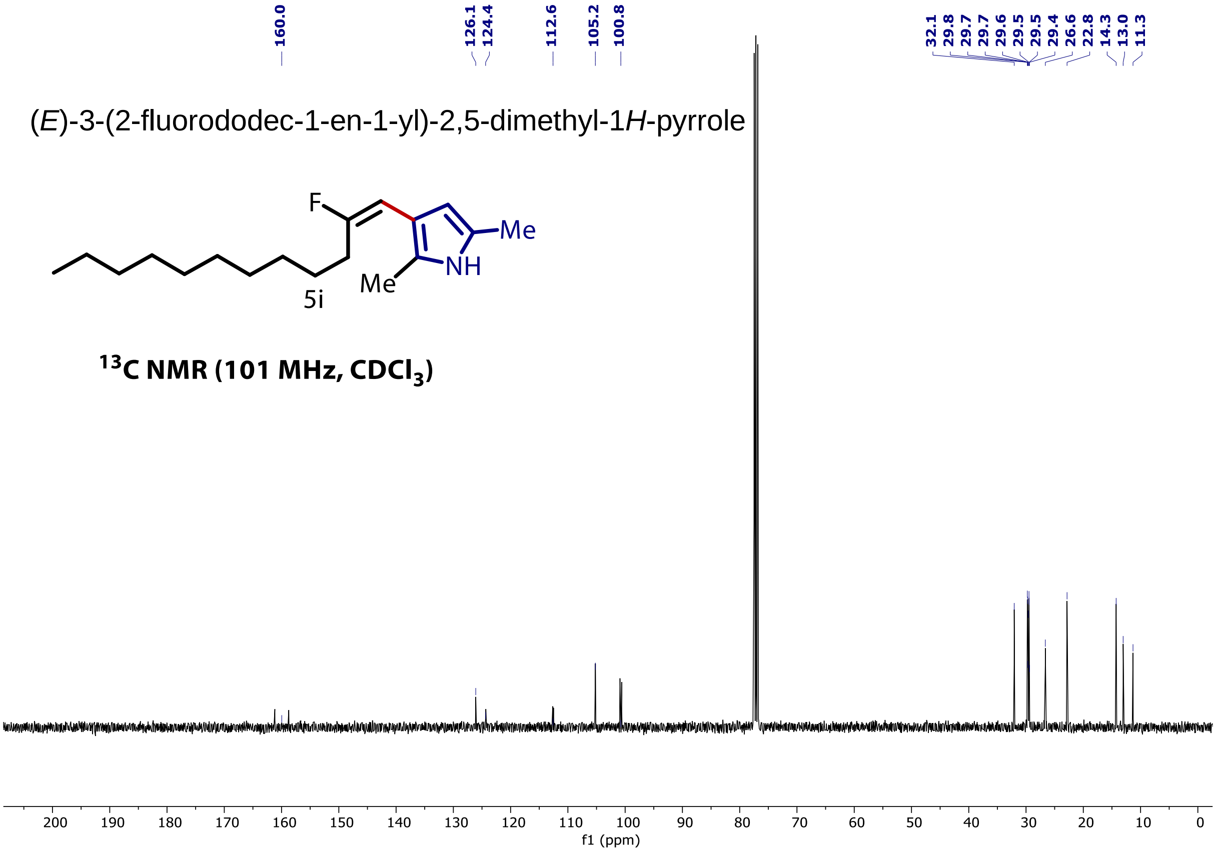
(*E*)-3-(2-fluorododec-1-en-1-yl)-2,5-dimethyl-1*H*-pyrrole
F
Me
NH
Me
5i
13C NMR (101 MHz, CDCl3)
160.0
126.1
124.4
112.6
105.2
100.8
32.1
29.8
29.7
29.7
29.6
29.5
29.5
29.4
26.6
22.8
14.3
13.0
11.3
f1 (ppm)

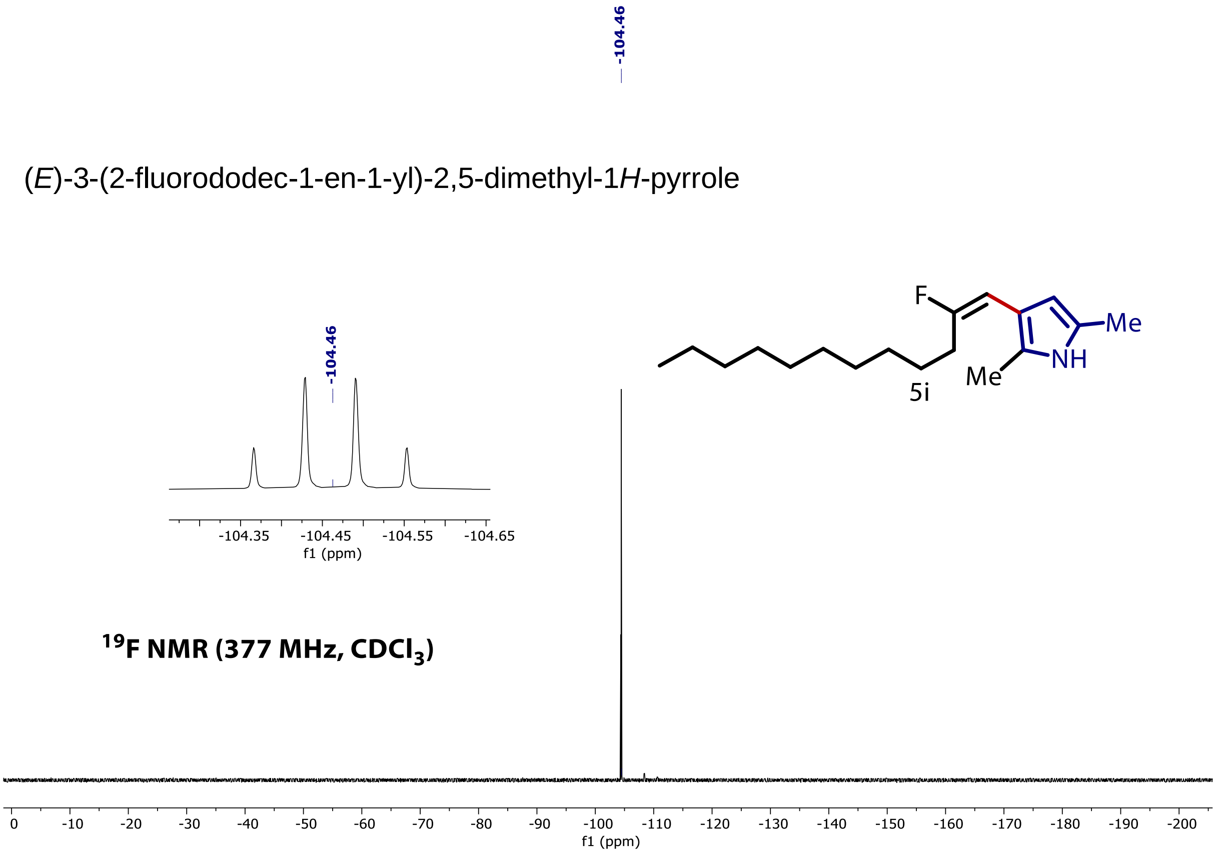
-104.46
(*E*)-3-(2-fluorododec-1-en-1-yl)-2,5-dimethyl-1*H*-pyrrole
-104.46
-104.35
-104.45
-104.55
-104.65
f1 (ppm)
F
Me
NH
Me
5i
19F NMR (377 MHz, CDCl3)
f1 (ppm)

(*Z*)-N-(2-fluoro-3-(1-methyl-1*H*-pyrrolo[2,3-b]pyridin-2-yl)allyl)-*N*-(4-fluorophenyl)-4-methylbenzenesulfonamide

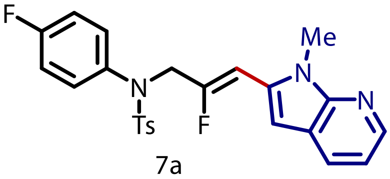

7a

^{1}H NMR (400 MHz, CDCl$_3$)

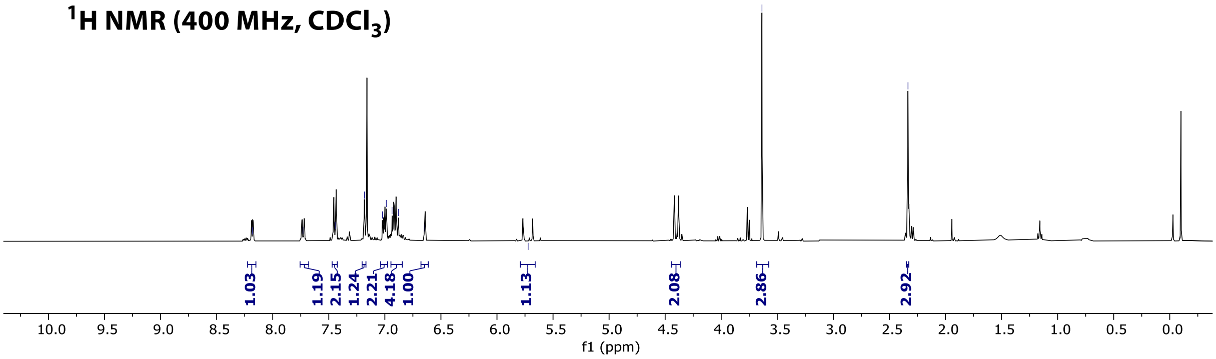

(*Z*)-N-(2-fluoro-3-(1-methyl-1*H*-pyrrolo[2,3-b]pyridin-2-yl)allyl)-*N*-(4-fluorophenyl)-4-methylbenzenesulfonamide

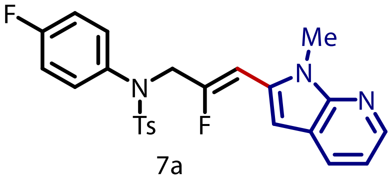

7a

^{13}C NMR (126 MHz, CDCl$_3$)

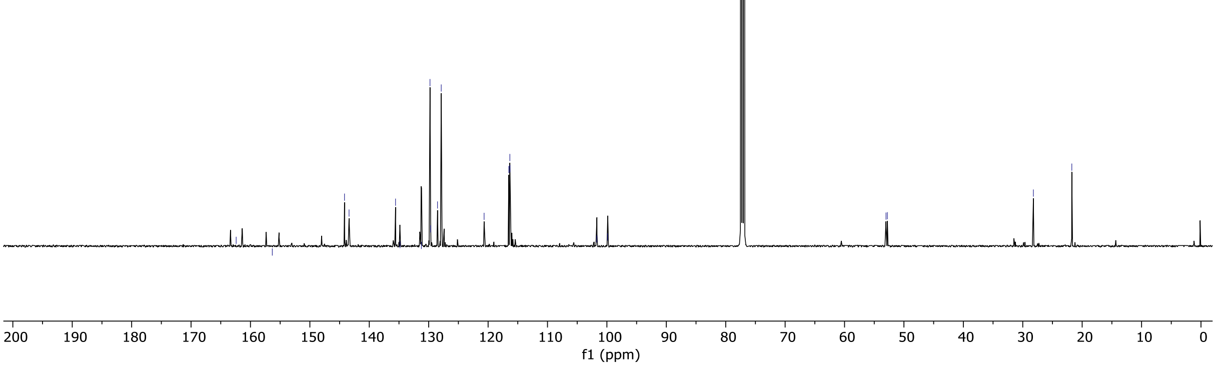

(*Z*)-N-(2-fluoro-3-(1-methyl-1*H*-pyrrolo[2,3-b]pyridin-2-yl)allyl)-*N*-(4-fluorophenyl)-4-methylbenzenesulfonamide

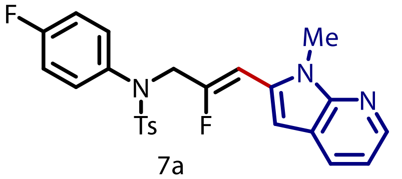

7a

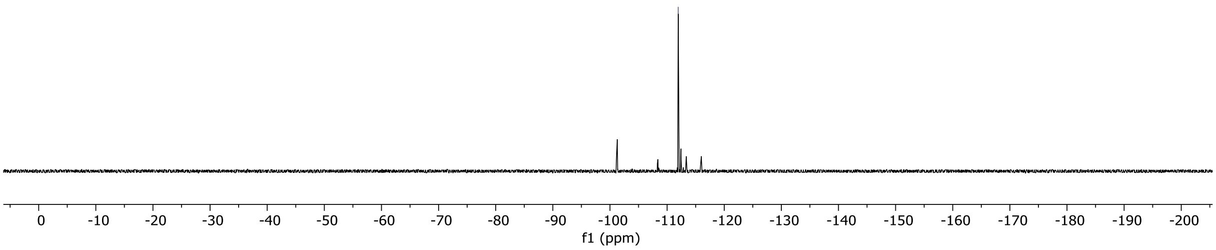

^{19}F NMR (377 MHz, $CDCl_3$)

(*Z*)-2-(2-fluoro-5-phenylpent-1-en-1-yl)-1,3,5-trimethoxybenzene

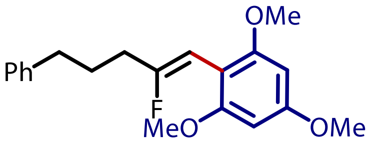

9a

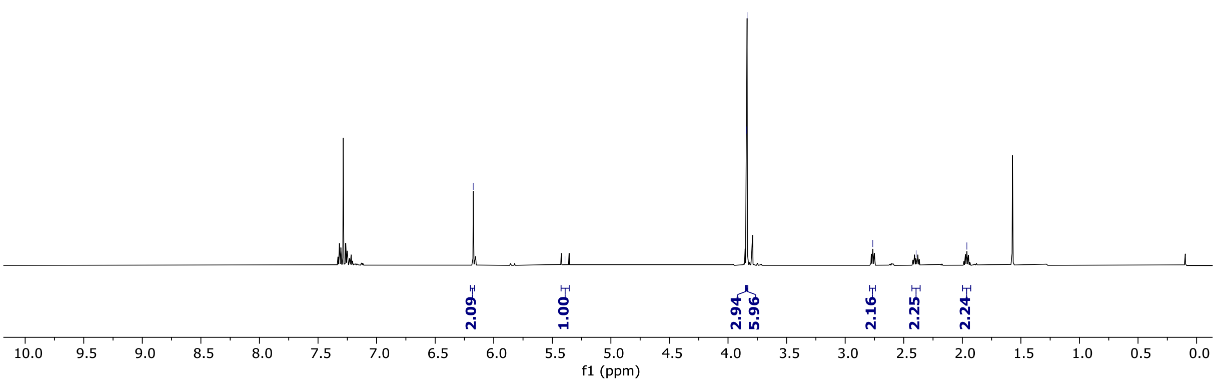

^{1}H NMR (600 MHz, $CDCl_3$)

(*Z*)-2-(2-fluoro-5-phenylpent-1-en-1-yl)-1,3,5-trimethoxybenzene

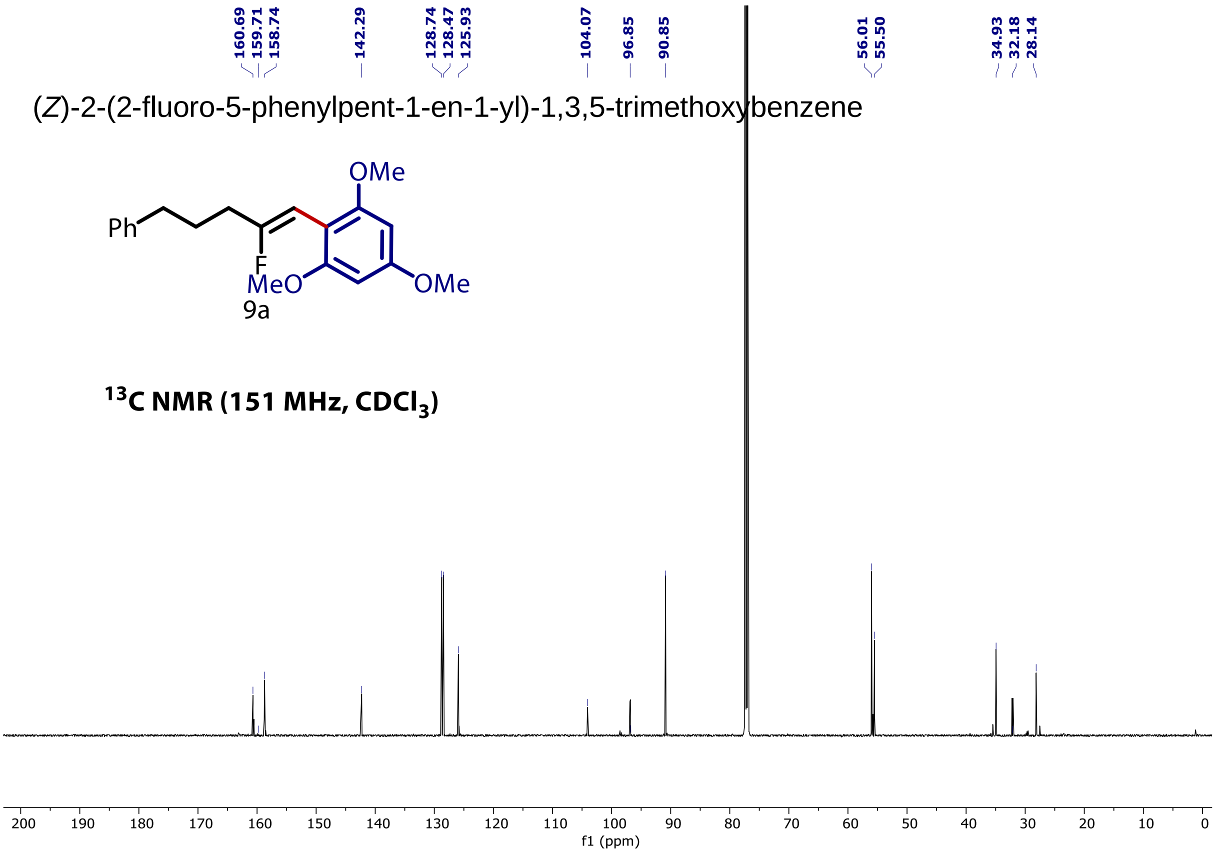

^{13}C NMR (151 MHz, $CDCl_3$)

(*Z*)-2-(2-fluoro-5-phenylpent-1-en-1-yl)-1,3,5-trimethoxybenzene

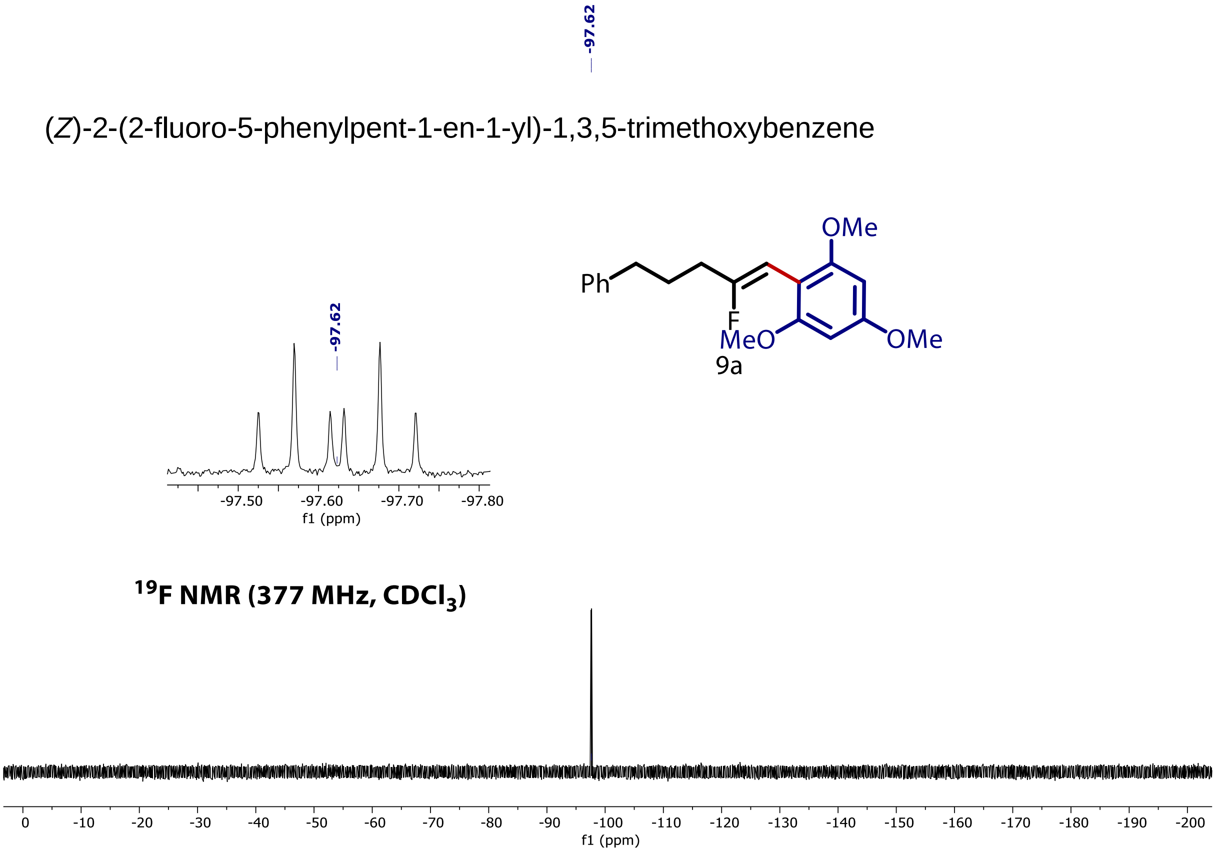

^{19}F NMR (377 MHz, $CDCl_3$)

(*E*)-2-(4-fluorostyryl)-1-methyl-1*H*-indole

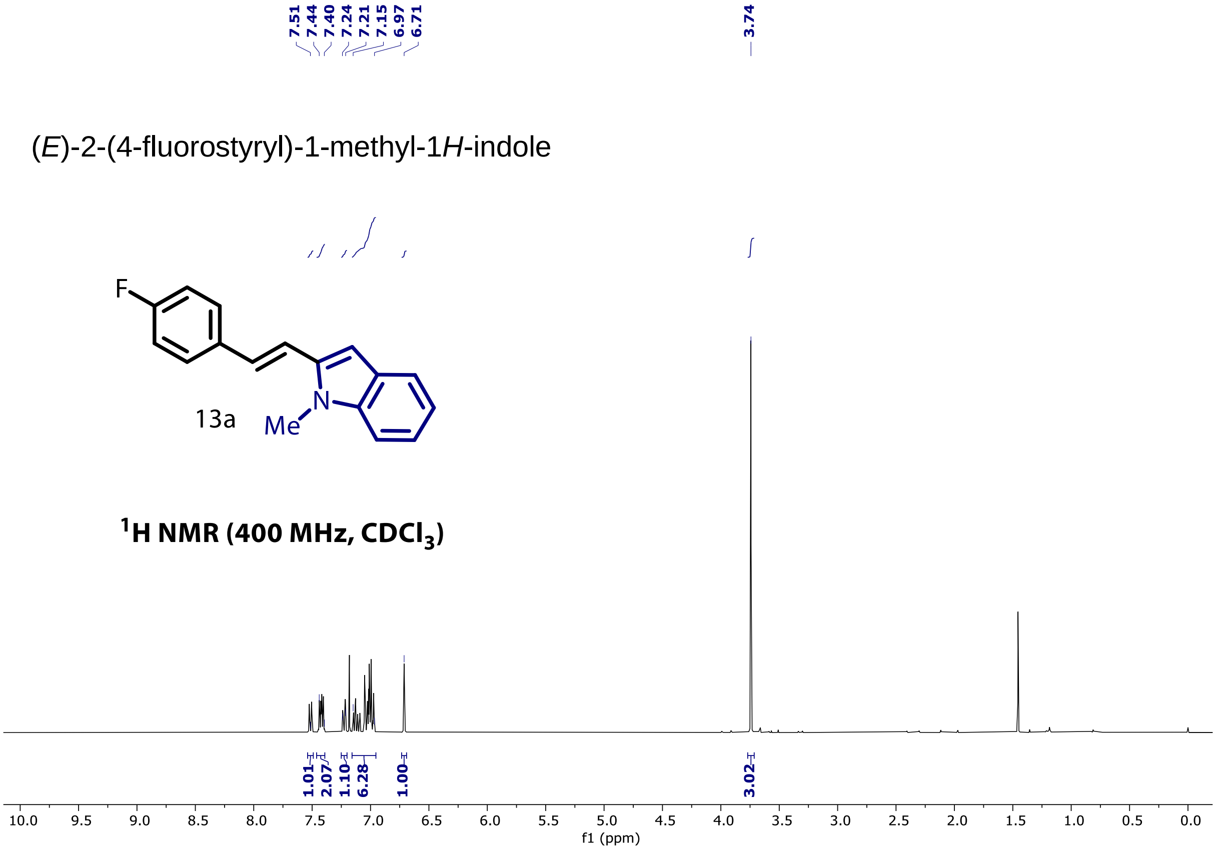

(*E*)-2-(4-fluorostyryl)-1-methyl-1*H*-indole

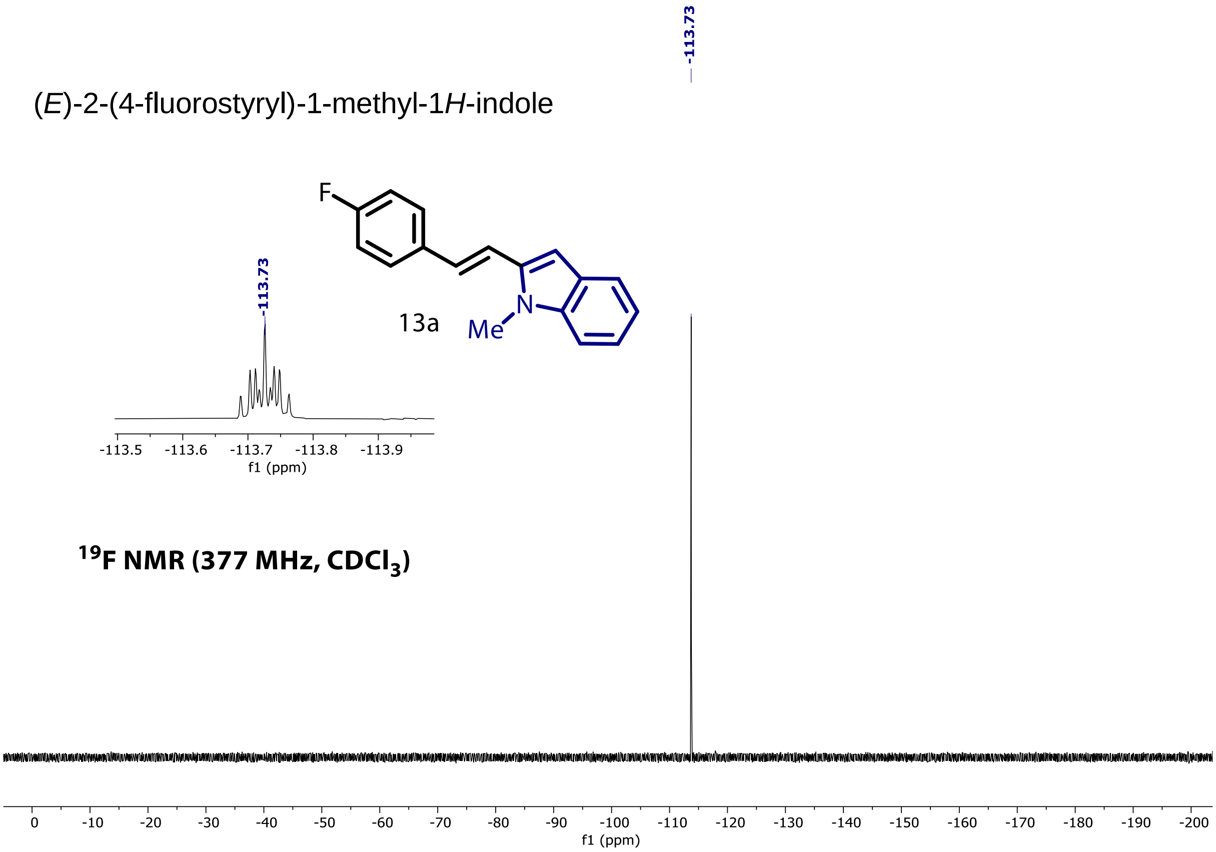

1-(1-Methyl-1*H*-indol-2-yl)-5-phenylpentan-2-one

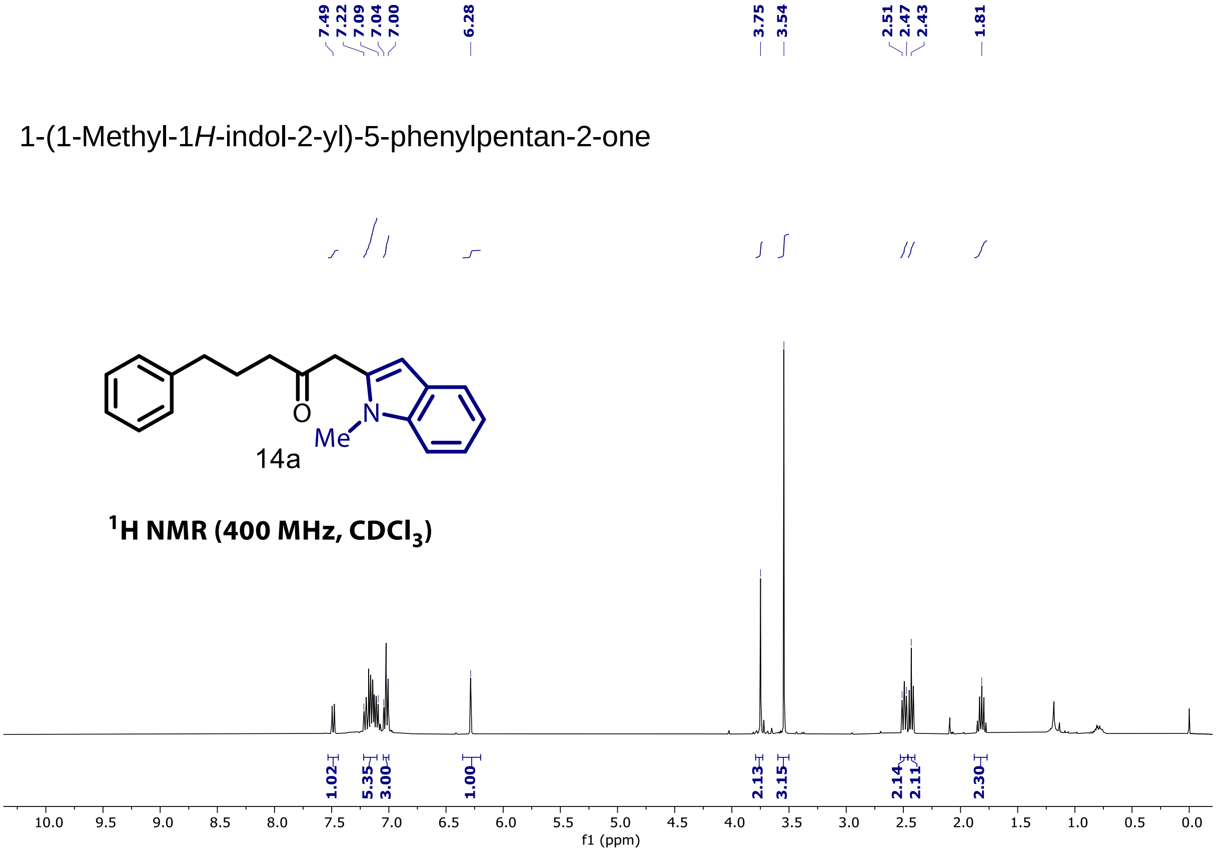

1-(1-Methyl-1*H*-indol-2-yl)-5-phenylpentan-2-one

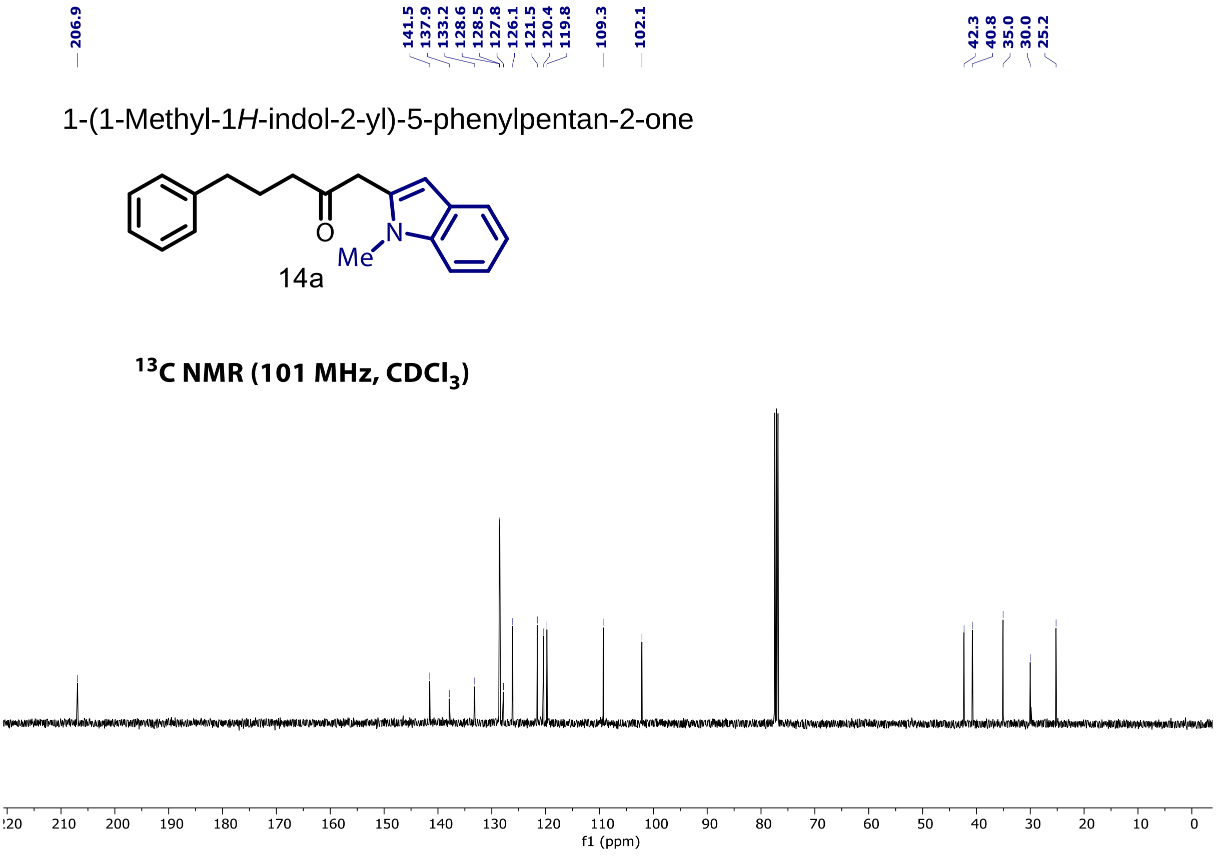

1-(1-Methyl-1*H*-indol-2-yl)dodecan-2-one

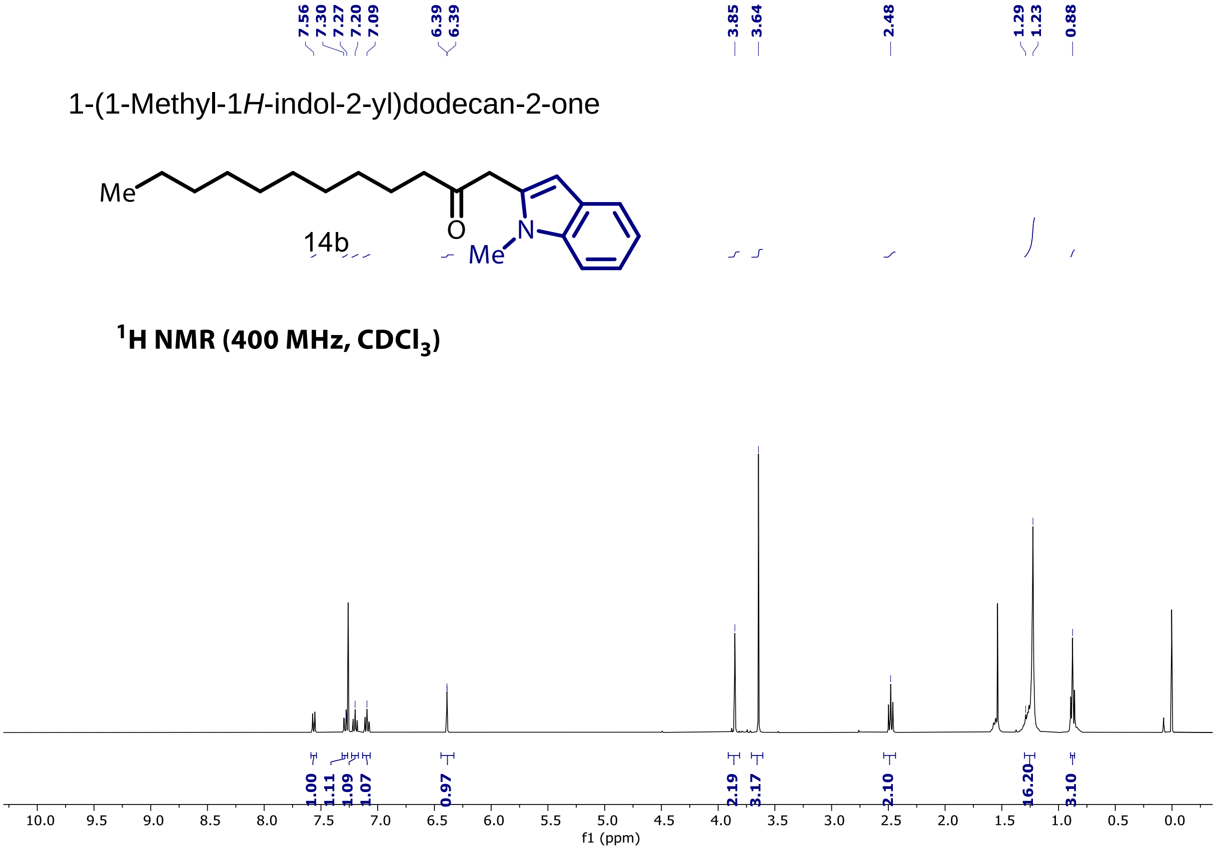

^{1}H NMR (400 MHz, $CDCl_3$)

1-(1-Methyl-1*H*-indol-2-yl)dodecan-2-one

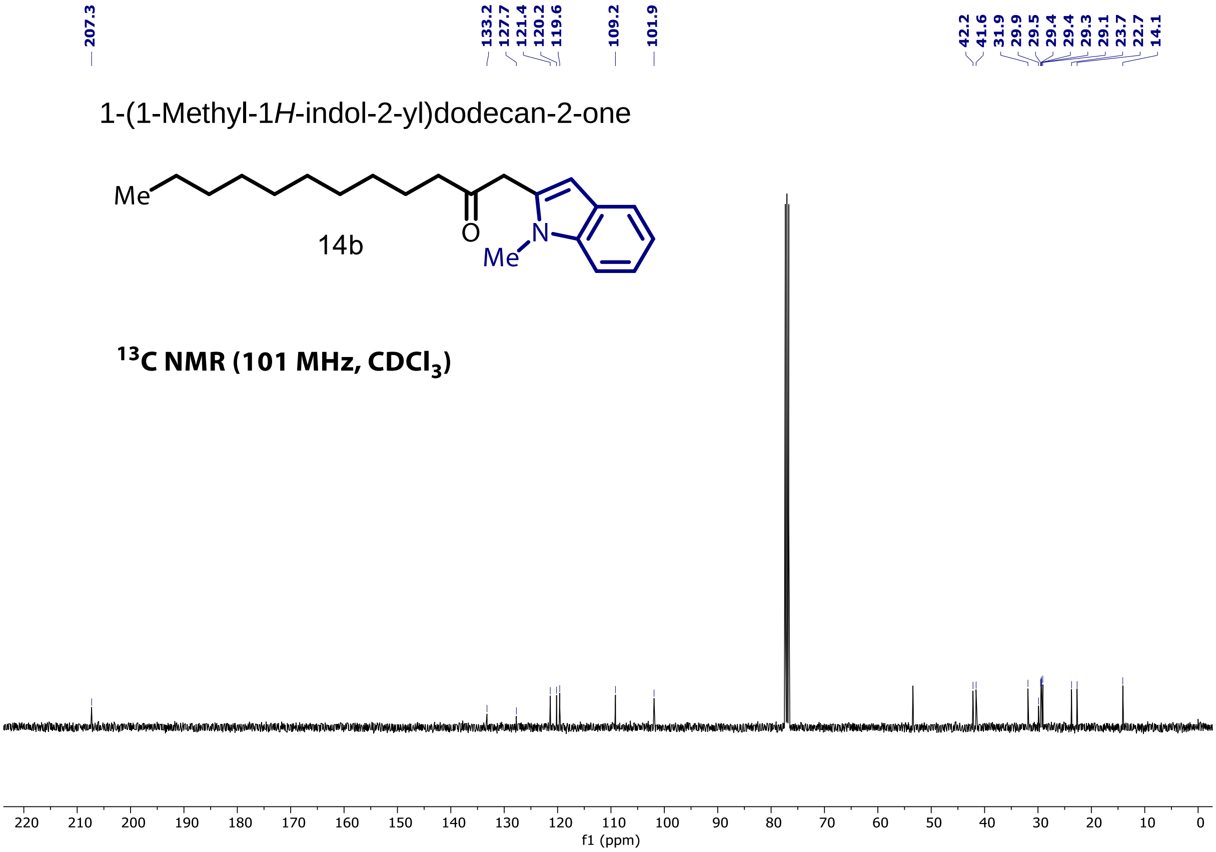

^{13}C NMR (101 MHz, $CDCl_3$)

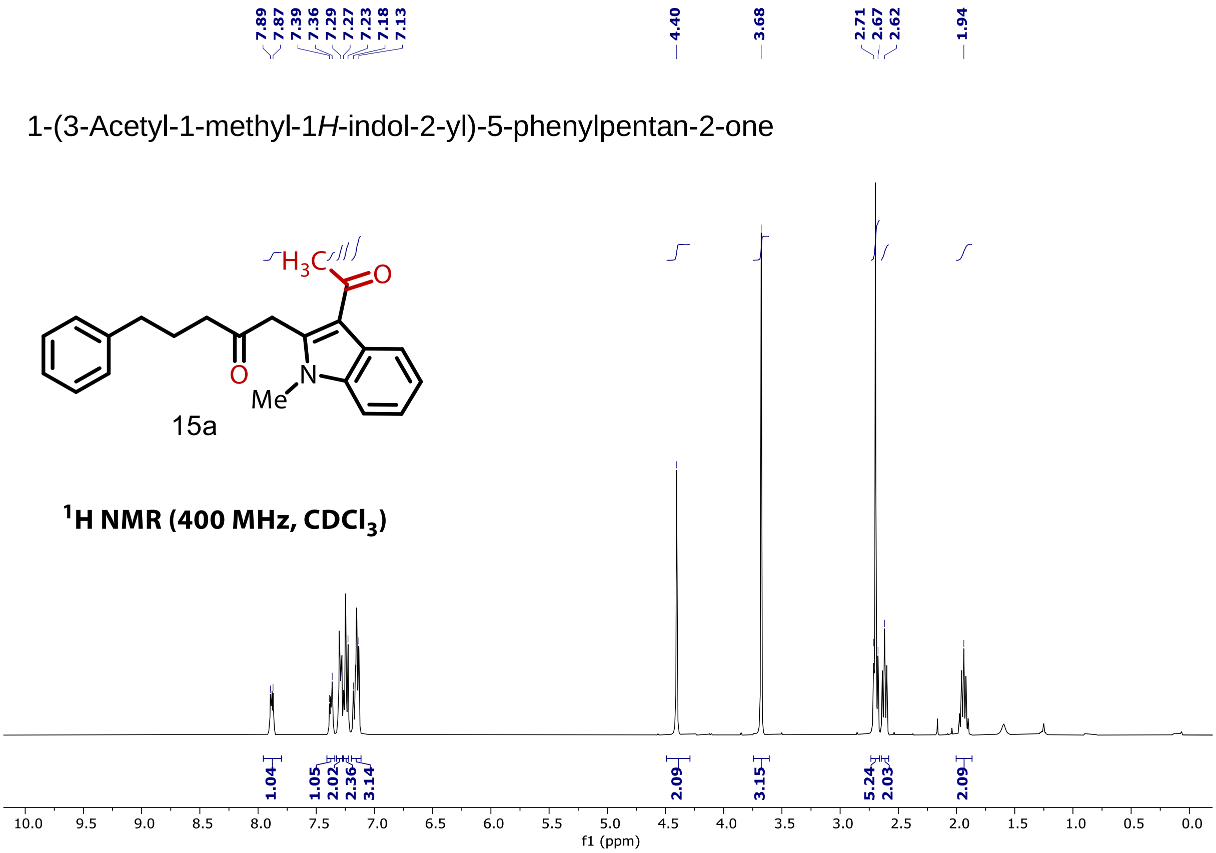
7.89 7.87 7.39 7.36 7.29 7.27 7.23 7.18 7.13
4.40
3.68
2.71 2.67 2.62
1.94
1-(3-Acetyl-1-methyl-1H-indol-2-yl)-5-phenylpentan-2-one
H3C
O
O
N
Me
15a
1H NMR (400 MHz, CDCl3)
1.04
1.05 2.02 2.36 3.14
2.09
3.15
5.24 2.03
2.09
10.0 9.5 9.0 8.5 8.0 7.5 7.0 6.5 6.0 5.5 5.0 4.5 4.0 3.5 3.0 2.5 2.0 1.5 1.0 0.5 0.0
f1 (ppm)

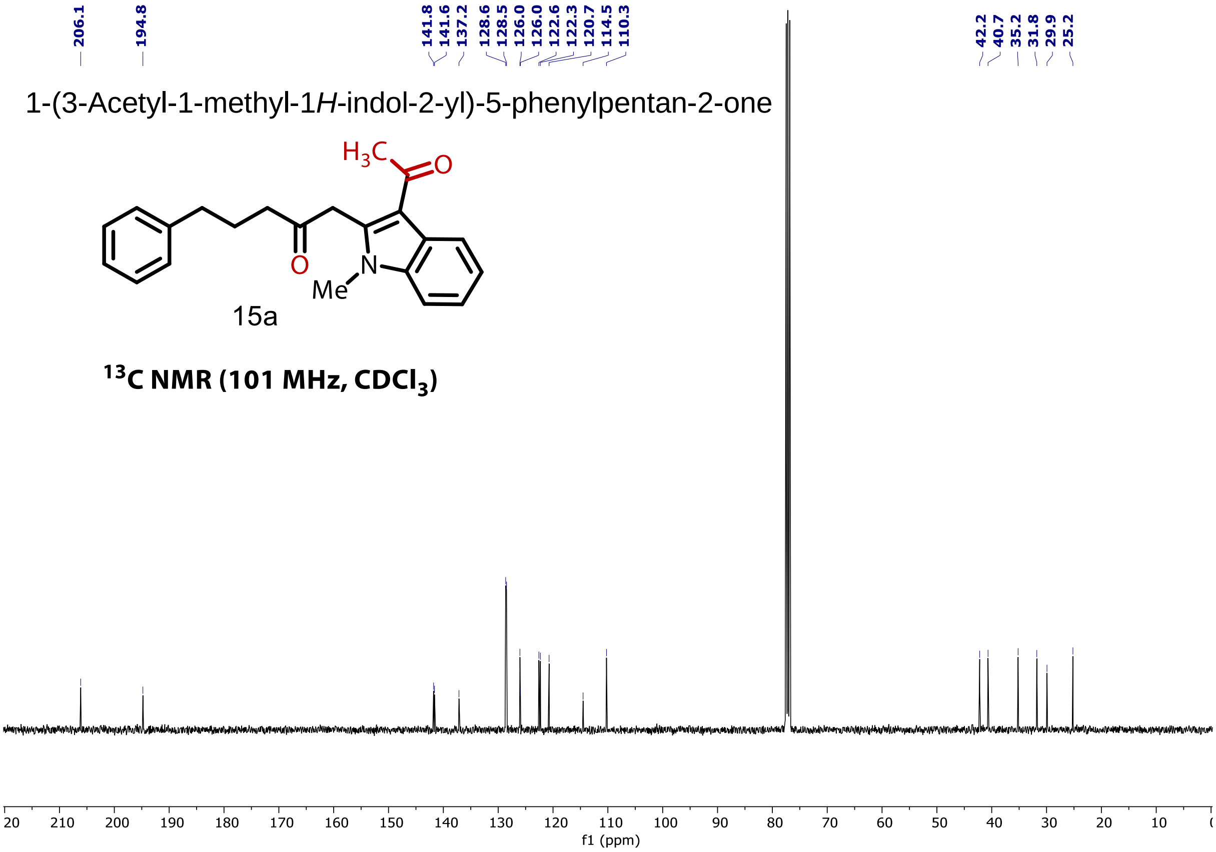
206.1
194.8
141.8 141.6 137.2 128.6 128.5 126.0 122.6 122.3 120.7 114.5 110.3
42.2 40.7 35.2 31.8 29.9 25.2
1-(3-Acetyl-1-methyl-1H-indol-2-yl)-5-phenylpentan-2-one
H3C
O
O
N
Me
15a
13C NMR (101 MHz, CDCl3)
210 200 190 180 170 160 150 140 130 120 110 100 90 80 70 60 50 40 30 20 10
f1 (ppm)

1-(1-Methyl-3-(2,2,2-trifluoroacetyl)-1*H*-indol-2-yl)-5-phenylpentan-2-one

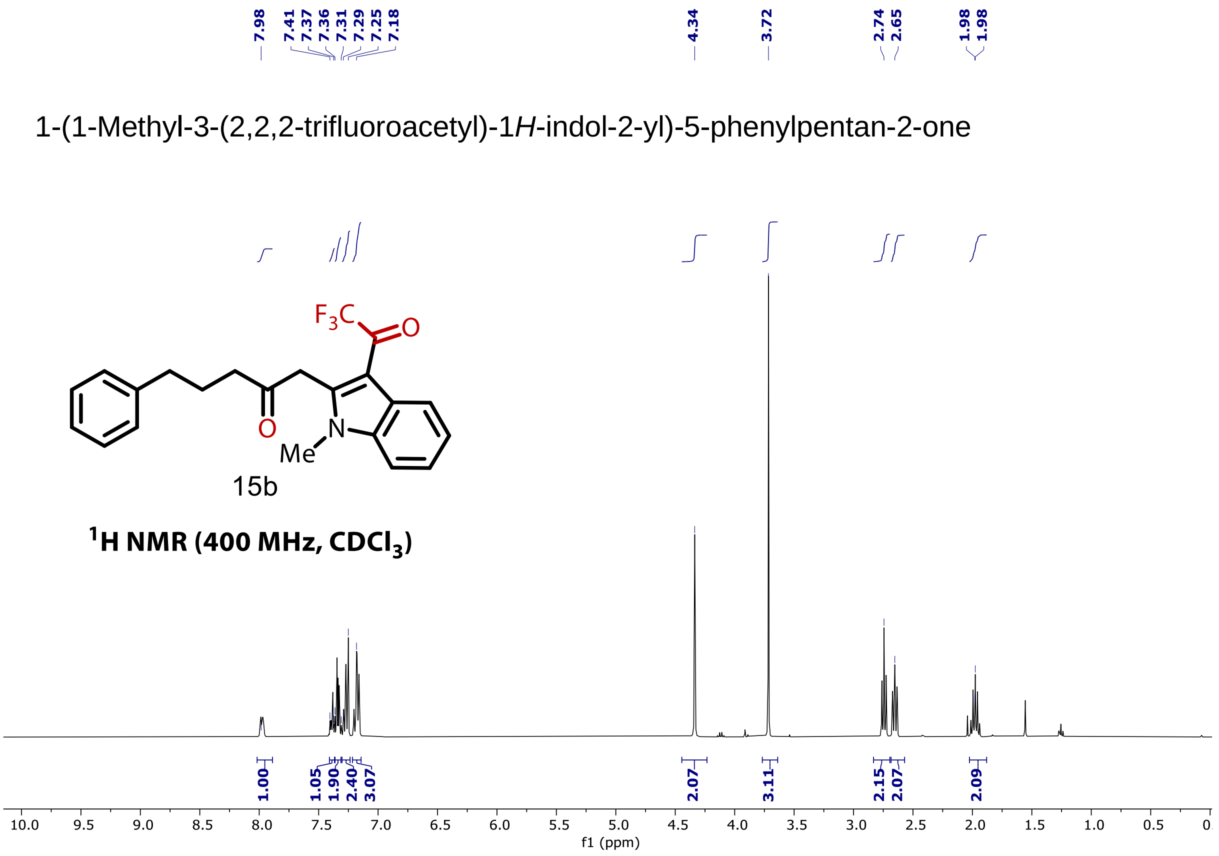

1-(1-Methyl-3-(2,2,2-trifluoroacetyl)-1*H*-indol-2-yl)-5-phenylpentan-2-one

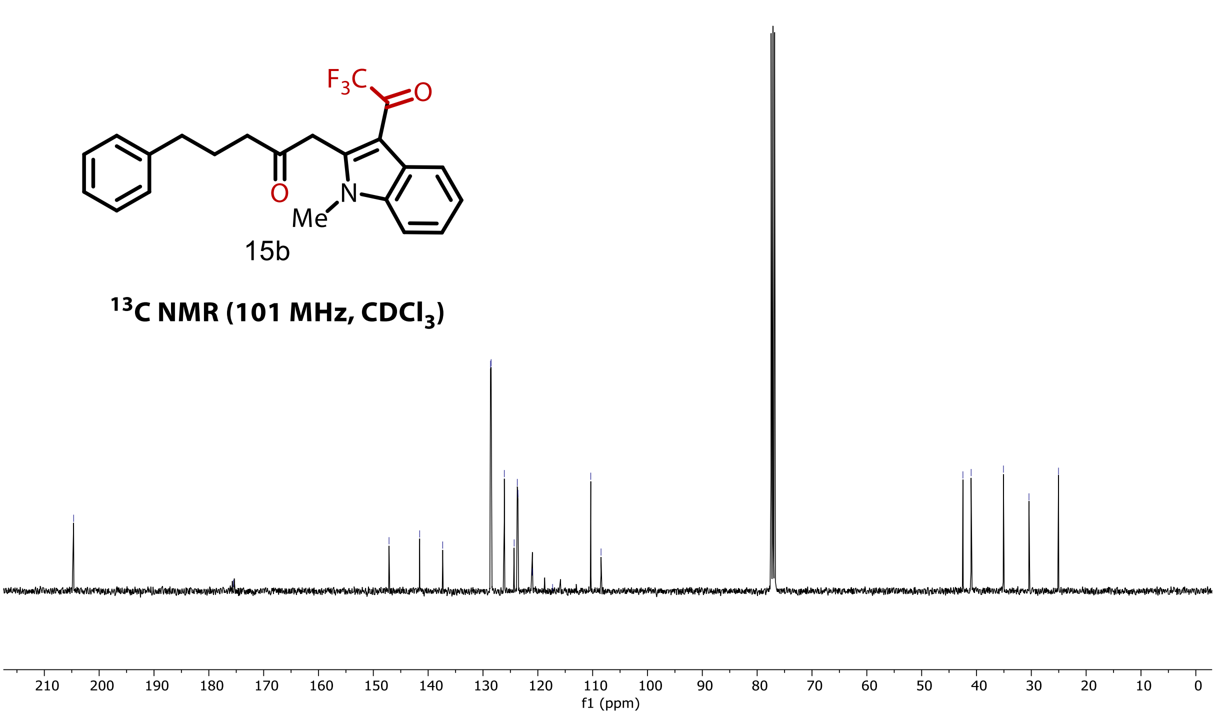

1-(1-Methyl-3-(2,2,2-trifluoroacetyl)-1H-indol-2-yl)-5-phenylpentan-2-one

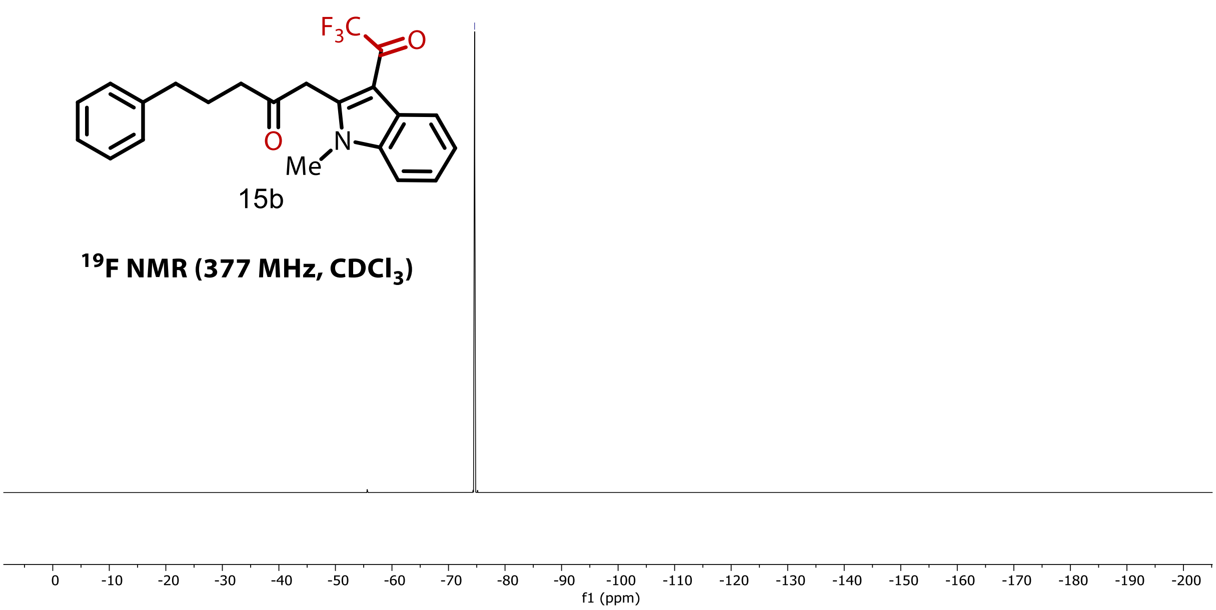

1-(3-(2,2-Difluoro-2-phenylacetyl)-1-methyl-1H-indol-2-yl)-5-phenylpentan-2-one

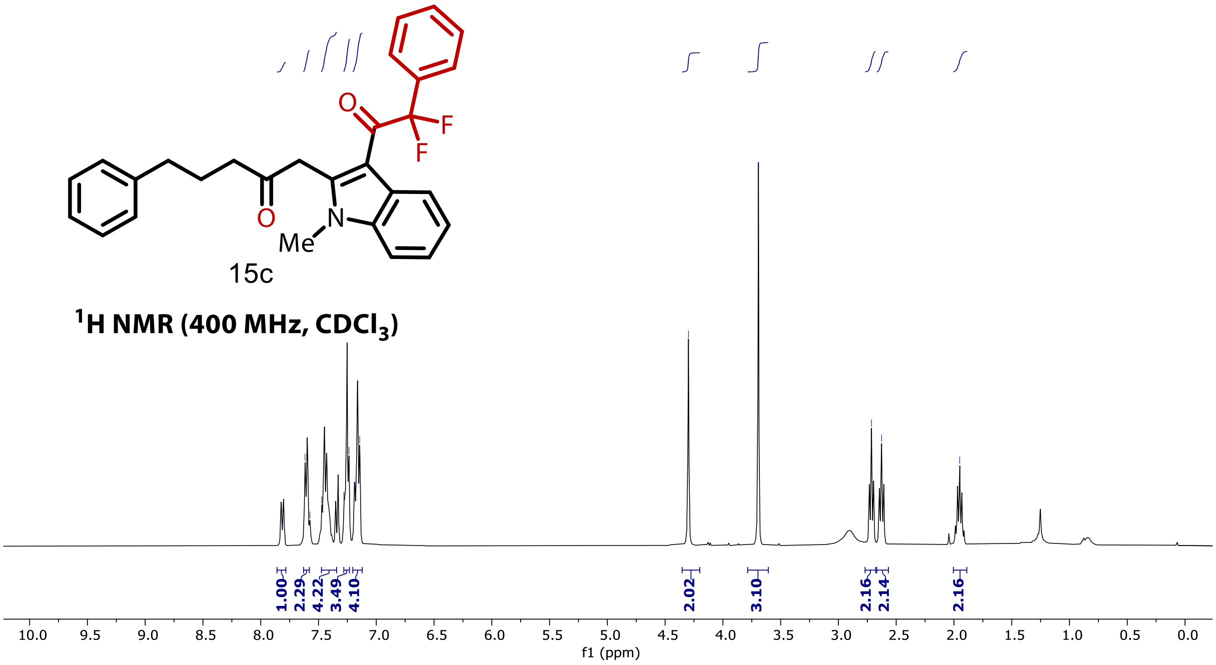

1-(3-(2,2-Difluoro-2-phenylacetyl)-1-methyl-1*H*-indol-2-yl)-5-phenylpentan-2-one

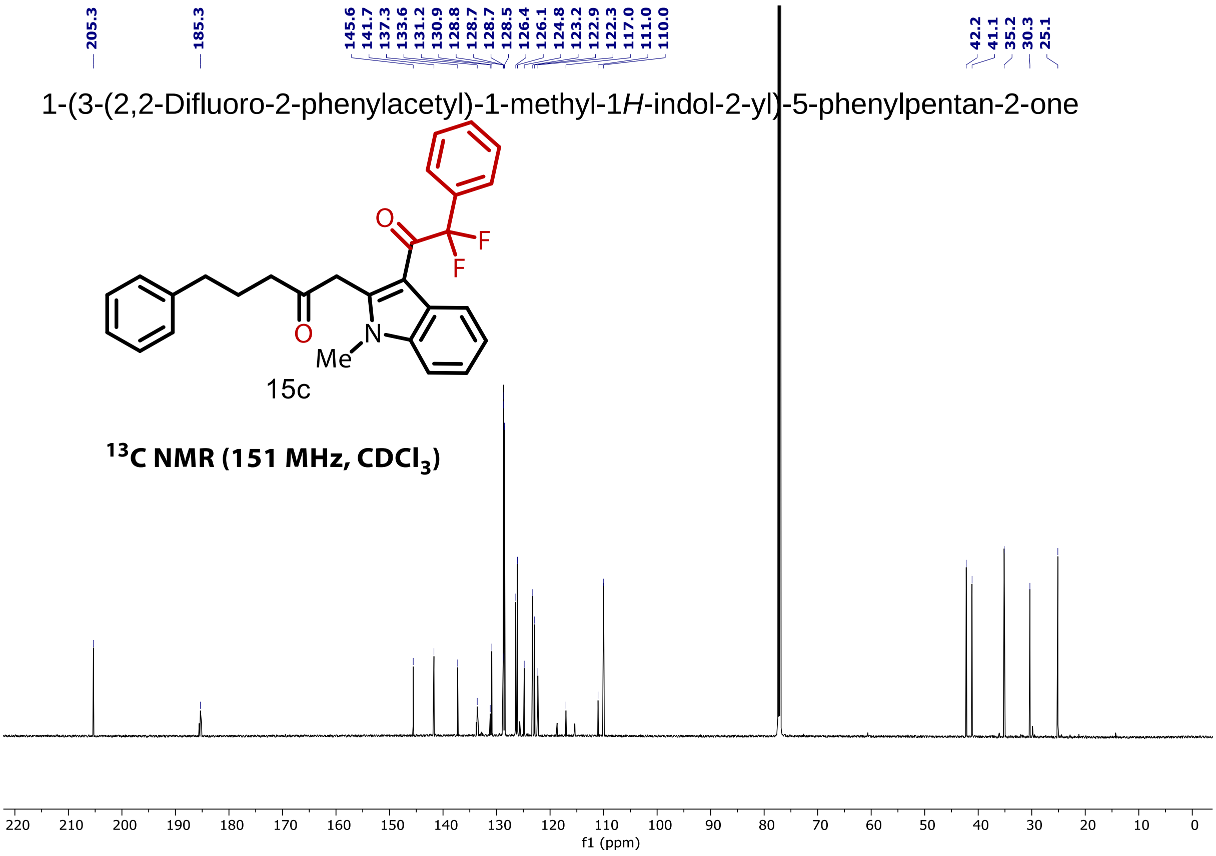

1-(3-(2,2-Difluoro-2-phenylacetyl)-1-methyl-1*H*-indol-2-yl)-5-phenylpentan-2-one

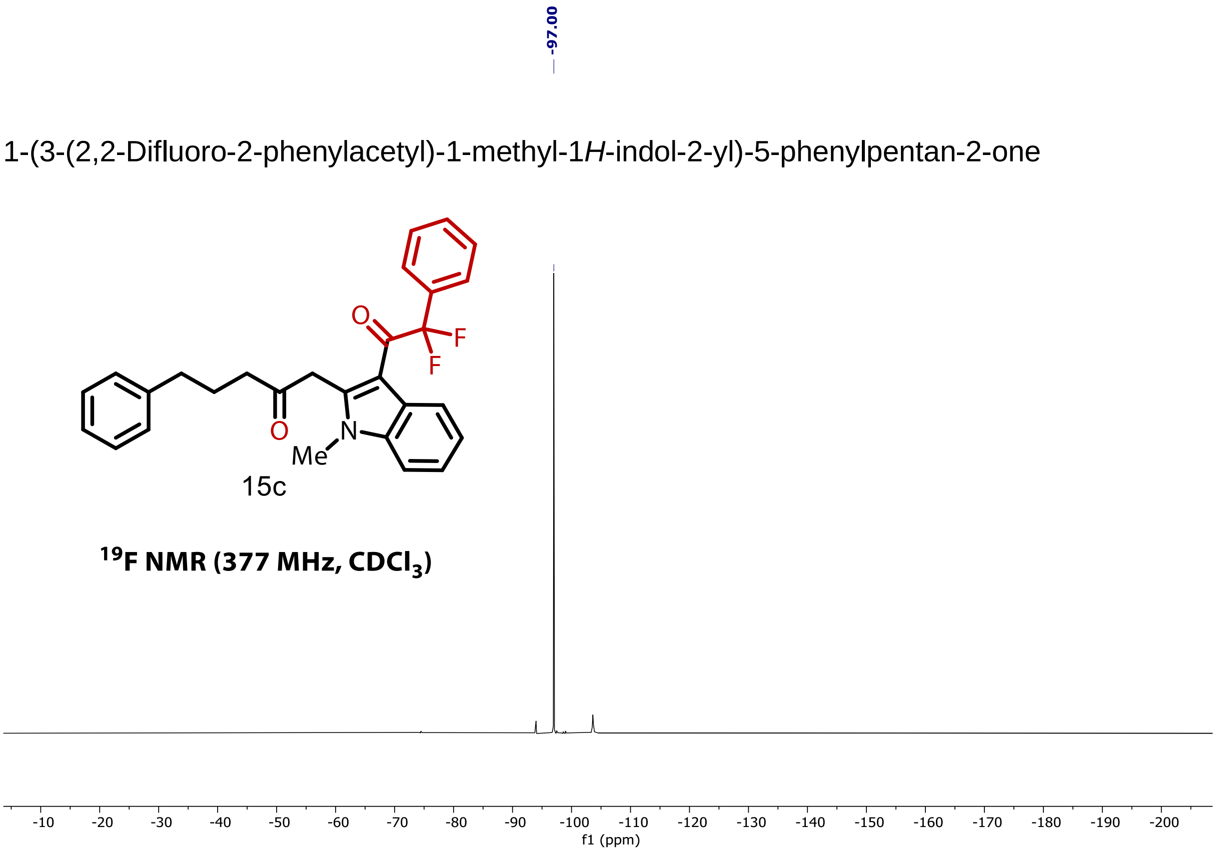

1-(3-(2,2-Difluoroacetyl)-1-methyl-1*H*-indol-2-yl)-5-phenylpentan-2-one

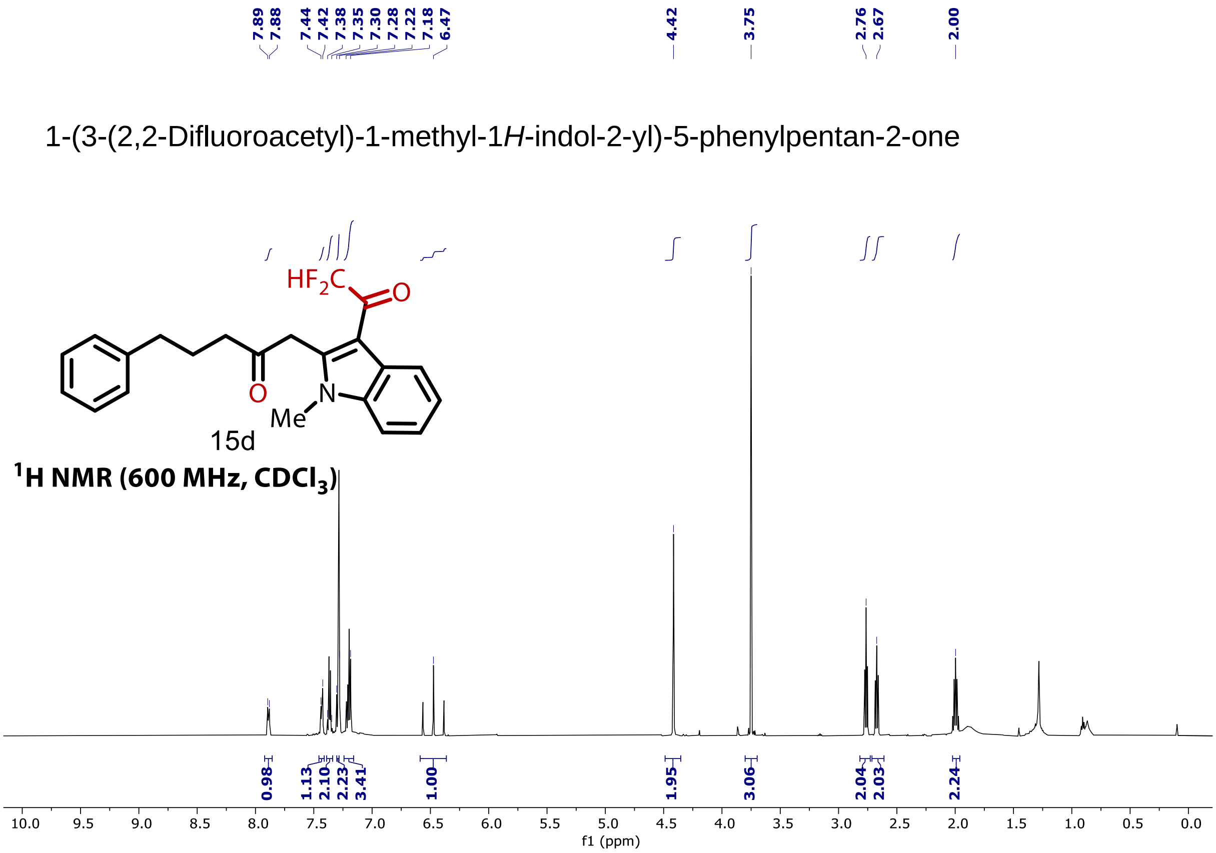

1-(3-(2,2-Difluoroacetyl)-1-methyl-1*H*-indol-2-yl)-5-phenylpentan-2-one

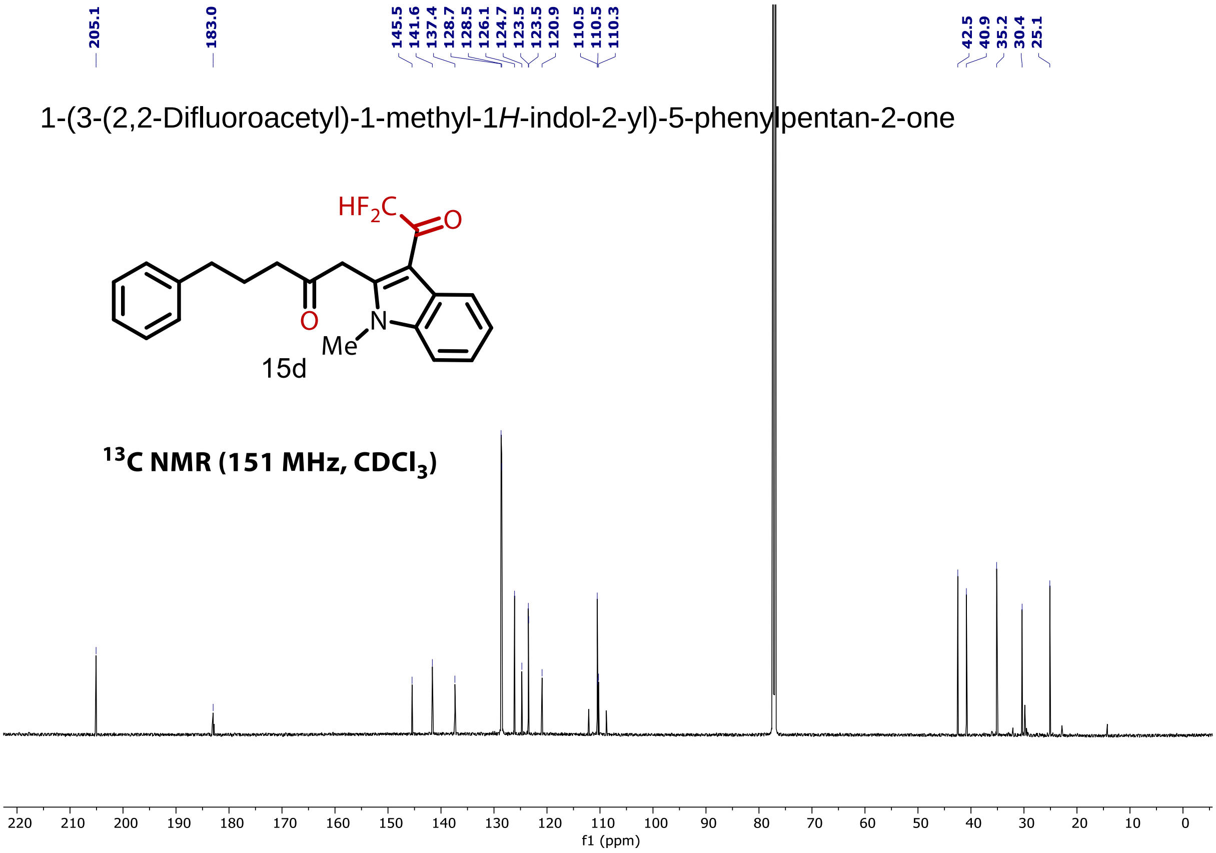

1-(3-(2,2-Difluoroacetyl)-1-methyl-1*H*-indol-2-yl)-5-phenylpentan-2-one

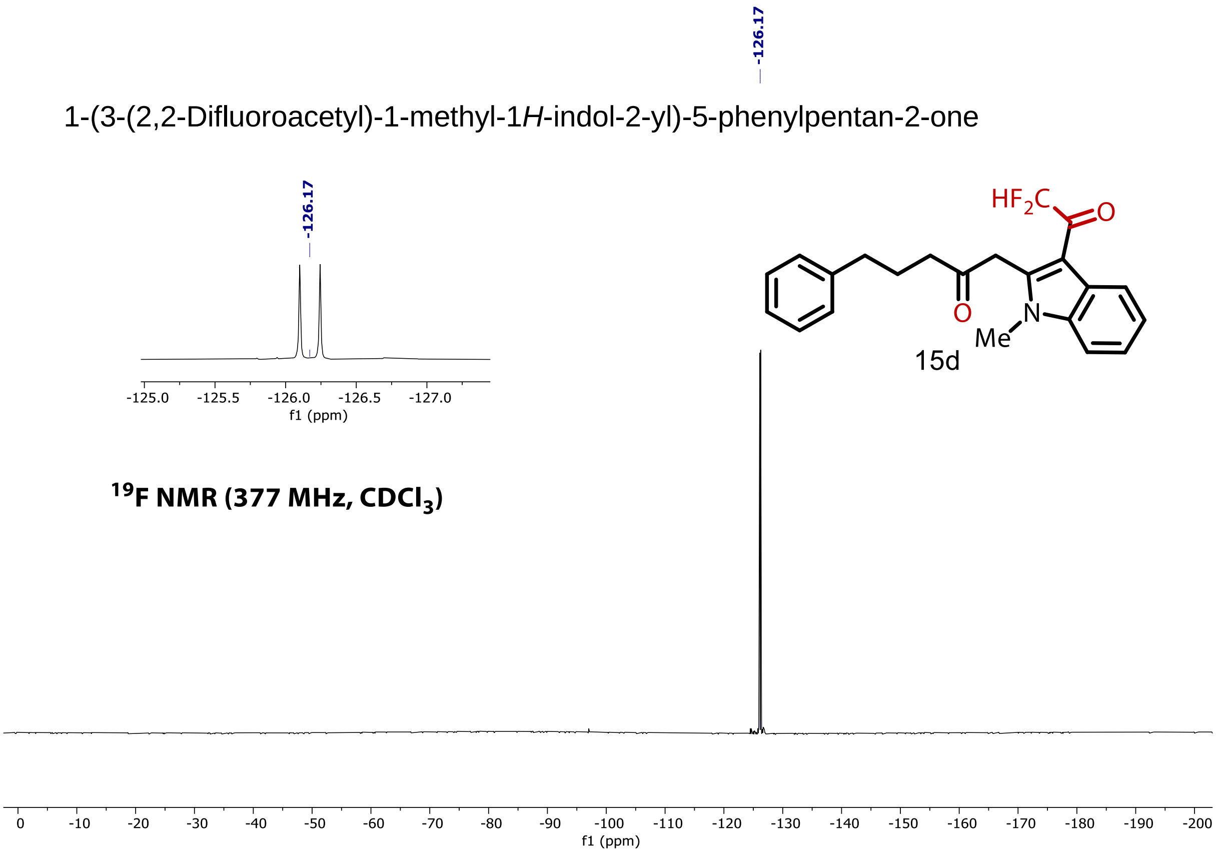

15d

^{19}F NMR (377 MHz, $CDCl_3$)

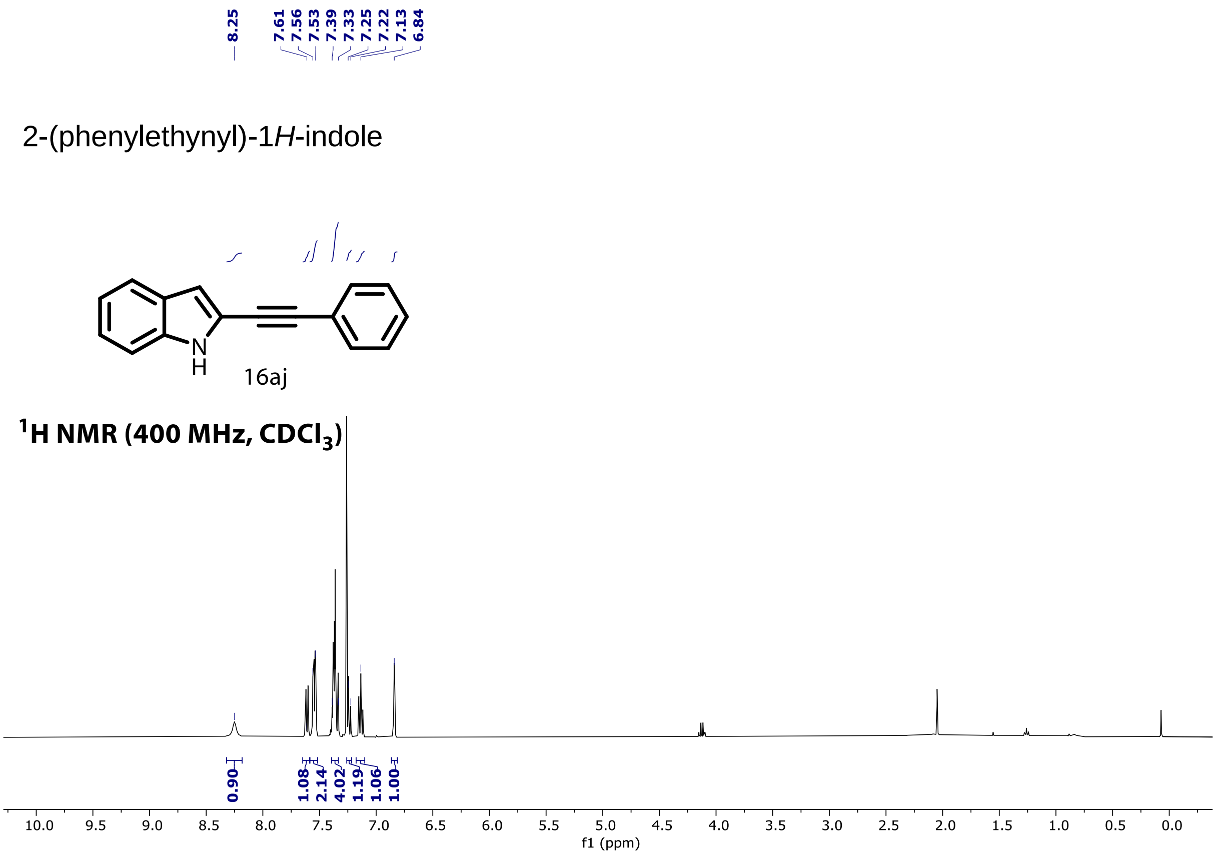
8.25
7.61
7.56
7.53
7.39
7.33
7.25
7.22
7.13
6.84
2-(phenylethynyl)-1H-indole
N
H
16aj
1H NMR (400 MHz, CDCl3)
0.90
1.08
2.14
4.02
1.19
1.06
1.00
10.0
9.5
9.0
8.5
8.0
7.5
7.0
6.5
6.0
5.5
5.0
4.5
4.0
3.5
3.0
2.5
2.0
1.5
1.0
0.5
0.0
f1 (ppm)

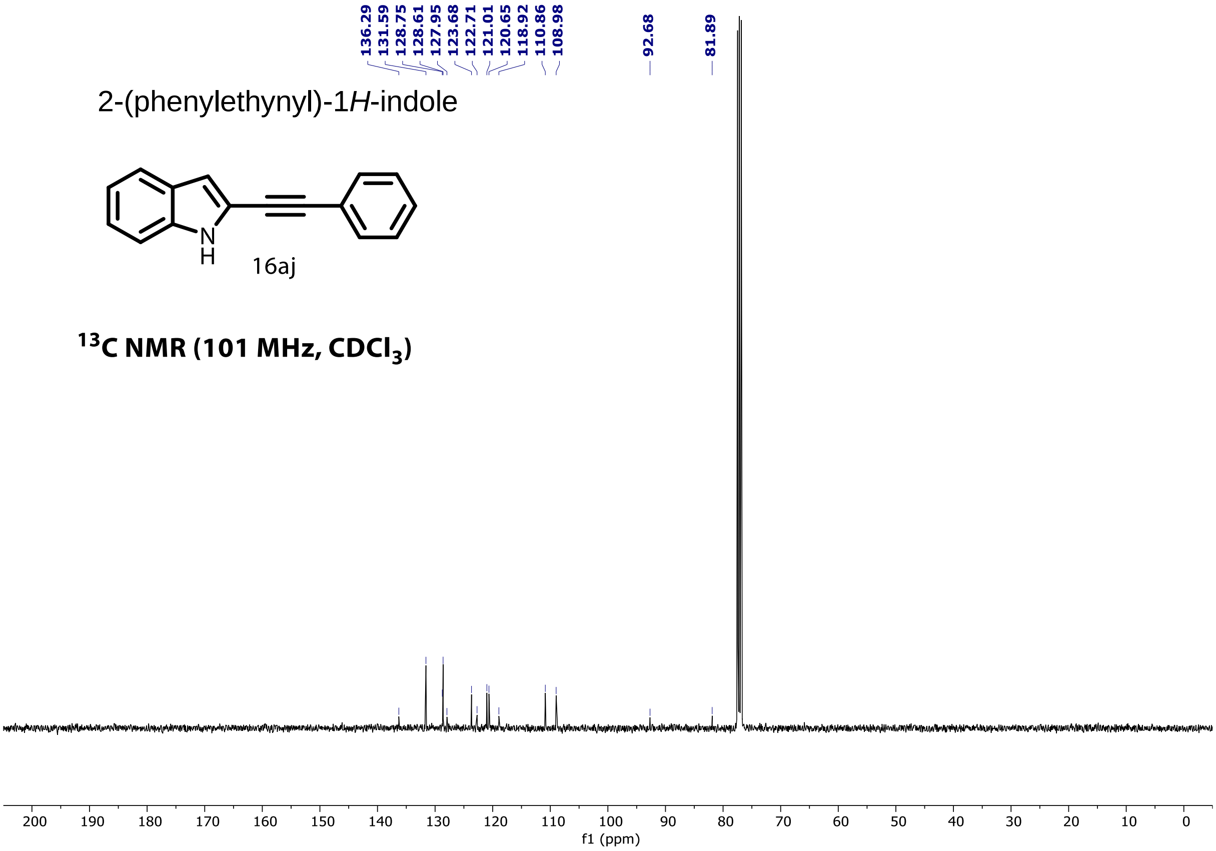
136.29
131.59
128.75
128.61
127.95
123.68
122.71
121.01
120.65
118.92
110.86
108.98
92.68
81.89
2-(phenylethynyl)-1H-indole
N
H
16aj
13C NMR (101 MHz, CDCl3)
200
190
180
170
160
150
140
130
120
110
100
90
80
70
60
50
40
30
20
10
0
f1 (ppm)

(*Z*)-2-(2,5-diphenylpent-1-en-1-yl)-1-methyl-1*H*-indole

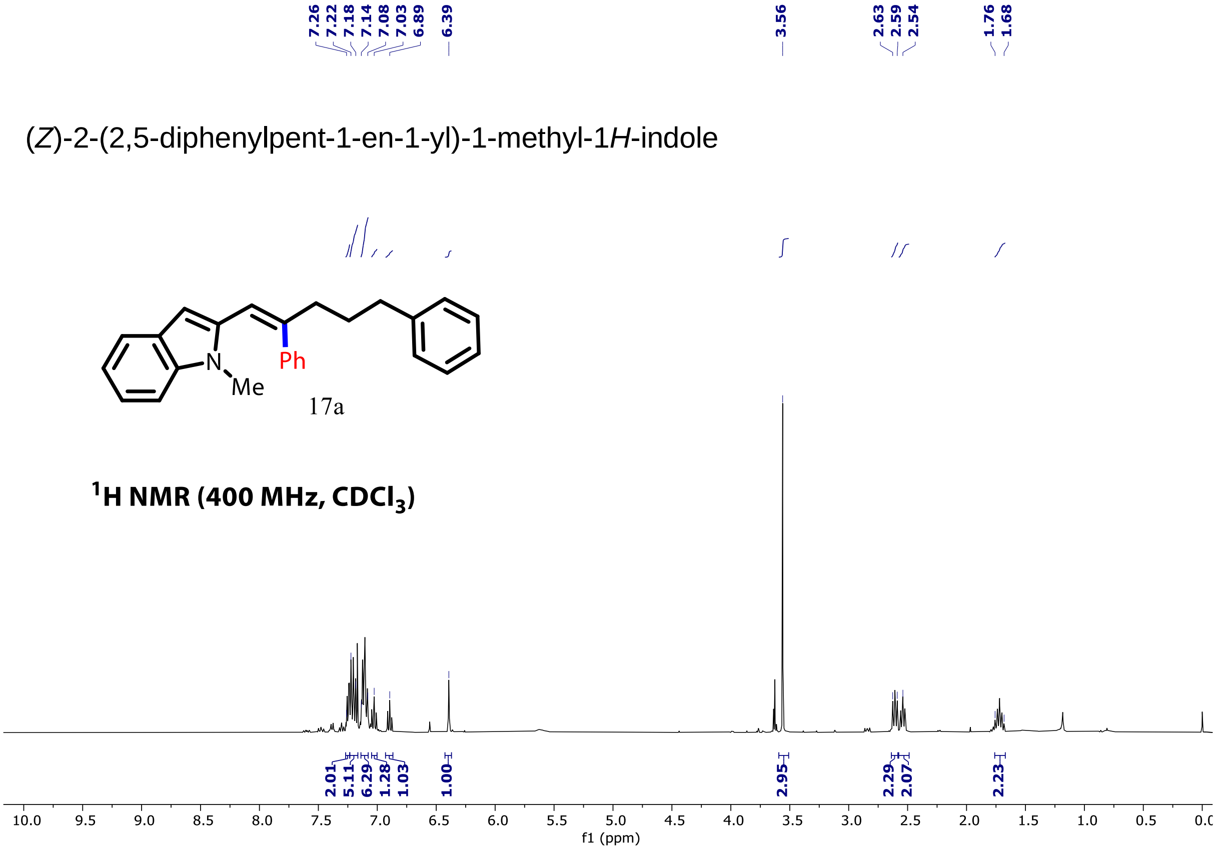

(*Z*)-2-(2,5-diphenylpent-1-en-1-yl)-1-methyl-1*H*-indole

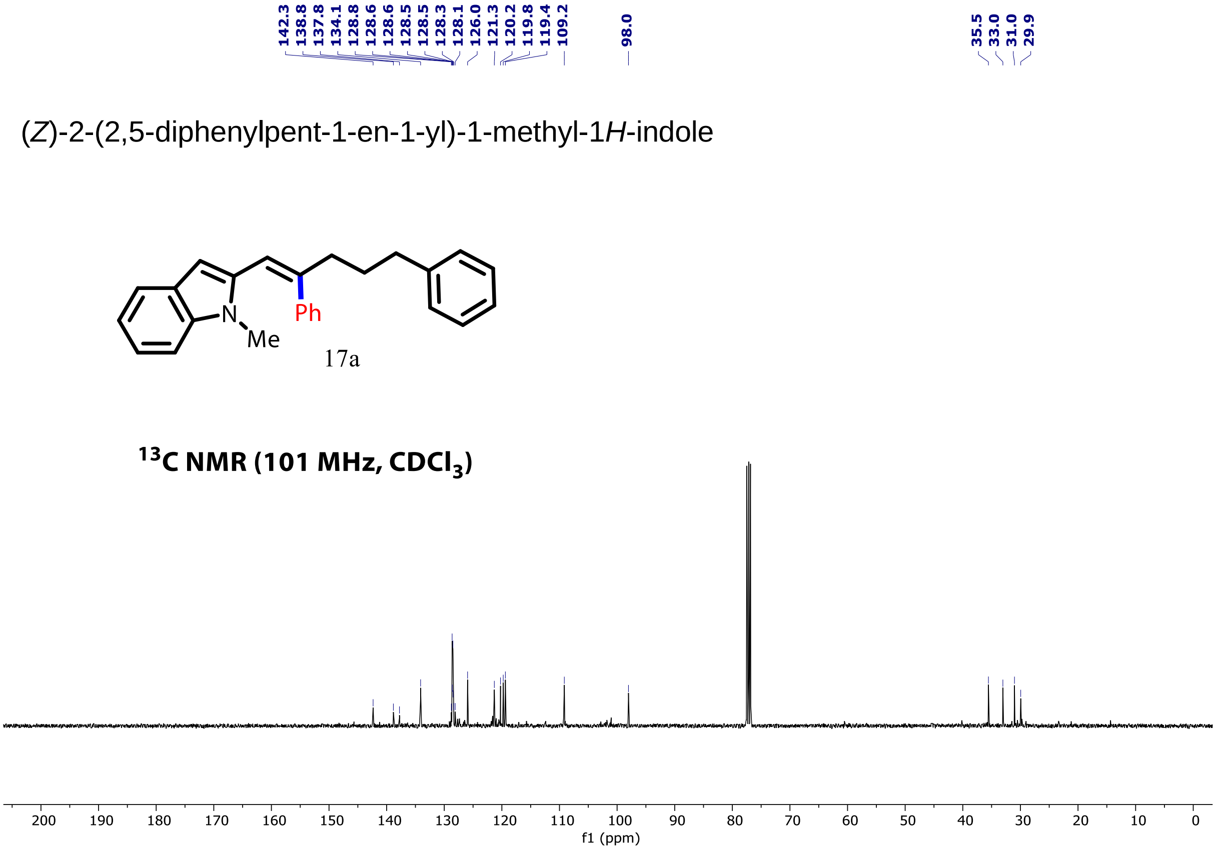

Supplement: QO-012-D5QO00521C-s001 [file QO-012-D5QO00521C-s001.pdf]
